# Transition Metal Nitrides: Multifunctional Catalysts and Energy Materials with Tailorable Architectures

**DOI:** 10.1002/smsc.202500331

**Published:** 2025-08-08

**Authors:** Narasimharao Kitchamsetti, Ana L.F. de Barros, Sungwook Mhin

**Affiliations:** ^1^ National & Local United Engineering Laboratory for Power Batteries Faculty of Chemistry Northeast Normal University Changchun 130024 P. R. China; ^2^ Narayana Engineering College Nellore Andhra Pradesh 524004 India; ^3^ Laboratory of Experimental and Applied Physics Centro Federal de Educação Tecnológica Celso Suckow da Fonseca Av. Maracanã Campus 229 Rio de Janeiro 20271‐110 Brazil; ^4^ Department of Energy and Materials Engineering Dongguk University Seoul 04620 Republic of Korea

**Keywords:** characteristics, dimensions, multifunctional applications, preparation strategies, transition metal nitrides

## Abstract

Transition metal nitrides (TMNs) exhibit notable multifunctionality due to their superior physicochemical and catalytic attributes. This review delineates their crystal architectures, thermodynamic robustness, and inherent merits such as enhanced catalytic activity, sintering resistance, and operational selectivity. Density functional theory analyses have illuminated their electronic, optical, vibrational, plasmonic, mechanical, and morphological characteristics. TMNs, including mono‐, bi‐, and tri‐metallic variants, are synthesized via routes like ammonolysis, chemical vapor deposition, electrodeposition, and pyrolysis. Their structural dimensionality (0D–3D) significantly influences performance, with structural engineering enhancing their functional characteristics. Applications span photocatalysis (hydrogen evolution reaction, oxygen evolution reaction, overall water splitting, hydrogen peroxide production (H_2_O_2_), carbon dioxide reduction (CO_2_RR), and pollutant degradation), electrocatalysis, energy storage (batteries, supercapacitors), and photovoltaics, emphasizing TMNs technological relevance.

## Graphical abstract

This article discusses the preparation methods, structural engineering, and key properties of transition metal nitrides, as well as their versatile applications in supercapacitors, rechargeable batteries, electrocatalytic and photocatalytic processes, and solar cell technologies.

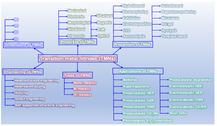

## 1 Introduction

Transition metal nitrides (TMNs) have gained significant attention due to their unique characteristics and broad applications. While crystallography research has advanced considerably, progress is also being made in less‐explored areas. However, fragmented knowledge, scattered across journals, reports, and conferences, makes it challenging for researchers to grasp the field holistically.^[^ ^1^, ^2^, ^3^ ^]^ This can lead to overlooked breakthroughs or difficulties in interpreting new findings.^[^ ^4^, ^5^, ^6^ ^]^ TMNs are formed by introducing nitrogen (N) into TM lattices, resulting in high melting points, mechanical strength, elasticity, and excellent conductivity.^[^ ^7^, ^8^, ^9^ ^]^ These traits make them valuable in catalysis, energy storage, and materials science. Notably, earth‐abundant TMNs emerge as affordable alternatives to platinum (Pt)‐group metals (PGMs), with improved catalytic performance.^[^ ^10^ ^]^ Their efficiency depends on nitride phase, surface structure, defects, and reaction mechanisms.^[^ ^11^, ^12^, ^13^ ^]^ TMNs are effective in reactions like ammonia synthesis, decomposition, and hydrodenitrogention (HDN),^[^ ^14^, ^15^ ^]^ with reactivity linked to their electronic and structural characteristics. Interestingly, due to similar electronegativities and crystal structures between carbon (C) and N, TMNs often behave like transition metal carbides (TMCs). For example, Group 6 carbides, with an extra electron, resemble Group 5 nitrides in function.^[^ ^16^, ^17^, ^18^ ^]^

TMNs have been extensively employed as catalysts in diverse chemical processes, such as hydrogenolysis, methane dry reforming (CH_4_ + CO_2_ → 2H_2_ + 2CO), plastic depolymerization, and biomass conversion. Their catalytic performance, particularly when compared to TMCs, is strongly dependent on precursor selection and reaction conditions.^[^ ^19^, ^20^, ^21^ ^]^ For instance, molybdenum nitride (Mo_2_N) exhibits higher activity than molybdenum carbide (Mo_2_C) in acetone hydrolysis due to its greater abundance of basic sites, whereas MoC_*x*_ outperforms Mo_2_N in biomass deoxygenation reactions.^[^ ^22^ ^]^ Additionally, in Fischer–Tropsch synthesis, the catalytic properties of both nitrides and carbides are influenced by the choice of elemental precursors. Although TMNs, TMCs, and base TMs are frequently examined together, their divergent chemical reactivities and morphological characteristics highlight the need for customized strategies in their application to chemical synthesis and renewable energy technologies.^[^ ^23^ ^]^

**Scheme 1:**
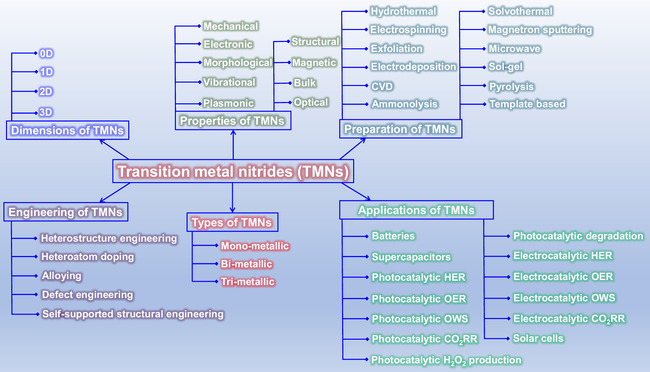
A graphical representation summarizes the key features discussed in this review.

This review provides a systematic and critical synthesis of TMNs, addressing their crystallographic configurations, thermodynamic stability, and intrinsic advantages. TMNs are distinguished by their superior catalytic activity, diminished sintering susceptibility, and enhanced selectivity under mild thermal conditions. DFT analyses offer foundational insights into their multifunctional characteristics, spanning electronic, optical, vibrational, plasmonic, mechanical, bulk, magnetic, and structural–morphological attributes. A classification framework is presented, organizing TMNs into mono‐, bi‐, and tri‐metallic systems, alongside an evaluation of synthesis techniques (e.g., ammonolysis, CVD, electrodeposition, and pyrolysis). The discussion underscores how structural engineering governs their functional performance. Further, the review investigates the role of dimensionality and morphology, including nanoflowers (NF), nanospheres (NS), and nanowires (NWs), in modulating material behavior. Applications are comprehensively analyzed, covering photocatalytic processes (HER, OER, OWS, CO_2_RR, and pollutant degradation), electrocatalytic systems (HER, OER, OWS, CO_2_RR), and energy storage technologies (batteries, SCs), as well as solar energy conversion. By unifying functional characteristics, structural diversity, and dimensional design principles, this review establishes a conceptual framework to guide future TMN‐based material innovation. **Scheme** **1** schematically encapsulates the review's scope and key contributions.

### 1.1 Crystal Structure

**Figure 1:**
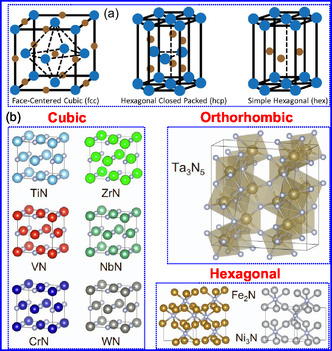
a) Common TMN crystal structures. Adapted with permission.^[^ ^27^ ^]^ Copyright 2016, Wiley‐VCH. b) The crystal structures of TiN, VN, CrN, ZrN, NbN, and WN. Adapted with permission.^[^ ^40^ ^]^ Copyright 2023, Elsevier B.V.

TMNs are classified as interstitial compounds characterized by the incorporation of N atoms into the octahedral or tetrahedral voids of host metal lattices. This interstitial solid solution behavior yields densely packed configurations that markedly enhance electrical conductivity relative to their parent metals.^[^ ^24^, ^25^, ^26^ ^]^ Crystallographic analyses reveal that TMNs predominantly adopt hexagonal (HEX), hexagonal close‐packed (HCP), and face‐centered cubic (FCC) symmetries (**Figure** **1**a),^[^ ^27^ ^]^ where N atoms occupy interstitial positions without disrupting the metallic framework. The resultant crystal phase is critically determined by the metal‐to‐N (M/N) stoichiometric ratio, with both electronic band structure and geometric constraints governing phase stability.^[^ ^28^, ^29^, ^30^ ^]^ Geometric modeling demonstrates that simple closed‐packed structures (FCC, HCP, and HEX) form when the nonmetal‐to‐metal radius ratio (r_N_/r_M_) is <0.59, whereas higher ratios promote complex lattice geometries. Structural integrity is contingent upon the spatial compatibility between interstitial sites and N atoms; suboptimal matching results in weakened metal‐N bonding and thermodynamic instability.^[^ ^31^, ^32^, ^33^ ^]^ The electrocatalytic characteristics of TMNs are governed by an interplay of crystallographic configuration and electronic structure. Nonstoichiometric deviations from a 1:1 M/N ratio generate vacancy defects that modify the lattice environment and catalytic efficacy. Metal‐rich phases (M/N > 1) frequently demonstrate superior catalytic activity owing to preserved metallic states and modified d‐band centers.^[^ ^34^, ^35^, ^36^ ^]^ Phase formation trends can be rationalized through the valence electron‐to‐atom ratio (e/A), which predicts the stability of body‐centered cubic (BCC), HCP, or FCC arrangements. Notably, the formation of directional dp‐hybridized bonds between metal d‐orbitals and N p‐orbitals elevates the e/A ratio in TMNs compared to pure metals, a conclusion supported by X‐ray photoelectron spectroscopy (XPS) analyses of electronic structure modifications.^[^ ^37^, ^38^, ^39^ ^]^

Peng and colleagues^[^ ^40^ ^]^ systematically investigated the crystallographic characteristics of TMNs to establish structure‐property relationships governing their performance in nitride decomposition and ammonia (NH_3_) synthesis under acidic conditions. Their study revealed that prototypical TMNs (TiN, VN, CrN, ZrN, NbN, and WN) crystallize in a cubic rock‐salt (NaCl‐type) structure (Fm3m space group), whereas Ta_3_N_5_ adopts an orthorhombic (*ortho*‐) phase (Cmcm space group) consisting of edge‐ and corner‐sharing TaN_6_ octahedra, with Ta cations exhibiting both tri‐ and tetracoordinated configurations with N ligands. Comparative structural analysis demonstrated that Fe_2_N and Ni_3_N assume HEX symmetry, belonging to the P31m and P6322 space groups, respectively. In these systems, each N atom occupies octahedral interstices coordinated by six metal atoms in distorted geometries, though with distinct coordination environments: Fe cations display tri‐coordinate bonding with N, while Ni cations adopt bi‐coordinate configurations (Figure 1b). These structural observations collectively classify TMNs as interstitial compounds, wherein N incorporation into metallic host lattices generates densely packed configurations with enhanced electrical conductivity. The catalytic performance of TMNs is fundamentally governed by two interrelated factors: 1) the M/N stoichiometric ratio, which determines electronic structure modifications, and 2) the specific atomic arrangement within the crystal lattice. Metal‐rich phases (M/N > 1) consistently exhibit superior electrocatalytic activity, attributed to preserved metallic character and modified d‐band electronic states. This behavior originates from dp‐orbital hybridization between transition metal d‐states and N p‐orbitals, which generates the distinctive electronic configurations characteristic of TMNs.

### 1.2 Stability

The structural and functional stability of TMNs constitutes a fundamental determinant of their operational efficacy across diverse technological applications.^[^ ^41^, ^42^, ^43^, ^44^ ^]^ These materials demonstrate exceptional resistance to chemical degradation, oxidative processes, and thermomechanical stress, establishing their suitability for deployment in extreme operational environments including high‐temperature systems, protective surface coatings, and advanced cutting implements. From a catalytic perspective, this stability assumes particular significance in processes such as the HER and oxygen reduction reaction (ORR), where maintenance of active surface sites and resistance to performance degradation are critical operational parameters.^[^ ^45^, ^46^, ^47^, ^48^ ^]^ The remarkable stability of TMNs extends beyond catalytic applications, with their inherent mechanical durability and sustained electronic conductivity enabling effective integration in electronic components, energy storage architectures, and industrial machinery.^[^ ^49^, ^50^, ^51^ ^]^ The stability profile of TMNs manifests through multiple advantageous characteristics: 1) inhibition of deleterious phase transformations under operational conditions, 2) mitigation of oxidative degradation pathways, and 3) preservation of structural integrity throughout prolonged service periods. These attributes collectively contribute to enhanced manufacturability, operational reliability, and performance consistency during both synthesis protocols and practical implementation.^[^ ^52^ ^]^

**Figure 2:**
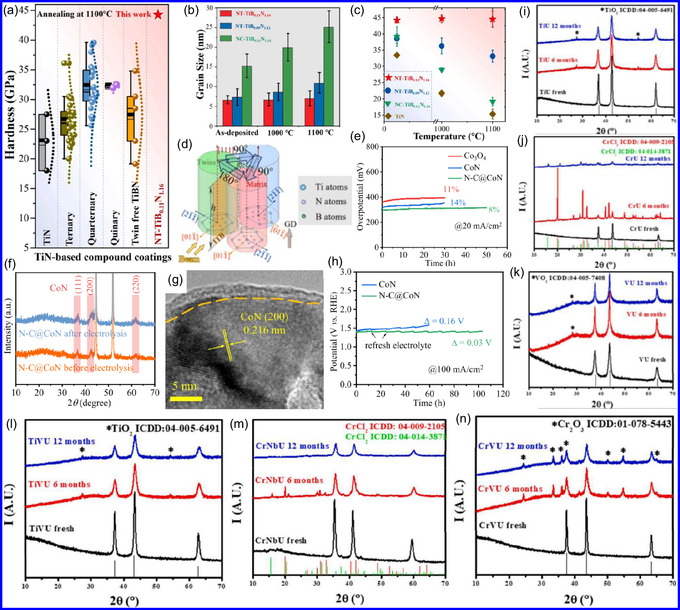
a) TiN coatings exhibit stable performance at 1100 °C, with minimal degradation. b) The average grain size, c) nanoindentation hardness, and d) columnar grain alignment of TiB_0.11_N_1.16_ coatings were systematically characterized. Adapted with permission.^[^ ^53^ ^]^ Copyright 2024, Elsevier B.V. e,h) Thermal stability assessments, f) XRD characterization, and g) HR‐TEM of N‐C@CoN. Adapted with permission.^[^ ^54^ ^]^ Copyright 2024, Elsevier B.V. i–n) XRD patterns of fresh samples compared to specimens aged 6 and 12 months under ambient conditions. Adapted with permission.^[^ ^55^ ^]^ Copyright 2024, Elsevier B.V.

Yang's team^[^ ^53^ ^]^ demonstrated that a nanotwinned TiB_0.11_N_1.16_ ceramic coating exhibits exceptional thermal stability, retaining both its high twin density and grain morphology following prolonged exposure to 1100 °C for 1 h (**Figure** **2**a). Remarkably, the material maintained a hardness of 44.5 GPa under these conditions, significantly exceeding the performance of both twin‐free TiB_0.11_N_1.16_ and conventional TiN‐based coatings subjected to equivalent thermal treatment. Microstructural analysis revealed that this enhanced stability originates from three key factors: 1) boron (B) segregation at grain boundaries, 2) the coexistence of coherent and incoherent twin boundaries, and 3) constrained crystallographic orientation variations within individual grains (Figure 2b,c). This study provides critical insights into the stabilizing role of nanotwin architecture in ceramic materials under thermal stress. While conventional high‐angle grain boundaries typically promote energy accumulation and thermal instability, the dense nanotwin network in this system effectively mitigates such degradation mechanisms. The cross‐sectional microstructure examination revealed randomly oriented nanotwins that collectively suppress grain growth during thermal processing through mutual mechanical constraint. This twin‐mediated stabilization mechanism operates via two complementary pathways: 1) inhibition of individual twin formation and 2) restriction of multidirectional grain coarsening (Figure 2d). Consequently, the coating maintains its structural integrity under extreme thermal conditions. This work establishes an innovative materials design paradigm for achieving thermal stability through deliberate twin‐structure engineering and grain boundary modification, offering significant implications for the development of next‐generation high‐temperature ceramic coatings.

Tang and collaborators^[^ ^54^ ^]^ evaluated the electrochemical stability of a N‐doped C‐coated CoN catalyst (N‐C@CoN), with particular emphasis on its exceptional cycling durability. The investigation revealed that the N‐C@CoN hybrid system maintained stable catalytic performance over 50 h of continuous operation, exhibiting merely an 8% increase in overpotential, a significant improvement compared to both Co_3_O_4_ and unmodified CoN reference catalysts under identical testing conditions (Figure 2e). The enhanced stability was mechanistically attributed to the conformal N–C coating, which effectively suppressed both morphological degradation and deleterious phase transformations during prolonged electrolysis. Comprehensive structural characterization through XRD (Figure 2f) and HR‐TEM (Figure 2g) confirmed the preservation of crystalline integrity and surface chemical composition following extended electrochemical testing. Notably, surface modification via urea plasma treatment was demonstrated to enhance the catalyst's textural characteristics, resulting in a 32% increase in electrochemical specific surface area (SSA) (ECSA) and corresponding improvements in catalytic performance metrics. Accelerated durability testing under industrially relevant conditions (100 mA cm^−2^) further confirmed the system's robustness, with N‐C@CoN maintaining stable operation for >100 h, a fourfold enhancement over bare CoN catalysts (Figure 2h). The study establishes that the N–C coating serves dual protective functions: 1) preventing oxidative dissolution of active CoN species and 2) maintaining structural integrity during redox cycling.

Li's team^[^ ^55^ ^]^ established a synthetic protocol for fabricating noble metal‐containing multimetallic nitride nanocatalysts and systematically evaluated their chemical stability under electrocatalytic operating conditions. The comparative stability analysis revealed a distinct advantage of bimetallic systems over monometallic analogs, with significantly improved resistance to chemical degradation. Accelerated aging studies through controlled air exposure (6–12‐month duration) demonstrated: 1) monometallic nitrides (TiU, NbU, and VU) progressively oxidized to tetragonal oxide phases; 2) bimetallic compositions (TiNbU and TiVU) maintained structural integrity without requiring C passivation; and 3) CrU underwent complete decomposition to CrCl_2_/CrN phases, while bimetallic CrVU and CrNbU preserved their original rock‐salt structure. 4) Time‐dependent chloride depletion in CrU/CrNbU systems (Figure 2i–n), attributed to hygroscopic behavior. These findings establish critical structure‐stability relationships, emphasizing the necessity of optimizing: 1) metal composition selection, 2) electrolyte pH compatibility, and 3) environmental exposure protocols. The long‐term operational stability of TMNs emerges as a fundamental requirement for practical implementation, particularly in demanding applications such as HER and ORR electrocatalysis. Two complementary stabilization approaches were demonstrated to be effective: 1)nanostructural engineering through twin boundary formation and B segregation and 2) surface modification via protective C coatings. This work provides fundamental insights into degradation mechanisms while establishing design principles for developing durable TMN‐based electrocatalytic systems. The stability enhancement strategies offer promising pathways for extending catalyst lifetimes in real‐world electrochemical applications.

### 1.3 Advantages

TMNs offer a range of distinct advantages over conventional transition metal oxides (TMOs) in oxidation reactions, which can be outlined as follows:

#### 1.3.1 Superior Catalytic Activity

**Figure 3:**
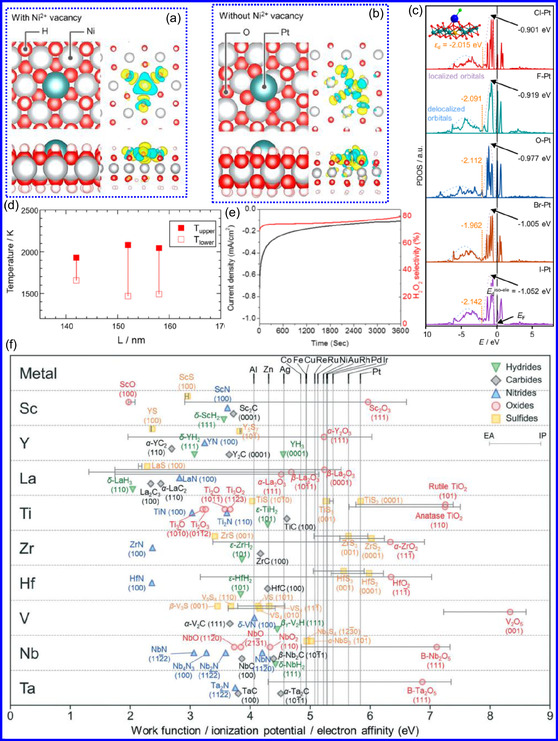
a,b) Charge density differential plots reveal enhanced Pt adsorption on Ni(OH)_2_ with or without Ni^2+^ vacancies. c) PDOS analysis demonstrates ligand‐field‐modified Pt 5d‐band electronic structures in SACs anchored on NiFe‐LDH substrates. Adapted with permission.^[^ ^58^ ^]^ Copyright 2023, American Chemical Society. d) Critical temperature thresholds for photothermally‐induced structural deformation in plasmonic NCs. Adapted with permission.^[^ ^61^ ^]^ Copyright 2021, AIP Advances. e) Stability test assessments of the Pt SACs. Adapted with permission.^[^ ^62^ ^]^ Copyright 2016, Wiley‐VCH. f) First‐principles calculations reveal temperature‐dependent wave function localization in TMNs. Adapted with permission.^[^ ^65^ ^]^ Copyright 2022, Royal Society of Chemistry.

TMNs exhibit enhanced catalytic activity relative to TMOs, primarily due to their unique electrical and structural characteristics. Unlike TMOs, which primarily exhibit ionic and covalent bonding, TMNs incorporate a diverse range of covalent, ionic, and metallic bonds.^[^ ^35^, ^56^, ^57^ ^]^ The formation of metal‐N (M–N) bonds in TMNs modifies the original crystal architecture of the metals, resulting in D‐band contraction and an elevated density of electronic states (DOS) near the Fermi level. This change enables TMNs to mimic noble metal behavior in electrocatalytic systems (**Figure** **3**a–c).^[^ ^58^ ^]^ Enhanced electronic configurations facilitate stronger adsorption between the catalyst surfaces and reactants by increasing the number of accessible active sites. These characteristics not only accelerate reaction kinetics but also highlight TMNs as promising candidates for energy conversion and storage technologies.^[^ ^24^ ^]^ In summary, the superior catalytic performance of TMNs as compared to TMOs can be attributed to their unique M–N bonding, which significantly boosts the DOS near the Fermi level, enhances reactant adsorption, promotes rapid reaction kinetics, and improves overall catalytic efficiency. These features emphasize the potential of TMNs for advanced applications in electrochemical energy conversion and storage systems.

#### 1.3.2 Sintering Sensitivity

Sintering, characterized by the agglomeration and growth of catalyst particles at elevated temperatures, leads to a reduction in both SSA and catalytic efficiency. Although TMNs are generally more susceptible to sintering than TMOs, they exhibit considerable resistance under specific conditions.^[^ ^59^, ^60^ ^]^ Empirical studies indicate that TMNs such as VN and TiN preserve their structural integrity and SSA even at high temperatures, primarily due to the strength of their M–N bonds. These robust bonds enable a resilient lattice that can endure extreme thermal conditions without significant structural degradation. At the nanoscale, where materials often undergo phase relaxation prior to melting, TMNs demonstrate unexpectedly high thermal stability.^[^ ^11^ ^]^ For instance, epitaxially grown TiN films measuring 80 nm on Al_2_O_3_ substrates remain stable at temperatures up to 1400 °C, exhibiting minimal grain growth under vacuum conditions (Figure 3d).^[^ ^61^ ^]^ Similarly, ZrN nanocubes (NCs) maintain stability across a broad thermal spectrum (1127–1827 °C), outperforming Au nanoparticles (NPs). Additionally, TiN, ZrN, and HfN NPs show negligible sintering when subjected to temperatures of 1000 °C in an Ar atmosphere. These results highlight the exceptional sintering resistance and catalytic durability of TMNs, especially in extreme operational environments.^[^ ^61^ ^]^

#### 1.3.3 Selectivity

The incorporation of N into TMNs significantly impacts oxidation reactions by enhancing selectivity and serving as both a promoter and structural modifier. This influence arises from the distinctive surface chemistry of TMNs, which facilitates the selective adsorption and activation of reactive species, thus directing reactions toward preferred oxidation intermediates.^[^ ^35^ ^]^ N doping modifies the electronic characteristics of the metal framework, affecting charge transfer, the hybridization of metal d‐orbitals with nonmetal sp‐states, and causing lattice expansion. These modifications improve the catalytic behavior of TMNs, aligning their adsorption characteristics with those of noble metals. Yang and collaborators^[^ ^62^ ^]^ examined the ORR using Pt single‐atom catalysts (SACs) supported on TMNs, employing a rotating ring disk electrode (RRDE) in acidic conditions. Their findings revealed that Pt atoms at specific surface sites favored a two‐electron pathway, achieving high selectivity for H_2_O_2_. A Pt/TiN catalyst with a Pt loading of 0.35 wt% exhibited a 65% selectivity for H_2_O_2_, albeit with a lower ORR current. In contrast, catalysts featuring lower Pt loadings (0.05 and 0.1 wt%) achieved up to 90% selectivity for H_2_O_2_, although they displayed reduced ORR activity due to a limited number of active sites (Figure 3e). Overall, TMNs enhance selectivity in various catalytic applications, including hydrotreatment, CO oxidation, alcohol reforming, and cinnamaldehyde hydrolysis, rendering them economically viable for selective oxidation. Furthermore, stability tests indicated that Pt SACs on TMNs sustained their H_2_O_2_ selectivity over time while experiencing minimal activity loss. These results highlight the promise of TMN‐supported SACs in stable and efficient selective oxidation processes.

#### 1.3.4 Lower Operating Temperatures

TMNs offer a unique catalytic advantage due to their capacity to facilitate oxidation reactions at lower temperatures compared to traditional TMOs. This advantage is attributed to the incorporation of N atoms, which effectively reduce the activation energy (AE) of catalytic processes.^[^ ^63^ ^]^ A 2022 investigation into the work functions (WFs) of various material classes, including TMOs, TMCs, TMNs, sulfides, and hydrides, revealed that TMNs have the lowest WFs, typically ranging from 2.25 to 4.25 eV.^[^ ^64^ ^]^ In contrast, noble metals like Ag and Au demonstrate significantly higher WFs, at 4.6 and 5.4 eV, respectively, while TMOs can reach values as high as 8.5 eV (Figure 3f).^[^ ^65^ ^]^ The lower WF in TMNs directly translates to reduced AE requirements, enabling reactions to occur at milder temperatures. This characteristic offers multiple advantages, including decreased energy consumption, minimized risk of high‐temperature side reactions, reduced operational costs, and improved catalyst longevity.^[^ ^65^ ^]^ Thus, TMNs represent efficient, stable, and cost‐effective catalytic materials for oxidation processes conducted under moderate thermal conditions.

### 1.4 Theoretical Insights and Role of DFT in TMNs Research

DFT is an essential computational method for elucidating and optimizing the characteristics of TMNs, particularly within the fields of catalysis and materials science. By conducting an exhaustive electronic structure analysis, DFT provides insights into how N incorporation affects critical characteristics, including the DOS, band structure, and WFs, all of which are fundamental determinants of catalytic activity.^[^ ^66^ ^]^ Furthermore, DFT enables the assessment of various catalytic parameters, such as efficiency, selectivity, and stability, under different environmental conditions. This capability aids in the rational design of high‐performance and durable TMN‐based catalysts. Additionally, DFT is crucial for predicting surface characteristics, magnetic properties, and electronic behavior, thereby providing theoretical frameworks that minimize dependence on extensive experimental trial‐and‐error procedures.^[^ ^67^ ^]^

**Figure 4:**
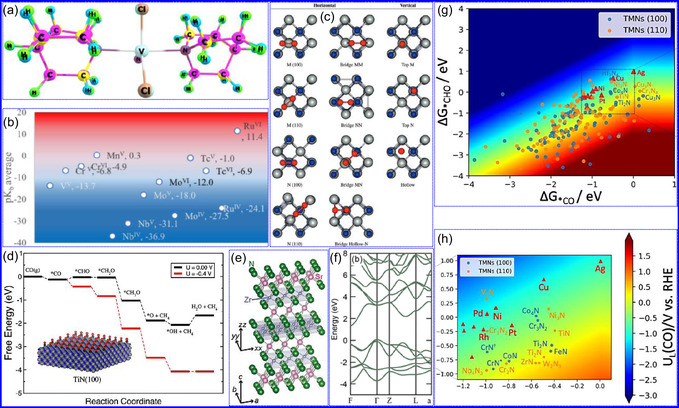
a) DFT‐optimized VCl_2_(quinuclidine)_2_(N) structures exhibit minimal geometric deviation (RMSD = 0.09 Å), b) Linear regression (R^2^ = 0.98) compares pK_b_ values across 28 CrN complexes calculated at B3LYP/def_2‐SVP_ vs B3LYP/6‐311 + G(d,p) levels with SMD(acetonitrile) solvation. Adapted with permission.^[^ ^68^ ^]^ Copyright 2022, American Chemical Society. c) DFT‐optimized adsorption configurations of O_2_ on MN(001) reveal both horizontal and vertical binding modes, with M (gray), N (blue), and O (red) atoms color‐coded for clarity. Adapted with permission.^[^ ^69^ ^]^ Copyright 2008, American Chemical Society. d) The free energy diagram for CO_2_RR on TiN(100) shows energetically favorable steps at both 0 V (black) and −0.4 V vs. RHE (red), with all intermediates stabilized under applied potential. Adapted with permission.^[^ ^70^ ^]^ Copyright 2020, American Chemical Society. e) SrZrN_2_ crystallizes in the α‐NaFeO_2_ type layered structure, while f) DFT calculations reveal a direct BG of 1.8 eV at Γ‐point with hybridized Zr‐4d/N‐2p orbitals dominating the valence band maximum. Adapted with permission.^[^ ^71^ ^]^ Copyright 2014, American Chemical Society. g,h) DFT‐calculated adsorption free energies (ΔG_*CO_ vs ΔG_*CHO_) plotted as contour maps reveal distinct activity trends across TMNs (circles), with optimal intermediates binding near the volcano peak. Adapted with permission.^[^ ^72^ ^]^ Copyright 2023, American Chemical Society.

In a comprehensive investigation, Alharbi's group^[^ ^68^ ^]^ utilized DFT to assess the basicity (pK_b_(N)) of 3d and 4d TMNs, focusing on elements such as V, Cr, Mn, Nb, Mo, and Ru. Their methodology consisted of calculating intrinsic protonation energies while examining the influence of various factors, including metal identity, oxidation state, coordination number, and ligand presence, on basicity. A noteworthy observation was the root mean square deviation (RMSD) of atomic positions, indicating a high degree of structural consistency across different basis sets (**Figure** **4**a). The findings revealed a general decreasing trend in basicity from left to right across the 3d and 4d series, as well as an increase in basicity from 3d to 4d elements. While the trends in basicity are primarily governed by metal identity and oxidation state, ligand effects also play a significant role. Multiple basis sets were evaluated for their effectiveness in predicting pK_b_ values for 128 nitride complexes, with the def2‐svp set striking an optimal balance between accuracy and computational efficiency (Figure 4b). This study highlights the intricate interplay of electronic and structural parameters that influence the catalytic properties of TMNs.

Graciani and colleagues^[^ ^69^ ^]^ performed an extensive investigation into the fundamental oxidation mechanisms of early TMNs, specifically ScN, TiN, and VN. The results indicated that O_2_ adsorption and subsequent dissociation on these nitrides surfaces are generally exothermic; however, the oxidation behavior exhibits significant differences between ScN and the other two materials. In the cases of TiN and VN, the oxidation process is predominantly controlled by O‐metal interactions, whereas ScN demonstrates substantial participation of surface N atoms. This distinction is understood through the variations in electronic structure, particularly the density of 3d electrons in proximity to the Fermi level. The study identified two major oxidation pathways: 1) the formation of NO through N–O recombination, which is thermodynamically unfavorable and likely to occur only at elevated temperatures, and 2) the removal of N atoms, which is thermodynamically favorable yet limited by a high O‐to‐N surface ratio. Moreover, the incorporation of O into the bulk of these materials necessitated heat input across all cases. These findings elucidate the reasons why the oxidation of TiN and VN generally require high temperatures, leading to the formation of TMOs (MO_*x*_), rather than mixed oxynitrides (MN_*x*_O_*y*_), suggesting that oxidation is not a viable pathway for synthesizing mixed oxynitrides (Figure 4c).

Karamad and his team^[^ ^70^ ^]^ performed a DFT study to evaluate the catalytic performance and electrochemical stability of heteroatom‐doped TMNs in the context of CO_2_RR. Their investigation encompassed various polymorphs doped with 25% concentrations of elements such as B, P, Sb, Bi, and C, focusing on their resistance to *OH poisoning and structural degradation under electrochemical conditions. Although several TMNs exhibited promising catalytic activity for CO_2_RR, many faced limitations due to competing HER or adverse *OH adsorption effects at the onset potentials of CO_2_RR. Notably, TiN(100) emerged as a highly effective catalyst, demonstrating excellent selectivity while operating at a moderate overpotential of −0.46 V (RHE), outperforming Cu(211) in both selectivity and efficiency (Figure 4d). Importantly, TiN(100) showed high catalytic activity without requiring dopants, establishing it as a benchmark catalyst for this application. The study concluded that methane (CH_4_) is the final product of CO reduction on TiN(100), with the elimination of *OH to form water, rather than the transformation of *CO to *CHO, as the rate‐determining step (RDS). These findings highlight the importance of considering catalytic efficiency, electrochemical stability, and the interactions among CO_2_RR, HER, and *OH reduction in catalyst design.

Ohkubo and colleagues^[^ ^71^ ^]^ conducted a comprehensive study on the electronic band structures and thermoelectric characteristics of complex metal nitrides SrZrN_2_ and SrHfN_2_, which crystallize in a layered α‐NaFeO_2_‐type structure. By employing DFT in conjunction with Boltzmann transport theory, the authors found that despite their layered geometries, these compounds exhibit 3D electronic networks and isotropic charge transport characteristics. This behavior arises from the distinct contributions of Sr and Zr/Hf orbitals within the conduction bands. Moreover, the study predicts that both materials demonstrate considerable potential for thermoelectric applications, as evidenced by their substantial Seebeck coefficients and favorable thermoelectric figures of merit (Figure 4e,f).

Yohannes's team^[^ ^72^ ^]^ performed a comprehensive high‐throughput DFT study that evaluated around 800 TMNs for their potential efficacy as catalysts in CO_2_RR. Through thermodynamic analysis, CoN, CrN, and TiN emerged as the most promising candidates due to their favorable reactivity and stability over time. The researchers also employed machine learning (ML) regression models to analyze key descriptors that affect catalyst stability, discovering a significant correlation between the group number of the metal and the binding energies of *OH. Adsorption energies for *CHO and *CO across various TMNs were systematically mapped (Figure 4g,h), revealing that many TMNs demonstrate a stronger attraction for *CHO, which disrupts the typical linear scaling relationship between adsorbates. This behavior can be attributed to the differing bonding characteristics: *CHO binds through both C and O, while *CO binds exclusively via C. The combination of DFT and ML establishes a robust framework for accelerating the identification of next‐generation energy materials. Overall, the study underscores the pivotal role of DFT in the rational design and enhancement of TMNs for catalytic applications, emphasizing their potential in thermoelectric, oxidation, and CO_2_RR contexts.

### 1.5 Characteristics

TMNs are characterized by a diverse range of properties that critically affect their performance across various applications.^[^ ^73^ ^]^ Their electronic attributes, such as heightened electron concentration and a high DOS near the Fermi level, enhance both catalytic activity and electrical conductivity, essential qualities for catalysis and nanoelectronics. The optical characteristics of TMNs promote efficient light absorption and carrier generation, which are crucial for photocatalytic and optoelectronic applications, including LEDs and photodetectors. Vibrational analyses offer valuable insights into atomic interactions and phase transitions, aiding in structural optimization. Additionally, plasmonic effects enhance local electromagnetic fields, which in turn can improve photocatalytic efficiency; however, vulnerability to oxidation may diminish this benefit. On the mechanical front, TMNs exhibit outstanding thermal stability and hardness, making them suitable for energy storage and flexible device applications. Their strong bulk characteristics ensure durability under extreme thermal and mechanical conditions, positioning them as ideal candidates for industrial and aerospace applications. Magnetic characteristics, influenced by defects and crystal field effects, lead to advanced ferromagnetic behavior. Structurally, TMNs demonstrate morphological flexibility that varies with precursor conditions, enhancing their adaptability in performance. Finally, features such as high SSA and porosity further enhance their functional capabilities in energy storage and photocatalytic systems.^[^ ^74^ ^]^

#### 1.5.1 Electronic Characteristics

The transition from micro‐ to nanoelectronics has significantly propelled the development of efficient nitride nanostructures, particularly those based on TMNs.^[^ ^75^ ^]^ The substitution of N atoms into metal lattice sites enhances electron density at catalytic surfaces, thereby altering the electronic characteristics of the host materials. This substitution results in changes to bond lengths and a contraction of the d‐band, which increases the DOS near the Fermi level, a phenomenon reminiscent of noble metals.^[^ ^76^ ^]^ Additionally, N modifies the bonding characteristics from predominantly ionic to a more covalent or mixed nature. As a result, TMNs display unique characteristics compared to their parent metals, including altered adsorption behaviors and significantly modified lattice parameters. For instance, N incorporation leads to an expansion of the interatomic spacing within various metal lattices. Wide bandgap (BG) semiconductors, such as AlN and GaN, are particularly well‐suited for applications involving high voltage and temperature.^[^ ^77^ ^]^ These materials also showcase high electron availability and localized charge densities, making them ideal for sensing applications. Moreover, Zn_3_N_2_, which possesses a direct BG of 1.4 eV, demonstrates excellent electrical performance characterized by high carrier concentration and low resistivity.

**Figure 5:**
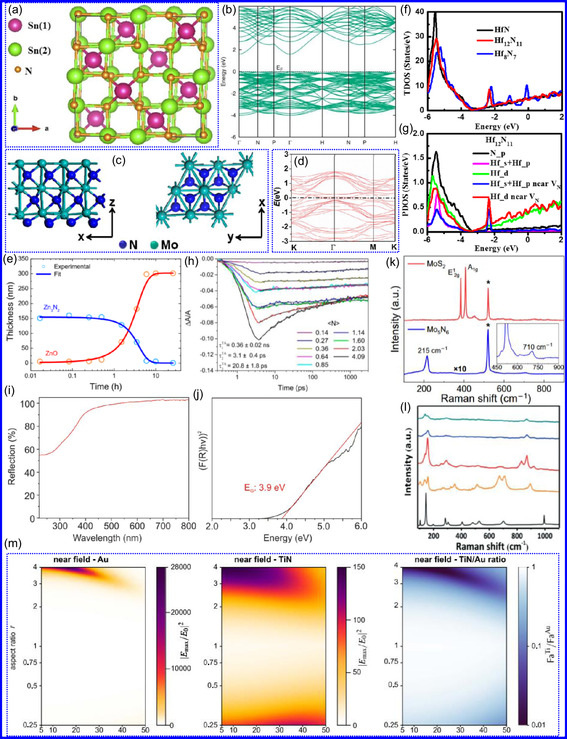
a) Sn_3_N_4_ crystallizes in a cubic spinel configuration and b) first‐principles calculations reveal its characteristic electronic band dispersion. Adapted with permission.^[^ ^79^ ^]^ Copyright 2019, IOP Publishing. c) Atomic architecture: top/side views of 2D MoN honeycomb lattice. d) Electronic fingerprint: DOS reveals metallic Mo‐d dominance. Adapted with permission.^[^ ^83^ ^]^ Copyright 2017, American Chemical Society. e) Zn_3_N_2_ and ZnO layer thicknesses were calculated for samples at 80% rH and 40 °C. Adapted with permission.^[^ ^85^ ^]^ Copyright 2021, American Chemical Society. f,g) DOS and PDOS for Hf_8_N_7_, Hf_12_N_11_, HfN, Hf_11_N_12_, and Hf_7_N_8_. Adapted with permission.^[^ ^86^ ^]^ Copyright 2014, American Chemical Society. h) ΔA/A transients were recorded for fractional absorption changes at a wavelength of 500 nm. Adapted with permission.^[^ ^87^ ^]^ Copyright 2019, American Chemical Society. i) UV‐Vis and j) Tauc plots of GaN NPs. Adapted with permission.^[^ ^88^ ^]^ Copyright 2020, Royal Society of Chemistry. k) Raman analysis of MoS_2_ and Mo_5_N. Adapted with permission.^[^ ^90^ ^]^ Copyright 2020, Science Advances. l) Raman analysis of V_2_O_3_. Adapted with permission.^[^ ^91^ ^]^ Copyright 2016, Royal Society of Chemistry. m) Characteristics of Au and TiN spheroids under light at a wavelength of 800 nm are influenced by both the aspect ratio and equivalent radius. Adapted with permission.^[^ ^95^ ^]^ Copyright 2016, Springer Nature.

Ceramic TMNs, including ZrN, TiN, HfN, and NbN, exhibit significant electronic conductivity and can display varying stoichiometries depending on the preparation conditions.^[^ ^78^ ^]^ Mg_3_N_2_, characterized by its cubic crystalline structure, demonstrates high electrical conductivity due to the hybridization of Mg and N orbitals. Zn_3_N_2_ is notable for its optoelectronic applications, possessing a direct BG that ranges from 1.7 to 2.4 eV (**Figure** **5**a,b),^[^ ^79^ ^]^ with its resistivity subject to modulation through O‐doping. Similarly, Sn_3_N_4_ thin films, produced via reactive sputtering, exhibit n‐type conductivity with resistivity values from 3 to 14 × 10^−2^ Ω cm^−1^. This conduction is attributed to charge carriers induced by N vacancies, which increase under enhanced radio frequency power, contributing to scattering phenomena.^[^ ^80^ ^]^ TMNs with rock‐salt structures, such as ZrN, HfN, and NbN, are also recognized for their superconducting characteristics at elevated transition temperatures and high bulk moduli, indicating their potential in superconductive material design.^[^ ^81^ ^]^ Furthermore, TMNs display catalytic activity comparable to that of noble metals like Pt. For instance, MoN has shown remarkable performance in the HDN of pyridine.^[^ ^82^ ^]^ 2D MoN NSs, synthesized through a salt‐templating approach, exhibit metallic behavior without a discernible BG, demonstrating exceptional conductivity, as confirmed by both experimental and computational studies (Figure 5c,d).^[^ ^83^ ^]^ In conclusion, the electronic characteristics of TMNs significantly enhance their catalytic efficiency and conductive performance. The integration of N increases surface electron concentration narrows the d‐band and elevates the DOS near the Fermi level, thus mimicking noble‐metal activity. Additionally, the bonding character shifts from ionic to covalent or mixed states, altering adsorption dynamics and lattice parameters. These attributes, along with TMNs ability to maintain high carrier densities and low resistivity, establish them as optimal candidates for applications in nanoelectronics, catalysis, sensing, and superconductivity.

#### 1.5.2 Optical Characteristics

Nanostructured materials have attracted substantial attention due to their exceptional photocatalytic performance, primarily attributed to their wide BGs in the UV region. This property enables the effective utilization of visible light, facilitating diverse chemical processes. Upon light absorption, photons excite electrons, resulting in the generation of electron‐hole pairs on the material's surface.^[^ ^76^ ^]^ In the absence of recombination, these charge carriers are separated, leading to the creation of an electric current, a crucial mechanism for applications in solar energy conversion and environmental remediation.^[^ ^84^ ^]^ Optical modeling conducted by Ropero–Real's group reveals that Zn_3_N_2_ single layers possess a porosity of 15%–23%, as determined by void concentration analysis (Figure 5e).^[^ ^85^ ^]^ TMNs from Group IVB, including TiN, ZrN, and HfN, are particularly attractive due to their tunable optical and electronic characteristics, which can be precisely controlled through doping or defect engineering. Notably, variations in the stoichiometry of HfN films enable precise modulation of optical reflectance, as described by the Drude–Lorentz model, which characterizes the response of free electrons to electromagnetic radiation (Figure 5f,g).^[^ ^86^ ^]^ Furthermore, Zn_3_N_2_ colloidal quantum dots (QDs) represent a significant advancement in nanoscale optoelectronics. Their size‐dependent optical behavior, resulting from quantum confinement, allows for the tuning of absorption and emission characteristics. Smaller QDs exhibit larger BGs, making them suitable for applications in the visible and infrared regions. Additionally, their substantial Stokes shift, which is the difference between absorption and emission wavelengths, minimizes reabsorption losses, thereby enhancing the performance of light‐emitting devices (Figure 5h).^[^ ^87^ ^]^

Nanotechnology is increasingly focused on the engineering of materials with precisely tailored BGs to meet specific functional requirements. This customization is often achieved by modifying the morphology of nanomaterials. A prime example of this approach is the preparation of GaN NPs via microwave‐assisted techniques, which exhibit a pronounced blue shift in their BG to 3.9 eV. This shift is attributed to quantum confinement effects, whereby the reduction in particle size alters the electronic structure, resulting in a BG that exceeds that of bulk GaN. The GaN NPs also exhibit strong fluorescence, particularly within the green spectral range, with an emission peak at 523 nm when excited at 274 nm (Figure 5i,j). These characteristics make GaN NPs highly suitable for a wide range of optoelectronic applications, including high‐efficiency LEDs, laser diodes, and biological imaging probes.^[^ ^88^ ^]^ In conclusion, the optical characteristics of TMNs are crucial in enhancing their photocatalytic and optoelectronic properties. Their intrinsically broad BGs in the UV range facilitate effective visible‐light absorption, promoting electron‐hole generation necessary for current production in energy and environmental technologies. Moreover, tunability through doping, stoichiometric control, and quantum confinement, as demonstrated in Zn_3_N_2_ and GaN QDs, allows for precise regulation of optical characteristics, thereby advancing technologies in lighting, detection, and imaging systems.

#### 1.5.3 Vibrational Characteristics

TMNs exhibit distinct vibrational characteristics that differentiate them markedly from their metallic or oxide precursors. These differences are primarily attributed to substantial alterations in crystalline structure during the nitridation.^[^ ^89^ ^]^ A prominent example is the transformation of MoS_2_ into Mo_5_N_6_, which results in significant modifications in vibrational characteristics as observed via Raman spectroscopy. According to research by Cao's group,^[^ ^90^ ^]^ this conversion also affects the material's optical contrast and photonic properties. MoS_2_ is characterized by two dominant Raman modes, A_1g_ and E^1^_2g_, located near 384 and 407 cm^−1^, respectively. After transformation into Mo_5_N_6_, these modes are no longer present; instead, new peaks appear near 215 and 710 cm^−1^ (Figure 5k), indicating a pronounced change in crystal symmetry and bonding environment. The concurrent loss of photoluminescence in Mo_5_N_6_ further supports the occurrence of structural reconfiguration. Raman intensity mapping confirms the absence of MoS_2_‐related signals, suggesting a complete and homogeneous conversion. A similar vibrational evolution is observed in the transition from V_2_O_5_ to VN.^[^ ^91^ ^]^ An intermediate oxide, V_2_O_3_, forms initially, which is subsequently converted into VN. Throughout the reaction, Raman spectra show a decline in V_2_O_5_ and V_2_O_3_ features, while new, distinct vibrational modes characteristic of VN emerge (Figure 5l). Although the final product retains the morphology of the precursor, its vibrational signature is entirely distinct. In conclusion, TMNs undergo profound vibrational changes upon conversion from their precursors, reflecting extensive crystal restructuring and underscoring the unique physicochemical identity of these materials.

#### 1.5.4 Plasmonic Characteristics

Surface plasmonic resonance (SPR) denotes the collective oscillation of conduction electrons at the boundary between a metallic surface and a dielectric medium when stimulated by visible or near‐infrared (NIR) light. When these oscillations are spatially confined to nanoscale domains on the metal surface, the phenomenon is termed localized SPR (LSPR).^[^ ^92^ ^]^ LSPR is particularly noteworthy for its capacity to amplify the local electromagnetic field significantly at the nanoscale.^[^ ^93^ ^]^ This field enhancement is fundamental to a wide array of applications, including biosensing, high‐resolution imaging, and nanoelectronic devices. One of the hallmark advantages of LSPR is its ability to confine light to dimensions smaller than its wavelength, facilitating exceptional sensitivity in detection systems.^[^ ^94^ ^]^ Within this framework, Group IV TMNs, notably HfN, TiN, and ZrN, have emerged as promising substitutes for conventional plasmonic metals such as Au. These TMNs offer similar plasmonic properties while demonstrating superior thermal stability, chemical robustness, and mechanical durability. For example, HfN exhibits tunable plasmonic resonances across the visible spectrum and possesses a high melting point and strong structural integrity, making it well‐suited for demanding operational environments (Figure 5m).^[^ ^95^ ^]^

TiN is recognized for its outstanding plasmonic efficiency, which facilitates strong light absorption and the subsequent generation of high‐energy electrons. These electrons play a pivotal role in photocatalytic reactions, particularly when TiN NPs are combined with TiO_2_ nanostructures, yielding significantly enhanced photocatalytic activity. Notably, TiN NPs enable the solar‐driven reduction of bicarbonate and oxidation of glycerol, underscoring their potential in solar energy harvesting applications.^[^ ^96^ ^]^ Despite these advantages, TiN's performance is hindered by its tendency to oxidize upon air exposure. This oxidation leads to the development of an oxide shell that induces a red shift in the LSPR peak and diminishes its intensity, thereby impairing overall plasmonic performance. Analytical methods such as XPS and STEM/EDS have shown that TiN NPs with reduced N content are particularly susceptible to oxidation.^[^ ^97^ ^]^ To address these limitations, researchers have adopted innovative synthesis techniques, including nonthermal continuous‐flow plasma processes that yield TiN NPs with strong LSPR in the NIR region. Furthermore, surface stabilization strategies using conformal coating have been developed to preserve the integrity and plasmonic functionality of TiN under elevated temperatures.^[^ ^98^ ^]^ The integration of advanced interface engineering, such as plasmonic Schottky (PSI) and Ohmic (POI) interfaces, offers additional optimization pathways. PSIs selectively collect energetic electrons capable of overcoming the Schottky barrier, whereas POIs facilitate the harvesting of both low‐ and high‐energy carriers to improve photocurrent output. Devices employing TiN/TiO_2_ configurations exhibit linear current‐voltage behavior and exceed the performance of comparable Au‐based systems, owing to TiN's favorable Fermi level alignment for efficient interfacial charge transfer.^[^ ^99^ ^]^ In summary, while challenges related to oxidation persist, TiN remains a compelling material for plasmonic and photocatalytic applications, with recent advances in synthesis and stabilization enhancing its operational durability and functional potential.

#### 1.5.5 Mechanical Characteristics

**Figure 6:**
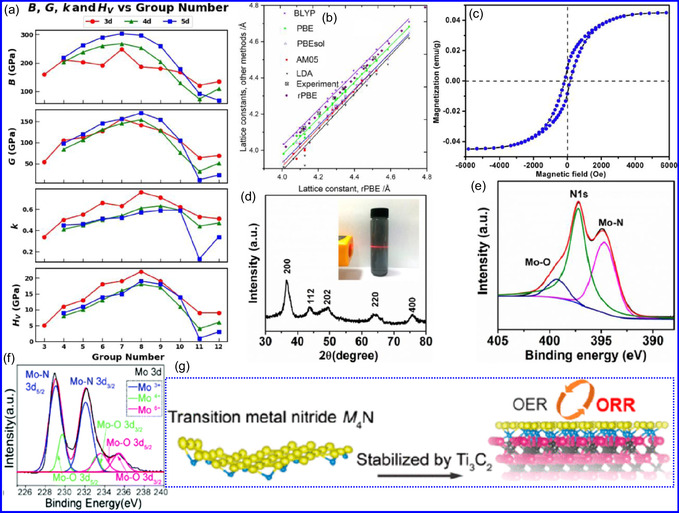
a) Influence of group numbers on B, G, k, and H_*v*_ in mechanically stable MN_6_ materials. Adapted with permission.^[^ ^101^ ^]^ Copyright 2023, Elsevier B.V. b) Lattice constants comparison from LDA, AM05, BLYP, PBE, PBE_sol_, and rPBE methods. Adapted with permission.^[^ ^66^ ^]^ Copyright 2023, Royal Society of Chemistry. c) Magnetization hysteresis loop for AlN. Adapted with permission.^[^ ^110^ ^]^ Copyright 2020, Elsevier B.V. d) XRD of 2D MoN with an inset showing Tyndall phenomenon in water. e) XPS N 1s and Mo 3p spectra of 2D MoN. Adapted with permission.^[^ ^83^ ^]^ Copyright 2017, American Chemical Society. f) XPS analysis of 2D MoN. Adapted with permission.^[^ ^113^ ^]^ Copyright 2021, Royal Society of Chemistry. g) Morphological studies of M_4_N. Adapted with permission.^[^ ^116^ ^]^ Copyright 2020, American Chemical Society.

TMNs are distinguished by their exceptional thermodynamic stability at elevated temperatures, coupled with superior thermal resistance and mechanical hardness, often comparable to or exceeding that of noble metals. These materials feature adjustable carrier concentrations and exhibit superior mechanical robustness, magnetic characteristics, and catalytic efficiency, frequently outperforming conventional precious metals.^[^ ^100^ ^]^ Using DFT, Kandel and colleagues^[^ ^101^ ^]^ evaluated the mechanical characteristics of 29N‐rich TMNs (M:N = 1:6) in the HEX phase, focusing on bulk modulus (B), shear modulus (G), and Vickers hardness (H_*v*_). A consistent trend was observed: B, G, and H_*v*_ values increased with the atomic group number, peaking at Group 8 before declining. Specifically, Group 8 TMNs exhibited peak hardness values of 22 GPa (3d), 18 GPa (4d), and 19 GPa (5d), respectively (**Figure** **6**a). Further confirmation of TMN structural robustness was provided by formation energy (FE) calculations.^[^ ^79^ ^]^ Compounds like Mg_3_N_2_ and Zn_3_N_2_ exhibited negative FEs, supporting their thermodynamic stability. Sn_3_N_4_, prepared via NH_3_‐based nitridation of Sn, displayed an FE of −13.7 eV, affirming its structural integrity.^[^ ^102^ ^]^ Group III nitrides such as AlN, GaN, and InN demonstrated impressive hardness values, 17.7, 10.2, and 11.2 GPa, respectively, attributed to strong covalent bonding and quantum confinement effects in their 2D configurations.^[^ ^103^ ^]^ Mechanical evaluation techniques such as nanoindentation, AFM, and mechanical resonance are typically used for these ultrathin films. Furthermore, computational methods, including DFT and molecular dynamics (MD), underscore the promise of 2D TMNs like WN NSs with W‐terminated surfaces, which combine high mechanical strength and energy efficiency, attributes that suit them for flexible electronics. MoN NSs similarly shows promise as durable, flexible electrodes for high‐performance SCs.^[^ ^104^ ^]^ In conclusion, TMNs offer a unique combination of hardness, thermal resilience, and structural robustness, making them ideal for applications involving extreme environments and flexible technologies.

#### 1.5.6 Bulk Characteristics

Bulk characteristics of TMNs are integral to their functionality in high‐performance and extreme‐environment applications. Their inherent mechanical strength, particularly resistance to compressive and shear stress, enables their deployment in scenarios involving substantial mechanical load.^[^ ^105^ ^]^ TMNs also exhibit exceptional thermal stability, a critical requirement for operation in high‐temperature regimes. These bulk attributes additionally affect key material characteristics, including electrical conductivity, magnetic characteristics, corrosion resistance, and catalytic activity.^[^ ^106^ ^]^ Such a broad spectrum of performance makes TMNs attractive candidates for use in aerospace, energy, and heavy industrial sectors. A detailed understanding and control of these parameters are essential for tailoring TMNs to meet specific application requirements.^[^ ^107^ ^]^ In this regard, Lynn and colleagues^[^ ^66^ ^]^ conducted DFT calculations to investigate the bulk characteristics of TMNs across the 3d to 5d TM series. Six exchange‐correlation functionals, LDA, AM05, BLYP, PBE, rPBE, and PBE_sol_, were employed. Of these, rPBE yielded the most accurate lattice constants in comparison with experimental data. Results from AM05, PBE, rPBE, and PBE_sol_ were found to lie intermediate to those predicted by LDA and BLYP. A strong correlation was observed between lattice constants and FEs, reflecting the influence of atomic radius and electronegativity across the TM series. The study also revealed that the formation of N vacancies is generally more thermodynamically favorable than TM vacancies, with N‐vacancy formation likelihood inversely proportional to the magnitude of FE. This implies that TMNs with more negative FEs possess greater defect resistance (Figure 6b). In conclusion, the bulk, mechanical, thermal, and electronic characteristics of TMNs are foundational to their deployment in advanced technologies, and DFT‐based modeling plays a pivotal role in guiding material design.

#### 1.5.7 Magnetic Characteristics

The magnetic characteristics of TMNs derive from the presence of unpaired d‐electrons in TM atoms, facilitating interactions with external magnetic fields. Phenomena such as ferromagnetism (FM), antiferromagnetism (AFM), and paramagnetism (PM) are governed by atomic configurations, bonding environments, and crystal symmetry. These attributes render TMNs highly applicable in magnetic data storage, spintronics, and catalysis enhanced by magnetic fields.^[^ ^108^ ^]^ Iron nitrides are particularly significant due to their high magnetization and cost‐effective synthesis. A notable achievement includes the rapid preparation of ε‐Fe_3_N_1.33_ using a diamond anvil cell under 5 GPa and 1400 K, triggered by an X‐ray free electron laser (XFEL), yielding a homogeneous phase with enhanced magnetic behavior.^[^ ^109^ ^]^ Similarly, Dy‐doped AlN NSs exhibit room‐temperature FM, attributed to Al vacancies and Dy^3+^ substitution, as evidenced by hysteresis loops measured via vibrating sample magnetometry (Figure 6c).^[^ ^110^ ^]^ Spin state variability was also observed in Fe_8_N monolayers: hollow‐site Fe_8_N exhibited low‐spin FM, while bridge‐site Fe_8_N demonstrated high‐spin FM, driven by variations in 3d orbital splitting induced by local crystal fields. N‐diffusion, with an energy barrier of ≈1.5 eV, facilitates interlayer transitions, influenced by processing conditions and rare‐earth doping.^[^ ^111^ ^]^ Furthermore, DFT studies on ZnSnN_2_ monolayers revealed that Zn or Sn vacancies increase the work function and induce magnetic ordering while maintaining thermal robustness.^[^ ^112^ ^]^ In conclusion, the tunable magnetic characteristics of TMNs, particularly in iron nitrides, doped nitrides, and defective ternary systems, position them as strong candidates for emerging applications in spin‐based electronics and magnetically modulated technologies.

#### 1.5.8 Structural Characteristics

TMNs are commonly synthesized by transforming other 2D materials, such as metal oxides and sulfides, while preserving the intrinsic framework of the metal precursors. For instance, the salt‐template method for synthesizing MoN from MoO_3_ retains the HEX morphology of the original oxide.^[^ ^83^ ^]^ The resultant MoN NSs exhibit a HEX lattice with minimal structural distortion, making them promising candidates for ion implantation in energy storage devices. Similar approaches have been used to synthesize other TMNs, such as W_2_N and V_2_N. While many TMNs do not naturally possess a layered structure, recent advancements, including those by Xiao and collaborators,^[^ ^83^ ^]^ have enabled the synthesis of ultrathin, non‐layered TMNs, such as MoN. In particular, δ‐phase MoN, which adopts a HEX lattice, is often targeted due to its excellent thermodynamic stability and moderate mechanical flexibility (Figure 6d,e). Topochemical synthesis methods have also been applied to achieve cubic‐phase TMNs, such as CrN, TiN, and NbN, which form pseudo‐2D NFs composed of interconnected nanocrystals. Additionally, cubic‐phase TMNs like VN can be synthesized via ammonolysis, with the resulting crystal structures dependent on the thermal conditions during the reaction (Figure 6f).^[^ ^113^ ^]^ Recently, the production of 2D TMNs, such as TiN NSs, has been explored for applications as protective coatings on Zn electrodes in rechargeable batteries. These coatings significantly enhance the cyclic stability of the devices.^[^ ^114^ ^]^ In summary, HEX and cubic morphologies dominate the crystal structures of TMNs, with HEX being the more prevalent form.^[^ ^91^ ^]^ The structural flexibility of TMNs, combined with advanced synthesis techniques, highlights their potential in various energy and electronic applications.

#### 1.5.9 Morphological Characteristics

The morphology of TMNs is defined by physical characteristics including particle size, shape, porosity, and surface roughness. TMNs typically retain the morphological features of their precursor materials. For example, Bi's group^[^ ^115^ ^]^ synthesized VN mesocrystal NSs that preserved the structure of their Na_2_V_6_O_16_ precursor. Structural analysis revealed that the stability of these NSs was influenced by their crystallographic orientation, with the (111) plane exhibiting greater stability due to its lower surface energy (Figure 6g).^[^ ^116^ ^]^ Specific surface area (SSA) plays a critical role in TMNs performance in applications like photocatalysis and energy storage. Wang and colleagues,^[^ ^117^ ^]^ reported that MoN NSs exhibited an SSA of 118.98 m^2^ g^−1^ with mesopores around 4 nm in diameter, promoting efficient ion diffusion and photon interaction. Xiao's team^[^ ^118^ ^]^ found that CrN nanocrystals possessed the largest SSA among CrN, TiN, and NbN, which facilitated chemical adsorption. Additionally, Jiao and colleagues^[^ ^119^ ^]^ synthesized TiN NSs with a pleated, porous structure and an SSA of 123 m^2^ g^−1^. Hou's group^[^ ^91^ ^]^ observed that increasing annealing temperatures enhanced the SSA and affected the formation of micropores, with pore volume (P_*v*_) being highly sensitive to synthesis conditions. Overall, the morphology of TMNs, including SSA, pore structure, and the retention of precursor shape, is intricately linked to synthesis methods and significantly influences their functional properties in applications such as energy storage, catalysis, and adsorption.^[^ ^120^ ^]^ Moreover, while all material characteristics contribute to overall functionality, the electronic, optical, and plasmonic characteristics of TMNs present significant opportunities for innovation in energy conversion, environmental technologies, and next‐generation electronic devices.

### 1.6 Dimensions of TMNs

The electrochemical behavior and structural traits of TMNs are considerably affected by their dimensionality. 0D TMNs, while providing extensive reactive SSA, frequently face challenges related to agglomeration. In contrast, 1D NWs demonstrate improved electron transport and mechanical strength, making them exceptionally suitable for applications in energy conversion devices. 2D TMNs, which possess planar structures, enable efficient electron mobility and enhanced charge‐storage capabilities, although their synthesis remains a complex task. 3D TMNs, on the other hand, exhibit optimal ion transport and charge transfer characteristics, particularly in thick electrode materials, thereby proving essential for next‐generation batteries and energy storage systems. This section provides an in‐depth discussion of these dimensional characteristics.

#### 1.6.1 0D

TMNs typically necessitate high temperatures for the successful incorporation of N into metallic frameworks or for the substitution of O‐atoms in oxide precursors. This process is fundamentally constrained by kinetic barriers. During nitridation, volumetric changes often occur, leading to particle fragmentation and agglomeration. For instance, the transformation of NiFe(OH)_2_ nanostructures into Ni_3_FeN NPs showcases a significant increase in both particle size and depth. Interestingly, smaller NPs are linked to a larger SSA and enhanced electrochemical properties.^[^ ^121^ ^]^ To address aggregation issues and promote uniformity, advanced synthesis techniques are essential, especially for the production of 0D TMNs. One effective method is the urea‐glass technique, which allows for the generation of fine ZrN NPs at around 800 °C, effectively mitigating agglomeration while enhancing surface activity. Furthermore, incorporating C‐based materials such as rGO and CNTs has been shown to be beneficial, as they improve dispersion, electrical conductivity, and mass transport, and also enable tunable electronic characteristics through interactions with TMNs.^[^ ^122^ ^]^

**Figure 7:**
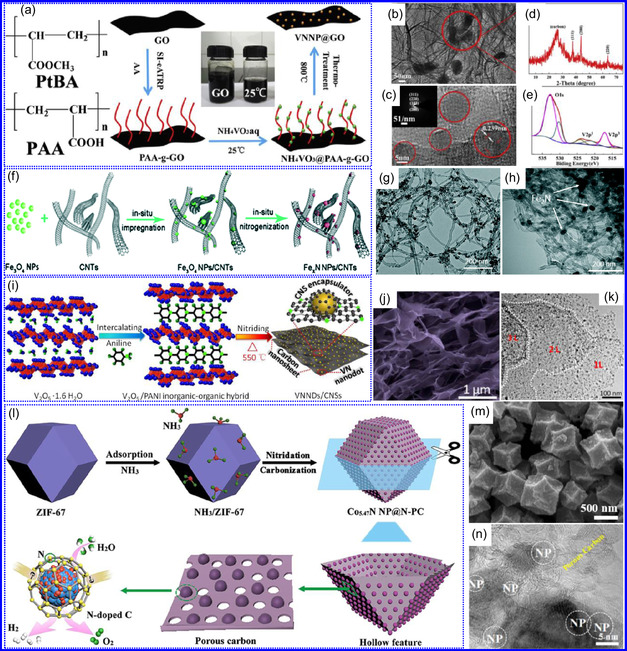
Preparation strategies for 0D TMNs: a–d) Preparation approach, TEM, XRD, and e) XPS analysis of VNNP@GO. Adapted with permission.^[^ ^123^ ^]^ Copyright 2019, Elsevier B.V. f–h) Preparation approach, and TEM analysis of Fe_2_N NP/CNT. Adapted with permission.^[^ ^124^ ^]^ Copyright 2018, Royal Society of Chemistry. i–k) Preparation approach, SEM, and TEM analysis of VNNDs/CNSs. Adapted with permission.^[^ ^125^ ^]^ Copyright 2018, Elsevier B.V. l–n) Preparation approach, SEM, and TEM analysis of Co_5.47_N NP@N‐PC. Adapted with permission.^[^ ^126^ ^]^ Copyright 2018, American Chemical Society.

He and colleagues^[^ ^123^ ^]^ utilized an electrochemical surface‐induced atomic transfer radical polymerization technique to synthesize 0D VN NPs. These NPs exhibited uniform dispersion on GO, leading to the formation of a layered composite. The cooperative interaction between the electrochemically active VN and the conductive GO substrate resulted in enhanced performance, ensuring sustained electrode functionality across repeated charge‐discharge cycles. The fabrication of the VNNP@GO composite occurred in multiple steps: First, poly(acrylic acid) (PAA) rings were covalently attached to the GO surface. Next, the ammonium metavanadate (AMV) was integrated into the PAA structures, followed by thermal processing (**Figure** **7**a). Polymerization commenced with the anchoring of poly(tert‐butyl acrylate) (‐PtBA) chains to GO, which were then hydrolyzed to form ‐PAA. AMV molecules were attracted to the GO‐PAA complex through electrostatic forces. The final thermal treatment under a N_2_ and NH_3_ atmosphere facilitated the formation of the VNNP@GO electrode. Characterization techniques such as TEM, XRD, and XPS confirmed the uniform dispersion of VN NPs and their polycrystalline structure. TEM analysis revealed distinct diffraction patterns corresponding to the VN crystal planes, validating the VN‐C composite architecture (Figure 7b–e). Electrochemical assessment in a 2 M KOH solution indicated a specific capacitance (C_sp_) of 109.7 F g^−1^ with a 93% retention of capacitance after 5000 cycles. While the ambient temperature during AMV introduction had minimal influence, the dimensions of the polymer rings were critical in determining VN doping levels and electrochemical properties.

Hui and colleagues^[^ ^124^ ^]^ conducted a study on enhancing the catalytic performance of 0D TMNs, specifically Fe_2_N, supported on CNTs for H_2_ production (**NH**_**3**_ **→ H**_**2**_ **+ N**_**2**_) via NH_3_ decomposition at lower temperatures (Figure 7f). The investigation revealed that both the nitrogenization temperature and the concentration of Fe significantly influenced the size and distribution of Fe_2_N crystallites. The improved catalytic activity was attributed to the formation of stable, uniformly dispersed Fe_2_N species, which were facilitated by increased nitrogenization temperatures and higher Fe content during NH_3_ flow. TEM analysis indicated that Fe_3_O_4_ concentrations of up to 10 wt% on CNTs resulted in nanocrystals that were homogeneously distributed with consistent dimensions. After nitrogenization at 500 °C, Fe particles were found to be well‐dispersed on CNTs; however, at a 15 wt% loading of Fe_3_O_4_, notable agglomeration occurred. Furthermore, the nitrogenization temperature impacted on the size of Fe_2_N NPs: Even distribution was maintained at 400 and 500 °C, while aggregation was significant at 600 °C. Importantly, the phase structure of Fe_2_N crystallites remained stable across all temperatures studied (Figure 7g,h). These findings highlight the importance of controlling the density of active sites in Fe_2_N and optimizing their interactions with CNTs to enhance catalytic efficiency in NH_3_ decomposition.

Qing's team^[^ ^125^ ^]^ developed a spatially confined preparation approach to create a novel 0D in 2D lamellar composite featuring VNNDs embedded within carbon NSs (CNSs) (Figure 7i). This engineered material exhibited an impressive volumetric capacitance of 1203.6 F cm^−3^ at a current density of 1.1 A cm^−3^, exceeding the performance of traditional C and TMOs/TMNs‐based SCs. Despite a considerable thickness of 150 μm, the composite maintained a capacitance of 867.1 F cm^−3^ and demonstrated excellent cycling stability, retaining 90% of its capacitance after 10 000 cycles. The flexible SCs constructed with the VNNDs/CNSs composite also showed remarkable electrochemical stability, achieving 91% capacitance retention after repeated cycling. SEM analysis revealed that the precursor V_2_O_5_.1.6H_2_O NTs had lateral dimensions ranging from 0.5 to 2 μm and thicknesses between 4 and 10 nm. The intercalation of aniline monomers within these NSs facilitated the formation of aniline‐intercalated V_2_O_5_ NTs. SEM images further confirmed that the resulting PANI/V_2_O_5_ NTs retained structural similarity to the original V_2_O_5_.1.6H_2_O NTs. The concentration of PANI was tunable by adjusting the molar ratio of aniline to V_2_O_5_. Subsequent thermal nitridation of the PANI/V_2_O_5_ NTs resulted in the final VNNDs/CNSs composite while preserving the nanotubular morphology (Figure 7j). TEM revealed lattice fringes with a spacing of 0.24 nm, corresponding to the (111) planes of cubic VN, confirming the successful embedding of crystalline VNNDs within amorphous CNSs. The intercalation of VNNDs contributes to a plethora of electroactive sites and provides protection against electrochemical oxidation, thereby enhancing capacitance retention and long‐term durability (Figure 7k). This preparation method presents promising strategies for designing high‐performance, flexible, and energy‐dense electrode materials.

Chen and colleagues^[^ ^126^ ^]^ synthesized an innovative nanocomposite, Co_5.47_N NPs encapsulated within N‐doped porous C (N–PC) polyhedra, designated as Co_5.47_N NP@N‐PC. This material demonstrates notable bifunctional electrocatalytic efficiency, achieving low overpotentials of 149 and 248 mV for HER and OER, respectively, at a current density of 10 mA cm^−2^. An electrolyzer employing Co_5.47_N NP@N‐PC on both electrodes in an alkaline environment exhibited superior performance compared to the Pt/IrO_2_ benchmark, providing the same current density at a reduced cell voltage of 1.62 V (Figure 7l). Structural characterization via SEM and TEM revealed that the ZIF‐67 precursors initially exhibited smooth polyhedral morphologies ranging from 500 to 700 nm. Following nitridation in NH_3_ at 700 °C, the materials retained their dodecahedral shape while developing a rough and porous exterior (Figure 7m). TEM and HR‐TEM imaging confirmed the presence of Co_5.47_N nanocrystals embedded within a graphitic C (GC) matrix, characterized by a d‐spacing of 0.208 nm corresponding to the (111) crystal plane of Co_5.47_N, surrounded by three to five GC layers (Figure 7n). Enhanced catalytic activity is attributed to the synergistic effects of N vacancies in Co_5.47_N, improved conductivity, N‐doping in C, and the porous morphology. In conclusion, 0D TMNs exhibit significant potential across various technological sectors due to their elevated surface reactivity and electrochemical properties. Although high‐temperature synthesis presents challenges such as particle aggregation, innovations like the urea‐glass method and C‐based supports (e.g., GO an CNTs) have facilitated the production of stable, well‐dispersed TMNs, leading to increases in SSA and enhanced electrochemical performance, as evidenced by the remarkable activity of VN NPs and CoN‐based composites.

Quantum dots (QDs), characterized as nanoscale 0D semiconducting structures, significantly enhance the performance of TMNs through their distinctive photonic and electronic characteristics, which arise from quantum confinement effects. The combination of QDs with TMNs leads to improved light absorption and catalytic efficiency, owing to their tunable optical BGs and high SSAs.^[^ ^127^ ^]^ This interaction allows for precise engineering of BGs, enhancing the electronic characteristics essential for electrocatalysis and energy storage, while also promoting effective charge carrier dynamics by reducing recombination events. Additionally, QDs provide structural stability to TMNs, protecting them from environmental degradation. Consequently, this integration expands the functional applications of TMNs, facilitating advancements in areas such as electronics, bioimaging, and quantum computing technologies.^[^ ^128^ ^]^

**Figure 8:**
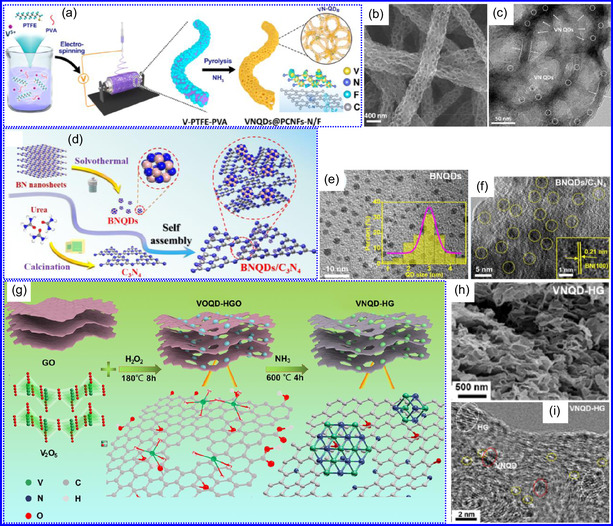
a,b) Preparation strategy, SEM, and c) TEM images of VNQDs@PCNFs‐N/F. Adapted with permission.^[^ ^129^ ^]^ Copyright 2022, American Chemical Society. d–f) Preparation strategy, TEM, and HR‐TEM images of BNQDs/g‐C_3_N_4_. Adapted with permission.^[^ ^130^ ^]^ Copyright 2022, Elsevier B.V. g–i) Preparation strategy, SEM, and HR‐TEM images of VNQD‐HG. Adapted with permission.^[^ ^131^ ^]^ Copyright 2021, American Chemical Society.

Yuan's group^[^ ^129^ ^]^ introduced a sophisticated SCs architecture featuring VNQDs embedded within N and F co‐doped 1D porous C nanocages (PCNFs‐N/F) as the anode, complemented by an asymmetric cathode made from APCNFs‐N/F (**Figure** **8**a). This configuration enhances electrochemical kinetics and structural stability by promoting efficient electron transport and alleviating volume expansion issues during redox cycling. The co‐doped C framework, notable for its high pore density and defect sites, facilitates rapid ion diffusion, thereby supporting pseudocapacitive energy storage. The device demonstrated an impressive energy density of 157.1 Wh kg^−1^ at a power density of 198.8 W kg^−1^, while maintaining 95 Wh kg^−1^ at a much higher power density of 9100.5 W kg^−1^. Morphological assessments using FE‐SEM and TEM indicated that the VNQDs@PCNFs‐N/F composite forms a well‐connected 1D nanocage network (Figure 8b), with VNQDs sized between 2 and 5 nm evenly distributed throughout the nanocages. This distribution effectively reduces particle agglomeration and volume changes during cycling, thereby promoting enhanced ion mobility. TEM analysis revealed lattice fringes of 0.206 nm, corresponding to the (200) plane of VN. This research presents a promising method for optimizing the electrochemical balance between electrode components in asymmetric SCs (Figure 8c).

Li and collaborators^[^ ^130^ ^]^ created a metal‐free electrocatalyst by integrating boron nitride QDs (BNQDs) with g‐C_3_N_4_, which significantly enhanced N_2_RR performance. The BNQDs/g‐C_3_N_4_ composite achieved an NH_3_ production rate of 72.3 μg h^−1^ mg^−1^ at −0.3 V, demonstrating a Faradaic efficiency (FE) of 19.5% at −0.2 V, outperforming pure BNQDs, g‐C_3_N_4_, and other nonmetallic catalysts (Figure 8d). DFT simulations indicated that the hybrid structure facilitates N_2_ activation while mitigating HER interference. XRD patterns displayed the characteristic (100) and (002) planes of g‐C_3_N_4_, with no distinct peaks for BNQDs due to their low diffraction intensity. Raman spectroscopy revealed a prominent peak at ≈1370 cm^−1^, associated with the E_2g_ vibrational mode of B–N bonding. XPS confirmed the presence of B, C, and N, highlighting B 1s and N 1s signals indicative of B–N and N–B bonds, along with significant electronic interactions between the BNQDs and g‐C_3_N_4_. The catalyst was synthesized using a self‐assembly method, employing BNQDs derived from BNNSs through solvothermal processing and g‐C_3_N_4_ produced via urea pyrolysis. TEM imaging demonstrated a uniform dispersion of BNQDs (≈3.2 nm) over g‐C_3_N_4_ NSs, exhibiting a wrinkled morphology (Figure 8e). HR‐TEM confirmed the ordered anchoring of BNQDs to the g‐C_3_N_4_ matrix with observable lattice fringes corresponding to the (100) plane of HEX BNs. Elemental mapping verified a homogeneous distribution of B, C, and N across the composite (Figure 8f). This study presents a promising approach to designing high‐efficiency nonmetal heterostructured catalysts for electrochemical NH_3_ synthesis.

In a separate study, Li and colleagues^[^ ^131^ ^]^ developed VNQDs integrated into a holey graphene structure (VNQD‐HG) to stabilize sulfur (S) cathodes in LSBs. This VNQD‐HG composite effectively captures, anchors, and catalyzes the conversion of Li polysulfides (LiPSs) and Li sulfide (Li_2_S), thereby improving battery performance (Figure 8g). The structure boasts a high capacity for LiPSs adsorption, strong binding to Li_2_S, and rapid redox kinetics. Its porous network, both in‐plane and out‐of‐plane, not only aids S storage but also speeds up the transport of Li ions and electrons. Consequently, the modified S cathode achieved an impressive initial capacity of 1320 mAh g^−1^, quick charging capabilities, and an outstanding 99.9% capacity retention over 500 cycles. The LSBs also demonstrated excellent cycling and rate performance, indicating their potential as long‐lasting, shuttle‐free energy storage systems. SEM images revealed a 3D porous structure in VNQD‐HG, which facilitates efficient S infiltration and ionic/electronic transport (Figure 8h), while TEM showed evenly distributed VNQDs as black dots on crumpled graphene NSs. HR‐TEM further illustrated in‐plane nanopores and increased interlayer spacing (0.386 nm) due to N/O doping, which is beneficial for LiPSs confinement and ion diffusion (Figure 8i). The VNQDs were uniformly sized at 2–3 nm, with lattice fringes of 0.207 nm corresponding to the (200) VN plane. Elemental mapping confirmed the even distribution of C, V, and N, highlighting the catalytic synergy between VNQDs and the graphene matrix. This research provides a valuable framework for creating high‐performance, durable LSBs. In summary, QDs significantly enhance the performance of TMNs through quantum confinement, leading to improved light absorption, stability, and catalytic activity across various fields, including SCs, N_2_RR, and battery technologies.

Nanospheres (NS) are essential in the creation, design, and enhancement of TMNs for various uses, especially in catalysis and energy storage. Acting as structural templates, NS influence the size, shape, and microstructural features of TMNs, resulting in uniform 0D nanostructures with beneficial traits like increased porosity and higher SSA. Their high surface‐to‐volume ratio and small size boost the number of active catalytic sites, enhancing electrocatalytic performance.^[^ ^132^ ^]^ Furthermore, NS improves electron mobility and lower charge‐transfer resistance, which are vital for efficient energy storage devices. They also help maintain structural integrity and durability by accommodating volume changes during cycling. Additionally, the NS structure ensures optimal exposure of catalytically advantageous crystal facets, further increasing catalytic effectiveness. The ability of NS to enable controlled porosity significantly enhances mass transport and diffusion, thereby improving the overall performance of TMN‐based systems.^[^ ^132^ ^]^

**Figure 9:**
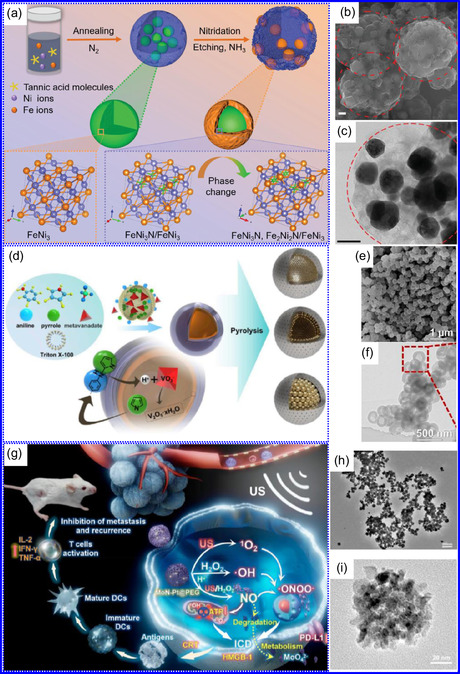
a–c) Preparation strategy, SEM, and TEM images of FeNi_3_‐N. Adapted with permission.^[^ ^133^ ^]^ Copyright 2021, Wiley‐VCH. d–f) Preparation strategy, SEM, and TEM images of HHS‐VO_*x*_@PACP‐0.01 M. Adapted with permission.^[^ ^134^ ^]^ Copyright 2020, Elsevier B.V. g–i) Preparation strategy, TEM, and HR‐TEM images of MoN‐Pt@PEG. Adapted with permission.^[^ ^135^ ^]^ Copyright 2023, American Chemical Society.

Chen's team^[^ ^133^ ^]^ developed a surface phase engineering method to improve the OER performance of TMNs. They created FeNi_3_‐based nitrides and alloy NPs (FeNi_3_‐N) that were encapsulated in a C matrix. This method allowed for the formation of dual‐phase nitrides on the surface of the NPs through atomic diffusion during the nitridation process. The optimized FeNi_3_‐N electrocatalyst showed excellent OER activity, characterized by a low overpotential, a small Tafel slope, and strong operational stability at high current densities. The FeNi_3_‐N catalysts were produced by adjusting the nitridation conditions, specifically the temperature and duration in an NH_3_ atmosphere, resulting in different variants labeled as FeNi_3_‐N‐X‐Y (where *X* and *Y* represent temperature and time, respectively) (**Figure** **9**a). Structural analysis using SEM and TEM indicated that higher nitridation intensities led to rougher surfaces, particularly in the FeNi_3_‐N‐350‐8 sample (Figure 9b,c), which exhibited features like wrinkles and perforations that increased the SSA. The catalysts were composed of ≈700 nm clusters of uniformly distributed 90 nm NPs embedded in C, which helped prevent agglomeration and enhanced the availability of active sites. DFT calculations further indicated that the dual‐phase nitride structure lowers energy barriers and optimizes the d‐band center, significantly improving OER efficiency.

Xu and his team^[^ ^134^ ^]^ created a one‐pot, domino‐driven method for synthesizing hollow C NS embedded with ultrafine TMNs. The synthesis involves a series of steps: first, micelle‐surface copolymerization forms a polymer shell; next, copolymerization‐induced oxometallate precipitation occurs within the micelles; and finally, controlled pyrolysis is performed. The initial aqueous mixture includes a surfactant (Triton X‐100), comonomers (aniline and pyrrole), and a metal precursor (such as AMV), which together kickstart the hierarchical synthesis (Figure 9d). During copolymerization, the generation of protons leads to the condensation of AMV into hydrated V_2_O_5_ in the micelle core, while the shell forms on the micelle's surface. After drying, this process results in hollow VO_*x*_@PACP spheres (HHS‐VO_*x*_@PACP‐xx), where “xx” refers to the precursor concentration. The subsequent pyrolysis in an NH_3_ environment converts VO_*x*_ into VN, producing HHS‐VN@C‐xx‐yy, with “yy” indicating the pyrolysis temperature. Structural analysis using TEM and HAADF‐STEM reveals uniform hollow NS (≈248 nm outer and ≈130 nm inner diameter) with amorphous V evenly distributed along the cavity walls (Figure 9e,f). The absence of crystalline V phases is confirmed by XRD analysis. Importantly, this synthesis method is adjustable, allowing for the controlled production of nanoscale nitrides with improved electrochemical properties. The hollow structure enhances ion diffusion and contributes to better K^+^ storage performance.

Bai's group^[^ ^135^ ^]^ developed MoN@PEG, a TMN nanostructure designed for the targeted release of nitric oxide (NO) in cancer treatment. This material takes advantage of the high electronegativity of N and the weakened Mo–N bond, allowing for H^+^‐induced denitrogenation and NO release in conditions typical of the tumor microenvironment (TME) (Figure 9g). Additionally, reactive oxygen species (ROS) help break down NH_*x*_ intermediates, increasing the NO output to 94.1 ± 5.6 μM without the need for additional carriers. MoN@PEG has inherent catalytic properties, functioning as both a peroxidase mimic and a sonosensitizer. A composite version, MoN‐Pt@PEG, was created to enhance sonodynamic therapy (SDT) by improving charge separation and ROS production. When subjected to ultrasound, this results in the in‐situ generation of the cytotoxic compound peroxynitrite (.ONOO^‐^). The particles gradually break down over 14 days, showing a high level of biodegradability (94.6% clearance through excretion). MoN is produced from MoO_2_ NPs through a simple NH_3_‐driven nitridation method. TEM analysis shows that the porous nanostructures have a diameter of ≈80 nm and consist of grains smaller than 10 nm (Figure 9h,i), with a high SSA (65 m^2^ g^−1^) and P_*v*_ (0.2 cm^3^ g^−1^), as verified by N_2_ adsorption isotherms and H_3_‐type hysteresis. XRD, HR‐TEM, and SAED confirm crystallinity on the (202) and (200) planes, while XPS indicates Mo–N bonding, surface oxidation, and different valence states of Mo. This research presents a new platform for NO delivery, advancing TMN‐based immunotherapy and sonodynamic cancer treatments.

#### 1.6.2 1D

1D TMNs have attracted significant interest for their application in next‐generation technologies due to their remarkable physical and chemical characteristics.^[^ ^136^ ^]^ Their 1D structure, typically found as NWs or NTs, creates continuous pathways for electron movement, thereby improving the electrical performance of devices such as batteries, SCs, and electrocatalysts.^[^ ^137^ ^]^ These structures also possess a high surface‐to‐volume ratio, which provides numerous active sites for chemical reactions. Furthermore, 1D TMNs exhibit excellent mechanical strength and stability, making them ideal for use in flexible electronics and environments that are challenging.^[^ ^138^ ^]^ Their design also promotes ion diffusion and enhances the performance of composites through synergistic effects.^[^ ^139^ ^]^

**Figure 10:**
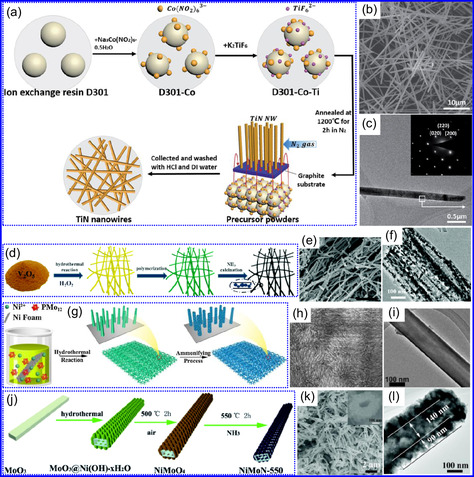
Preparation strategies for 1D TMNs: a–c) Preparation strategy, SEM, and TEM images of TiN NWs. Adapted with permission.^[^ ^140^ ^]^ Copyright 2016, Royal Society of Chemistry. d–f) Preparation strategy, SEM, and TEM images of VN@C NWs. Adapted with permission.^[^ ^141^ ^]^ Copyright 2018, Royal Society of Chemistry. g–i) Preparation strategy, SEM, and TEM images of 1D P‐NiMo_4_N_5_@Ni. Adapted with permission.^[^ ^142^ ^]^ Copyright 2019, Elsevier B.V. j–l) Preparation strategy, SEM, and TEM images of 1D Ni‐Mo nitride NTs. Adapted with permission.^[^ ^143^ ^]^ Copyright 2017, Royal Society of Chemistry.

Han and colleagues^[^ ^140^ ^]^ successfully created a novel CVD method to produce 1D TiN NWs, proving their effectiveness as catalysts for HER. The synthesis involved combining anion‐exchange resin powders with Co(NO_2_)_6_^3‐^ and TiF_6_^2‐^ precursors, followed by annealing under a graphite bar at 1200 °C in a N_2_ atmosphere for 2 h. This resulted in a golden‐brown coating of TiN NWs on the substrate. After synthesis, the NWs were purified using HCl acid and DI water (**Figure** **10**a). SEM analysis revealed well‐defined 1D structures, with NWs averaging lengths of up to 100 μm and widths of ≈330 nm, which are different from bulk TiN NPs (Figure 10b). The resulting network structure provides a high SSA, improved charge mobility, and excellent conductivity. TEM and HR‐TEM analyses confirmed the single‐crystalline nature of the NWs and their uniform elemental distribution, with a lattice spacing of 0.21 nm (Figure 10c). Electrochemical tests showed remarkable HER performance, with an overpotential of 92 mV at 1 mA cm^−2^ and a Tafel slope of 54 mV dec^−1^. Importantly, the NWs retained their performance after 20 000 cycles and 100 h in acidic conditions, demonstrating their durability and catalytic capabilities.

Zhu's team^[^ ^141^ ^]^ created a 1D VN@N‐C NWs (VN@C) composite for LSBs. By utilizing the polar characteristics of VN and the conductive qualities of C, this composite improves S redox reactions, reduces the dissolution of LiPSs, and decreases self‐discharge. The synthesis starts with V_2_O_5_ NWs, which act as both a precursor and a structural template (Figure 10d). These NWs are coated with polypyrrole (PPy), resulting in a core‐shell architecture (V_2_O_5_@PPy), which is then thermally treated in an NH_3_ atmosphere to produce the VN@C composite. SEM analysis of the V_2_O_5_ NWs revealed smooth surfaces, with diameters between 30 and 90 nm and lengths reaching several μms (Figure 10e). After the PPy coating, the NWs maintained their 1D structure, though with a slight increase in size. The final VN@C composite retained its 1D shape, featuring an outer C layer thickness of 10–20 nm (Figure 10f). Electrochemical tests showed impressive results, including a discharge capacity of 875 mAh g^−1^ at 1 C, a rate capability of 550 mAh g^−1^ at 6 C, and strong performance even with a high S loading of 4.2 mg cm^−2^. These results indicate significant potential for advancing high‐performance LSBs.

Shen and colleagues^[^ ^142^ ^]^ developed hierarchical 1D P‐NiMo_4_N_5_@Ni catalysts with a controlled structure using a two‐step method. They incorporated polyoxometalates (PMo_12_) during the synthesis to effectively guide nucleation and crystal growth, resulting in unique nanostructures on Ni foam (NF). The first step involved a hydrothermal treatment with PMo_12_, Ni(NO_3_)_2_, urea, and NF at 180 °C for 12 h, which produced disordered NRAs made of Ni–Mo oxide complexes (Figure 10g). PMo_12_ was crucial in concentrating Ni^2+^ ions at the substrate interface, promoting the vertical alignment of the NRs on the NF. In the second step, the precursor was annealed in an NH_3_ atmosphere at 700 °C for 4 h, resulting in twisted NiMo_4_N_5_ NRs on NF, referred to as P‐NiMo_4_N_5_@Ni‐1. SEM analysis revealed that the precursor consisted of NiMoO_4_.xH_2_O and Ni_2_O_3_H, organized into uniform rod‐like clusters with smooth surfaces (Figure 10h). TEM imaging showed a consistent distribution of elements, and post‐annealing analysis indicated that the twisted NR structure was due to the replacement of O atoms with N. Changes in the amount of Ni precursor led to various morphologies, such as nanoflower and tremella‐like NSs arrays (Figure 10i). While the elemental distribution remained uniform across samples, morphological irregularities emerged when urea was omitted or sodium molybdate was used instead of PMo_12_. The final P‐NiMo_4_N_5_@Ni‐1 catalyst demonstrated a high SSA and excellent bifunctional electrocatalytic performance for overall water splitting (OWS), achieving 50 and 100 mA cm^−2^ at low cell voltages of 1.59 and 1.66 V, respectively. This research lays a strong foundation for the strategic design of nitride‐based electrode materials for advanced electrochemical applications.

Yin's group^[^ ^143^ ^]^ developed a simple, multi‐step method to synthesize 1D Ni–Mo nitride NTs, which act as efficient and stable bifunctional electrocatalysts for OER and HER. These NTs demonstrated low overpotentials of 295 mV for the OER and 89 mV for the HER at a current density of 10 mA cm^−2^. When used in a complete alkaline water electrolyzer, the system maintained the same current density at a cell voltage of about 1.59 V. The synthesis involved the hydrothermal creation of MoO_3_ NRs, which were then coated with Ni(OH)_2_.xH_2_O using a solvothermal method to form core‐shell structures. These intermediates were annealed in air to produce NiMoO_4_ NTs, which were later transformed into Ni–Mo nitride NTs by heating in an NH_3_ atmosphere at 550 °C for 2 h. The final product, NiMoN‐550 NTs, included crystalline Ni_0.2_Mo_0.8_N, metallic Ni, and a small amount of Ni_3_N (Figure 10j). SEM images showed uniform tubular structures with diameters around 330 nm and lengths on the μm scale (Figure 10k), while TEM confirmed distinct lattice fringes corresponding to Ni_0.2_Mo_0.8_N and metallic Ni (Figure 10l). These NTs demonstrated superior stability compared to traditional Pt and IrO_2_ catalysts at high current densities. Mechanistic investigations revealed that NiOOH and NH species were active for OER, while Ni(OH)_2_, NH, and Mo species were essential for HER. This approach highlights a promising pathway for creating cost‐effective, high‐performance electrocatalysts for large‐scale OWS. In summary, 1D TMNs are notable for their improved electron mobility, high surface‐area‐to‐volume ratios, and mechanical strength, making them ideal for applications in energy storage, catalysis, and flexible electronics.

NRs in TMNs have distinct structural and functional characteristics that greatly improve their effectiveness in various fields, such as catalysis, energy storage, and biomedical uses. Their high surface‐to‐volume ratio provides many active sites, making them very efficient for catalytic processes. Furthermore, their anisotropic shape aids in directional electron transport, which is essential for high‐performance devices like SCs and batteries.^[^ ^73^ ^]^ The presence of reactive crystalline surfaces on NRs enhances their catalytic activity, especially in important reactions like the HER and OER. NRs also demonstrate greater mechanical stability than other nanostructures, making them suitable for prolonged use in challenging environments.^[^ ^144^ ^]^ Their synthesis allows for precise morphological control, enabling optimization for specific applications, such as improving light absorption in photocatalysis.^[^ ^144^ ^]^ Additionally, their elongated shape facilitates effective mass transport in electrochemical systems, and their incorporation into composite materials boosts mechanical strength, conductivity, and overall catalytic activity. These attributes make NRs in TMNs highly adaptable, durable, and reactive, making them ideal components for advancing technologies in energy, sensing, and environmental cleanup.^[^ ^145^ ^]^

**Figure 11:**
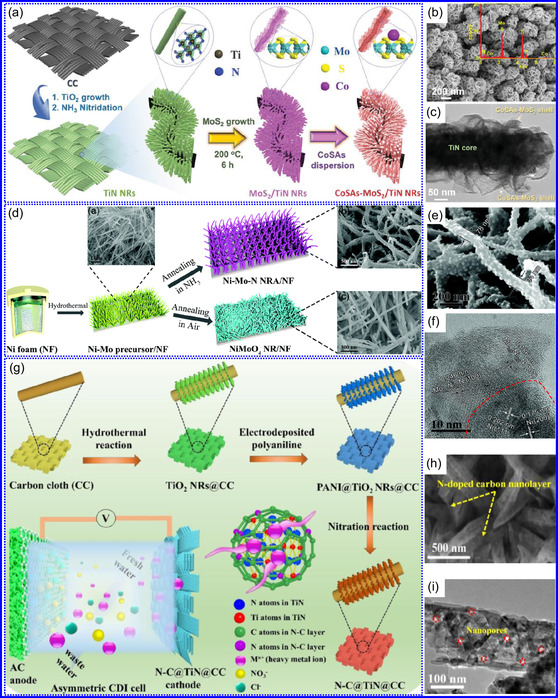
a–c) Preparation strategy, SEM, and TEM images of CoSAs‐MoS_2_/TiN composites. Adapted with permission.^[^ ^146^ ^]^ Copyright 2021, Wiley‐VCH. d–f) Preparation strategy, FE‐SEM, and TEM images of Ni NPs‐decorated Ni_0.2_Mo_0.8_N NRs matrix. Adapted with permission.^[^ ^147^ ^]^ Copyright 2017, Royal Society of Chemistry. g–i) Preparation strategy, SEM, and TEM images of 3D self‐supported N‐C@TiN@CC composites. Adapted with permission.^[^ ^148^ ^]^ Copyright 2022, Elsevier B.V.

Lee and colleagues^[^ ^146^ ^]^ created a high‐performance bifunctional electrocatalyst by combining Co SACs (CoSAs) with MoS_2_ NSs supported on 3D TiN NRs, resulting in a hierarchical CoSAs‐MoS_2_/TiN NRs structure. This composite exhibited outstanding HER performance across various pH levels, achieving overpotentials of only 187.5 mV in acidic conditions, 131.9 mV in alkaline conditions, and 203.4 mV in neutral conditions to reach a current density of 10 mA cm^−2^ when used as a self‐supporting cathode. The fabrication process started with the growth of TiO_2_ NRs (150 nm in diameter) on C cloth (CC), which were then transformed into TiN through high‐temperature annealing in NH_3_. MoS_2_ NSs were then deposited onto the TiN NRs using a hydrothermal method, followed by the immersion of Co precursors and subsequent annealing and acid etching to produce the final CoSAs‐MoS_2_/TiN NRs catalyst (**Figure** **11**a). SEM analysis revealed a stable core‐shell architecture (300 nm in diameter, 75 nm shell thickness) without any visible structural defects (Figure 11b). TEM imaging further validated the close interface between the CoSAs‐MoS_2_ NSs and TiN NRs (Figure 11c). The catalyst also showed significant OER activity, with overpotentials of 454.9 mV (acidic), 340.6 mV (alkaline), and 508 mV (neutral) at 10 mA cm^−2^. These results position CoSAs‐MoS_2_/TiN NRs as a strong and effective electrocatalyst for OWS in various pH conditions.

Jiang's group^[^ ^147^ ^]^ created Ni NP‐decorated Ni_0.2_Mo_0.8_N NRAs on NF through a simple nitridation using a NiMoO_4_ precursor, as illustrated in Figure 11d. The process involved soaking cleaned NF in a solution with Ni^2+^ and MoO_4_^−^ ions, which allowed for the in situ growth of Ni‐Mo precursor NRs uniformly on the NF surface. After nitridation at 500 °C for 4 h in an NH_3_ atmosphere, the porous structure was maintained, while the linear NRs transformed into tapered, cone‐like shapes adorned with Ni NPs. This hierarchical structure improved the SSA and reduced ion diffusion distances, enhancing electrochemical activity. In contrast, NiMoO_4_ NRs that were air‐annealed showed structural flaws, highlighting the benefits of nitridation for maintaining morphology. FE‐SEM revealed bent and tapered NRs with diameters of 70–90 nm and rough surfaces abundant in Ni NPs (Figure 11e). TEM confirmed that the rods were about 1.5 μm long and displayed lattice fringes corresponding to the (111) and (200) planes of crystalline Ni, as well as the (100) and (101) planes of Ni_0.2_Mo_0.8_N. Elemental mapping showed a uniform distribution of Mo and N, with Ni concentrated on the surfaces of the NPs (Figure 11f). The resulting material achieved a high areal capacity of 2446 mC cm^−2^ at 2 mA cm^−2^ and demonstrated excellent cycling stability. When utilized in HSCs alongside rGO, the system provided an energy density of 40.9 Wh kg^−1^ at 773 W kg^−1^ and maintained 80.1% of its capacitance after 6000 cycles, highlighting its potential for future energy storage technologies.

Sun's team^[^ ^148^ ^]^ developed an innovative method for creating self‐supporting composite electrodes for capacitive deionization (CDI) focused on heavy metal removal. The resulting electrode, named N‐C@TiN@CC, features N–C that encapsulates TiN NRs arranged in a flower‐like structure and anchored to a CC substrate (Figure 11g). This design combines a 3D, flower‐shaped open framework with a hierarchically porous architecture, which improves electrical connectivity, ion adsorption capacity, and mass transport efficiency. SEM and TEM images confirmed that the initially smooth CC surface was uniformly coated with dense rutile TiO_2_ NRs (≈714.4 nm in length and ≈196.1 nm in width) through hydrothermal treatment and thermal annealing in air. These NRs were then transformed into N‐C@TiN@CC by electrodepositing PANI and nitriding in NH_3_ (Figure 11h). HR‐TEM analysis confirmed the presence of the (111) crystalline plane of TiN and showed a uniform N‐C layer of 5.8 nm thickness surrounding the NRs, which enhances interfacial bonding and facilitates effective electron transport (Figure 11i). The electrode displayed a high density of nanopores, which increased the SSA and P_*v*_, both crucial for CDI performance. Furthermore, the N–C layer improved electrical conductivity, decreased interfacial charge resistance, and provided mechanical support to the TiN, reducing structural degradation during ion adsorption and desorption. The composite achieved a deionization efficiency of ≈98% in single, binary, and multi‐component simulated wastewater streams. Ex situ XRD and XPS analyses further clarified the ion removal mechanisms. This research offers a cost‐effective and scalable method for producing high‐performance TMN‐based CDI electrodes for heavy metal remediation. Overall, TMN NRs, due to their high aspect ratio, anisotropic conduction, and structural durability, show significant potential in energy storage, catalysis, sensing, and environmental remediation applications.

NWs within TMNs play a crucial role in improving material performance across various fields such as catalysis, energy storage, and electronics.^[^ ^149^ ^]^ Their high surface‐to‐volume ratio increases the number of reactive sites, enhancing catalytic activity and charge transfer efficiency.^[^ ^150^ ^]^ The 1D structure of NWs allows for effective electron transport, which lowers interfacial resistance and boosts electrical conductivity. Additionally, their porous and elongated design promotes efficient ion movement, reduces diffusion distances, and speeds up electrochemical reactions.^[^ ^73^ ^]^ NWs also enable the creation of composite architectures that combine different material properties for better performance. Their significant aspect ratio aids in thermal management, making them ideal for applications at elevated temperatures.^[^ ^151^ ^]^

**Figure 12:**
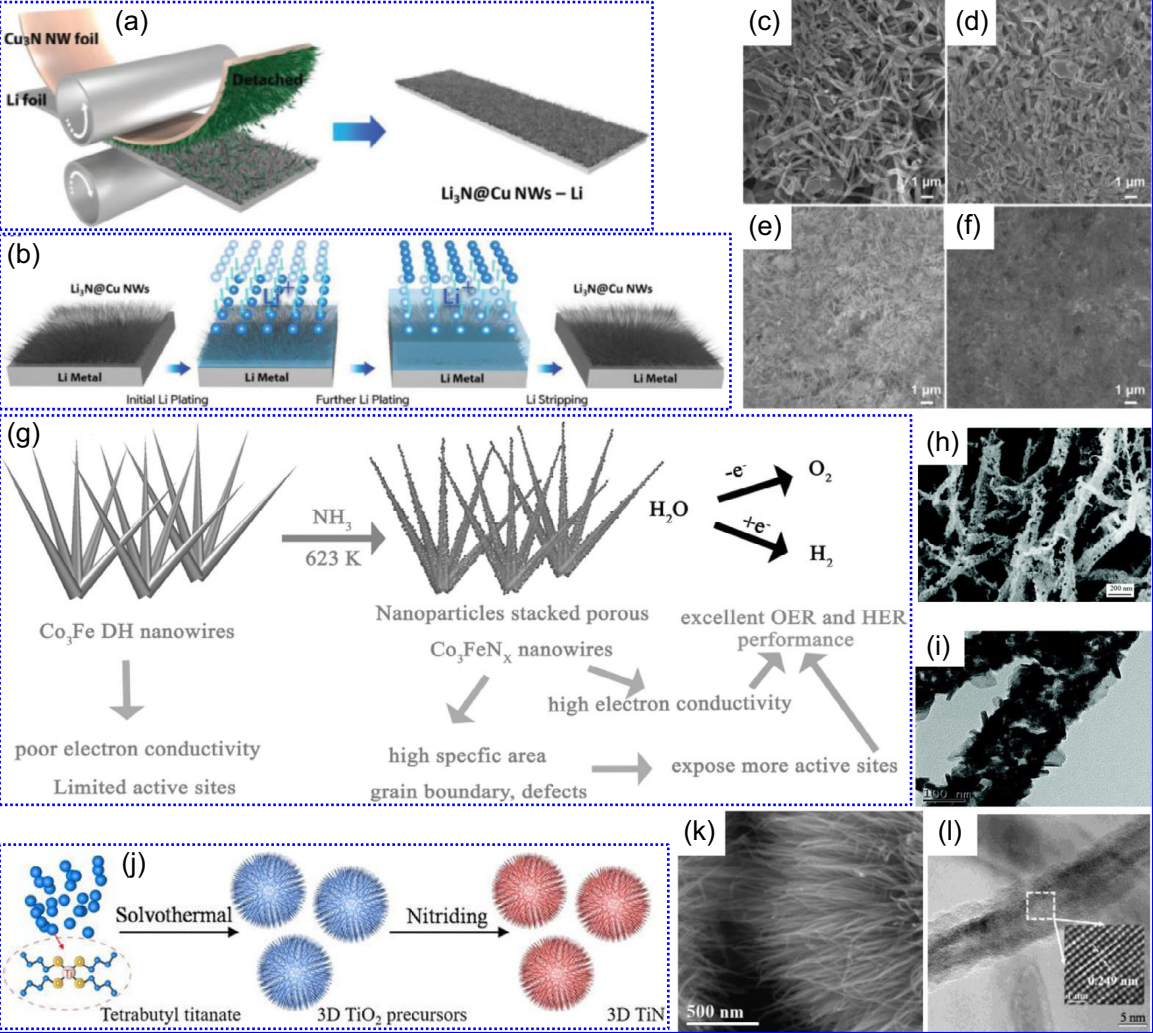
a–f) Preparation strategy, and SEM images of Li_3_N@Cu NWs‐Li electrode. Adapted with permission.^[^ ^152^ ^]^ Copyright 2020, Wiley‐VCH. g–i) Preparation strategy, SEM, and TEM images of NSP‐Co_3_FeN_*x*_ NWs. Adapted with permission.^[^ ^153^ ^]^ Copyright 2016, Royal Society of Chemistry. j–l) Preparation strategy, SEM, and TEM images of 3D porous mono‐/bi‐metallic nitrides. Adapted with permission.^[^ ^154^ ^]^ Copyright 2019, Wiley‐VCH.

Paik's team^[^ ^152^ ^]^ introduced an economical roll‐press technique for printing Cu_3_N NWs onto Li metal surfaces, making them suitable as high‐voltage cathodes in carbonate‐based electrolytes. This one‐step fabrication creates an insulating layer of Li_3_N@Cu NWs, which improves Li‐ion transport through a 3D network that takes advantage of Li_3_N's excellent conductivity. This engineered interface encourages even and flat Li deposition, effectively preventing dendritic growth. The modified Li electrode shows low overpotentials and remarkable cycling stability in symmetric cells, even at high current densities. In full‐cell setups with LiCoO_2_ cathodes, the system maintains stable performance for over 300 cycles, while pairing with Li_4_Ti_5_O_12_ isolates cathode effects, achieving consistent efficiency for more than 1000 cycles under practical conditions. **Figure** **12**a,b illustrates the NW synthesis, where pristine Cu foil is submerged in a solution of (NH_4_)_2_S_2_O_8_ and NaOH, resulting in Cu(OH)_2_ NWs after 10 min. Changes in color in the solution and foil indicate the transformation. These NWs, around 300 nm long and several microns wide, are converted to Cu_3_N through nitridation at 350 °C for 90 min in an Ar/NH_3_ atmosphere, maintaining the original crystalline structure. XRD analyses confirm the phase changes from Cu to Cu(OH)_2_ and then to Cu_3_N, which align with the observed color changes. Cross‐sectional SEM images show dendritic Li plating and surface porosity on untreated Li metal, leading to poor electrochemical performance due to uneven Li‐ion pathways, structural rigidity, and electrolyte degradation (Figure 12c–f). In contrast, Li deposition on Li_3_N@Cu NWs‐modified Li surfaces is uniform and dense, with dendrite suppression maintained after multiple Li stripping cycles. The 3D structure and high ionic conductivity of the Li_3_N@Cu NWs interface facilitate even Li‐ion distribution and lower local current densities, enhancing plating behavior. In comparison, bare Li surfaces show dendritic formations and capacity loss. The unique morphology and composition of the Li_3_N@Cu NWs coating significantly improve Li‐metal battery performance, akin to that seen in CuO NW‐printed electrodes.

Wang and colleagues^[^ ^153^ ^]^ created NPs‐stacked porous Co_3_FeN_*x*_ NWs that serve as highly effective electrocatalysts for water electrolysis. These catalysts were produced using simple nitridation. First, Co_3_Fe‐LDHs were grown on NF through a hydrothermal process. Then, annealing at 350 °C in an NH_3_ environment resulted in the formation of NPs‐stacked porous Co_3_FeN_*x*_ NWs on NF (Figure 12g). SEM was used to analyze the morphological and microstructural characteristics of the synthesized Co_3_FeN_*x*_ NWs (Figure 12h). XRD confirmed the 1D NW structure of the Co_3_Fe‐LDH precursor. After the NH_3_ treatment at 350 °C, the Co_3_FeN_*x*_ maintained its porous NW structure, with XRD patterns indicating the creation of TMNs like Co_2_N and Fe_3_N. TEM showed lattice fringes, grain boundaries, and structural defects within the NWs, which are thought to improve electrocatalytic activity. The ideal annealing temperature was determined to be 350 °C, which optimally balanced the retention of morphology and connectivity between NPs for effective catalytic activity (Figure 12i). This research presents a promising method for producing highly active bifunctional electrocatalysts that are effective for both HER and OER.

Tian's group^[^ ^154^ ^]^ developed a versatile and effective two‐step synthesis for producing 3D porous single‐ and bi‐metallic nitride structures with controlled morphology, size, and elemental composition. The resulting urchin‐like TiN nanostructures consist of many interconnected NWs, providing a high SSA and improved charge transport characteristics. The synthesis of 3D TiN involves two main steps (Figure 12j): First, 3D urchin‐shaped TiO_2_ nanostructures are formed through solvothermal treatment, followed by ammonolysis in a circulating NH_3_ environment to transform the oxide into TiN. The final product, about 2.7 μm in diameter, features a network of radially aligned porous NWs, which are well‐suited for mass transport in energy applications. TEM confirmed the high crystallinity of the TiN NWs, showing a 0.24 nm interplanar spacing typical of the FCC TiN (111) phase. The surfaces of the NWs, which have convex shapes and distinct atomic steps, suggest a high density of catalytic sites (Figure 12k). Bimetallic nitrides like Ti_0.9_Co_0.1_N and Ti_0.9_Cu_0.1_N exhibited improved electrocatalytic performance compared to their single‐metal counterparts, due to synergistic electronic effects. SEM analysis indicated that these binary structures maintained the 3D morphology of TiN (Figure 12l), and XRD confirmed the FCC TiN phase with slight peak shifts resulting from lattice contraction due to Cu/Co substitution. The synthesis produces high‐purity materials with accurate stoichiometry and no detectable impurities. SSA measurements showed high values of 126.1, 130.4, and 119.7 m^2^ g^−1^ for TiN, Ti_0.9_Co_0.1_N, and Ti_0.9_Cu_0.1_N, respectively, due to their unique 3D porous structure. These materials also demonstrated excellent electrochemical stability and maintained their structural and compositional integrity under operational conditions. Overall, TMNs NWs significantly enhance material efficiency in energy and catalytic systems, thanks to their high surface‐to‐volume ratios, superior electron mobility, and structural versatility.

TMN NTs have become essential nanostructures due to their distinctive structural, electrical, and chemical characteristics, which allow for their use in various advanced technologies.^[^ ^155^ ^]^ The high SSA of NTs improves their catalytic and energy storage capabilities by increasing the number of reactive sites. Their tubular shape also promotes fast electron transport, making them particularly beneficial for high‐performance energy storage devices like SCs and batteries.^[^ ^156^ ^]^ Their inherent stability ensures they maintain structural integrity under harsh conditions, while their geometry enhances the exposure of active SSAs, boosting electrocatalytic efficiency. Additionally, the adjustable nature of NTs permits strategic alterations through doping or functionalization, expanding their applications in catalysis, energy storage, and biomedical fields.^[^ ^157^ ^]^ Their compatibility with composite materials can lead to synergistic effects that significantly improve overall performance. Furthermore, defect engineering allows for the customization of electronic characteristics and the creation of novel active sites for specific reactions. Together, these features make TMN NTs highly promising candidates for the development of next‐generation functional materials.^[^ ^158^ ^]^

**Figure 13:**
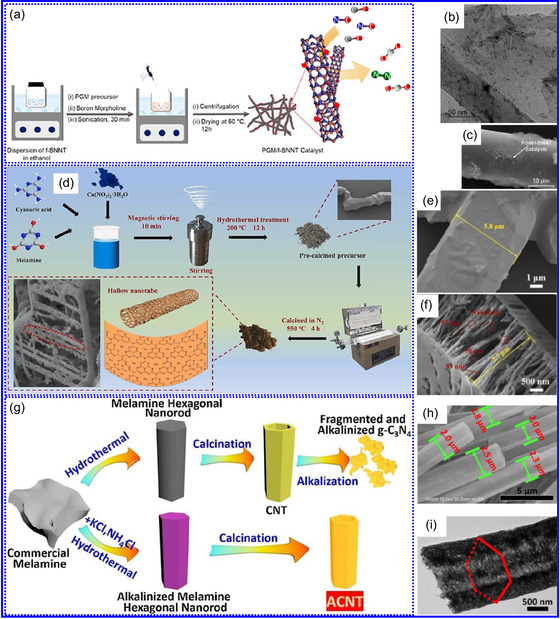
a–c) Preparation strategy, SEM, and TEM images of f‐BNNT. Adapted with permission.^[^ ^159^ ^]^ Copyright 2023, American Chemical Society. d–f) Preparation strategy, SEM, and TEM images of Cu‐HNCN. Adapted with permission.^[^ ^160^ ^]^ Copyright 2022, Elsevier B.V. g–i) Preparation strategy, SEM, and TEM images of ACNT. Adapted with permission.^[^ ^161^ ^]^ Copyright 2022, Elsevier B.V.

Kim's group^[^ ^159^ ^]^ introduced a new type of catalyst using precious group metal (PGM) NPs attached to functionalized BNNTs (f‐BNNTs), which showed outstanding catalytic activity for reducing NO with CO at room temperature (**Figure** **13**a). Even with a low PGM loading of 0.7–0.8 wt%, the catalysts achieved over 99% conversion of NO at temperatures as low as 200 °C. Their thermal stability was confirmed with Rh/f‐BNNTs, which maintained nearly complete NO conversion after being aged thermally at 600 and 700 °C in humid air. The high catalytic activity is attributed to the synergistic effects between the evenly distributed PGM NPs and the excellent surface characteristics of f‐BNNTs, providing a promising approach to minimize PGM usage. For industrial testing, the catalyst was applied to quartz fibers and a monolithic substrate. SEM images (Figure 13b) confirmed effective catalyst application with well‐distributed 2–4 nm NPs. Additional HR‐TEM and EDS analyses confirmed the uniform presence of Pd, Pt, and Rh particles. The multilayered application of the catalyst was evident from the observed reduction in size of the monolithic substrate. The study also highlighted the significance of NP uniformity in improving catalytic accessibility. The sonochemical deposition method, facilitated by a reducing agent, resulted in uniformly distributed 3–5 nm PGM NPs (Figure 13c). In contrast, without a reducing agent, the NPs tended to clump together, especially at the edges of the NTs. The functionalization of BNNTs was essential for achieving optimal NP dispersion and catalytic efficiency.

Sun's team^[^ ^160^ ^]^ developed Cu‐HNCN catalysts with widely distributed CuN_*x*_ active sites aimed at breaking down antibiotic pollutants, specifically using tetracycline (TET) as a test compound. The research examined the morphology of the catalyst, characteristics of the active sites, and how various reaction parameters, such as H_2_O_2_ concentration, catalyst amount, pH, and the presence of other substances, affected degradation efficiency. DFT calculations provided insights into the electronic structure and charge transfer processes. Through frontier electron density (FED) simulations and liquid chromatography‐mass spectrometry (LC‐MS), the researchers identified active intermediates, proposed a mechanism for catalytic degradation, and outlined the degradation pathway for TET. The Cu‐HNCN catalyst was produced by hydrothermal treatment and calcination of a precursor mixture that included Cu(NO_3_)_2_.3H_2_O, melamine, and cyanuric acid. Initially, the precursor had a structured cavity made of stacked 2D planes, which changed into NTs with mesopores after calcination (Figure 13d). SEM showed porous NTs with a hollow center, measuring 70–90 nm in diameter, which improved SSA and catalytic accessibility. Analysis after calcination confirmed a uniform distribution of Cu, C, and N species (Figure 13e). TEM images displayed a porous single‐layer tube surface with 20–30 nm mesopores, while the lack of Cu‐based NPs or crystalline features indicated the successful removal of Cu–O or Cu–C complexes. HAADF imaging supported the even distribution of Cu, C, and N and emphasized the close proximity of Cu and N atoms, suggesting Cu–N coordination, with no Cu–O interactions detected. Cu species were widely dispersed across disordered 2D HNCN sheets (Figure 13f). This study introduces a new method for stabilizing catalysts and improving electron mobility, making a significant contribution to the development of effective environmental catalysts for wastewater treatment.

Zhang and collaborators^[^ ^161^ ^]^ introduced a new technique for creating alkalinized C nitride NTs (ACNTs), which significantly improved their ability to generate H_2_O_2_. The addition of hydroxyl groups greatly enhanced H_2_O_2_ production, with ACNTs showing a 13‐fold increase compared to unmodified CNs. The research provides a mechanistic understanding of how alkalinization, preservation of morphology, and reduced self‐decomposition contribute to better photocatalytic performance. Initially, the process involved alkalinization after synthesis through melamine hydrolysis and calcination, resulting in CNTs (Figure 13g). However, traditional treatments with NaOH or KOH compromised the integrity of the NTs, leading to fragmented CNs, as shown by SEM (Figure 13h). A revised approach using a KCl/NH_4_Cl mixture partially maintained the morphology but did not achieve adequate alkalinization. Ultimately, pre‐alkalinizing the melamine precursor was the most effective method, preserving structural integrity during polymerization. The resulting ACNTs were over 20 μm in length and 1–3 μm in width, with TEM confirming their hollow interiors. ACNT‐5 displayed a clear tubular structure and a large internal volume (Figure 13i), which enhanced mass transfer in liquid‐phase catalysis. Additionally, ACNT‐5 had a BET SSA of 63.53 m^2^ g^−1^, exceeding that of CNs and indicating greater exposure of active sites. This study introduces a new approach for creating durable, nonmetallic photocatalysts. More generally, it highlights the importance of NTs in TMNs due to their high SSA, efficient charge transport, and adjustable characteristics, making them suitable for use in catalysis, energy storage, and biomedical applications.

#### 1.6.3 2D

The integration of advanced 2D materials into energy conversion and storage systems has attracted considerable interest because of their structural and electronic benefits. Unlike 0D materials, where electron transport is hindered by interactions between self‐assembled nanocrystals, 2D materials provide longer conductive pathways that enhance electron mobility.^[^ ^162^ ^]^ Although noble metals are known for their excellent catalytic and electrochemical properties, their practical use is restricted due to their scarcity and high costs.^[^ ^138^ ^]^ As a result, research has shifted toward alternative materials such as TMOs,^[^ ^163^ ^]^ TMDCs,^[^ ^164^ ^]^ MOFs,^[^ ^165^ ^]^ black phosphorus (BP),^[^ ^166^ ^]^ and TMNs.^[^ ^167^ ^]^ TMNs are particularly attractive because of their high electronic density, stability, tunable BGs, and strong catalytic abilities, making them ideal for electrochemical and photocatalytic uses. Their catalytic effectiveness is attributed to fast electron transport between active sites and electrode interfaces, as well as their impressive performance in SC electrode configurations.^[^ ^168^ ^]^ Nanoscale TMNs have advantages over bulk materials, mainly due to their smaller particle sizes and larger SSA, which improve charge storage and electrochemical responsiveness. However, synthesizing 2D TMNs is challenging due to thermodynamic limitations that make traditional TMOs synthesis methods ineffective. To address these challenges, innovative fabrication techniques, such as salt‐template methods, have been developed, allowing for the successful creation of 2D TMNs. These developments have paved the way for new applications in energy technologies.^[^ ^83^ ^]^

**Figure 14:**
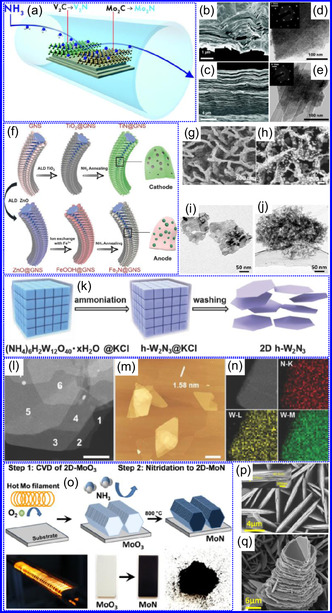
Preparation strategy for 2D TMNs: a–e) Preparation strategy, SEM, and TEM images of Mo_2_CT_*x*_ and V_2_CT_*x*_. Adapted with permission.^[^ ^169^ ^]^ Copyright 2017, Royal Society of Chemistry. f–j) Preparation strategy, SEM, and TEM images of 2D TiN cathode and Fe_2_N anode. Adapted with permission.^[^ ^170^ ^]^ Copyright 2015, Wiley‐VCH. k–n) Preparation strategy, SEM, TEM, and EDS images of h‐W_2_N_3_ NTs. Adapted with permission.^[^ ^171^ ^]^ Copyright 2018, Wiley‐VCH. o–q) Preparation strategy, and SEM image of 2D δ‐MoN NTs. Adapted with permission.^[^ ^172^ ^]^ Copyright 2017, American Chemical Society.

Urbankowski's team^[^ ^169^ ^]^ employed a thermal ammoniation process at 600 °C to create 2D TMNs from Mo_2_CT_*x*_ and V_2_CT_*x*_. This process involves replacing C with N atoms, which come from the breakdown of NH_3_ molecules (**Figure** **14**a). Structural analysis indicated that Mo_2_N largely maintains the morphology of its parent MXene, while V_2_C changes into a composite structure that includes trigonal V_2_N and cubic VN phases. SEM images confirmed that the layered structure of both Mo_2_NT_*x*_ and V_2_NT_*x*_ was preserved after ammoniation, demonstrating structural stability at high temperatures. EDS showed significant N incorporation, with the Mo_2_CT_*x*_ ratio changing from Mo:N:C of 2:0:5.35 to 2:2.04:0 and V_2_CT_*x*_ from 2:0:3.91 to 2:2.46:0.58. This change in composition was positively associated with the increase in ammoniation temperature. The absence of oxide species in SEM further validated the thermal and chemical stability of these materials at temperatures up to 600 °C in the presence of NH_3_ (Figure 14b,c). TEM revealed a distorted HEX structure in Mo_2_NT_*x*_ and a pattern in V_2_NT_*x*_ that matched either the (001) plane of trigonal V_2_N or the (111) plane of cubic VN, consistent with XRD data (Figure 14d,e). Importantly, the synthesized nitrides displayed metallic conductivity, unlike the semiconducting properties of the original carbides. These results underscore ammoniation as a promising method for converting carbide MXenes into highly conductive 2D nitrides, opening up new possibilities for advanced material applications.

Zhu and colleagues^[^ ^170^ ^]^ developed quasi‐solid‐state ASCs utilizing a 2D TiN cathode and an Fe_2_N anode, both supported by highly conductive graphene NSs (GNS), along with a neutral gel electrolyte made of PVA/LiCl. The electrodes were prepared by applying atomic layer deposition (ALD) to create 20 nm films of TiO_2_ and ZnO on GNS substrates. The ZnO was then transformed into FeOOH through a solution‐phase reaction. Thermal annealing of the TiO_2_/GNS and FeOOH/GNS composites in an NH_3_ atmosphere at temperatures of 800 and 600 °C, respectively, resulted in the formation of porous TiN and evenly distributed Fe_2_N NPs on GNS (Figure 14f–h). TEM indicated pore sizes between 3 and 10 nm and confirmed the presence of TiN (111) and (220) planes. XRD analysis confirmed the successful conversion of TiO_2_ into crystalline TiN with a FCC structure (Figure 14i,j). The resulting device demonstrated excellent electrochemical performance, retaining 98% of its capacitance after 20 000 GCD cycles. It also maintained about 99% capacity across scan rates from 10 to 100 mV s^−1^, achieving an energy density of 15.4 Wh kg^−1^ and a power density of 6.4 Wh kg^−1^. This approach, which combines ALD and NH_3_ annealing, presents a promising method for developing TMNs‐based energy storage devices with enhanced rate capability and cyclic durability.

Song's group^[^ ^171^ ^]^ created ultrathin 2D HEX W_2_N_3_ (h‐W_2_N_3_) NSs using a salt‐template method at ambient pressure. The procedure involved applying (NH_4_)_6_H_2_W_12_O_40_.xH_2_O onto a KCl substrate to create a composite precursor (Figure 14k), which was then ammoniated at 750 °C in an NH_3_ environment. After dissolving the KCl template in DI water, freestanding 2D h‐W_2_N_3_ flakes were obtained. SEM confirmed the flakes lateral size (≈5 μm) and ultrathin thickness (≈1.5 nm) (Figure 14l), while TEM showed a single‐crystalline HEX lattice with a 0.25 nm interplanar spacing, corresponding to the (100) plane (Figure 14m). Elemental mapping through EDS further confirmed the HEX arrangement of W atoms (Figure 14n). The strong epitaxial interaction between KCl and h‐W_2_N_3_ significantly lowered the FE, aiding in the synthesis. The material exhibited outstanding HER activity, achieving an onset potential of −30.8 mV and an overpotential of −98.2 mV at a current density of 10 mA cm^−2^.

Chakrapani's team^[^ ^172^ ^]^ proposed a two‐step strategy for creating μm‐scale 2D nanostructures of δ‐MoN. The process begins with the fabrication of MoO_3_ nanostructures using hot‐filament CVD, followed by a reduction annealing step in an NH_3_ environment, which converts the oxide into its nitride form. This adaptable technique allows for the transformation of TMOs into nitrides, sulfides, or carbides by suitable gaseous environments like NH_3_, H_2_S, or CH_4_. The resulting δ‐MoN electrode exhibits remarkable electrochemical stability, consistently delivering a capacity of 320 mAh g^−1^ over 200 cycles without any noticeable structural or performance degradation (Figure 14o). SEM confirms that the morphology of the MoO_3_ nanostructures is preserved after the nitridation process. The nanostructures, produced on FTO substrates, are vertically aligned, with crystallite widths ranging from 20 μm to 30 μm and thicknesses between 5 nm and 40 nm. This synthesis is scalable for substrate areas of at least 1 inch, depending on the size of the filament used. The nanostructures were removed from the FTO substrate, mixed with a conductive binder, and applied to Cu foil to function as electrodes in LIBs. Post‐synthesis SEM analysis confirmed the retention of structural integrity, and electrochemical testing in a half‐cell LIB configuration demonstrated their energy storage capabilities (Figure 14p,q). Overall, the research highlights the growing interest in 2D TMNs, which provide high SSA, improved charge transport, and structural adaptability, making them promising candidates for energy storage and generation applications. Recent advancements in synthetic techniques, such as salt templating and ammoniation, have addressed thermodynamic challenges, paving the way for the application of TMNs in SCs and batteries.

Significant research has focused on creating highly efficient photocatalysts for solar energy conversion, especially given the rising global energy needs and environmental pollution.^[^ ^173^ ^]^ Following the groundbreaking research by Wang's team^[^ ^174^ ^]^ on OWS using g‐C_3_N_4_ as a photocatalyst, this material has attracted considerable interest due to its strong chemical and thermal stability, adjustable band structure, and effective absorption of visible light. However, its practical photocatalytic efficiency is limited by a relatively low SSA and the quick recombination of photoexcited electron–hole pairs.^[^ ^175^ ^]^ Several strategies have been suggested to improve its photocatalytic performance, including the construction of heterostructures, doping with heteroatoms, creating N vacancies, and manipulating its morphology and structural characteristics. Bulk g‐C_3_N_4_ is usually produced through the thermal polycondensation of precursors like melamine, urea, or thiourea, resulting in a layered structure held together by van der Waals forces, similar to graphene.^[^ ^176^ ^]^ To address its inherent limitations, researchers have investigated exfoliating it into 2D nanostructures to enhance SSA and improve electron transport. Techniques such as liquid‐phase exfoliation, mechanical milling, and post‐synthetic thermal etching have been utilized for this purpose.^[^ ^177^ ^]^ Among these, thermal etching is particularly appealing due to its cost‐effectiveness, simplicity, and environmental friendliness. Recent research has shown that rapid thermal treatment can lead to the creation of porous g‐C_3_N_4_ structures with pores around 50 nm in size, which increases the density of active sites and enhances mass and charge transfer. However, achieving scalable and controllable synthesis of few‐layered (*fl*) porous g‐C_3_N_4_ continues to be a significant challenge.^[^ ^178^ ^]^

**Figure 15:**
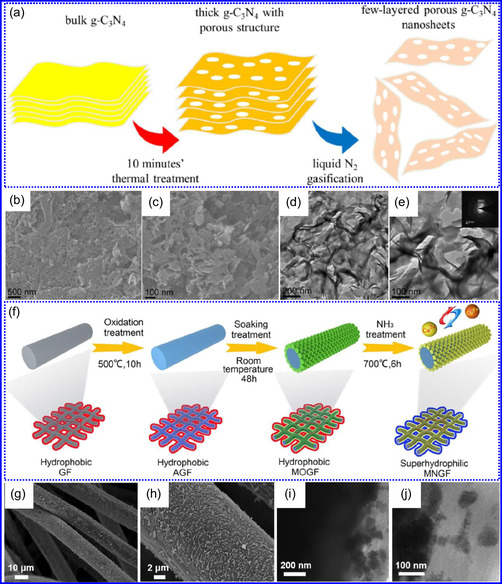
a–e) Preparation strategy, SEM, TEM, and HR‐TEM images of fl‐P‐CN. Adapted with permission.^[^ ^179^ ^]^ Copyright 2020, Elsevier B.V. f–j) Preparation strategy, SEM, and TEM images of MNGF. Adapted with permission.^[^ ^180^ ^]^ Copyright 2022, Elsevier B.V.

Shimada and collaborators^[^ ^179^ ^]^ introduced an efficient two‐step methodology for synthesizing porous *fl*‐g‐C_3_N_4_ nanoplates. The process began with heating pristine g‐C_3_N_4_ at 750 °C in air, which caused interlayer expansion and partial etching by weakening van der Waals forces. This was followed by immersion in liquid N_2_ (*l*‐N_2_), which led to rapid gasification and facilitated the exfoliation of the g‐C_3_N_4_ layers. The combination of high‐temperature calcination and cryogenic exfoliation successfully produced ultrathin, porous 2D structures (**Figure** **15**a). XRD analysis indicated that both bulk CN and *fl* porous CN (fl‐P‐CN) maintained similar crystalline structures, with a noticeable shift in the (002) peak from 27.4° to 27.9°, reflecting a decrease in interlayer distance from 0.326 to 0.32 nm and an increase in stacking density. The reduced intensity and broadening of the peaks suggested a disruption in interlayer ordering. FTIR spectra of *fl*‐P‐CN displayed clearer characteristic absorption bands, indicating the removal of amorphous materials. Raman spectroscopy, conducted with 266 nm UV excitation, revealed vibrational modes around 970 and 700 cm^−1^, linked to symmetrical N‐breathing in triazine units. The reduced G‐band at 1634 cm^−1^ indicated enhanced structural order and a more delocalized π‐electron system within the heptazine framework. SEM (Figure 15b,c) and TEM (Figure 15d) analyses confirmed a change in morphology from stacked lamellae to wrinkled, meso‐/macroporous nanoplates in *fl*‐P‐CN, with pore sizes between 10 nm and 100 nm. HR‐TEM imaging (Figure 15e) revealed nanopores, distorted lattice fringes, and C vacancies. The final product had a NS thickness of 8.5 nm, significantly thinner than the original CN (≈55 nm), which contributed to improved charge separation and enhanced photocatalytic activity.

Sun's team^[^ ^180^ ^]^ proposed a new method to improve cerium (Ce)‐based redox flow batteries (RFBs) by using MoN as a highly conductive and super hydrophilic electrocatalyst. Their research showed that it is possible to create scalable and dense arrays of MoN nanoplatelets directly on graphite felt (GF) through a nitriding strategy (Figure 15f). The MoN‐coated GF (MNGF) exhibited a well‐structured, open nanoplate morphology that enhanced surface exposure to the electrolyte, thus speeding up redox kinetics. Contact angle measurements indicated the extreme hydrophilicity of MNGF, with angles close to 0°, reflecting excellent wettability and catalytic accessibility. XRD analysis confirmed the crystalline nature of the materials: the original GF displayed typical peaks at 26° and 44°, while MNGF showed patterns consistent with pure MoN. The morphological changes from pristine GF to AGF, MOGF, and MNGF were further illustrated using SEM (Figure 15g,h) and TEM (Figure 15i,j). While GF retained a porous 3D structure, AGF underwent oxidation that functionalized its surface without altering its structure. MOGF showed uniform deposition of MoO_2_, whereas MNGF revealed hierarchical micro/nanostructures that facilitated ion transport and accessibility to active sites. TEM images indicated a 350 nm thick MoN layer, with lattice fringes corresponding to the (200) and (002) planes, and SAED identified reflections at (101), (002), and (201), confirming a polycrystalline MoN structure. This research highlights the electrocatalytic potential of MoN nanoplates in enhancing Ce redox processes and improving RFB efficiency. Coupled with advancements in g‐C_3_N_4_ photocatalyst development through thermal etching and *l*‐N_2_ processing, these findings represent significant progress in material innovation for energy storage and conversion technologies.

#### 1.6.4 3D

3D TMNs are distinguished by their complex 3D designs, which often feature interconnected frameworks or porous structures. These characteristics greatly enhance the SSA and the density of active sites, improving charge transfer processes and overall material stability, both of which are essential for advanced applications.^[^ ^149^ ^]^ However, to achieve optimal electrochemical activity, it is crucial to ensure that active sites are accessible and that charge transport within the electrode is efficient. This challenge is particularly pronounced in commercial electrodes that are thicker than a few microns or have mass loadings greater than 10 mg cm^−2^, where accessibility to active sites and charge mobility can be restricted.^[^ ^181^ ^]^ To address these issues, the creation of 3D hierarchical TMN structures is strongly encouraged. These designs aim to maximize electrolyte porosity and enhance ion diffusion pathways, leading to better material utilization. By integrating hierarchical porosity at various length scales, 3D structures facilitate the exposure of active sites, improve charge transfer kinetics, and maintain structural integrity even under high mass loading conditions. As a result, well‐designed 3D TMNs allow for the use of thicker electrodes without compromising efficiency or electrochemical stability.^[^ ^182^ ^]^

**Figure 16:**
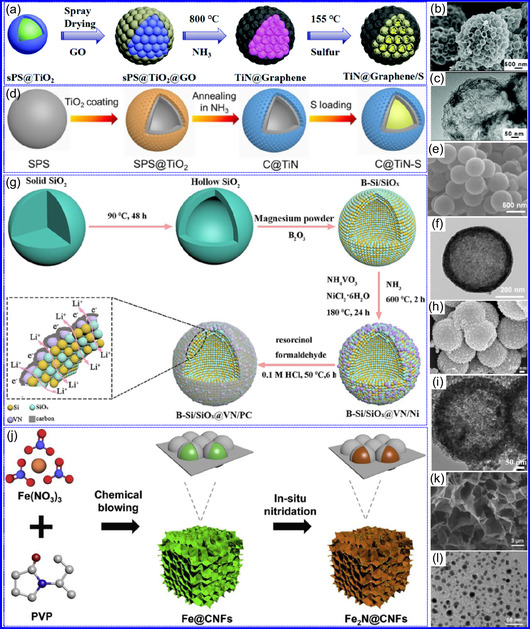
Preparation strategy for 3D TMNs: a–c) Preparation strategy, SEM, and TEM images of PTG. Adapted with permission.^[^ ^183^ ^]^ Copyright 2019, Royal Society of Chemistry. d–f) Preparation strategy, SEM, and TEM images of C@TiN. Adapted with permission.^[^ ^184^ ^]^ Copyright 2019, Elsevier B.V. g–i) Preparation strategy, SEM, and TEM images of B‐Si/SiO_*x*_@VN/PC. Adapted with permission.^[^ ^185^ ^]^ Copyright 2019, American Chemical Society. j–l) Preparation strategy, SEM, and TEM images of Fe_2_N@CNFs. Adapted with permission.^[^ ^186^ ^]^ Copyright 2020, Elsevier B.V.

Luo and colleagues^[^ ^183^ ^]^ employed a spray‐drying technique to create a 3D pomegranate‐shaped TiN@graphene (PTG) composite, which showed impressive capabilities as an S host for LSBs. The composite demonstrated exceptional electrochemical stability, thanks to the synergistic interactions between the 3D graphene framework and the polar TiN NS (**Figure** **16**a). The PTG/S electrode achieved an initial discharge capacity of 1249 mAh g^−1^, maintained 810 mAh g^−1^ after 200 cycles at 0.5 C, and held 663 mAh g^−1^ after 500 cycles at 1 C, resulting in capacity retention of 78%. SEM was used to examine the morphology of the composites, including PTG and graphene spherical clusters (PG). Before carbonization, the samples exhibited a 3D pomegranate‐like structure (Figure 16b). Following carbonization in an NH_3_ atmosphere, both PG and PTG preserved their spherical shapes, with sizes between 1 and 5 μm, derived from primary NS of about 400 nm in diameter. In the PG samples, some NS showed signs of collapse, while in PTG, the TiN NS were uniformly surrounded by rGO shells. TEM confirmed that PTG consisted of hollow TiN NS coated with graphene layers, with distinct lattice fringes corresponding to the (200) plane of cubic TiN observed (Figure 16c). This architecture significantly enhances the chemisorption and conversion of LiPSs, thereby contributing to its excellent electrochemical performance.

Ding's group^[^ ^184^ ^]^ created a multifunctional polysulfide mediator made of C‐hollow NS attached to 3D TiN (C@TiN) dual‐shell hollow nanostructures (Figure 16d). This innovative design combines physical confinement, chemical adsorption, and catalytic conversion of S species. In comparison to TiO_2_‐based materials, the C@TiN‐S composites (containing ≈70 wt% S) show enhanced reaction kinetics and better polysulfide retention, making them highly effective cathode materials for LSBs. The C@TiN‐S electrode achieved a reversible capacity of 453 mAh g^−1^, a high coulombic efficiency (CE) of around 99%, and minimal capacity loss (0.0033% per cycle) over 300 cycles at a high rate of 3 C. When the S loading increased to 4.2 mg cm^−2^, the electrode sustained a stable capacity of 820 mAh g^−1^ over 150 cycles at 0.2 C. Structural and electrochemical analyses (Figure 16e,f) confirmed strong interactions between TiN and LiPSs, which aided in the catalytic breakdown of long‐chain Li_2_S_8_ and the oxidation of Li_2_S during cycling. This research presents a promising approach to improving the performance of LSBs by using multifunctional mediators.

Chen and collaborators^[^ ^185^ ^]^ successfully created 3D hollow B‐Si/SiO_*x*_@VN/PC NS using a combination of modified magnesiothermic reduction, a solvothermal method, and RF (resorcinol‐formaldehyde) polymer coating. These NS consist of a B‐doped Si/SiO_*x*_ core that contains VN NPs and is surrounded by a partially graphitized, nanoporous C shell. The addition of B doping and VN NPs significantly boosts electrical conductivity, enhancing both rate capability and cycling performance. The C shell also contributes to structural stability during extended cycling by preventing collapse (Figure 16g). SEM images show uneven surface morphologies due to NP decoration. Without the B‐Si/SiO_*x*_ framework, VN tends to form agglomerated NPs (30–50 nm), which could lead to faster electrode degradation. After RF coating and thermal treatment, the NS maintain their structural integrity and display smoother surfaces. Structural analyses of B‐Si/SiO_*x*_@VN/C and B‐Si/SiO_*x*_@C show similar morphologies (Figure 16h). TEM confirms a hollow structure with an outer shell made of fine nanograins. The porous texture, with VN NPs distributed across the rough surfaces, facilitates electrolyte penetration. HR‐TEM reveals a C layer containing Si nanograins and VN NPs. Furthermore, N_2_ adsorption‐desorption tests validate the mesoporous characteristics and high SSA, which help reduce volume changes during cycling (Figure 16i). This approach presents a promising method for improving both electrical conductivity and structural stability in electrode materials.

Wu's team^[^ ^186^ ^]^ developed a novel approach for creating graphene‐encapsulated TMNs, like Fe_2_N, integrated within 3D CNSs. This technique combines chemical puffing with in situ nitridation, resulting in 3D Fe_2_N@CNFs that exhibit significantly improved performance in LIBs (Figure 16j). The Fe@CNFs demonstrate the structural development of 3D CNSs, featuring macropores several μm wide and NSs ≈40 nm thick. These CNSs show a consistent distribution of Fe NPs (Figure 16k). TEM confirms the presence of core‐shell structures, where Fe NPs are surrounded by *fl* graphene, a result of the catalytic effect of Fe during PVP pyrolysis. After in situ nitridation at 500 °C in an NH_3_ environment, the CNSs framework remains stable while the Fe NPs transform into Fe_2_N, still encased in graphene shells (Figure 16l). This structure prevents NP agglomeration, promotes electrolyte ion diffusion, and improves electronic conductivity. Consequently, the 3D Fe_2_N@CNFs exhibit excellent Li storage capabilities, sustaining performance over 500 cycles and achieving a high‐rate capacity of 215 mAh g^−1^ at 10 A g^−1^. Additionally, their use in fully assembled LIBs shows promising discharge capacities, highlighting their practical applicability. Overall, these results emphasize the essential role of 3D TMNs in addressing challenges related to thick electrodes by enhancing porosity, ion diffusion, and structural integrity. TMNs such as TiN and Fe_2_N hold significant potential for improving the electrochemical performance of next‐generation energy storage devices like LIBs and LSBs.

Nanoflowers (NF), a distinctive nanostructured form of TMNs, significantly improve performance in a variety of applications. Their petal‐like structure provides a high SSA, which increases interaction with reactants and enhances catalytic activity.^[^ ^187^ ^]^ This design also reveals numerous active sites, thereby improving electrocatalytic processes such as the HER and OER.^[^ ^188^ ^]^ Additionally, morphology promotes efficient charge transport and excellent electrical conductivity, making it beneficial for fuel cells and energy storage systems. The NF structure helps prevent particle agglomeration and sintering, thereby improving structural stability.^[^ ^189^ ^]^ Owing to their quantum effects and elevated surface‐to‐volume ratios, NF exhibit exceptional optical and electronic characteristics, rendering them well‐suited for sensor technologies and photonic systems.^[^ ^190^ ^]^ Collectively, the incorporation of NF in TMNs enhances catalytic efficiency, electrical characteristics, and structural integrity, promoting their utility in sustainable energy and environmental uses.

**Figure 17:**
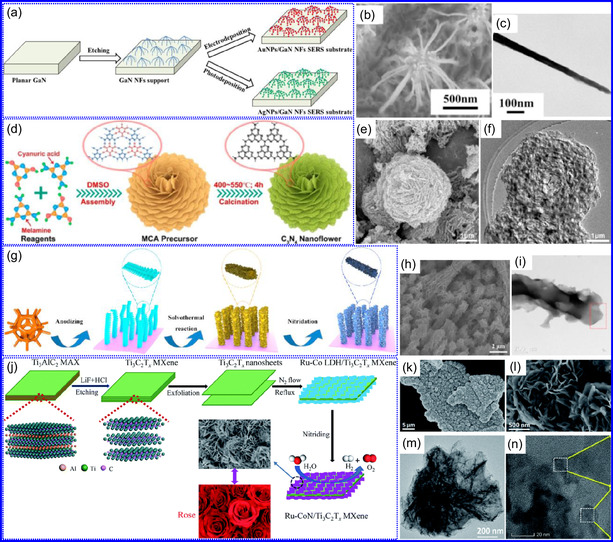
a–c) Preparation strategy, SEM, and TEM images of 3D GaN NF. Adapted with permission.^[^ ^191^ ^]^ Copyright 2017, Elsevier B.V. d–f) Preparation strategy, SEM, and TEM images of C_3_N_4_ NF. Adapted with permission.^[^ ^192^ ^]^ Copyright 2023, American Chemical Society. g–i) Preparation strategy, FE‐SEM, and FE‐TEM of Cu_3_N@NiCo‐N/Cu. Adapted with permission.^[^ ^193^ ^]^ Copyright 2022, Elsevier B.V. j–n) Preparation strategy, SEM, and TEM images of Ru‐CoN/Ti_3_C_2_T_*x*_. Adapted with permission.^[^ ^194^ ^]^ Copyright 2021, Royal Society of Chemistry.

Pan and colleagues^[^ ^191^ ^]^ successfully developed SERS substrates utilizing 3D GaN NFs synthesized through metal‐assisted photochemical etching (MaPEtch), a technique characterized by its minimal lattice distortion and structural adaptability. Following the synthesis, the 3D GaN NFs were functionalized with Au NPs using in situ electroplating or Ag NPs through in situ photo‐deposition, resulting in 3D Au NPs/GaN NFs or Ag NPs/GaN NFs SERS substrates (**Figure** **17**a). SEM analysis indicated an increase in surface roughness post‐electrodeposition, revealing ≈20 nm Au NPs that were uniformly arranged and closely packed, with interparticle gaps measuring less than 5 nm. The dimension of the gap (d) being smaller than the NPs size (D) facilitates a highly concentrated electromagnetic field between the Au NPs. CV curves illustrated the dynamics of the electrodeposition, where initial peaks represented the conversion of Au(III) to Au(0), subsequently leading to NP formation. The observed variations in reduction peak intensity during successive scans suggested that NP growth preferentially occurred on existing Au NPs, while the reduction of cathodic current was attributed to the depletion of Au(III) ions. Intriguingly, the formation of fragile Au NPs at lower potentials was linked to immediate nucleation and HER, which hindered NP growth. XRD analysis confirmed the presence of FCC Au structures, while EDS validated the elemental composition of Au. TEM imaging highlighted the structural characteristics of the 3D GaN NFs, composed of dispersed GaN NWs (Figure 17b), which exhibited smooth surfaces and high‐quality single‐crystalline properties, oriented along the (0002) axis and measuring ≈1 μm in length. To improve the reproducibility of SERS measurements, Raman spectra were collected using a 10× objective, ensuring that the laser focus encompassed a range of GaN NFs (Figure 17c). The enhanced 3D structures significantly outperformed traditional 2D planar and porous GaN substrates, yielding much stronger Raman signals, which underscores the importance of substrate morphology. These innovative substrates demonstrated excellent repeatability, stability, and sensitivity, achieving detection of bovine serum albumin at concentrations as low as 10^−6^ M, thus highlighting their potential as effective biosensors for SERS applications.

Niu and colleagues^[^ ^192^ ^]^ proposed a novel approach to improve electrochemiluminescence (ECL) performance by altering the crystalline structure of C_3_N_4_ NF. They synthesized porous C_3_N_4_ NF through a two‐step procedure that involved the self‐assembly of melamine and cyanuric acid in a DMSO solution to form supramolecular melamine‐cyanuric acid (MCA) precursors, which were then thermally annealed at varying temperatures in an ambient air muffle furnace (Figure 17d). The synthesis was confirmed using XRD and FTIR. The XRD patterns of the MCA precursor displayed sharp peaks indicative of a HEX supramolecular structure, characterized by hydrogen‐bonded chains and π‐stacked aromatic rings. FTIR analysis verified the existence of N‐H···O and N‐H···N interactions. Initial polymerization was observed at 350 °C, although complete formation of C_3_N_4_ had yet to occur. Between the temperatures of 400–550 °C, the XRD data for C_3_N_4_ NF revealed distinct peaks near 13° and 27°, corresponding to the (100) and (002) planes, with peak sharpening at higher temperatures suggesting improved crystallinity and reduced interlayer spacing. These structural changes facilitate better charge transport between heptazine layers, a crucial aspect of ECL enhancement. Morphological assessments via SEM and TEM indicated that the MCA precursors formed uniform, flower‐type NPs (≈2–3 μm) with NSs ≈50 nm thick. Following calcination, the C_3_N_4_ NF maintained their flower‐type morphology but exhibited hollow interiors, likely due to Ostwald ripening during crystallization (Figure 17e). TEM images showed that the final structures comprised interconnected, rippled, and shortened NSs with irregular pores. These structural features enhance the SSA, improve electrolyte diffusion, and provide a greater number of active reaction sites (Figure 17f). Importantly, the higher‐crystallinity C_3_N_4_ NF exhibited substantially stronger and more stable ECL signals when in the presence of K_2_S_2_O_8_ compared to their less‐crystalline counterparts.

Liu's team^[^ ^193^ ^]^ achieved a breakthrough in electrode materials with the successful development of a multilayer porous heterostructure, Cu_3_N@NiCo‐N/Cu, through a continuous multi‐step synthesis. This innovative electrode demonstrates exceptional electrochemical characteristics, boasting a C_sp_ of 8.49 F cm^−2^ at 1 mA cm^−2^, while impressively maintaining 90.1% of its initial capacity after 8000 GCD cycles. As illustrated in Figure 17g, the fabrication process initiates with the anodic growth of blue Cu(OH)_2_ NRs on a Cu foam substrate, which is subsequently followed by the hydrothermal deposition of yellow, petal‐like NiCo(OH)_2_ NSs onto these NRs. The hydroxides undergo a postnitridation that transforms them into gray‐brown nitrides. FE‐SEM analysis confirms the consistent formation of Cu(OH)_2_ NRs, which display a 3D network morphology, supported by XRD peaks characteristic of the material. Following the hydrothermal treatment, the NiCo(OH)_2_ NSs assemble into a flower‐type structure atop the NRs. Notably, the nitridation successfully preserves the architecture of the NRs, resulting in a starfish‐like cross‐section that optimizes accessibility for electrolyte interaction. XRD patterns indicate the presence of distinct peaks related to Cu_3_N, with the final synthesized material exhibiting a more ordered and regular organization compared to its analogs (CuO@NiCo‐O/Cu and Cu(OH)_2_@NiCo‐N/Cu), all retaining that characteristic flower‐type morphology, as shown in Figure 17h. TEM analysis further reveals a uniform coating of NiCo‐N flakes on the Cu_3_N NRs, which measure ≈220 nm. Enhanced magnified images illustrate a double‐layered, porous shell structure that is conducive to ion transport. The presence of lattice fringes verifies the crystallinity of Cu_3_N, and elemental mapping showcases a uniform distribution of Cu, Ni, Co, and N throughout the structure. Notably, the analysis indicates a higher N content and a Ni‐to‐Co atomic ratio of around 2:1 in the Cu_3_N@NiCo‐N/Cu, corroborating the successful synthesis as depicted in Figure 17i. When incorporated into ASCs paired with an rGO anode, this device achieves an impressive energy density of 124.9 μWh cm^−2^ at a power density of 1.6 mW cm^−2^.

Yan and colleagues^[^ ^194^ ^]^ pioneered the synthesis of Ru‐doped, rose‐type CoN NF composites, termed Ru‐CoN/Ti_3_C_2_T_*x*_, through a novel self‐assembly and nitridation strategy on a Ti_3_C_2_T_*x*_ substrate, as depicted in Figure 17j. The process began with the preparation of Ti_3_C_2_T_*x*_ NSs by selectively removing Al layers from Ti_3_AlC_2_ using an HCl/LiF solution, followed by exfoliation and subsequent drying. These NSs, exhibiting a delicate and uniform morphology as characterized by SEM, served as a substrate for the deposition of Ru‐CoN nanoclusters. To form the Ru–Co LDH/Ti_3_C_2_T_*x*_ precursors, Ti_3_C_2_T_*x*_ was refluxed with Co^2+^ and Ru^3+^ salts alongside urea at 100 °C within a N_2_ atmosphere. The final architecture of Ru‐CoN/Ti_3_C_2_T_*x*_ was realized through in situ nitridation at 380 °C using NH_3_ gas. SEM and HR‐SEM analyses verified the preservation of a rose‐type porous structure formed by interconnected NSs, which ensures a high density of active sites while facilitating enhanced mass and charge transfer (Figure 17k,l). TEM and HR‐TEM imaging illustrated the uniform distribution of ultrathin NSs on the Ti_3_C_2_T_*x*_ surface, showing distinct lattice fringes that correspond to the (111) and (200) planes of crystalline CoN (Figure 17m,n). The amalgamation of Ru‐CoN with Ti_3_C_2_T_*x*_, paired with the innovative nanostructure and customized electronic characteristics, led to superior HER and OER performance alongside remarkable durability during OWS in alkaline environments. This design approach highlights a promising avenue for the advancement of MXene‐based electrocatalysts.

Yolk‐shell nanostructures in TMNs are instrumental in enhancing the efficacy of various applications, such as environmental remediation, energy storage, and catalysis.^[^ ^195^ ^]^ Their unique design, characterized by a hollow interior encased in a porous outer shell, significantly amplifies the SSA and the availability of active sites, thereby fostering improved catalytic and electrochemical activities. Furthermore, this architecture facilitates effective mass and electron transport, leading to increased conductivity and expedited reaction kinetics.^[^ ^196^ ^]^ The yolk‐shell configuration also provides structural stability, accommodating volumetric changes during operational cycles, thus reducing material degradation and prolonging device longevity. Additionally, the shell structure prevents core aggregation, thereby ensuring a homogeneous distribution of active components critical for reliable performance.^[^ ^197^ ^]^ Beyond these intrinsic advantages, yolk‐shell designs allow for the controlled release of active species, which is particularly advantageous in drug delivery systems and environmental remediation practices. The synergistic interactions between core and shell materials further augment their functional capabilities, establishing yolk‐shell TMNs as leading candidates for advanced material applications.^[^ ^198^ ^]^

**Figure 18:**
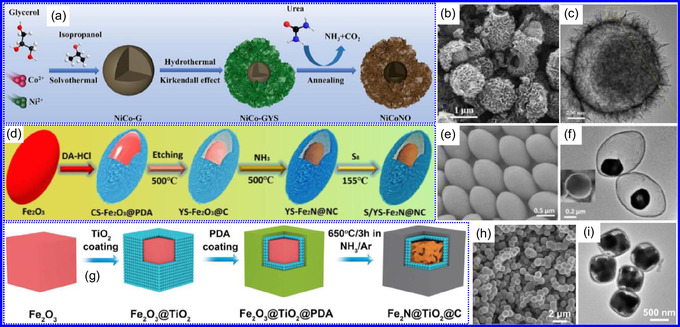
a–c) Preparation strategy, SEM, and TEM images of NiCoNO. Adapted with permission.^[^ ^199^ ^]^ Copyright 2023, Wiley‐VCH. d–f) Preparation strategy, FE‐SEM, and TEM images of YS‐Fe_2_N@NC. Adapted with permission.^[^ ^200^ ^]^ Copyright 2022, Elsevier B.V. g–i) Preparation strategy, SEM, and TEM images of double‐shelled Fe_2_N@TiO_2_@C microcubes. Adapted with permission.^[^ ^201^ ^]^ Copyright 2021, Wiley‐VCH.

Liu and colleagues^[^ ^199^ ^]^ formulated a scalable method for the synthesis of yolk‐shell heterogeneous structures composed of NiCo‐N and oxides (NiCoNO), achieved through the use of metal glycerate precursors combined with urea as a N source. This novel approach, differing from traditional oxide/hydroxide and NH_3_‐based methods, produces structures with significant meso‐ and macro‐porosity, thereby enhancing mass and charge transport characteristics for energy storage applications. The resulting NiCoNO materials exhibit a remarkable C_sp_ of 1878 F g^−1^ at 1 A g^−1^, alongside a capacity retention of 83.9% after 5000 cycles at 10 A g^−1^, demonstrating exceptional pseudocapacitive characteristics. Furthermore, solid‐state ASCs constructed with NiCoNO and activated carbon (AC) electrodes achieved a notable energy density of 64.2 Wh kg^−1^, sufficient to illuminate an LED for several min. The preparation pathway depicted in **Figure** **18**a initiates with the coordination of Ni^2+^, Co^2+^, and glycerol, leading to the formation of NiCo‐G precursors characterized by a uniform spherical shape with a radius of 500 nm. Following this, a hydrothermal treatment at 150 °C facilitates hydrolysis, resulting in the development of a yolk‐shell framework embedded with NSs. The final nitridation process involves the pyrolysis of urea into NH_3_, which interacts with Ni^2+^/Co^2+^ ions to produce TMNs while preserving the yolk‐shell configuration. Concurrently, oxide formation gives rise to the NiCo‐N and NiCo‐O heterostructures. SEM images confirm the integrity of the yolk‐shell architecture (Figure 18b), whereas TEM analysis reveals a thin and porous structure that promotes improved electron mobility. HR‐TEM observations further identify lattice fringes attributed to CoN (111) and CoNiO_2_ (200) planes, with interplanar spacings of 0.248 and 0.211 nm, respectively (Figure 18c). This innovative preparation approach paves the way for the development of high‐performance TMNs‐based materials for next‐generation SCs and energy devices.

Aslam's group^[^ ^200^ ^]^ utilized a simple hydrothermal synthesis to create Fe_2_N embedded within a N‐C yolk‐shell structure (YS‐Fe_2_N@NC), tailored to serve as S hosts in room‐temperature sodium‐sulfur batteries (RT‐NSBs). This innovative hollow and microporous configuration features a vacuum‐like surface that effectively accommodates volume fluctuations while stabilizing both S NPs and soluble polysulfides. The N‐C shell not only enhances electrical conductivity but also limits polysulfide diffusion through a combination of physical confinement and chemical interactions, culminating in impressive electrochemical performance. The preparation process (Figure 18d) commenced with the formation of a damson blue‐hued Fe_2_O_3_ precursor through hydrothermal techniques. Subsequent C coating established a core‐shell structure (CS‐Fe_2_O_3_@C), which was then processed through etching to yield a yolk‐shell design (YS‐Fe_2_O_3_@C). A final thermal treatment in an NH_3_ environment resulted in the formation of YS‐Fe_2_N@NC. Extensive characterization methods, including HR‐TEM, SAED, XRD, Raman spectroscopy, and BET analysis, validated the material's crystallinity, composition, and porosity. The precursor demonstrated a high SSA of 337.2 m^2^ g^−1^, making it well‐suited for polysulfide adsorption. Following S loading through melt‐diffusion, the BET SSA dramatically decreased to 22.4 m^2^ g^−1^, indicating successful S incorporation. FE‐SEM and TEM images (Figure 18e,f) illustrated that S infiltrated the N‐C shell and deposited onto the Fe_2_N core. This study introduces a pioneering polar yolk‐shell catalyst system poised to enhance RT‐NSB performance.

Du's group^[^ ^201^ ^]^ has developed double‐shelled Fe_2_N@TiO_2_@C yolk‐shell sub microcubes to serve as anodes in potassium‐ion batteries (PIBs). This innovative architecture effectively accommodates the volume changes that occur during electrochemical cycling while providing ample K^+^ ion interaction sites and improving electrical conductivity (Figure 18g). Consequently, the anode demonstrates a high C_sp_, outstanding cycling stability, and excellent rate performance. Elemental mapping through EDS revealed a homogeneous distribution of Fe_2_N, TiO_2_, and C within the microstructure, with Fe_2_N located at the center, TiO_2_ forming the intermediate layer, and an N‐C layer enclosing the entire structure. XPS analysis confirmed the incorporation of C and N dopants, alongside various chemical bonds. N_2_ adsorption–desorption experiments exhibited a SSA of 19.7 m^2^ g^−1^ and an average pore diameter of 16 nm, indicating strong electrolyte accessibility and efficient K‐ion diffusion pathways. The Fe_2_O_3_ precursors were synthesized through straightforward co‐precipitation, resulting in particles ≈650 nm in size with a smooth morphology. Following the deposition of the TiO_2_ shell, the surface roughened and adopted a core‐shell structure (Figure 18h), with TEM affirming the uniform coverage of TiO_2_ (Figure 18i). XRD analyses confirmed the presence of both cubic Fe_2_O_3_ and anatase TiO_2_ phases. The dual‐shell construction was finalized with the addition of a polydopamine (PDA) layer. This research significantly advances the field of robust TMN‐based anodes for PIBs. Overall, yolk‐shell nanostructures play a critical role in enhancing SSA, promoting mass and electron transfer, and preserving structural integrity, as demonstrated by various recent developments such as NiCoNO, Fe_2_N@NC, and Fe_2_N@TiO_2_@C in SCs, NSBs, and PIBs.

Recent advancements in catalytic performance have predominantly centered on techniques such as heteroatom doping, defect engineering, interface modulation, and morphological control. Notably, surface engineering has garnered significant interest due to its capacity to modulate electronic configurations, enhance charge mobility, and reveal catalytically active sites.^[^ ^202^ ^]^ For instance, heterostructures like MoS_2_/Ni_3_S_2_ and MoS_2_/Fe_5_Ni_4_S_8_ demonstrate remarkable bifunctional catalytic activity for both the OER and HER.^[^ ^203^ ^]^ Furthermore, catalysts with 3D hierarchical architectures, such as NiFe‐LDH@NiCoP, exhibit enhanced performance attributed to their expanded SSAs and efficient gas release mechanisms.^[^ ^204^ ^]^ However, despite these significant strides, 3D NiMo‐based nitrides, while promising for catalytic applications, still necessitate further enhancements in both activity and durability, especially in the realm of energy‐efficient urea electrolysis aimed at H_2_ generation. Consequently, the pursuit of cost‐effective, advanced 3D hierarchical NiMo‐derived heterostructured nitrides is crucial for the improvement of UOR and HER processes.^[^ ^205^ ^]^

**Figure 19:**
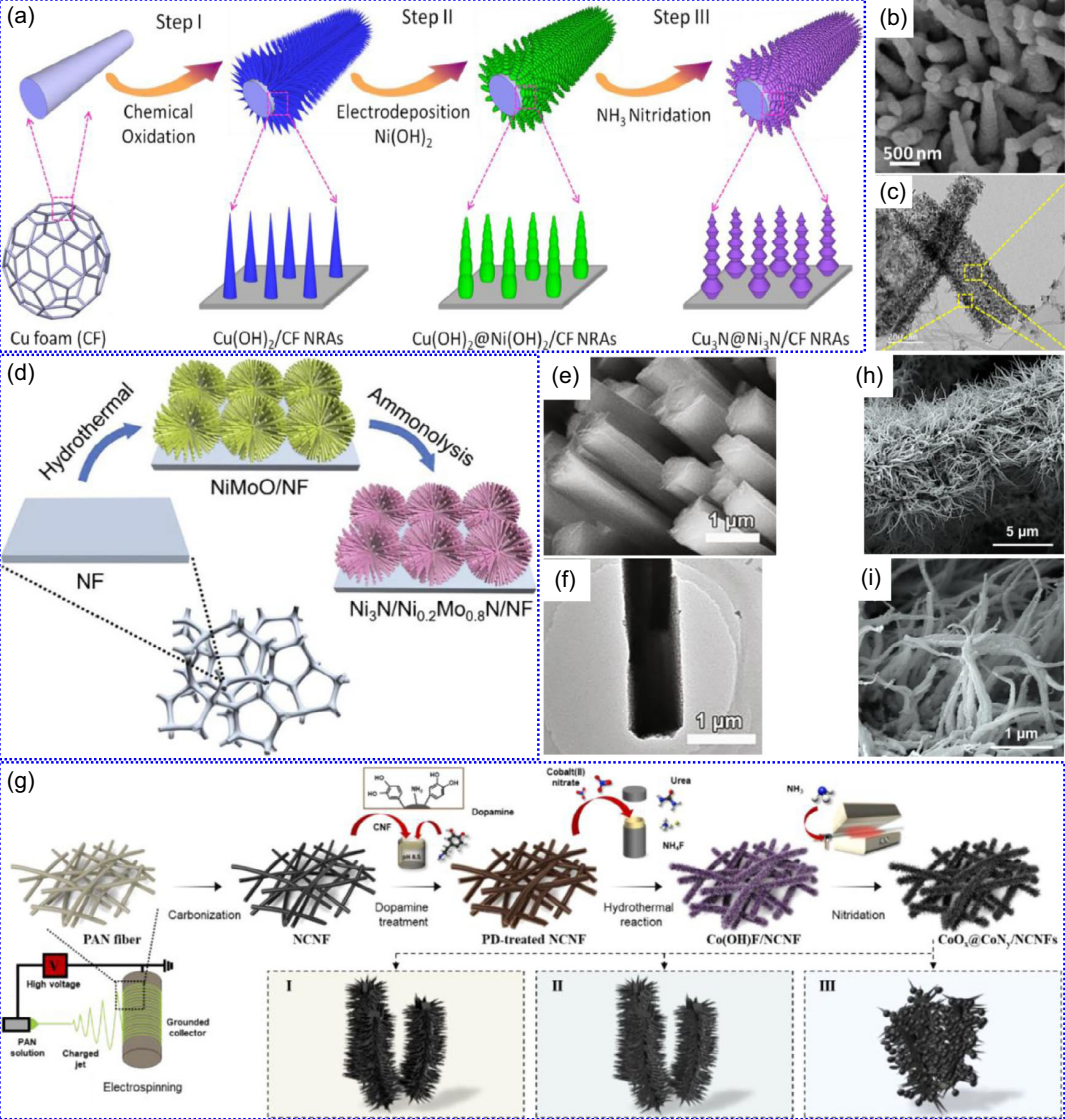
a–c) Preparation strategy, SEM, and TEM images of Cu_3_N@Ni_3_N/CF NRAs. Adapted with permission.^[^ ^206^ ^]^ Copyright 2023, Elsevier B.V. d–f) Preparation strategy, SEM, and TEM images of Ni_3_N@Ni_0.2_Mo_0.8_N/NF. Adapted with permission.^[^ ^207^ ^]^ Copyright 2021, Elsevier B.V. g–i) Preparation strategy, and SEM of CoO_*x*_@CoN_*y*_/NCNF. Adapted with permission.^[^ ^208^ ^]^ Copyright 2021, American Chemical Society.

Jun and his group^[^ ^206^ ^]^ tackled the challenge of preparing Cu_3_N@Ni_3_N NRAs on CFs, creating a self‐supported electrode material for solid‐state SCs. The 3D nanostructured architecture promotes rapid charge transport, enhances the availability of active sites, and exhibits excellent electrical conductivity due to the synergistic interactions between the Cu_3_N and Ni_3_N phases, as illustrated in **Figure** **19**a. This innovative design yielded outstanding electrochemical performance, demonstrating a C_sp_ of 390.5 mAh g^−1^, remarkable cycling stability with 94.9% retention after 10 000 cycles, and robust rate capability. A complete solid‐state SC device utilizing Cu_3_N@Ni_3_N NRAs in conjunction with AC electrodes achieved an energy density of 71.8 Wh kg^−1^, while maintaining structural integrity over prolonged usage. Morphological analysis via FE‐SEM revealed vertically aligned Cu(OH)_2_ NRAs, measuring 50–250 nm in diameter and 5–10 μm in length, formed through the alkaline oxidation of CFs. These served as templates for the uniform electrodeposition of Ni(OH)_2_ NSs. Following nitridation at 400 °C, the resultant Cu_3_N@Ni_3_N NRAs maintained their morphology, characterized by NRs composed of densely packed NPs, depicted in Figure 19b. TEM and HR‐TEM analyses confirmed the presence of a 20 nm‐thick Ni(OH)_2_ shell surrounding 250 nm Cu core rods, with observable mesopores and crystal planes corresponding to Cu_3_N (100) and Ni_3_N (111). Surface defects and voids functioned as electrochemically active sites, thereby enhancing the reactivity of the material. STEM‐EDS mapping demonstrated a uniform distribution of Cu, Ni, and N elements across the NRAs, as shown in Figure 19c. This research introduces a cost‐effective approach to creating high‐performance electrode materials feasible for next‐generation energy storage applications.

Zhu's group^[^ ^207^ ^]^ fabricated 3D hierarchical Ni_3_N/Ni_0.2_Mo_0.8_N heterojunction microspheres (MS) by assembling uniform NRAs on a NF substrate, resulting in the structure denoted as Ni_3_N/Ni_0.2_Mo_0.8_N/NF (Figure 19d). The metallic characteristics of the prepared structure, along with the synergistic interactions between the nitrides, contributed to its remarkable trifunctional catalytic activity for HER, OER, and UOR. Noteworthy performance was observed, with the catalyst achieving current densities of 10 and 200 mA cm^−2^ at relatively low cell voltages of 1.47 and 1.702 V during OWS. Furthermore, when OER was replaced with UOR at the anode, the corresponding cell voltages were further reduced to 1.348 and 1.514 V for the same current densities. The hierarchical Ni_3_N/Ni_0.2_Mo_0.8_N/NF electrode was prepared through a simple hydrothermal approach followed by nitridation. Initially, NiMoO/NF MS, characterized by uniformly distributed NRs, were cultivated on the 3D NF substrate. These were subsequently annealed to yield NiMoO_4_/NF while maintaining their NR‐type morphology. Upon nitridation, the precursors transitioned into Ni_3_N/Ni_0.2_Mo_0.8_N/NF, preserving the crucial hierarchical architecture (Figure 19e). XRD and HR‐TEM analyses validated the successful formation of Ni_3_N and Ni_0.2_Mo_0.8_N heterojunctions, which exhibited well‐defined interfaces that enhance catalytic performance through synergistic effects (Figure 19f). EDS and STEM elemental mapping further confirmed the uniform distribution of Ni, Mo, N, and O within the NRs.

Kim and collaborators^[^ ^208^ ^]^ developed an advanced composite featuring brush‐type Co oxynitride (CoO_*x*_@CoN_*y*_) NRs embedded within N‐C NFs (NCNF). This CoO_*x*_@CoN_*y*_/NCNF composite exhibited remarkable bifunctional catalytic activity for both the ORR and OER in alkaline conditions. The enhancement in performance can be ascribed to the synergistic interactions between the CoO_*x*_ sheath and CoN_*y*_ core, which promote efficient charge transfer and result in the creation of abundant active sites. Furthermore, the distinct growth of CoO_*x*_@CoN_*y*_ on the NCNF substrate provides optimal adsorption sites for ORR intermediates, thereby facilitating effective charge transport. The system also demonstrates excellent durability, which is due to its isolated and regenerable active sites that mitigate catalytic cross‐talk and sustain long‐term performance in applications such as fuel cells, electrolyzers, and zinc‐air batteries (ZABs). As illustrated in Figure 19g, the fabrication begins with the electrospinning and subsequent carbonization of PAN to create NCNF. A PDA coating is then applied to enhance surface hydrophilicity. Following this, Co(OH)F NRs are vertically grown onto the NCNF through hydrothermal synthesis involving Co(NO_3_)_2_.6H_2_O, urea, and NH_4_F. Thermal annealing at varying nitridation temperatures (450, 550, and 650 °C) in an NH_3_ atmosphere yields the CoO_*x*_@CoN_*y*_/NCNF composites. TGA confirmed that NRs constitute 79.5 wt% of the composite. Characterization through SEM and TEM revealed that the NCNF exhibits a uniform 1D fibrous morphology with a diameter of 500 nm (Figure 19h,i). Posthydrothermal treatment, dense Co(OH)F NRs were observed growing perpendicularly on the NCNF. Following nitridation, brush‐like CoO_*x*_@CoN_*y*_ NRs with diameters of about 50 nm and lengths ranging from 2 to 5 μm were formed, displaying strong adherence to the NCNF substrate. However, higher nitridation temperatures (650 °C) resulted in grain coarsening, which could decrease SSA and P_*v*_. In summary, this study emphasizes the importance of surface engineering and structural optimization, including heteroatomic doping and 3D hierarchical designs, in enhancing catalytic performance, as similarly demonstrated in advancements such as MoS_2_/Ni_3_S_2_, MoS_2_/Fe_5_Ni_4_S_8_, Ni_3_N/Ni_0.2_Mo_0.8_N, and Cu_3_N@Ni_3_N systems.

A comparative analysis elucidates the unique advantages presented by 0D, 1D, 2D, and 3D TMNs across various technological applications. 0D TMNs are noted for their remarkable reactivity stemming from their high SSA; however, they are prone to particle aggregation during preparation, which detrimentally impacts their stability. In contrast, 1D TMNs, characterized by their elongated structures, facilitate superior electron transport and exhibit enhanced mechanical robustness, making them well‐suited for applications in flexible electronics and energy storage systems. 2D TMNs offer enhanced conductivity and tunable catalytic activity, thereby proving effective in energy conversion and storage applications, although challenges in preparation persist. Notably, 3D TMNs are recognized for their hierarchical architectures that promote efficient ion diffusion, increased accessibility to electrolytes, and improved charge transfer, a combination of attributes that is crucial for high‐capacity electrodes. As a result, 3D TMNs demonstrate exceptional versatility and electrochemical performance, positioning them as optimal candidates for advanced energy storage technologies, including LIBs and LSBs.

## 2 Types of TMNs

TMNs can be prepared using a variety of physical methods, including plasma processing,^[^ ^209^ ^]^ CVD,^[^ ^210^ ^]^ and laser techniques.^[^ ^211^ ^]^ However, these methods often produce a limited range of products characterized by relatively simple morphologies, such as thin films, and typically necessitate strict operational conditions.^[^ ^212^ ^]^ In contrast, chemical preparation approaches, such as the nitridation of TMOs,^[^ ^27^ ^]^ the conversion of LDHs,^[^ ^213^ ^]^ and the reaction of inorganic salts at elevated temperatures (300‐2000 °C) within N_2_/NH_3_ atmospheres,^[^ ^214^ ^]^ provide enhanced versatility. Notably, thermal decomposition of NH_3_ occurs at milder temperatures (300–800 °C) when contrasted with pyrolysis processes, which operate above 1200 °C.^[^ ^215^ ^]^ Additional methodologies, such as solvothermal techniques and the thermal decomposition of polymeric precursors, also rely heavily on the use of N‐rich reagents. The properties of TMNs, shaped by their metallic composition, architecture, surface features, and heterogeneity, enable customized enhancements in catalytic performance via strategic modifications.^[^ ^216^ ^]^

### 2.1 Monometallic TMNs

**Figure 20:**
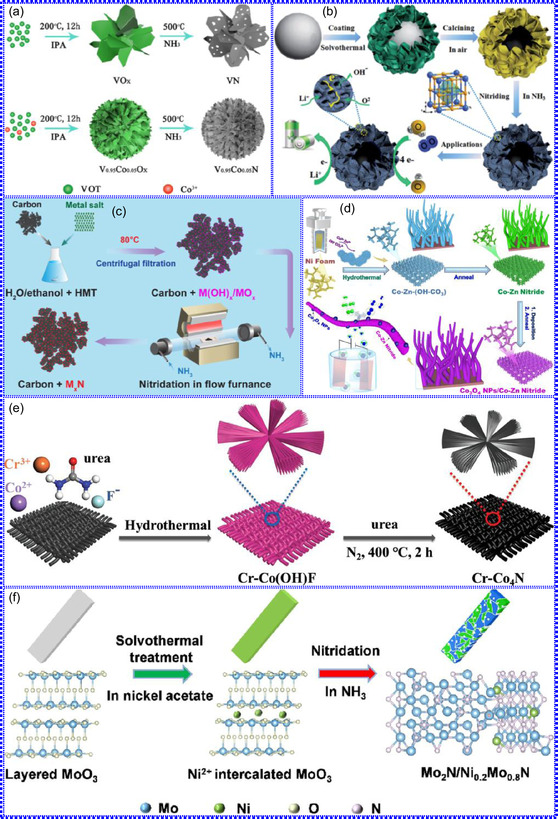
Preparation mechanism of a) V_0.95_Co_0.05_N MFs. Adapted with permission.^[^ ^220^ ^]^ Copyright 2018, American Chemical Society. b) VN HSs. Adapted with permission.^[^ ^221^ ^]^ Copyright 2016, Royal Society of Chemistry. c) C‐based TMN catalysts. Adapted with permission.^[^ ^45^ ^]^ Copyright 2022, Science Advances. d) Co_3_O_4_ NPs/Co‐Zn nitride. Adapted with permission.^[^ ^225^ ^]^ Copyright 2023, Royal Society of Chemistry. e) Cr‐doped Co_4_N NRs on CC. Adapted with permission.^[^ ^226^ ^]^ Copyright 2019, Wiley‐VCH. f) Mo_2_N/Ni_0.2_Mo_0.8_N NBs. Adapted with permission.^[^ ^227^ ^]^ Copyright 2023, Elsevier B.V.

Monometallic TMNs, consisting of a single TM and N, typically crystallize into structures such as HCP, FCC, or simple HEX geometries. Research on electrocatalysts based on monometallic TMNs largely concentrates on the manipulation of their morphology and atomic coordination.^[^ ^217^ ^]^ Luo and colleagues^[^ ^218^ ^]^ found that early TMNs (ETMNs), including ScN, CrN, VN, and TiN, exhibit limited activity toward the ORR, which can be attributed to a lack of d‐electrons and suboptimal surface structures. The catalytic efficiency of these materials is further hindered by difficulties in accurately controlling NPs size and morphology. To improve performance, significant efforts have been made to develop nanomaterials with larger SSAs, such as NFs and NSs architectures.^[^ ^219^ ^]^ For instance, Liao's group^[^ ^220^ ^]^ produced 3D porous, Co‐doped VN microflowers (MF) through straightforward solvothermal preparation followed by nitridation at 500 °C in an NH_3_ atmosphere (**Figure** **20**a). The incorporation of Co and the distinct 3D porous architecture markedly enhance ORR efficiency by increasing the density of active sites and enriching the d‐electron population in V. XRD studies indicated that the Co‐doped V_0.95_Co_0.05_N MF preserved a structure akin to pristine VN, with peak shifts confirming successful Co incorporation. XRD patterns remained uniform across different nitridation temperatures, with higher temperatures yielding larger crystallite sizes. Complete conversion to nitride occurred at 500 °C, with no detectable CoN or metallic Co phases. Remarkably, the Co‐doped samples exhibited smaller diffraction peaks, indicative of a thinner, flower‐type morphology. TEM and SEM images reveal that the VO_*x*_ precursor MF initially possess smooth surfaces and average dimensions of 810 nm. Following nitridation, the MF retains their shape but become progressively more porous, demonstrating excellent structural stability. Notably, the Co‐doped VN MF displayed remarkable durability, with a current decay of less than 12% over 25 000 s, positioning them as promising candidates for fuel cell applications.

Cao and colleagues^[^ ^221^ ^]^ described a template‐assisted approach for the preparation of VN hollow spheres (HSs) that are constructed from porous NTs. These VN HSs are characterized by a hierarchical macro‐/mesoporous architecture, which includes macropores with diameters ≈250 nm, mesopores measuring between 5 nm and 30 nm, a substantial SSA of 66 m^2^ g^−1^, and a uniformly mesoporous shell with a thickness of around 55 nm (Figure 20b). The rigid templates employed in this methodology are C spheres, precisely sized at 400 nm in diameter. These spheres are coated with amorphous VO_*x*_ NSs using a solvothermal process, in which VO(acac)_2_ serves as the V precursor, leading to the formation of core‐shell structured C@VO_*x*_ spheres. Following calcination in an O_2_‐rich atmosphere, the C cores are eliminated, facilitating the transformation of the amorphous VO_*x*_ shells into crystalline V_2_O_5_, thereby yielding V_2_O_5_ HSs. XRD studies confirm the complete phase transition from V_2_O_5_ to pure cubic VN, without the emergence of any alternative VN_*x*_ phases. The resultant VN crystallizes in an FCC structure, exhibiting an average crystallite size of 10.1 nm. This hierarchical architecture enhances mass transport, resulting in exceptional Li‐storage capabilities and elevated catalytic activity for the ORR. The findings of this study underscore a promising strategy for the design of hierarchical porous nitrides with improved performance in LIBs and various energy conversion applications.

Zeng's team^[^ ^45^ ^]^ conducted a comprehensive investigation into a series of C‐supported TMN catalysts (M_*x*_N/C, where M represents Ti, V, Cr, Mn, Fe, Co, and Ni) for the ORR under alkaline conditions (Figure 20c). The crystalline phases of the nitrides were ascertained through XRD and TEM, while XPS and STEM‐EELS analyses elucidated a distinctive nitride‐core/oxide‐shell configuration. Among the studied catalysts, Co_3_N/C, MnN/C, and Fe_3_N/C demonstrated remarkable ORR activity, with Co_3_N/C achieving a peak power density of 700 mW cm^−2^ in tests involving H_2_‐O_2_ fuel cells. XAS studies indicated that Co_3_N/C exhibits structural stability at potential lower than 1 V but experiences considerable degradation at potentials surpassing 1.2 V and thus accentuating the necessity for a passivation layer to improve stability during OER conditions. These findings are pivotal for the advancement of durable TMN‐based electrocatalysts aimed at sustainable energy technologies. In conclusion, despite the promising electrocatalytic characteristics of monometallic TMNs, their performance is frequently limited by insufficient d‐electron density and suboptimal surface morphologies. However, recent developments, including the engineering of 3D porous architectures and HSs, have significantly enhanced their electrochemical properties. Notable instances include Co‐doped VN MF and VN HSs, both of which augment SSA and active site accessibility, thereby improving catalytic activity. Although some stability issues persist, these advancements highlight the potential of mono‐metallic TMNs for future sustainable energy applications.

### 2.2 Bimetallic TMNs

Monometallic TMNs face challenges in achieving catalytic efficiencies that rival their Pt‐based counterparts.^[^ ^222^ ^]^ In response to this limitation, researchers have investigated the preparation of bi‐metallic TMNs, resulting from the combination of two distinct TMs with N. The principal aim of this strategy is to create heterojunction nanostructures via surface coupling and TM atom doping. This doping methodology modifies the d‐electron configuration and electronic structure of the nitrides, thereby enhancing their electrochemical properties and catalytic performance.^[^ ^223^ ^]^ For instance, He and his team^[^ ^224^ ^]^ successfully prepared Co‐ and N*‐co*‐doped VN (VCoN) nanoplates, which exhibited markedly improved electrocatalytic activity for both the OER and HER, attributed to an increased SSA and heightened active site density compared to pure VN. In a similar vein, Luo and colleagues^[^ ^218^ ^]^ demonstrated that doping VN with 3d TMs effectively increased the d‐electron density, thus enhancing the performance of ORR, with Co‐doped VN showing the most significant improvement. Furthermore, Tang and his collaborators^[^ ^220^ ^]^ developed 3D VCoN MF that exhibited superior transport of electroactive species and efficient use of catalytic sites. These collective findings underscore that strategically enhancing d‐electron concentration through doping can significantly elevate the catalytic performance of TMN‐based materials.

Nguyen and collaborators^[^ ^225^ ^]^ presented a novel nanostructured material, consisting of a binary composite of Co‐Zn nitride NRs doped with ultrafine Co_3_O_4_ NPs that are supported on a NF, referred to as Co_3_O_4_ NPs/Co‐Zn nitride (Figure 20d). This composite demonstrates exceptional electrocatalytic activity for both the HER and OER under alkaline conditions. The catalyst attains a current density of 10 mA cm^−2^ at minimal overpotentials of 80.5 mV for the HER and 271.7 mV for the OER. SEM study indicates that post‐hydrothermal preparation, a secondary material uniformly coats the NF substrate, resulting in the formation of robust NRAs with an average diameter of 70 nm. XRD results affirm the high crystallinity of the Co‐Zn‐(OH‐CO_3_) precursor, which maintains its NR morphology after nitridation into the Co‐Zn nitride, albeit with a noticeably roughened surface. This surface roughness and the interconnected nature of NRs contribute to enhanced catalytic performance. TEM further corroborates a consistent NR morphology (≈60 nm) along with textured surfaces, with Co_3_O_4_ NPs constituting 14.08% of the composite structure. The inclusion of these NPs increases the SSA and introduces additional active sites. Electrochemical assessments reveal low Tafel slopes of 54.4 and 52.4 mV dec^−1^, indicating favorable kinetics for the reactions. Furthermore, the catalyst achieves current densities of 10 and 50 mA cm^−2^ at low cell voltages of 1.59 and 1.81 V, respectively, and exhibits remarkable stability over a 60 h period without significant performance degradation.

Luo's team^[^ ^226^ ^]^ prepared porous bi‐metallic Cr‐doped Co_4_N NRs on CC and conducted a systematic evaluation of their electrocatalytic behavior for the HER in alkaline environments (Figure 20e). The Co_4_N NRs, doped with 10% Cr, exhibited exceptional catalytic activity, achieving low overpotentials of 21 and 99 mV at current densities of 10 and 100 mA cm^−2^, respectively. Furthermore, these NRs demonstrated impressive long‐term stability. Theoretical calculations suggest that enhanced HER performance can be attributed to improved adsorption and dissociation of H_2_O molecules, in addition to optimized H_2_ adsorption free energy (ΔG_H*_) resulting from the incorporation of Cr. The research also investigated the effects of doping Co_4_N with other TMs, including Mo, Mn, and Fe, providing a broader understanding of the mechanistic insights into HER catalysis and informing the design of advanced electrocatalytic materials.

Zheng and collaborators^[^ ^227^ ^]^ developed a dual‐phase heterostructure catalyst, Mo_2_N/Ni_0.2_Mo_0.8_N nanobelts (NBs), through the nitridation of Ni‐intercalated MoO_3_ NBs (Figure 20f). By meticulously adjusting the Ni content, they successfully incorporated Ni^2+^ ions into the Mo_2_N matrix without inducing phase separation or structural degradation, thus creating multiple Mo_2_N/Ni_0.2_Mo_0.8_N interfaces. This design facilitated strong electronic interactions, which enhanced both the stability and HER behavior of the catalyst. Remarkably, the Mo_2_N/Ni_0.2_Mo_0.8_N catalyst surpassed commercial Pt/C catalysts under alkaline and simulated seawater conditions, achieving low overpotentials of 26 and 127 mV at current densities of 10 and 100 mA cm^−2^, respectively, alongside a Tafel slope of 31 mV dec^−1^. DFT calculations revealed that the altered H_2_ adsorption and desorption properties at the surfaces of the heterostructure significantly contributed to its superior HER activity. This study introduces an innovative approach to designing highly efficient bi‐metallic nitride‐based catalysts for water electrolysis. In conclusion, while monometallic TMNs often underperform compared to Pt‐based catalysts, recent advancements in bi‐metallic TMNs featuring heterojunction structures and TM doping illustrate a marked improvement in catalytic behavior. Notable examples include Co‐doped VN, Co‐Zn nitride NRs, Cr‐doped Co_4_N, and dual‐phase Mo_2_N/Ni_0.2_Mo_0.8_N catalysts, all demonstrating high efficiency and stability, indicating promising developments for sustainable energy applications.

### 2.3 Trimetallic TMNs

Trimetallic TMNs consist of three distinct metallic elements integrated into a singular structure. These materials have garnered significant attention in the field of catalysis owing to their capability to synergistically harness the unique characteristics of each constituent metal, thereby improving electrochemical behavior and catalytic efficiency.^[^ ^228^ ^]^ The incorporation of multiple metal species allows for modifications to the electronic structure, d‐band center, and overall catalyst stability. Moreover, the inclusion of three metals provides enhanced flexibility in the optimization of essential parameters such as SSA, density of active sites, and conductivity, which collectively contribute to superior catalytic behavior in reactions like the HER and ORR. In comparison to their mono‐ or bimetallic counterparts, trimetallic TMNs exhibit significantly enhanced performance by capitalizing on the combined advantages of diverse metals. These characteristics render trimetallic TMNs particularly appealing for demanding applications that necessitate robust catalytic activity, prolonged operational stability, and high durability, such as fuel cells, OWS, and other electrochemical energy systems.^[^ ^229^ ^]^

**Figure 21:**
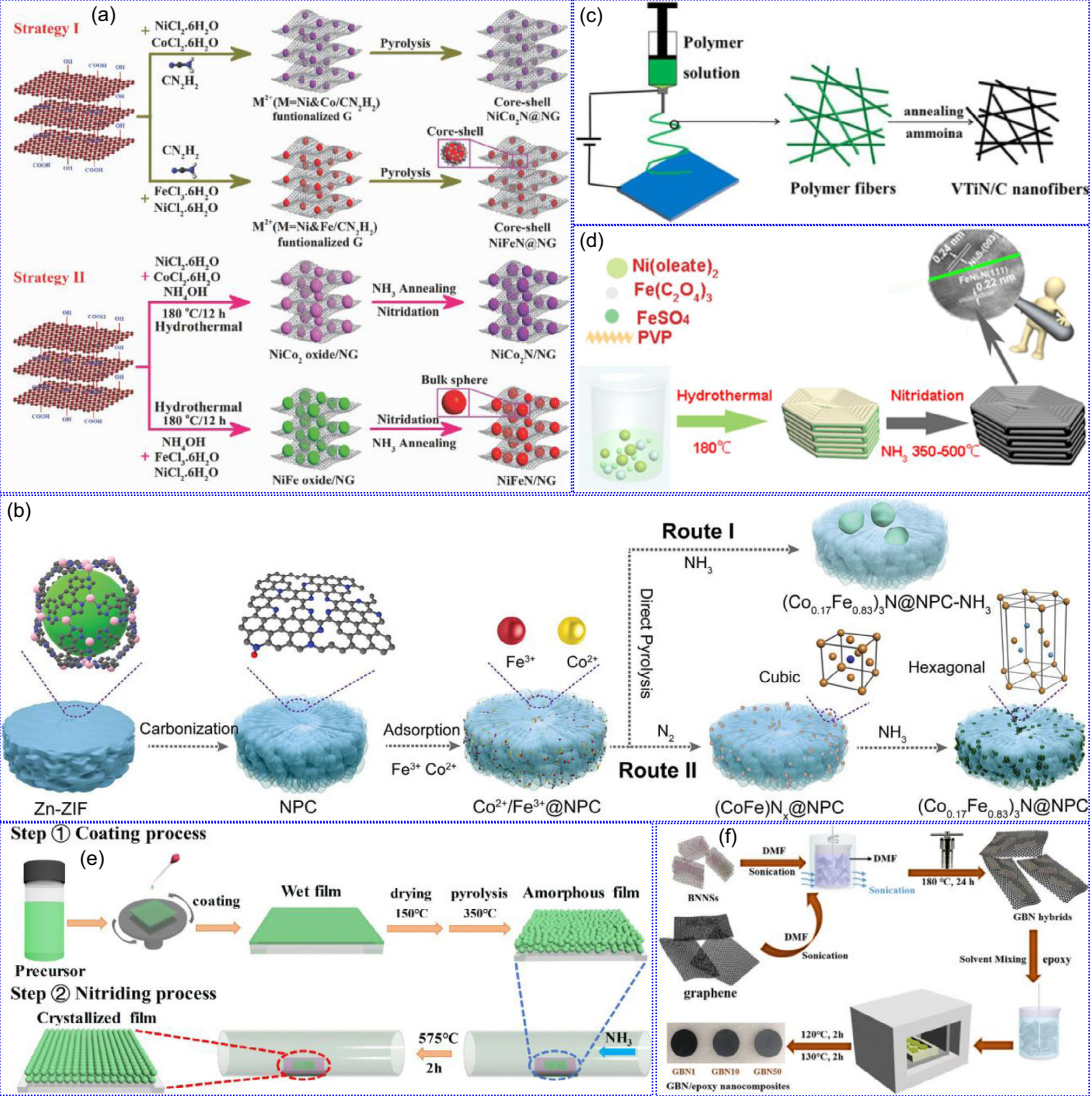
Preparation strategies of a) NiCo_2_N@NG and NiFeN@NG composites. Adapted with permission.^[^ ^230^ ^]^ Copyright 2018, Wiley‐VCH. b) (Co_*x*_Fe_1‐*x*_)_3_N@NPC. Adapted with permission.^[^ ^231^ ^]^ Copyright 2023, Wiley‐VCH. c) VTiN/C NFs. Adapted with permission.^[^ ^232^ ^]^ Copyright 2015, Wiley‐VCH. d) FeNi_3_N‐Ni_3_S_2_. Adapted with permission.^[^ ^233^ ^]^ Copyright 2023, American Chemical Society. e) Ag_*x*_Ni_1‐*x*_NNi_3_. Adapted with permission.^[^ ^234^ ^]^ Copyright 2022, Elsevier B.V. f) GBN NPs and epoxy composites. Adapted with permission.^[^ ^235^ ^]^ Copyright 2020, Elsevier B.V.

Lee's group^[^ ^230^ ^]^ has devised an innovative approach for preparing N‐doped graphene (NG) encapsulated tri‐metallic TMN composites, targeted at high‐performance electrodes for diverse solid‐state ASCs (**Figure** **21**a). The core‐shell NiCo_2_N@NG and NiFeN@NG composites were prepared via a straightforward pyrolysis utilizing GO, metal precursors, and cyanamide at 800 °C. These composites demonstrated markedly improved electron and ion transport properties, resulting in enhancements in C_sp_, rate capability, and cycling stability. An adaptable ASC device was assembled employing NiCo_2_N@NG as the cathode and NiFeN@NG as the anode, exhibiting exceptional energy and power performance in a flexible, portable setup. SEM image revealed a homogenous distribution of NiCo_2_N NPs (10–12 nm) encapsulated within NG NSs, which collectively formed a robust core‐shell architecture and interconnected 3D porous networks. The pyrolysis process effectively facilitated the incorporation of N into both the NiCo_2_ alloy and the graphene framework, thereby enhancing catalytic activity, conductivity, and material stability. In contrast, a traditional two‐step preparation yielded larger (≈40–50 nm) and morphologically inconsistent NiCo_2_N NPs that lacked core‐shell characteristics, leading to reduced electrochemical stability. The practical applicability of these ASCs was underscored by their capability to power a bright red LED, highlighting their potential for advanced flexible energy storage solutions.

Hu's team^[^ ^231^ ^]^ has devised a novel “intermediate phase” strategy for preparing (Co_*x*_Fe_1‐*x*_)_3_N@NPC that are integrated within a porous C network and additionally coated with C. The transition from cubic to HEX phases facilitated the development of a more refined core‐shell architecture. The preparation of (Co_*x*_Fe_1–*x*_)_3_N@NPC involved a series of sequential processes including carbonization, adsorption, and nitridation. Initially, a cake‐shaped Zn‐ZIF precursor was transformed into NPC, characterized by a nanoscale architecture and the complete elimination of metal residues, such as Zn (Figure 21b). This multilayer NPC matrix provides voids and unsaturated atomic sites, thereby enhancing the capture of metal ions. While direct formation of TMN in an NH_3_ atmosphere frequently results in larger particles with reduced exposure of active sites, the intermediate phase methodology presents an alternative pathway. By incorporating NPs within the C framework, this method generates a more sophisticated and intricate structure. The intermediary cubic Fe_4_N phase, alloyed with Co, played a crucial role in the eventual formation of (Co_0.17_Fe_0.83_)_3_N. Raman spectroscopy confirmed the presence of a GC shell, which significantly improved catalytic behaviors. The prepared (Co_0.17_Fe_0.83_)_3_N@NPC demonstrated exceptional bifunctional activity for the ORR and OER. XAS studies and DFT calculations indicated robust electronic interactions between Fe and Co, which facilitated the adsorption of O_2_ intermediates. Remarkably, this catalyst surpassed the performance of commercial Pt/C and IrO_2_ benchmarks in aqueous solutions and ZABs. These findings suggest a promising approach and provide theoretical insights into the design of high‐performance nitride‐based electrocatalysts.

Zhang and collaborators^[^ ^232^ ^]^ developed VTiN/C NFs as advanced electrode materials for SCs via a facile electrospinning approach. This method enabled the homogeneous dispersion of VTiN NPs within a conductive C matrix, ensuring strong interfacial connectivity (Figure 21c). The resulting architecture facilitated efficient electron/ion transport and provided a high SSA for electrochemical interactions. The optimized VTiN‐4/C NFs exhibited a remarkable C_sp_ of 430.7 F g^−1^ at 0.5 A g^−1^, coupled with outstanding rate capability. TEM revealed smooth, uniform NFs with an average diameter of ≈350 nm. The VTiN NPs were evenly distributed within amorphous C, which suppressed grain agglomeration and enhanced ion diffusion. HR‐TEM confirmed well‐defined lattice fringes with a spacing of 0.249 nm, larger than that of pure VN or TiN, demonstrating successful Ti incorporation into the VN crystal lattice. Tuning the V‐to‐Ti atomic ratio enabled modulation of the electrochemical properties, highlighting the promise of VTiN/C NFs for next‐generation SC applications.

Shen's group^[^ ^233^ ^]^ prepared a FeNi_3_N‐Ni_3_S_2_ composite via a facile two‐step process comprising hydrothermal treatment followed by nitridation (Figure 21d). The composite with 12 wt% Ni_3_S_2_ exhibited outstanding OER activity, delivering a low overpotential of 230 mV and a Tafel slope of 38 mV dec^−1^ in 1 M KOH, both superior to those of benchmark IrO_2_ and pristine FeNi_3_N. The remarkable performance was attributed to strong electronic interactions between FeNi_3_N and Ni_3_S_2_ phases. DFT calculations revealed enhanced hydroxyl adsorption and O_2_ generation pathways in the composite. When employed as a cathode in ZABs, the material demonstrated excellent cycling stability, with only a 25 mV increase in charging voltage after 240 cycles. Systematic studies with varying Ni_3_S_2_ content (0–26 wt%) showed that the 12% Ni_3_S_2_ formulation yielded optimal performance, confirming via LSV an overpotential of 230 mV at 10 mA cm^−2^. This formulation also exhibited a superior Tafel slope (38 mV dec^−1^) compared to FeNi_3_N (39 mV dec^−1^) and IrO_2_ (54 mV dec^−1^), emphasizing its promise as a high‐performance OER electrocatalyst.

Sun and collaborators^[^ ^234^ ^]^ investigated AgNNi_3_‐based anti‐perovskite materials as efficient electrocatalysts for the HER in alkaline media (Figure 21e). Structural stability of Ag_*x*_Ni_1–*x*_NNi_3_ was retained for compositions with *x* < 0.8. Among the series, Ag_0.76_Ni_0.24_NNi_3_ exhibited a low onset overpotential (*η*_onset_) of 36 mV and an overpotential of 122 mV at 10 mA cm^−2^ in 1 M KOH. Enhanced HER performance was achieved with 0.18 Ag/Ag_0.8_Ni_0.2_NNi_3_, yielding an exceptional η_onset_ of 13 mV and η_10_ of 81 mV. Microstructural investigations using AFM, FE‐SEM, and TEM revealed a decrease in grain size with increasing Ag content from *x* = 0.57 to 0.8, with Ag‐rich phases localized at grain boundaries. FE‐SEM cross sections confirmed uniform film thickness (≈1 μm) and structural homogeneity. TEM of Ag_0.76_Ni_0.24_NNi_3_ confirmed a polycrystalline morphology with grains of 200–400 nm. HR‐TEM showed a lattice spacing of 2.2 Å corresponding to the (111) plane. In the 0.18 Ag/Ag_0.8_Ni_0.2_NNi_3_ composite, particles at grain boundaries were observed, with HR‐TEM identifying lattice fringes of 2.37 Å (Ag) and 2.22 Å (Ag_0.8_Ni_0.2_NNi_3_). These findings underscore the potential of AgNNi_3_‐based systems as high‐performance HER catalysts under alkaline conditions.

Fu's team^[^ ^235^ ^]^ developed NPs composites by integrating BNNSs with graphene via a multi‐step process involving stirring, sonication, and hydrothermal treatment (Figure 21f). Composites incorporating as little as 5 wt% of GBN_1_ or GBN_10_ exhibited significantly improved thermal conductivity. This enhancement was attributed to the phonon‐conductive properties of BNNSs, which simultaneously suppressed electron migration across graphene layers. Among the variants, GBN_1_ demonstrated superior thermal expansion behavior compared to GBN_10_ and GBN_50_, while also exhibiting lower electrical resistivity due to its minimal BNNS loading. In contrast, GBN_10_ composites balanced enhanced thermal conductivity with effective electrical insulation. Additionally, all composites showed robust thermal stability, elevated decomposition temperatures, and favorable dielectric profiles, characterized by increased dielectric constants and reduced dielectric losses. Collectively, these findings suggest GBN_10_/epoxy composites are ideal for thermal management in electronic systems. In a broader context, this study reinforces that tri‐metallic TMNs improve catalytic efficiency through synergistic effects among three metal constituents, enhancing SSA, active site density, and electrical conductivity. Recent innovations, including N‐C composites, Co‐rich Fe_4_N, FeNi_3_N‐Ni_3_S_2_, and Ag_*x*_Ni_1–*x*_NNi_3_, represent significant strides in energy storage and conversion. These materials exhibit remarkable stability and activity, positioning tri‐metallic TMNs as promising candidates for next‐generation energy technologies.

A comparative assessment of mono‐, bi‐, and trimetallic TMNs reveals notable differences in electrocatalytic activity. Monometallic TMNs, though inherently active, suffer from limitations such as suboptimal surface morphology and a paucity of d‐electrons, which hinder their catalytic efficiency. Advances in morphology, such as the development of 3D porous structures and VN HSs, have partially mitigated these issues, yet their performance still falls short of that achieved by Pt‐based catalysts. In contrast, bi‐metallic TMNs leverage synergistic effects via heterojunction formation and TM atom doping, leading to marked improvements in both catalytic activity and operational stability. Trimetallic TMNs further elevate these attributes by integrating three distinct TM elements, which boosts active site density, SSA, and overall conductivity. This ternary composition enables precise tuning of electrochemical properties, establishing tri‐metallic TMNs as the most versatile and high‐performing candidates for sustainable energy technologies.

## 3 Preparation

TMNs can be synthesized via a broad array of techniques, each conferring unique advantages in terms of structural and functional optimization. Ammonolysis and CVD are frequently employed for fabricating high‐purity films with controlled thickness and stoichiometry. Electrodeposition and electrospinning are effective in producing coatings and NFs, while exfoliation techniques enable the disassembly of bulk precursors into NSs, significantly enhancing the SSA. Hydrothermal and solvothermal methods offer fine morphological control under elevated temperature and pressure. Meanwhile, magnetron sputtering and pyrolysis support the formation of thin films and NPs with tailored features. Microwave‐assisted preparation ensures rapid and uniform heating, often resulting in novel material phases. Sol–gel routes facilitate a controlled transition from liquid to solid, making them suitable for synthesizing customized nitrides. Additionally, template‐assisted methods enable the fabrication of well‐defined nanostructures. Together, these diverse synthetic strategies underpin the versatility and high‐performance capabilities of TMNs across various applications. Detailed discussions of each approach are provided in the subsequent sections.

### 3.1 Ammonolysis

Exsolution has emerged as a highly effective approach for anchoring metal nanostructures within solid‐state matrices. This process typically involves the thermal reduction of multi‐metallic oxides under a reducing atmosphere,^[^ ^236^ ^]^ promoting the migration of metal ions to the surface where they form zero‐valent metal NPs that are partially embedded within the oxide framework.^[^ ^237^ ^]^ These exsolved NPs exhibit robust interfacial adhesion, contributing to enhanced electrocatalytic activity and structural integrity. However, the inherently low electrical conductivity of oxide supports can limit the activation of these immobilized NPs, necessitating conductivity enhancement for optimal performance.^[^ ^238^ ^]^ One viable strategy involves ammonolysis, which enables the stabilization of noble metal NPs within conductive TMN matrices derived from multimetallic oxides incorporating noble elements.^[^ ^239^ ^]^ For instance, the nitridation of Ti_1–*x*_Ru_*x*_O_2_ NWs yields 1D, defect‐rich TiN NTs.^[^ ^240^ ^]^ Subsequent NH_3_ heat treatment induces the exsolution of Ru NPs into the porous TiN NTs, thereby improving both electron and mass transport, ultimately boosting catalytic activity. Despite these advancements, the fundamental mechanisms underlying metal NP exsolution via ammonolysis in TMN systems remain poorly understood and warrant further investigation.^[^ ^240^ ^]^

**Figure 22:**
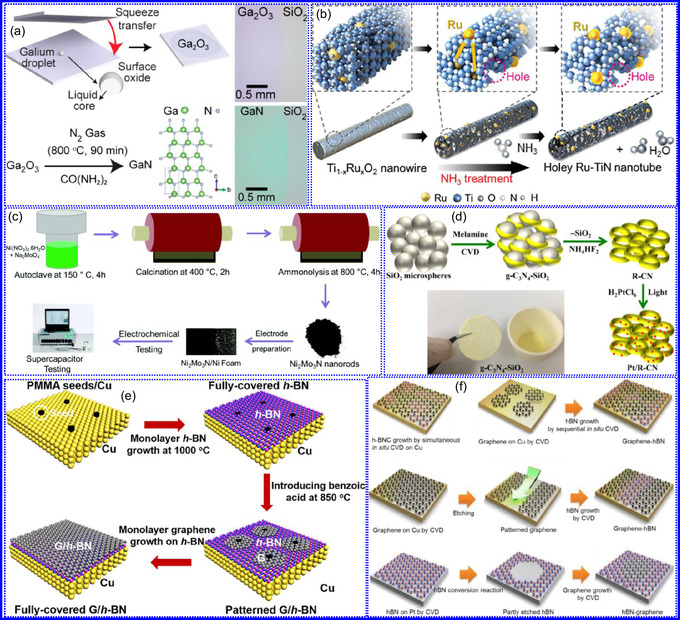
Pictorial representation of the ammonolysis of a) GaN and InN NSs. Adapted with permission.^[^ ^241^ ^]^ Copyright 2023, American Chemical Society. b) Ru‐TiN NTs. Adapted with permission.^[^ ^242^ ^]^ Copyright 2024, Wiley‐VCH. c) Ni_2_Mo_3_N NRs. Adapted with permission.^[^ ^243^ ^]^ Copyright 2020, Royal Society of Chemistry. Pictorial representation of the CVD of d) R‐CN and Pt/R‐CN. Adapted with permission.^[^ ^250^ ^]^ Copyright 2018, American Chemical Society. e) G/h‐BN stacks on Cu foils. Adapted with permission.^[^ ^251^ ^]^ Copyright 2016, American Chemical Society. f) In‐plane graphene and h‐BN heterostructures. Adapted with permission.^[^ ^252^ ^]^ Copyright 2016, Elsevier B.V.

Wang's group^[^ ^241^ ^]^ prepared Cm‐scale ultrathin GaN and InN NSs via the ammonolysis of liquid metal‐derived 2D oxide precursors (**Figure** **22**a). Upon deposition onto tailored substrates, these NSs exhibited high crystallinity with preferential orientation along the (001) plane. The GaN NSs, with an average thickness of ≈1.3 nm, displayed an optical BG of 3.5 eV. Notably, GaN prepared at 800 °C achieved a carrier mobility of 21.5 cm^2^ V^−1^ s^−1^ at room temperature, comparable to that of CVD‐grown MoS_2_. Extending the methodology to InN involved bromination of In_2_O_3_ to yield InBr_3_, followed by ammonolysis at ≈630 °C using urea as the N source. The resulting InN NSs measured ≈2 nm in thickness. HR‐TEM analysis of GaN NSs, formed by depositing Ga_2_O_3_ on Si_3_N_4_ TEM grids followed by ammonolysis, confirmed the formation of ultrathin, transparent sheets. FFT and atomic‐resolution imaging further demonstrated crystalline ordering consistent with the wurtzite GaN structure, with expansion along the (001) direction, as indicated by visible (002) lattice planes. This method provides a scalable and adaptable strategy for integrating 2D III‐nitride semiconductors into next‐generation electronics and optoelectronics.

Hwang and colleagues^[^ ^242^ ^]^ proposed a robust nitridation‐based strategy for the development of high‐performance electrocatalysts by embedding metal NPs within TMN frameworks. This process involved converting 1D Ti_1–*x*_Ru_*x*_O_2_ NWs into Ru‐exsolved, holey TiN NTs via ammonolysis (Figure 22b). The transformation simultaneously induced Ru NPs exsolution and N vacancy formation, both of which synergistically enhanced HER behavior. The intimate interaction and efficient charge transfer between the Ru NPs and the TiN NT matrix facilitated improved H_2_ adsorption/desorption kinetics. Compared to traditional Ru‐deposited structures, the exsolved architecture demonstrated superior HER activity and long‐term electrochemical stability. The methodology offers a scalable route for engineering TMN composites and may be extended to the synthesis of advanced metal phosphide and chalcogenide‐based materials for use in energy storage and sensing platforms.

Sharma's team^[^ ^243^ ^]^ prepared Ni_2_Mo_3_N NRs using a stepwise method that included hydrothermal synthesis, calcination, and subsequent ammonolysis (Figure 22c). XRD and XPS analyses verified the formation of the Ni_2_Mo_3_N phase, with NRs measuring ≈1 μm in length and 70 nm in width. SEM images revealed interconnected NR networks, while EDS confirmed the elemental composition (Ni, Mo, and N). TEM analysis supported these observations, identifying rod‐type structures ≈60 nm wide. HR‐TEM revealed lattice fringes with a 0.221 nm spacing, consistent with the (221) plane, and SAED patterns indicated a polycrystalline structure. The XRD profile confirmed an orthorhombic (orth‐) crystalline phase, along with minor peaks corresponding to Ni_0.2_Mo_0.8_N and metallic Ni. Electrochemical characterization showed a C_sp_ of 264 C g^−1^ at 0.5 A g^−1^, with 41% capacity retention at 5 A g^−1^ and 81.4% stability after 1000 cycles. ASCs utilizing a Ni_2_Mo_3_N//AC configuration delivered 157 C g^−1^ at 1 A g^−1^ with 95.7% retention. These devices also demonstrated high energy and power densities. Overall, ammonolysis proves to be a critical route for TMNs, enabling effective integration of metallic NPs, enhancing defect formation, and facilitating superior electrochemical performance.

### 3.2 CVD

Van der Waals heterostructures composed of graphene, h‐BN, and TMNs represent a pivotal class of materials in modern condensed matter physics and nanotechnology. These layered assemblies facilitate the engineering of electronic characteristics in 2D systems, thereby enhancing the operational efficiency of field‐effect transistors.^[^ ^244^ ^]^ Specifically, heterostructures formed by directly stacking graphene on h‐BN (G/h‐BN) exhibit superior charge carrier mobility, a consequence of the atomically smooth, chemically inert nature of h‐BN, which reduces phonon and impurity scattering. Moreover, the deliberate alignment of graphene relative to h‐BN yields moiré superlattices, enabling the investigation of emergent quantum phenomena.^[^ ^245^ ^]^ G/h‐BN heterostructures are commonly prepared via mechanical exfoliation,^[^ ^246^ ^]^ CVD,^[^ ^247^ ^]^ and direct epitaxial growth on h‐BN substrates.^[^ ^248^ ^]^ While exfoliation methods often result in irregular layer stacking, CVD provides improved cleanliness but may induce grain boundaries in the graphene layer, adversely affecting device performance.^[^ ^249^ ^]^

Cui and collaborators^[^ ^250^ ^]^ synthesized onion‐ring‐shaped g‐C_3_N_4_ microstructures through a straightforward in‐air CVD method. Samples denoted R‐CN‐200, R‐CN‐350, and R‐CN‐500 were produced using SiO_2_ MS templates of 200, 350, and 500 nm diameter, respectively. As control materials, Mix‐CN was obtained without employing CVD, and bulk g‐C_3_N_4_ (B‐CN) served as a reference (Figure 22d). XRD indicated a diminished intensity of the (002) reflection in R‐CN‐350, implying a significant impact of template size on crystallinity. SEM revealed uniform onion‐ring structures in R‐CN‐350, while R‐CN‐200, and R‐CN‐500 exhibited irregular and disrupted morphologies, respectively. TEM validated the structural robustness of R‐CN‐350 under mechanical and chemical stressors. The templated deposition of g‐C_3_N_4_ onto SiO_2_ MS followed by template removal yielded non‐aggregated, finely structured architectures with enhanced photocatalytic activity. Notably, the onion‐ring morphology reduced the material's BG to 2.58 eV (vs. 2.7 eV for B‐CN), promoting superior charge separation, prolonged carrier lifetimes, and a fivefold increase in HER efficiency.

Song's team^[^ ^251^ ^]^ established a seed‐mediated CVD technique for the controlled preparation of single‐crystalline G/h‐BN heterostructures. Polymethyl methacrylate (PMMA) patterns were precisely defined on Cu substrates using electron beam lithography (EBL), functioning as site‐specific nucleation centers during the CVD process. By modulating the inter‐seed spacing, the team effectively regulated nucleation density and domain size, thereby enhancing uniformity in domain morphology and crystallinity. The growth was performed via a two‐step low‐pressure CVD (Figure 22e), employing PMMA seed arrays (≈1 μm diameter, ≈160 nm height) at intervals of 5, 10, 15, and 20 μm. These seeds facilitated h‐BN monolayer formation and subsequently guided the ordered nucleation and expansion of graphene domains. The result was a highly organized heterostructure with the potential for large‐area, high‐quality integration into next‐generation electronic devices.

Wu and colleagues^[^ ^252^ ^]^ offer a thorough review of recent advancements in the CVD preparation of G/h‐BN heterostructures, focusing on both lateral (in‐plane) and vertical configurations. The near‐identical lattice parameters and crystal symmetries of graphene and h‐BN enable seamless in‐plane heterostructure formation, typically achieved via in situ CVD or hybrid in situ/ex situ techniques. Vertically stacked architectures, prepared through multistep CVD, benefit from clean interface formation, free from conversion‐related contamination, thereby improving both device efficiency and environmental responsiveness. Characterization via FFT confirms HEX symmetry and the spatial arrangement of C, B, and N atoms (Figure 22f). XPS identifies bonding interactions including BN, C‐C, C‐N, and C‐B at heterointerfaces, while UV‐vis spectra distinguish compositional domain variations impacting electronic characteristics. Overall, this section emphasizes the centrality of CVD in fabricating high‐performance TMNs and G/h‐BN systems, despite potential drawbacks such as grain boundary formation in graphene. The technique's ability to yield clean surfaces, controlled morphologies, and novel microstructures, such as onion‐ring‐shaped g‐C_3_N_4_, cements its value in emerging photocatalytic and electronic applications.

### 3.3 Electrodeposition

Electrodeposition (ED) serves as a critical technique in the synthesis of TMNs, offering precise regulation over key parameters such as morphology, film thickness, and microstructural properties.^[^ ^253^ ^]^ This level of control facilitates the customization of materials to meet specific functional requirements. Additionally, ED is recognized for its scalability and cost‐efficiency, with broad adaptability across diverse geometries and application environments.^[^ ^254^ ^]^ Its low‐temperature operational profile minimizes thermal degradation risks to substrates, and the prevalent use of aqueous electrolytes enhances environmental compatibility. Collectively, these attributes underscore the potential of ED as a viable and sustainable strategy for large‐scale TMN production in both commercial and technological contexts.^[^ ^255^ ^]^

**Figure 23:**
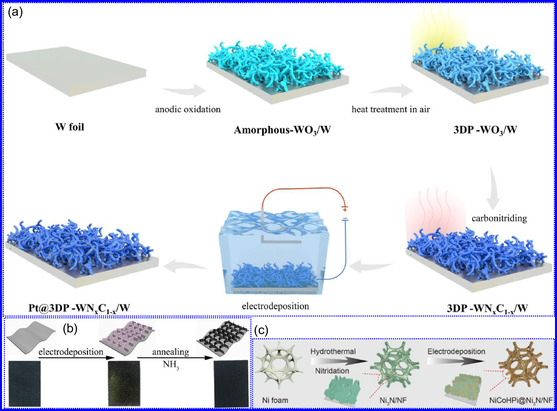
Pictorial demonstration of ED process of a) Pt@3DP‐WN_*x*_C_1‐*x*_/W. Adapted with permission.^[^ ^256^ ^]^ Copyright 2024, Elsevier B.V. b) 3D FeNi‐N/CFC. Adapted with permission.^[^ ^257^ ^]^ Copyright 2017, Wiley‐VCH. c) NiCoHPi@Ni_3_N/NF. Adapted with permission.^[^ ^258^ ^]^ Copyright 2022, American Chemical Society.

Li and colleagues^[^ ^256^ ^]^ engineered a binder‐free, 3D self‐supporting electrode, Pt@3DP‐WN_*x*_C_1–*x*_/W, comprising carbo‐nitride tungsten integrated with Pt NPs for efficient HER in acidic media. The preparation involved a multistep protocol (**Figure** **23**a). A W plate was preprocessed and subjected to anodic oxidation, forming a 3D nanoporous WO_3_ architecture (3DP‐WO_3_/W), which was thermally stabilized at 450 °C. Carbonitridation using melamine at 850 °C in an Ar atmosphere yielded a conductive 3DP‐WN_*x*_C_1–*x*_/W substrate. Temperature‐dependent phase evolution resulted in the formation of WN, WC, or mixed WN_*x*_C_1–*x*_ phases, with complete conversion to HEX WC at 1000 °C. Subsequent Pt deposition generated the final catalyst. XRD confirmed phase transitions, while SEM showed preservation of a coral‐like morphology with high SSA. Uniform Pt dispersion and N‐Pt bonding facilitated enhanced charge transport and H‐atom adsorption/desorption. The electrode achieved an overpotential of 219 mV at −300 mA cm^−2^, surpassing commercial 40% Pt/C in activity and mass performance, with a low Tafel slope and stable operation over 10 000 cycles. These results demonstrate the catalytic advantages of carbo‐nitridation for high‐performance electrode design.

Chen et al.^[^ ^257^ ^]^ fabricated a 3D FeNi‐N/CFC electrode utilizing a facile ED strategy followed by in‐situ nitriding. This method serves as an efficient alternative to hydrothermal, solvothermal, or CVD‐based catalyst deposition on conductive substrates. In this study, 0.25 mg of bi‐metallic FeNi‐N NSs were uniformly deposited onto a CC within 500 s, facilitating systematic investigation into the impact of doping density on HER and OER performance. By modulating the ED time, film thickness and doping density could be finely tuned. Optimal electrocatalytic activity for both HER and OER was observed at a duration of 500 s, with performance declining significantly outside this time window. Specifically, FeNi‐N/CC achieved a current density of 20 mA cm^−2^ at 232 mV for OER and 10 mA cm^−2^ at 106 mV for HER in 1 M KOH. As a bifunctional catalyst for OWS, it required only 1.55 V to reach 10 mA cm^−2^. Furthermore, the alkaline electrolyzer demonstrated stable performance at 360 mA cm^−2^ for over 60 h, underscoring the catalyst's practical potential for scalable H_2_ production (Figure 23b).

Sun's team^[^ ^258^ ^]^ engineered a sandwich‐like electrocatalyst, NiCoHPi@Ni_3_N/NF, through the integration of vertically aligned Ni_3_N NSs clusters grown on NF with a subsequent electrodeposited layer of bimetallic NiCoHPi (Figure 23c). The preparation began with the hydrothermal formation and nitridation of Ni_3_N NSs on NF, followed by a one‐step ED process to deposit the NiCoHPi coating. XRD confirmed the successful formation of both Ni_3_N and NiCoHPi phases. Morphological examination revealed a highly porous, interconnected NPs network, wherein the NiCoHPi layer maintained the structural integrity of the underlying NSs while increasing NP density and coating thickness. The nearly complete coverage of Ni_3_N surfaces with NiCoHPi significantly enhanced the electrochemically active interface. This hierarchical architecture afforded a high SSA and abundant active sites, while synergistic interactions between Ni_3_N and NiCoHPi promoted efficient electron transport. Additionally, the presence of (HPO_4_)^2‐^ anions facilitated hydroxide adsorption via Lewis acid‐base interactions. The catalyst exhibited outstanding bifunctional performance in alkaline simulated seawater, requiring overpotentials of 174 mV for HER and 365 mV for OER to achieve 100 mA cm^−2^. When applied as both electrodes in a full‐cell configuration, it delivered 100 mA cm^−2^ at 1.86 V, demonstrating its viability for seawater electrolysis. Overall, this work underscores ED's critical role in TMN preparation, providing structural precision, low thermal requirements, and environmentally sustainable processing for next‐generation energy applications.

### 3.4 ES

Electrospinning (ES) has emerged as a viable approach for producing high thermal‐conductivity composites, leveraging high‐voltage fields to generate well‐aligned micro‐ and NFs films.^[^ ^259^ ^]^ Despite its potential, the presence of internal air voids poses a significant limitation, as these voids hinder uniform filler dispersion and disrupt the formation of continuous thermal conduction pathways.^[^ ^260^ ^]^ To mitigate these drawbacks, techniques such as hot‐pressing and polymer blending have been explored to enhance inter‐fiber cohesion. However, such methods often introduce additional complexity to the fabrication and reduce control over interfacial thermal resistance.^[^ ^261^ ^]^ An alternative strategy combines ES with electrospraying, a synergistic technique that enables the deposition of composite NPs, improving filler connectivity and facilitating more efficient thermal transport.^[^ ^262^ ^]^

**Figure 24:**
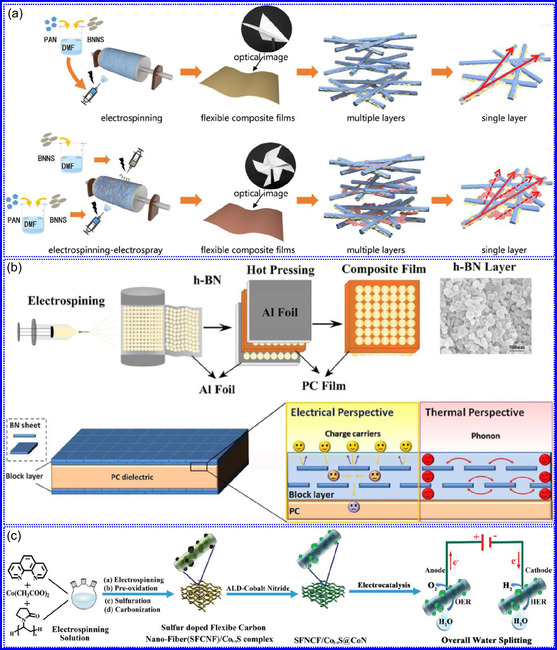
Diagram illustrating the electrospinning activity of a) BNNS/PAN. Adapted with permission.^[^ ^263^ ^]^ Copyright 2022, Elsevier B.V. b) h‐BN/PC/h‐BN. Adapted with permission.^[^ ^264^ ^]^ Copyright 2020, Elsevier B.V. c) SFCNF/Co_1‐*x*_S@CoN composite fibers. Adapted with permission.^[^ ^265^ ^]^ Copyright 2020, Wiley‐VCH.

Yao and collaborators^[^ ^263^ ^]^ introduced a novel fabrication method integrating ES with electrospraying to synthesize composite films exhibiting superior thermal conductivity. In this dual‐process approach, ES established the primary thermal conduction pathways, while electrospraying interconnected the electrospun fibers, thereby augmenting the thermal network. PAN was employed as the polymer matrix, and BNNSs, obtained via ball milling, served as thermally conductive fillers. SEM and AFM characterization revealed BNNSs with lateral dimensions ranging from 100 nm to 200 nm and thicknesses near 10 nm. XRD confirmed the structural integrity of h‐BN post‐processing, and FTIR spectra indicated the emergence of functional groups, notably NH_2_, which promote enhanced interaction and H‐bonding with PAN. TGA validated that the BNNSs content within the films accurately reflected intended loadings, ensuring precise thermal analysis. Remarkably, the composite exhibited substantial thermal conductivity (24.98 W m^‐1 ^K^−1^) at a 40 wt% BNNSs loading, attributed to the synergistic effect of electrospraying. These films also demonstrated effective thermal management in LED dissipation tests. This study marks the first report of combining ES and electrospraying for high‐performance thermal composites incorporating a variety of fillers and polymer systems (**Figure** **24**a).

Lei's group^[^ ^264^ ^]^ developed a dielectric composite film featuring a sandwich structure (h‐BN/PC/h‐BN) fabricated via a combined ES and hot‐pressing process, which activated the film surface. This method preserved the mechanical integrity of the films while markedly enhancing their thermal conductivity and energy storage capabilities. The outer h‐BN layers improved heat diffusion and limited thermal accumulation, while simultaneously acting as insulating barriers that reduced electrical conductivity and suppressed current density loss, thus minimizing Joule heating. The resulting films exhibited excellent thermal conductivity, an energy retention density of 5.52 J cm^−3^ at 500 MV m^−1^ and 100 °C, and high charging/discharging efficiency (87.2%). These characteristics were stable across a broad temperature range (ambient to 100 °C), confirming suitability for high‐temperature dielectric applications. Cross‐sectional SEM image revealed a uniform polycarbonate (PC) core with a thickness of 10–13 μm, flanked by consistent h‐BN layers measuring 1–5 μm. EDS elemental mapping confirmed homogeneous distribution of B and N, while XRD data showed distinct h‐BN diffraction peaks at 2*θ* = 26.75° and 54.94°, corresponding to the (002) and (004) planes, with no evidence of contamination. These findings demonstrate the potential for scalable, cost‐effective dielectric films capable of reliable performance at elevated temperatures (Figure 24b).

Guo and collaborators^[^ ^265^ ^]^ report the development of a novel Co‐based composite, S‐doped CNF (SFCNF)/Co_1–*x*_S@CoN, engineered to enhance bifunctional electrocatalytic activity in OWS. The preparation strategy integrates ES and ALD, built upon several foundational hypotheses: 1) S doping increases the electrical conductivity within the CNF matrix; 2) S‐Co interactions promote the in‐situ formation of Co_1–*x*_S NPs; 3) CoN is efficiently deposited on either Co_1–*x*_S NPs or CNFs via ALD; and 4) the Co_1–*x*_S‐CoN hybridization induces synergistic effects that boost catalytic activity for both the HER and OER. The fabrication process proceeds in two stages. First, a precursor solution containing PVP, Co(OAc)_2_, and other components is electrospun, then subjected to S doping and annealing at 800 °C, facilitating the conversion of Co‐O to Co_1–*x*_S and generating surface porosity. In the second phase, ALD deposits a uniform CoN layer over the NF framework. SEM, TEM, and EDS analyses reveal a homogeneous fibrous morphology with Co_1–*x*_S NPs uniformly dispersed, serving as nucleation sites for CoN. High‐resolution imaging confirms distinct lattice fringes in both NFs and NPs, and elemental mapping demonstrates uniform S, Co, and C distributions. The observed absence of N is attributed to its complete volatilization during annealing, which aids defect formation in Co_1–*x*_S. This work underscores the potential of ES‐based processes to create TMN composites with high electrocatalytic efficiency, despite challenges in achieving homogeneous filler dispersion. By integrating ES with electrospraying, improved thermal conductivity and inter‐filler connectivity are realized, advancing materials for thermal and electrochemical applications (Figure 24c).

### 3.5 Exfoliation

2D nanomaterials, such as graphene, h‐BN, CN, and TMNs, have attracted considerable interest owing to their outstanding mechanical, electrical, and thermal characteristics.^[^ ^266^ ^]^ These materials are typically synthesized via exfoliation of bulk layered precursors, yielding ultrathin sheets comprising one or several atomic layers. The landmark mechanical exfoliation of graphene from graphite in 2004,^[^ ^267^ ^]^ catalyzed a surge in research on other 2D materials, including h‐BN NSs. While h‐BN NSs are commonly produced through exfoliation, these processes often involve complex, energy‐intensive, and chemically aggressive conditions that may introduce contaminants and necessitate extensive post‐processing. Consequently, an optimal synthesis for *fl*‐h‐BN should offer scalability, environmental safety, and additive‐free operation, while retaining high efficiency and structural integrity.^[^ ^268^ ^]^

**Figure 25:**
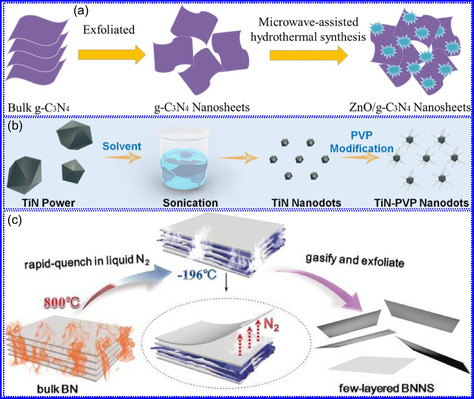
Pictorial demonstration of exfoliation synthesis of a) g‐C_3_N_4_/ZnO NSs. Adapted with permission.^[^ ^269^ ^]^ Copyright 2017, Elsevier B.V. b) TiN NDs. Adapted with permission.^[^ ^270^ ^]^ Copyright 2021, Elsevier B.V. c) BNNS. Adapted with permission.^[^ ^271^ ^]^ Copyright 2016, Wiley‐VCH.

Tian's team^[^ ^269^ ^]^ introduced a novel non‐metallic electrode constructed by integrating g‐C_3_N_4_ and ZnO NSs onto FTO substrates for the electrochemical detection of H_2_O_2_. The g‐C_3_N_4_ NSs, obtained via exfoliation, functioned as a microwave‐assisted hydrothermal reaction medium to facilitate the preparation of ZnO NSs. This hybrid structure enhances both H_2_O_2_ adsorption and electron transfer efficiency. The fabricated electrode exhibited a high sensitivity of 540.8 mA mM^−1^ cm^−2^ at an applied potential of −0.5 V, with a broad linear detection range spanning from 0.05 to 14.15 mM. Structural and compositional analyses using TEM, AFM, and XRD confirmed the uniform deposition of ZnO on g‐C_3_N_4_, the nanoscale thickness (4–5 nm) of the g‐C_3_N_4_ layers, and the retention of phase purity without any detectable impurities. Electrochemical characterization revealed excellent H_2_O_2_ reduction capability, highlighting the electrode's potential for application in biosensing and environmental monitoring (**Figure** **25**a).

Wang's team^[^ ^270^ ^]^ successfully prepared ultra‐small TiN NDs with an average diameter of 1.5 nm via a liquid‐phase exfoliation. These NDs exhibit strong absorption in the NIR‐II region, rendering them effective for both photoacoustic (PA) imaging and photothermal therapy (PTT) in oncological applications. Additionally, the presence of surface defects and partial oxidation to TiO_2_ enables the NDs to function as efficient sonosensitizers, producing ROS under ultrasound (US) stimulation, thereby facilitating sonodynamic therapy (SDT). The modest photothermal effect further enhances tumor oxygenation, amplifying the combined therapeutic outcomes of PTT and SDT. Owing to their sub‐2 nm size, the NDs are rapidly metabolized and excreted, minimizing concerns related to long‐term biotoxicity. TEM confirmed a dot‐like morphology with an average size of 1.54 ± 0.53 nm. XRD revealed a cubic TiN phase, indicating high crystallinity and purity. XPS data showed the presence of Ti and N, along with surface TiO_2_, which contributes to increased hydrophilicity and aqueous dispersibility. However, the NDs exhibited aggregation tendencies in physiological media, prompting the use of PVP surface functionalization to enhance colloidal stability. These findings underscore the potential of TiN NDs as multifunctional theranostic agents in cancer therapy (Figure 25b).

Sheng and colleagues^[^ ^271^ ^]^ developed an innovative exfoliation strategy for producing ultrathin BNNSs, employing a synergistic approach of high‐temperature expansion followed by *l*‐N_2_ quenching. The process involves thermal treatment of industrial‐grade h‐BN at 800 °C to induce lattice expansion, succeeded by rapid immersion in *l*‐N_2_ to facilitate exfoliation into *fl*‐NSs. Multiple thermal cycles yielded BNNSs comprising 1–5 layers with a production yield of 16–20%. SEM image revealed a significant size reduction and morphology transition in BNNS‐10 relative to the pristine bulk h‐BN. TEM confirmed the ultrathin, transparent, and uniform nature of BNNS‐10. XRD maintained the characteristic h‐BN crystalline structure, albeit with a diminished (002) peak and a 2θ shift from 26.82° to 26.70°, indicating increased interlayer spacing from 3.3 to 3.4 Å. Raman spectroscopy showed a G‐band shift from 1365.8 to 1366.8 cm^−1^, attributed to heightened in‐plane stress and reduced interlayer coupling. BET study reported a dramatic increase in SSA from 10 (bulk) to 278 m^2^ g^−1^ (BNNS‐10). This methodology supports the scalable preparation of BNNSs for advanced thermal management applications, including their integration into high‐performance TMN‐based composites via techniques such as electrospraying, hot‐pressing, and ALD.

### 3.6 Hydrothermal

h‐BN, colloquially termed “white graphene,” has garnered considerable interest due to its superior physical characteristics, including outstanding optical transparency, high dielectric strength, notable thermal conductivity, and exceptional chemical inertness.^[^ ^272^ ^]^ Its characteristic layered structure renders it particularly suitable for applications across catalysis, optoelectronic devices, and semiconductor platforms. Concurrently, BNQDs have emerged as appealing nanomaterials because of their low cytotoxicity, excellent carrier mobility, biocompatibility, and facile preparation. While top‐down fabrication techniques have been widely employed to produce BNQDs, bottom‐up synthetic strategies remain relatively underdeveloped, largely due to a scarcity of appropriate precursor materials.^[^ ^273^ ^]^ Although hydrothermal approach using a mixture of H_3_BO_3_ and NH_3_ has been explored, safety concerns stemming from volatility and toxicity have impeded its scalability. Melamine, an abundant and N‐rich compound, offers a potentially safer and more economical alternative for BNQDs production; however, its utility in this context requires further investigation.^[^ ^274^ ^]^

**Figure 26:**
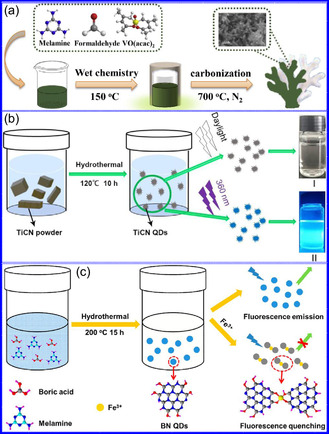
Diagram illustrating the hydrothermal synthesis of a) VN/C electrode. Adapted with permission.^[^ ^275^ ^]^ Copyright 2023, Elsevier B.V. b) TiCN QDs. Adapted with permission.^[^ ^276^ ^]^ Copyright 2019, Elsevier B.V. c) BNQDs. Adapted with permission.^[^ ^277^ ^]^ Copyright 2017, American Chemical Society.

To overcome prevailing challenges in SC electrode development, Wang and colleagues^[^ ^275^ ^]^ synthesized a coralloid‐structured VN/C composite via a facile hydrothermal route. Morphological analyses using SEM and TEM confirmed the formation of a uniform, coral‐like nanostructure characterized by branched interconnections. TEM further revealed VN NPs, ≈5–10 nm in diameter, homogeneously embedded within the C matrix. SAED patterns indicated the polycrystalline nature of VN with a cubic phase. XRD patterns displayed well‐defined VN peaks and significant amorphous C content, the latter contributing to improved electrical conductivity and mechanical integrity. Raman spectroscopy identified both sp^2^‐hybridized graphitic and sp^3^‐defective C, with increasing VN content correlating to heightened disorder. The synergistic interaction between VN and C enhanced electrochemical behavior by supplying conductive pathways and promoting ion mobility. Additionally, C encapsulation protected VN from electrolytic oxidation, thereby improving C_sp_ and cycling life. The VN/C electrode demonstrated a C_sp_ of 385 F g^−1^ at 1 A g^−1^ and maintained 88.9% of its capacitance after 10 000 cycles. A symmetric two‐electrode configuration further exhibited 93.3% retention, affirming the material's viability for high‐performance energy storage (**Figure** **26**a).

Li and colleagues^[^ ^276^ ^]^ successfully prepared TiCN QDs via a facile one‐step hydrothermal approach. The as‐prepared QDs exhibited excellent aqueous dispersibility and robust fluorescence, making them suitable candidates for multifunctional applications. Structural and compositional analyses were conducted using TEM, AFM, UV‐Vis, FTIR, fluorescence spectroscopy, and XPS. TEM revealed monodispersed QDs with an average diameter of 2.7 ± 0.2 nm, while HR‐TEM confirmed a lattice spacing of 0.216 nm, corresponding to the (200) plane of TiC_0.7_N_0.3_. AFM analysis corroborated a lateral size range of 2.7–4 nm, with a mean of 3.2 ± 0.3 nm, indicating a highly exfoliated nanostructure. FTIR spectra identified surface functionalities including —OH, ‐NH, C—C, C—N, C—O, and Ti—O, which enhance solubility and stability in aqueous environments. XPS confirmed the presence of Ti, N, C, and O, with corresponding bonding states such as Ti—C, Ti—N, Ti—O, C=C, C—N, C—O, C=O, and N—H. Notably, the fluorescence of TiCN QDs was quenched in the presence of Fe^3+^ ions, enabling a sensitive and label‐free detection method for Fe^3+^ in tap water. These findings underscore the importance of surface chemistry in determining the optical characteristics and application potential of TiCN QDs (Figure 26b).

Liu's group^[^ ^277^ ^]^ prepared BNQDs via a bottom‐up hydrothermal method by reacting H_3_BO_3_ with melamine at 200 °C for 15 h. The as‐prepared BNQDs displayed strong blue fluorescence under UV irradiation, enabling their evaluation as fluorescent sensors for TM ions. The BNQDs exhibited high selectivity and sensitivity toward Fe^3+^ ions, achieving a detection limit of 0.3 μM. Fluorescent mapping on fibers confirmed their utility as effective and visually appealing fluorophores. TEM image revealed BNQDs with an average diameter of ≈3 nm and distinct lattice fringes, confirming their crystallinity. AFM height profiles (≈0.7 nm) suggested monolayer characteristics. Surface functionalities were characterized via XPS, and their optical features were assessed using UV‐Vis and PL spectroscopy. The BNQDs exhibited a UV‐Vis absorption peak at 205 nm, blue fluorescence at 365 nm excitation, and a PL emission maximum at 400 nm with excitation peaks at 230 and 300 nm. These findings underscore the potential of BNQDs in Fe^3+^ detection and other chemical sensing applications. The results also demonstrate the versatility of hydrothermal synthesis for fabricating TMNs and related nanomaterials with tunable functionalities, including high‐capacitance VN/C electrodes and fluorescent TiCN QDs (Figure 26c).

### 3.7 MS

Magnetron sputtering (MS) is a critical physical vapor deposition (PVD) technique widely employed in the preparation of TMNs, providing precise control over film uniformity, composition, and thickness.^[^ ^278^ ^]^ This approach accommodates the deposition of various TMNs, including complex multilayer and composite architectures tailored for specific performance requirements. Its low operational temperature is advantageous for coating thermally sensitive substrates, minimizing heat‐induced deformation or stress.^[^ ^279^ ^]^ Owing to its scalability, precision, and versatility, MS is particularly well‐suited for industrial‐scale fabrication in electronics, automotive, and aerospace applications. TMNs produced via MS commonly exhibit superior structural integrity, electrical conductivity, tribological performance, and optical characteristics, underlining their relevance for high‐performance technologies.^[^ ^280^ ^]^

**Figure 27:**
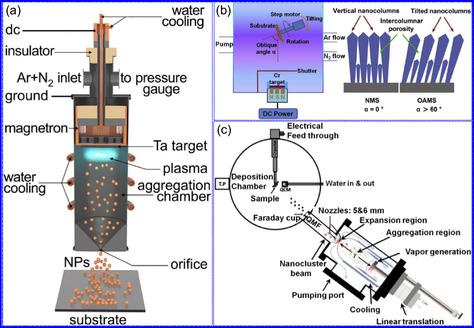
Diagram illustrating the MS synthesis of a) Ta_3_N_*y*_O_*x*_ NPs. Adapted with permission.^[^ ^281^ ^]^ Copyright 2021, Elsevier B.V. b) CrN thin films. Adapted with permission.^[^ ^282^ ^]^ Copyright 2019, Elsevier B.V. c) AlN NPs. Adapted with permission.^[^ ^283^ ^]^ Copyright 2020, MDPI.

Gordeev's team^[^ ^281^ ^]^ developed a reactive MS (RMS) approach in an Ar/N_2_ atmosphere to synthesize Ta_3_N_*y*_O_*x*_ NPs in a single, solvent‐free, and environmentally sustainable step. This method enables precise compositional control by adjusting the flow rates of N_2_ and O_2_. TEM showed a decrease in NP size from 27 ± 3 to 14 ± 3 nm with increasing N_2_ concentration, although excessive N_2_ introduction led to deposition rate declines due to target surface passivation by nonconductive species, an effect confirmed by variations in magnetron bias and optical emission spectra. Sputtering in pure Ar yielded metallic Ta NPs with a polycrystalline core and ≈2 nm amorphous oxide shell, as confirmed via SAED and XRD. Natural postsynthesis oxidation further modified surface chemistry. This RMS process, operating at room temperature under high‐pressure gas‐phase agglomeration, marks an advancement from single‐metal and alloy NP synthesis to ternary oxynitride systems. The method shows promise for producing tailored TM oxynitride NPs for PEC OWS and related applications (**Figure** **27**a).

Qi and collaborators^[^ ^282^ ^]^ conducted an in‐depth investigation into the fabrication of nanostructured porous CrN thin films via oblique angled MS (OAMS) for application in symmetric SCs. CrN was deposited directly onto Si wafers, serving a dual role as the current collector and active material. The study systematically assessed the impact of magnetron configuration and deposition pressure on both structural and electrochemical characteristics, elucidating structure‐property correlations. XRD study showed a dominant diffraction peak at 43°, corresponding to the (200) plane of FCC CrN, indicative of phase‐pure films. Among the three samples, the 3‐O variant, fabricated under high deposition pressure and oblique incidence, exhibited the highest density of structural defects. This was corroborated by Raman spectroscopy, which revealed two broad phonon‐related bands, most intense in the 3‐O sample, associated with lattice disorder. XPS spectra confirmed the presence of Cr‐N bonding along with adsorbed N species. The optimized film structure enabled symmetric SC devices to achieve a C_sp_ of 17.7 mF cm^−2^ at 1 mA cm^−2^, along with an energy density of 7.4 mWh cm^−3^ and a power density of 1.8 W cm^−3^, all while maintaining excellent cycling stability (Figure 27b).

Alawadhi's group^[^ ^283^ ^]^ employed MS in conjunction with inert gas condensation within an ultra‐high vacuum (UHV) system to synthesize AlN NPs. AFM, operated in noncontact dynamic mode using a piezoelectric, structurally refined tip, and quadrupole mass filter (QMF) study confirmed an average particle size of 3 nm. Raman spectroscopy affirmed the wurtzite phase of the AlN NPs, with Lorentzian fits revealing distinct vibrational modes: A_1_(TO) at 619 cm^−1^, E_1_(TO) at 670 cm^−1^, E_2_(high) at 653 cm^−1^, and E_1_(LO) and A_1_(LO) at 826 and 905 cm^−1^, respectively. Minor frequency shifts and peak broadening were ascribed to size effects and structural fragility. The optical BG was estimated at 5.1 eV, underscoring the NPs suitability for nanoelectronic device integration (Figure 27c). In summary, MS remains a critical technique for the synthesis of TMNs, offering fine‐tuned control over film composition, structure, and deposition temperature. Its applicability across a spectrum of TMN‐based systems, from tunable Ta_3_N_*y*_O_*x*_ NPs to high‐performance CrN films and optoelectronic‐grade AlN nanostructures, demonstrates its versatility in next‐generation material platforms.

### 3.8 Microwave

Microwave‐assisted synthesis has become a prominent strategy for the fabrication of TMNs, offering advantages such as rapid, uniform volumetric heating that promotes controlled nanostructure formation.^[^ ^284^ ^]^ This technique accelerates reaction kinetics, significantly reducing synthesis durations while simultaneously enhancing yield and process efficiency. Its inherently high energy efficiency minimizes thermal dissipation, making it well‐suited for scalable, industrial applications.^[^ ^285^ ^]^ Furthermore, microwave irradiation allows for precise modulation of particle size, morphology, and crystallinity, key parameters in optimizing TMNs for targeted functionalities.^[^ ^286^ ^]^ The method is also environmentally benign and adaptable across a broad range of material systems, highlighting its pivotal role in the advancement of nanomaterials research and application.^[^ ^286^ ^]^

**Figure 28:**
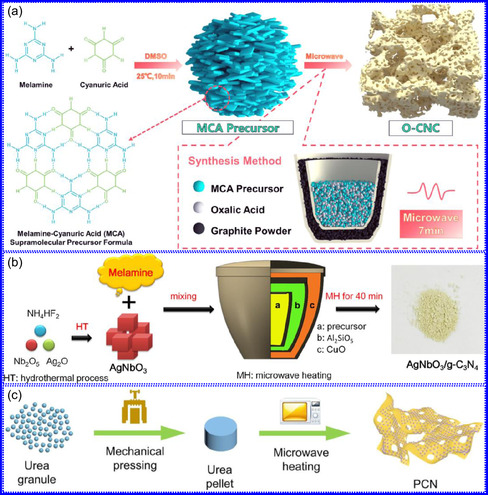
Pictorial demonstration of microwave synthesis of a) O‐CNC from the MCA precursor. Adapted with permission.^[^ ^287^ ^]^ Copyright 2021, American Chemical Society. b) AgNbO_3_/g‐C_3_N_4_‐M composite. Adapted with permission.^[^ ^288^ ^]^ Copyright 2019, Elsevier B.V. c) PCN. Adapted with permission.^[^ ^289^ ^]^ Copyright 2023, Elsevier B.V.

Huang and colleagues^[^ ^287^ ^]^ introduced an efficient method for synthesizing O‐doped CN‐containing C voids (O‐CNC) via a rapid 7‐min microwave annealing of MCA supramolecular aggregates, using oxalic acid as a doping and structuring agent (**Figure** **28**a). The resultant O‐CNC materials displayed a mesoporous architecture with tunable BGs, enhanced charge carrier separation, and superior two‐electron oxygen reduction capabilities conducive to H_2_O_2_ generation. SEM and TEM studies confirmed a retained hollow spherical morphology (3–4 μm) composed of NSs in the precursor CN, while the introduction of oxalic acid led to fragmentation into smaller (≈1 μm) hollow clusters comprised of ≈20 nm thick NSs. This transformation is attributed to gas evolution from oxalic acid decomposition, facilitating the disruption and reassembly of MCA‐derived structures at elevated temperatures. Increased oxalic acid concentrations resulted in further NSs thinning. Elemental mapping revealed homogeneous distributions of C, N, and O. XRD showed diminished (002) peak intensity and a slight peak shift, consistent with reduced layer thickness and interlayer distances. A previously unobserved peak at 29.3–29.4°, attributed to the (003) plane of triazine‐based g‐C_3_N_4_ (R3m space group), was identified in both CN and O–CNC samples. FTIR spectra confirmed structural and functional group integrity, with characteristic triazine and C–N heterocyclic bands, along with a peak at 1238 cm^−1^ confirming C–O–C bonds indicative of O‐doping. Enhanced crystallinity was evidenced by stronger diffraction and vibrational features. Collectively, this approach offers a rapid and tunable route for producing high‐performance CN‐based photocatalysts for H_2_O_2_ production.

He's group^[^ ^288^ ^]^ significantly improved the photocatalytic HER efficiency of g‐C_3_N_4_ by integrating AgNbO_3_ NCs via microwave‐assisted synthesis (Figure 28b). The 40‐min microwave treatment enabled direct growth of g‐C_3_N_4_ on AgNbO_3_ surfaces, fostering strong interfacial contact between the semiconductors. Structural characterization using XRD, FTIR, and XPS confirmed the successful formation of the AgNbO_3_/g‐C_3_N_4_ heterojunction. XRD data revealed distinct g‐C_3_N_4_ peaks at 13.1° and 27.5°, with the latter exhibiting a slight shift in microwave‐treated samples (g‐C_3_N_4_‐M), indicative of enhanced interlayer spacing and improved charge mobility. AgNbO_3_ maintained its characteristic cubic phase, although its peak intensity decreased with lower loadings (e.g., 0.5%). FTIR spectra displayed typical g‐C_3_N_4_ vibrational bands, while AgNbO_3_ signals were diminished due to its low content. TEM and HR‐TEM images confirmed the coexistence of AgNbO_3_ particles and thin g‐C_3_N_4_ layers, with lattice fringes further validating heterojunction formation. Despite minimal changes to SSA and light absorption, the composite exhibited enhanced charge separation as evidenced by reduced PL intensity and extended photocarrier lifetimes. These synergistic effects culminated in improved visible‐light‐driven H_2_ evolution, positioning the AgNbO_3_/g‐C_3_N_4_ system as a promising candidate for solar fuel applications.

Yuan and colleagues^[^ ^289^ ^]^ introduced a novel approach for enhancing photocatalytic H_2_ production under visible light via the microwave thermolysis of urea pellets to synthesize polymeric CN (PCN) (Figure 28c). This microwave‐assisted process yields two significant advantages: it disrupts the tri‐s‐triazine framework to enable an otherwise forbidden n‐π* electronic transition and improves crystallinity by enhancing the reactivity of N‐rich precursors. Consequently, the resulting PCN exhibits a highly ordered structure with reduced defects, significantly boosting HER activity. XPS and elemental analyses indicated that PCNmp‐30's enhanced crystallinity stems from microwave‐induced decomposition and powder densification, yielding a higher C/N ratio but lower activity than PCNfp. Morphological studies via SEM and TEM revealed that PCNmp‐30 comprises large, curved 2D porous NSs, evidence of structural distortion. While PCNfp had a slightly higher SSA (79.7 vs. 76.3 m^2^ g^−1^), both materials originate from the same precursor. Impressively, PCNmp‐30 exhibited a HER rate of 61.7 μmol h^−1^, nearly sixfold greater than that of conventionally prepared PCN. This work underscores the effectiveness of microwave synthesis in fabricating high‐performance TMNs, offering rapid, energy‐efficient, and environmentally friendly production with fine control over structural and morphological characteristics. Applications include mesoporous O‐doped CN structures, AgNbO_3_/g‐C_3_N_4_ composites, and crystalline PCNs for solar fuel technologies.

### 3.9 Pyrolysis

Pyrolysis has emerged as a pivotal technique for the controlled synthesis of TMNs, offering precise regulation over nanostructural parameters such as particle size, morphology, and crystallinity through the high‐temperature decomposition of precursors under inert atmospheres.^[^ ^290^ ^]^ This approach promotes material uniformity and purity by eliminating undesirable byproducts, leading to TMNs of superior structural integrity. Its versatility allows for the transformation of a wide array of precursors into TMNs with diverse chemical compositions and advanced architectures, such as core‐shell and porous nanostructures, which are particularly beneficial in catalytic and energy storage contexts.^[^ ^291^ ^]^ Moreover, pyrolysis is amenable to scalable production and is environmentally favorable due to reduced reliance on solvents and toxic reagents. These characteristics affirm pyrolysis as a highly effective and sustainable approach for fabricating high‐performance TMNs.^[^ ^292^ ^]^

**Figure 29:**
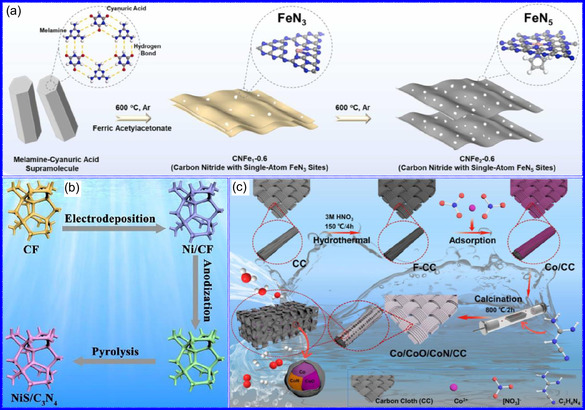
Diagram illustrating the pyrolysis synthesis of a) 2D CN. Adapted with permission.^[^ ^293^ ^]^ Copyright 2022, Elsevier B.V. b) NiS/C_3_N_4_ on CF. Adapted with permission.^[^ ^294^ ^]^ Copyright 2021, Elsevier B.V. c) Co/CoO/CoN/CC. Adapted with permission.^[^ ^295^ ^]^ Copyright 2023, Elsevier B.V.

Liu and colleagues^[^ ^293^ ^]^ developed a pyrolysis‐based method for fabricating porous 2D CN materials featuring well‐defined single‐atom FeN_5_ coordination sites and a notably high Fe loading of 16.64 wt%. The resulting catalyst exhibited excellent performance in PMS activation for the degradation of organic pollutants, including sulfamethoxazole, even at temperatures approaching 0 °C. The research involved a systematic exploration of Fe content and pyrolysis parameters to optimize catalytic efficiency. Structural and electronic characteristics were probed through various characterization techniques and corroborated by theoretical modeling, elucidating the origins of the catalyst's outstanding low‐temperature activity. The CN matrix was prepared via self‐assembly of MCA into supramolecular films, followed by pyrolysis‐induced graphitization and porosity formation. Initial pyrolysis led to the generation of FeN_3_ sites, with internal gas pressure fostering pore development. EDS confirmed uniform Fe dispersion, while XPS analysis of CNFe_2‐0.6_ revealed distinct N functionalities and an Fe 2p_3/2_ peak shift of 3.71 eV after secondary pyrolysis, indicating enhanced Fe electronic interaction. These results underscore the role of coordination tuning and structural design in advancing single‐atom Fenton‐like catalysts for low temperature‐environment applications (**Figure** **29**a).

Ma's team^[^ ^294^ ^]^ developed a facile and controllable route for fabricating a NiS core encapsulated within a C_3_N_4_ shell (NiS/C_3_N_4_) via a combination of electrochemical deposition and pyrolysis. X‐ray characterization confirmed the presence of N‐Ni‐S coordination sites, indicative of strong interfacial coupling between NiS and the C_3_N_4_ shell. The composite exhibited outstanding electrocatalytic activity for the OER, achieving an overpotential of 334 mV at 10 mA cm^−2^ and a Tafel slope of 45 mV dec^−1^, comparable to that of benchmark IrO_2_ catalysts. XRD displayed distinct peaks corresponding to both NiS and C_3_N_4_ phases, confirming their crystallinity. SEM and TEM images revealed a uniform CF substrate densely decorated with NiS/C_3_N_4_ NSs. The brighter TEM edges and darker core regions were attributed to the C_3_N_4_ shell and NiS core, respectively, while HR‐TEM confirmed lattice fringes of both materials. HAADF‐STEM and elemental mapping demonstrated uniform C_3_N_4_ coverage over NiS, with clear phase boundaries, validating successful encapsulation. The catalyst exhibited excellent long‐term OER stability over 50 h under alkaline conditions, a performance attributed to its high density of active sites and improved catalytic kinetics (Figure 29b).

Lang and colleagues^[^ ^295^ ^]^ prepared a Co/CoO/CoN composite integrated into a hierarchically porous NGC framework supported on CC via a pyrolysis strategy. The NGC matrix effectively inhibits metal NPs agglomeration, enabling the composite to serve directly as a self‐supporting electrode. The synergistic effects of the Co/CoO/CoN heterointerfaces facilitate improved electronic conductivity and enhanced charge transfer, thereby significantly boosting catalytic activity. Among the variants, the Co/CoO/CoN/CC‐2 architecture exhibited outstanding electrocatalytic behavior, achieving overpotentials of just 73 mV for the HER and 147 mV for the OER, surpassing numerous reported Co‐based systems. The OWS was accomplished at a low cell voltage of 1.48 V. The OER performance notably exceeded that of benchmark RuO_2_ (335 mV overpotential), and its Tafel slope (36.13 mV dec^−1^) was markedly lower than that of RuO_2_ (56.21 mV dec^−1^), underscoring the catalyst's superior kinetics (Figure 29c). These findings reinforce pyrolysis as an essential method for engineering TMNs with tunable morphology and crystallinity, offering a scalable and environmentally benign route to high‐performance electrocatalysts.

### 3.10 Sol–Gel

Traditional techniques for synthesizing spinel ferrite NPs, including hydrothermal,^[^ ^296^ ^]^ and microwave‐assisted methods^[^ ^297^ ^]^ often entail high costs, complex procedures, and adverse environmental impacts. As a result, there has been increasing interest in environmentally benign alternatives.^[^ ^298^ ^]^ Among these, honey‐assisted sol–gel auto‐combustion has gained attention for its operational simplicity and the multifunctional role of honey as both a fuel and reducing agent.^[^ ^299^ ^]^ Additionally, other natural materials, such as tragacanth gel and various plant extracts, have been employed effectively, enabling sustainable and cost‐efficient synthesis of ferrite NPs with excellent catalytic and photocatalytic characteristics.^[^ ^300^ ^]^

**Figure 30:**
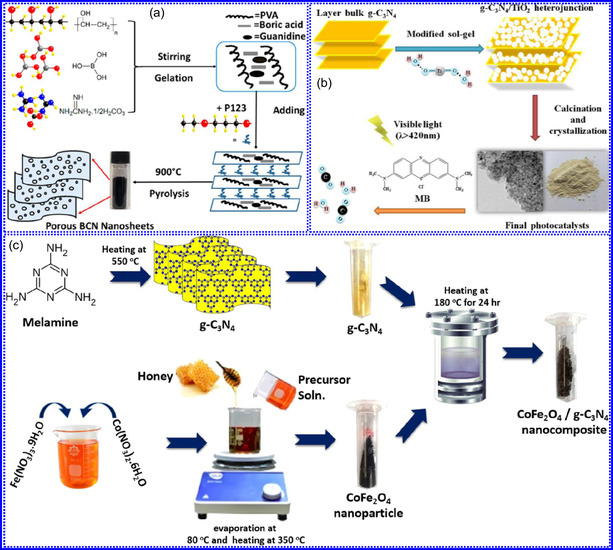
Pictorial demonstration of sol‐gel synthesis of a) BCN NSs. Adapted with permission.^[^ ^301^ ^]^ Copyright 2017, American Chemical Society. b) g‐C_3_N_4_, TiO_2_, and g‐C_3_N_4_/TiO_2_ composites. Adapted with permission.^[^ ^302^ ^]^ Copyright 2017, Elsevier B.V. c) CFO/g‐C_3_N_4_ composite. Adapted with permission.^[^ ^303^ ^]^ Copyright 2019, IOP Publishing.

In a notable advancement, Lei's team^[^ ^301^ ^]^ introduced a facile and cost‐effective sol‐gel approach for synthesizing porous BCN NSs, providing an economical alternative to conventional CVD techniques. The resultant BCN NSs exhibit a high SSA of 817 m^2^ g^−1^, a meso‐/microporous architecture, and excellent ORR catalytic behavior under both alkaline and acidic conditions, rivaling that of Pt/C catalysts. Furthermore, the materials demonstrate superior durability and methanol crossover resistance. The preparation involves cross‐linking PVA, H_3_BO_3_, and guanidine carbonate to form a polymeric gel, with the triblock copolymer (P123) incorporated as a porosity enhancer. Thermal treatment at 900 °C under N_2_ results in the formation of 2D porous BCN structures, facilitated by gas release from the decomposing P123 template. These features position BCN NSs as a highly promising, metal‐free ORR catalyst for fuel cell technologies (**Figure** **30**a).

Zheng and colleagues^[^ ^302^ ^]^ developed g‐C_3_N_4_/TiO_2_ composite photocatalysts with varying g‐C_3_N_4_ weight fractions via a modified sol‐gel technique. Bulk g‐C_3_N_4_, obtained through thermal calcination, served as a substrate for TiO_2_ crystallization, facilitating precise control over surface microstructure and limiting particle dispersion. The study investigated the underlying mechanisms contributing to the enhanced photocatalytic activity of these composites, particularly in the degradation of MB. FTIR spectroscopy revealed characteristic absorption bands: TiO_2_ exhibited signals at 1652 cm^−1^ (O‐H bending of adsorbed water) and 1422 cm^−1^ (Ti—O bond vibrations), while g‐C_3_N_4_ presented prominent bands between 1200 and 1650 cm^−1^ (C—N and C=N stretching), along with a distinctive 808 cm^−1^ peak (s‐triazine ring vibrations). In the composites, a slight red shift (≈2 cm^−1^) was detected in the C—N and C=N regions upon TiO_2_ incorporation, though the spectral profiles were predominantly characteristic of g‐C_3_N_4_ (Figure 30b).

Mangalraj's group^[^ ^303^ ^]^ prepared a g‐C_3_N_4_/CoFe_2_O_4_ (CFO) composite aimed at simultaneous Pb^2+^ ion adsorption and MB photodegradation. Relative to pristine g‐C_3_N_4_, the composite exhibited significantly enhanced photocatalytic and adsorption efficiencies. Notably, the magnetic characteristics imparted by CFO enabled facile recovery using an external magnetic field, underscoring the composite's practical applicability in water purification. XRD confirmed the preserved cubic spinel structure of CFO and revealed distinct diffraction peaks from both g‐C_3_N_4_ and CFO, validating the formation of a two‐phase composite. SEM identified lamellar g‐C_3_N_4_ sheets and spherical CFO NPs, with clear phase contrast. UV‐vis spectroscopy showed that g‐C_3_N_4_ absorbed primarily in the 200–450 nm range, while CFO demonstrated strong absorption in the visible region. The composite exhibited broadened visible light absorption, directly correlating with its superior photocatalytic behavior (Figure 30c). Overall, the sol–gel emerges as a versatile and eco‐friendly strategy for synthesizing TMNs, including spinel ferrites, BCN NSs, and photocatalytic composites with promising environmental remediation potential.

### 3.11 Solvothermal

Solvothermal is a highly versatile and widely adopted strategy for synthesizing TMNs, offering meticulous control over morphology, particle size, and crystallinity through precise regulation of parameters such as temperature, pressure, and reaction duration.^[^ ^304^ ^]^ Enhanced solubility and reactivity of precursors under solvothermal conditions facilitate the formation of uniform, high‐purity nanostructures. This approach accommodates the synthesis of a diverse array of TMNs, encompassing metal alloys and compound materials. Elevated reaction temperatures contribute to improved crystallinity, a key factor for applications in catalysis and electronic devices.^[^ ^305^ ^]^ Furthermore, the technique's inherent adaptability and scalability render it suitable for both laboratory research and industrial‐scale production. Optimization via solvent selection and the use of specific additives enables fine‐tuning of TMN characteristics, enhancing their utility in catalysis, energy storage, and environmental remediation.^[^ ^306^ ^]^

**Figure 31:**
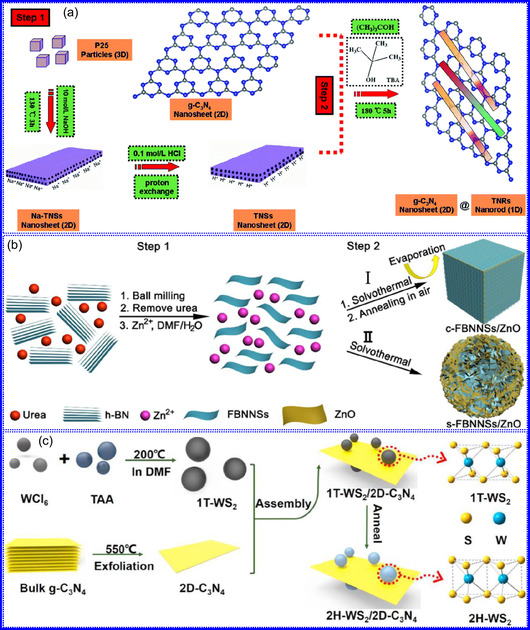
Diagram illustrating the solvothermal synthesis of a) TNR/g‐C_3_N_4_ composites. Adapted with permission.^[^ ^307^ ^]^ Copyright 2017, Royal Society of Chemistry. b) 3D c‐ and s‐FBNNSs/ZnO. Adapted with permission.^[^ ^308^ ^]^ Copyright 2019, American Chemical Society. c) 1T‐WS_2_/2D‐C_3_N_4_ and 2H‐WS_2_/2DC_3_N_4_. Adapted with permission.^[^ ^309^ ^]^ Copyright 2018, Elsevier B.V.

Wang's group^[^ ^307^ ^]^ employed a solvothermal‐assisted method to assemble TiO_2_ NRs onto large g‐C_3_N_4_ NSs. The NRs, measuring ≈200–300 nm in lateral dimension, were uniformly distributed across the g‐C_3_N_4_ surface. The TiO_2_ NRs/g‐C_3_N_4_ composite demonstrated enhanced photocatalytic activity, effectively removing potassium dichromate (Cr(VI)) and degrading rhodamine B (RhB) simultaneously. This improvement is attributed to efficient charge recombination suppression and enhanced RhB adsorption. These composites show strong potential for large‐scale applications in optoelectronics and water purification (**Figure** **31**a).

Lei and colleagues^[^ ^308^ ^]^ introduced a cost‐effective evaporation‐assisted solvothermal technique for synthesizing 3D superstructures comprising *fl*‐BNNSs integrated with ZnO, yielding both spherical and cubic morphologies. The formation of these superstructures was primarily governed by van der Waals and electrostatic interactions, which facilitated enhanced CO_2_ adsorption. The ZnO framework not only directed the morphological development of the composites but also increased the density of accessible adsorption sites. Notably, the spherical (s‐*fl*‐BNNSs/ZnO) structures demonstrated superior CO_2_ capture efficiency compared to their cubic counterparts, attributed to a higher availability of trapping sites. Additionally, the hollow variants of these composites further improved adsorption performance, underscoring the influence of structural design. Although s‐*fl*‐BNNSs/ZnO surpassed *fl*‐BNNSs, h‐BN, and GO in CO_2_ uptake, it remained less efficient than porous AC and MOFs. These results offer valuable insights into the self‐assembly behavior of *fl*‐BNNSs and the underlying mechanisms of gas adsorption (Figure 31b).

Li's group^[^ ^309^ ^]^ reported that metallic 1T‐phase WS_2_, synthesized through a facile solvothermal, functions as an effective co‐catalyst to enhance photocatalytic HER activity. The 1T‐WS_2_ phase is distinguished by its noble‐metal‐free composition, high electrical conductivity, and an abundance of catalytically active sites on its basal plane. The 1T‐WS_2_/2D‐C_3_N_4_ composite exhibited markedly improved HER behavior relative to both pristine 2D‐C_3_N_4_ and its counterpart with semiconducting 2H‐WS_2_. The metallic 1T phase was synthesized using WCl_6_ and thioacetamide (TAA) in DMF, where NH_4_^+^ ions facilitate the disruption of W‐W bonds, thus favoring the 1T structural configuration. Subsequent integration with exfoliated 2D‐C_3_N_4_ produced the high‐performance composite, while thermal treatment led to the formation of the less active 2H phase. These results underscore the significance of surface characteristics in dictating photocatalytic efficiency (Figure 31c). Overall, the solvothermal remains a highly adaptable and effective route for engineering TMNs, offering fine‐tuned control over their size, morphology, and crystallinity. The method enhances precursor reactivity and reproducibility, promoting the formation of high‐quality TMNs suited for applications in catalysis, energy storage, and environmental remediation. Through strategic manipulation of solvents and additives, solvothermal techniques enable the fabrication of functionally optimized nanomaterials, as exemplified by the creation of efficient photocatalysts and CO_2_ capture platforms.

### 3.12 Template Based

TMNs have garnered significant interest in the domains of energy storage and electrocatalysis, owing to their intrinsic properties such as high electrical conductivity, mechanical durability, and exceptional electrocatalytic activity.^[^ ^310^ ^]^ These characteristics facilitate enhanced electrochemical behavior, as demonstrated by VN,^[^ ^311^ ^]^ which has been employed in advanced electrode architectures for LSBs and SCs.^[^ ^312^ ^]^ A key limitation, however, lies in the ability to synthesize TMNs with well‐defined porous structures, which are critical for optimizing ion transport, mass diffusion, and the availability of active sites. While gas‐phase deposition methods yield high‐purity crystalline TMNs, they are hindered by high costs and limited scalability. In contrast, solution‐based approaches offer more accessible synthesis but often at the expense of structural and compositional integrity.^[^ ^313^ ^]^ In response, recent advancements have highlighted the efficacy of sacrificial salt templating as a viable strategy for producing 2D TMN and TMO NSs with tailored porosity and morphology.^[^ ^314^ ^]^

**Figure 32:**
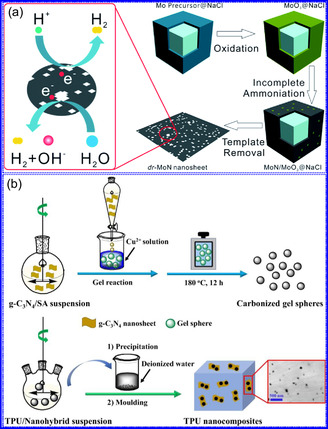
Pictorial demonstration of template synthesis of a) dr‐MoN catalysts. Adapted with permission.^[^ ^315^ ^]^ Copyright 2017, Royal Society of Chemistry. b) SACS, SACS‐C, CSACS‐C, and g‐C_3_N_4_. Adapted with permission.^[^ ^316^ ^]^ Copyright 2019, Elsevier B.V.

In this context, Cheng and collaborators^[^ ^315^ ^]^ introduced a novel salt‐templating strategy to synthesize large‐area, defect‐rich 2D MoN (dr‐MoN) NSs for HER applications. The synthesis involved the deposition of 2D HEX MoO_3_ NSs onto NaCl NPs, followed by partial ammonization, yielding MoN/MoO_3_ composites. Selective etching of MoO_3_ subsequently produced dr‐MoN NSs with abundant edge defects, which serve as active catalytic sites. The dr‐MoN exhibited exceptional HER activity in both acidic and alkaline environments, achieving overpotentials of 125 and 139 mV at a current density of 10 mA cm^−2^, respectively, and demonstrated impressive durability over 2000 electrochemical cycles and 20 h of continuous operation. The catalytic performance was further evaluated across three variants (dr‐MoN‐0, dr‐MoN‐1, and dr‐MoN‐3), synthesized by altering annealing conditions (**Figure** **32**a).

Hu's team^[^ ^316^ ^]^ synthesized a ternary composite consisting of g‐C_3_N_4_ NSs, C spheres, and Cu NPs, denoted as CSACS‐C, which was subsequently incorporated into a thermoplastic polyurethane (TPU) matrix. Comprehensive structural and combustion analyses of the composite were conducted. TEM revealed a well‐defined lamellar morphology (≈1 μm) adorned with Cu NPs. Elemental mapping and EDS confirmed the uniform distribution of C, N, and Cu within the composite, substantiating the successful formation of all intended components. Spectroscopic investigations of pristine TPU at 353 and 447 °C indicated prominent signals for various degradation products, including Ph‐NH_2_, CH_2_, CO_2_, NCO, carbonyl, and C–O–C species. Thermal decomposition occurred via a two‐step mechanism: initial degradation of hard segments into isocyanates, alcohols, olefins, and CO_2_, followed by soft segment breakdown. The TPU‐CSACS‐C composite exhibited an analogous degradation profile; however, an increase in second‐stage CO_2_ evolution suggested that CSACS‐C facilitates earlier decomposition, promoting oxidative degradation. This was further corroborated by lower emissions of volatile organic compounds (VOCs), decreased carbonyl content, and increased char yield (Figure 32b). In summary, template‐assisted fabrication offers a robust, scalable means of producing TMNs with tunable porosity and enhanced functionalities, as demonstrated by the fabrication of dr‐MoN NSs for HER and CSACS‐C composites for improved CO_2_ adsorption and flame retardancy.

A comparative assessment reveals that among various TMN synthesis techniques, template preparation stands out for its ability to precisely control porosity and tailor material properties. This method, often utilizing sacrificial agents such as salts, facilitates the formation of defect‐rich nanostructures with enhanced electrocatalytic performance and versatile functional applications. Its inherent scalability, economic viability, and systematic execution render it a compelling strategy for advanced technologies, including electrocatalysis and composite material development. Although other approaches like ammonolysis, CVD, and ED offer niche advantages for specific catalytic processes, template‐assisted synthesis offers broader adaptability and precision in structural engineering. A comprehensive comparison of these methodologies, delineating their benefits, limitations, and impact on TMN attributes, is summarized in **Table** **1**.

**Table 1:** The evaluation and comparison of different techniques used for the synthesis of TMNs.

S. No.	Preparation technique	Advantages	Disadvantages	References
1	Ammonolysis	Easy, modest temperature	Toxic NH_3_	[243, 255]
2	CVD	High purity	Complex, costly	[24, 215]
3	Electrodeposition	Adaptable, cheap	Thin films	[149, 255]
4	Electrospinning	Nanofiber shape	Processing required	[317, 511]
5	Exfoliation	High surface, 2D	Multistep, layered	[512, 513]
6	Hydrothermal	Nanoscale, low temperature	Complexity, scalability	[132, 137]
7	Magnetron sputtering	High purity	Thin films	[514, 515]
8	Microwave	Efficient, rapid	Uneven heating	[516, 517]
9	Pyrolysis	Simple production	High temperature	[136, 518]
10	Sol‐gel	Uniform particles	Slow processing	[132, 519]
11	Solvothermal	Particle tuning	Solvents, scalability	[309, 520]
12	Template	Customizable morphology	Template removal	[521, 522]
13	Direct nitridation	Simple, scalable	High temperature	[521, 523]

## 4 Engineering

The electronic structure of TMNs plays a critical role in determining their physical, chemical, and catalytic characteristics. Tailoring the structure of TMNs enhances electronic conductivity and overall performance.^[^ ^317^ ^]^ Below are several common strategies for tuning the electronic structure of TMNs.

### 4.1 Heterostructure Engineering

Heterostructures have emerged as a critical design strategy in the development of advanced electrode materials.^[^ ^318^ ^]^ The synergistic integration of TMNs with metals, oxides, and carbides within heterostructured architectures significantly enhances their electronic, physicochemical, and catalytic properties. Among these, transverse heterostructures have been extensively investigated for their superior performance in catalysis and energy storage applications.^[^ ^319^ ^]^ For instance, Cheetham's group^[^ ^320^ ^]^ utilized a bottom‐up route to engineer porous 2D TMN heterostructures with tunable pore architectures, achieving notable electrocatalytic efficiency and long‐term stability in the OER. Further studies on Mo‐N systems have revealed that catalytic behavior in HER is strongly influenced by the N/M ratio, which exhibits a volcano‐type relationship.^[^ ^321^ ^]^ The N/M ratio governs electronic delocalization and conductivity, thereby affecting catalytic activity.^[^ ^322^ ^]^ Specifically, reduced N content in Co_4_N has been correlated with enhanced electrical conductivity and improved OER kinetics. Moreover, engineering additional active sites, such as O‐vacancies, within Co_3_O_4_‐Co_4_N composites have been shown to further amplify electrocatalytic activity. These findings underscore the importance of precisely tuning the N/M ratio and other structural features to optimize the performance of TMN‐based catalysts.^[^ ^323^ ^]^

Yu and colleagues^[^ ^324^ ^]^ introduced an efficient one‐pot hydrothermal synthesis to construct a ternary Ru/Nb_2_O_5_@Nb_2_C heterostructured photocatalyst. In this system, Ru NPs act as active sites for HER, while Nb_2_C MXene sheets facilitate charge carrier separation as co‐catalysts. The hydrothermal simultaneously oxidized Nb_2_C to Nb_2_O_5_ NWs and reduced Ru^3+^ to metallic Ru, yielding a composite with remarkable HER activity and quantum efficiency. This strategy underscores the superior reducibility of MXenes over conventional noble metal photodeposition methods, enabling enhanced surface electron transfer. Due to their thermodynamic instability and O‐affinity, MXenes readily oxidize under oxidants like H_2_O_2_, O_2_, CO_2_, and H_2_O. Under hydrothermal conditions, carbides react with water, forming oxides, releasing CO_2_, and incorporating oxygen. Surfactants such as NaBF_4_ play a key role in modulating surface charges and promoting structural expansion during synthesis. The resulting heterostructure was characterized via XRD, confirming the transformation of Nb_2_AlC into Nb_2_C and the formation of Nb_2_O_5_; Ru peaks were absent, likely due to minimal loading or nanoscale dispersion. SEM images revealed Nb_2_O_5_ NW growth on exfoliated Nb_2_C with increased SSA and exposed active sites. TEM/HR‐TEM confirmed NW morphology (30–50 nm diameter) and a fringe spacing of 0.39 nm, consistent with Nb_2_O_5_ (001) planes. The observed 0.91 nm interlayer spacing verified the structural integration. Overall, this work highlights the significance of heterostructures in enhancing TMN electrochemical performance. Through careful compositional tuning and defect engineering (e.g., N/M ratio and O‐vacancies), catalysts such as Ru/Nb_2_O_5_@Nb_2_C demonstrate exceptional potential in HER and broader photocatalytic applications.

### 4.2 Alloying

This section highlights two essential strategies for enhancing TMNs: heterostructuring and alloying.^[^ ^325^ ^]^ Heterostructures refer to the combination of multiple materials at an interface, while alloying entails the uniform distribution of two or more metallic elements within a single material, such as multimetallic nitrides.^[^ ^326^ ^]^ The alloying approach is particularly advantageous due to its potential to combine the favorable attributes of individual metals, thus optimizing overall catalytic behavior.^[^ ^327^ ^]^ Greene's group^[^ ^328^ ^]^ studied the cubic pseudo‐binary alloy Ti_0.5_W_0.5_N, revealing that elevated valence electron concentration improved metal–metal bonding and facilitated interlayer sliding under mechanical stress. Similarly, Jaramillo and colleagues^[^ ^329^ ^]^ developed a NiN thin film via reactive sputtering, achieving outstanding electrocatalytic activity marked by high activity, selectivity, and stability. Mao's group^[^ ^330^ ^]^ proposed a carbonization–reduction (CAR) route to synthesize a MoNiNC HER electrode. Starting from a 3D polymer framework with molecularly dispersed Mo, the precursor was thermally converted to MoNiNC, exhibiting excellent performance in alkaline HER (0.1 M KOH), with overpotentials of 110 and 150 mV at 10 and 50 mA cm^−2^, respectively, on par with commercial Pt/C/NF. The electrode sustained 100 mA cm^−2^ for 24 h without performance degradation. SEM and elemental mapping revealed porous morphology and uniform NP dispersion. Structural analyses (XRD, TEM, and XPS) confirmed the presence of active species (Mo^2+/3+^, metal‐N bonds). DFT calculations attributed the enhanced activity to reduced binding energies facilitated by co‐doping with N and C and alloying with Ni. Collectively, this work underscores the critical contributions of heterostructure and alloying in developing robust, scalable HER catalysts, while also signaling the importance of further studies into operational stability under dynamic voltage conditions.

### 4.3 Heteroatom Doping

Extensive experimental and computational investigations have firmly established the efficacy of heteroatom doping in enhancing the structural and functional attributes of TMNs.^[^ ^331^ ^]^ This technique alters not only the chemical composition but also the physical, electrochemical, and catalytic behaviors of TMNs, particularly in the context of electrocatalysis and HER.^[^ ^332^ ^]^ The introduction of dopants induces localized lattice strain and atomic distortions, thereby facilitating charge redistribution within the crystal lattice. Doping strategies are typically classified into metal and nonmetal atom incorporation, each contributing either to the activation of inert TMN domains or the formation of novel catalytic sites.^[^ ^333^ ^]^ For instance, Pan's group^[^ ^334^ ^]^ synthesized NSs featuring spinel‐type nanostructures embedded in hollow N‐C polyhedrons, where N doping effectively exposed TM sites at the surface. This configuration exhibited superior HER activity due to the synergistic interplay between enhanced electron mobility and improved charge carrier separation, resulting in a greater density of active adsorption sites. Additionally, heteroatom doping has been shown to modulate the Gibbs free energy (ΔG_H_) of HER, thereby optimizing intrinsic catalytic efficiency in single‐phase TMNs.^[^ ^335^ ^]^

**Figure 33:**
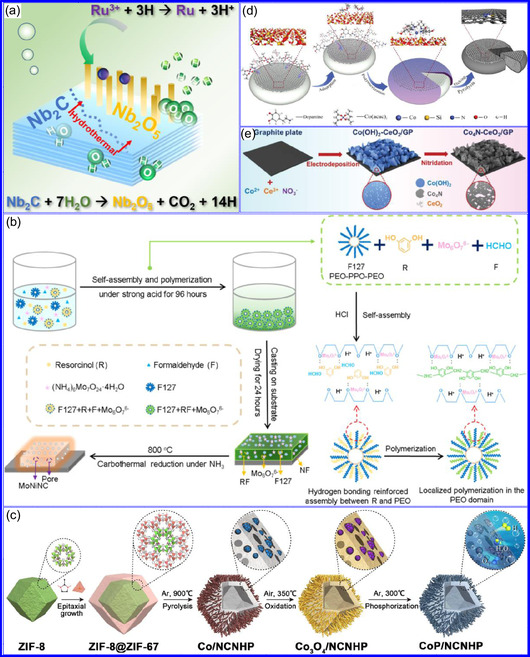
Preparation strategy of a) Ru@Nb_2_O_5_/Nb_2_C. Adapted with permission.^[^ ^324^ ^]^ Copyright 2022, Elsevier B.V. b) MoNiNC. Adapted with permission.^[^ ^330^ ^]^ Copyright 2017, Elsevier B.V. c) CoP/NCNHP. Adapted with permission.^[^ ^337^ ^]^ Copyright 2018, American Chemical Society. d) *p*‐CoSi_1_N_3_@D. Adapted with permission.^[^ ^342^ ^]^ Copyright 2022, Wiley‐VCH. e) Co_4_N‐CeO_2_/GP. Adapted with permission.^[^ ^345^ ^]^ Copyright 2020, Wiley‐VCH.

Khalifah and collaborators^[^ ^336^ ^]^ utilized a solid‐state synthesis to produce nano‐scale Co_0.6_Mo_1.4_N_2_, yielding a highly active electrocatalyst for the HER. The material's unique architecture, characterized by densely arranged N layers interspersed with Mo atoms in interstitial sites, facilitated structural distortion that improved bond selectivity. Impressively, this catalyst reached a current density of 10 mA cm^−2^ at an overpotential merely 0.1 V above that of commercial Pt/C, demonstrating excellent catalytic efficiency and operational stability. In a parallel study, Liu's group^[^ ^337^ ^]^ engineered a CoP/NCNHP composite via a multi‐step pyrolysis‐oxidation‐phosphidation protocol starting from ZIF‐8@ZIF‐67 precursors. Embedding CoP NPs within N‐CNT hollow polyhedral matrix imparted robust bifunctional electrocatalytic activity. The catalyst exhibited a low cell voltage of 1.64 V at 10 mA cm^−2^ and maintained structural integrity over 36 h of continuous operation. DFT analyses attributed enhanced HER kinetics to electron transfer from the NCNHP scaffold to CoP, which tuned the Co d‐band electronic structure near the Fermi level, favoring H_2_ adsorption. Morphological characterization via SEM, TEM, and HAADF‐STEM confirmed uniform rhombic dodecahedral geometry and successful formation of core‐shell architectures. Pyrolysis and selective etching yielded hollow structures rich in CNTs, significantly improving electron mobility. The long‐term durability was credited to the NCNHP shell, which conferred oxidation protection to CoP NPs (**Figure** **33**c). Collectively, this section emphasizes heteroatom doping as a key strategy in refining TMN‐based HER electrocatalysts, enhancing electron transport, structural robustness, and catalytic performance.

### 4.4 Defect Engineering

Defect engineering has garnered significant attention as a versatile strategy to enhance the electrochemical performance of 2D TMNs. By modulating surface and structural characteristics, including atomic vacancies, fractures, grain boundaries, and lattice distortions, researchers can significantly elevate catalytic activity.^[^ ^338^ ^]^ This methodology enables materials with intrinsically low conductivity to be transformed into semimetallic or metallic forms, thereby improving their electrocatalytic properties. Although the integration of defect engineering for optimizing HER and OER is still developing, recent progress has been notable.^[^ ^339^ ^]^ For instance, Zhang and collaborators^[^ ^340^ ^]^ fabricated bifunctional δ‐MnO_2_ NSs electrode incorporating O‐vacancies, which led to increased active site density, improved electrical conductivity, and enhanced performance in both HER and OER. Likewise, the strategic incorporation of N‐vacancies into PCN was found to redistribute electron density toward neighboring C atoms, thus amplifying HER activity.

Bao's team^[^ ^341^ ^]^ synthesized dr‐Ni_3_FeN NCs integrated with N‐doped graphene via controlled heating and nitridation. These composites demonstrated remarkable performance in alkaline media, surpassing both minimally modified counterparts and commercial IrO_2_, as evidenced by reduced overpotentials and elevated turnover frequencies. Similarly, Cheng and colleagues^[^ ^315^ ^]^ utilized NaCl templating and partial ammoniation of MoO_3_ NSs to produce dr‐MoN with outstanding HER activity across varying pH levels, highlighting its applicability in versatile OWS systems. Yang's team^[^ ^342^ ^]^ developed a 3D self‐supporting Co SACs (p‐CoSi_1_N_3_@D), leveraging diatomite as a dual‐function Si source and porous support. Both theoretical modeling and empirical data revealed that Si substitution for N enhanced electron conductivity and promoted effective interaction between PMS and the active Co center. The inherited hierarchical porosity facilitated high Co site accessibility and efficient mass transport, resulting in a turnover frequency (TOF) of 299.8 min^−1^ in bisphenol degradation. Collectively, these pioneering efforts illustrate the transformative impact of defect engineering on 2D TMNs, offering a promising route to improved HER and OER performance through tailored surface and electronic modifications.

### 4.5 Self‐Supported Structural Engineering

Recent investigations underscore the importance of improved electrical conductivity in optimizing the catalytic activity of TMNs. Enhanced conductivity mitigates electron transfer resistance and promotes faster reaction kinetics. One effective strategy involves anchoring TMNs onto highly conductive substrates such as NF or CF, which not only increase active site density but also enhance mass transport, resulting in improved electrocatalytic efficiency.^[^ ^343^ ^]^ Additionally, self‐supported catalysts, which operate without auxiliary binders or conductive agents, exhibit decreased resistivity and greater accessibility to active sites. For example, CoN NWAs synthesized on CC via a hydrothermal‐nitridation route demonstrated low overpotentials of 97 mV (HER) and 251 mV (OER) at 10 mA cm^−2^.^[^ ^344^ ^]^ The development of self‐supported mono‐ and bimetallic nitrides has also garnered attention. Notably, bi‐metallic NiCo_2_N showed excellent HER activity, requiring overpotentials of only 48 mV and 149 mV at 10 and 100 mA cm^−2^, respectively, attributed to its high electrical conductivity and large SSA. Designing self‐supported electrodes with hydrophilic surfaces further enhances performance under high current densities by facilitating charge transport and gas release. A prime example is the superhydrophilic Co_4_N‐CeO_2_ composite NSAs fabricated on a graphite plate through anion‐assisted electrodeposition and subsequent nitridation, achieving a cell voltage of 1.507 V at 10 mA cm^−2^ and operational stability for 50 h at 500 mA cm^−2^.^[^ ^345^ ^]^ Other self‐supported TMNs, including NiMo‐N NWs on NF and porous NiCo‐N NWs on CC, as well as Co‐, W‐, and Fe‐Ni‐based systems, have similarly demonstrated remarkable performance in OWS.^[^ ^346^ ^]^

Qu's group^[^ ^347^ ^]^ synthesized VN NWs clusters on CC (VN/CC) through nitridation of V_2_O_5_, resulting in increased exposure of active sites and enhanced electrolyte diffusion. This catalyst achieved an NH_3_ synthesis rate of 2.48 × 10^−10^ mol s^−1^ cm^−2^ and a FE of 3.58% in acidic media. In another study, Ni_3_N NPs dispersed on a C substrate (Ni_3_N/C) exhibited improved hydrogen oxidation reaction (HOR) activity due to electron transfer from the Ni_3_N to the C support. Additionally, a CoN@NC composite on NF demonstrated exceptional trifunctional catalytic behavior across OER, HER, and ORR, which was attributed to its 3D porous configuration and self‐supported framework.^[^ ^348^ ^]^ Despite these advancements, self‐supported TMN‐based catalysts face potential structural and compositional degradation under prolonged operation or high current densities, which can impair their electrocatalytic behavior. Mitigating these issues necessitates a detailed understanding of substrate‐catalyst interactions, wettability at the catalyst‐electrolyte interface, and the fundamental mechanisms that govern both catalytic activity and stability. In summary, conductivity enhancement plays a pivotal role in minimizing electron transfer resistance and improving TMN efficiency. While conductive supports and self‐supported configurations significantly advance HER and OER outcomes, ensuring long‐term operational stability remains a critical focus for future research.

## 5 Applications

TMNs exhibit outstanding chemical, electronic, and structural characteristics, making them highly adaptable for numerous applications. Their superior conductivity, stability, and catalytic performance have driven major progress in energy conversion, storage technologies, and environmental remediation. This section highlights the wide‐ranging uses of TMNs in areas such as photocatalysis, electrocatalysis, and energy devices, including HER, OER, CO_2_RR, OWS, as well as their roles in the development of batteries, SCs, and solar cells.

### 5.1 Batteries

LSBs are gaining attention as an alternative to conventional LIBs, owing to their ability to exploit S's natural abundance, environmental compatibility, and outstanding theoretical C_sp_ of 1675 mAh g^−1^.^[^ ^349^ ^]^ These features contribute to the superior theoretical energy efficiency of LSBs. Nonetheless, several inherent issues, such as the insulating characteristics of S, limited solubility of Li_2_S_2_ and Li_2_S, considerable volume changes during GCD cycles, and the notorious polysulfide shuttle effect, continue to pose significant challenges.^[^ ^350^ ^]^ In response, the scientific community has advanced strategies including the development of optimized cathode structures, multifunctional binders, enhanced separators, and interlayer configurations.^[^ ^351^, ^352^ ^]^ Carbonaceous materials like porous C,^[^ ^353^ ^]^ CNTs,^[^ ^354^ ^]^ spheres,^[^ ^355^ ^]^ and graphene,^[^ ^356^ ^]^ have been explored for their physical trapping capabilities of LiPSs, though their nonpolar nature weakens polysulfide interactions. Alternatively, polar materials, especially TMNs, exhibit strong LiPSs affinity, high conductivity, and catalytic efficiency.^[^ ^357^ ^]^ Yet, excessively strong TMNs, Li_2_S_2_/Li_2_S interactions may hinder the desorption of end products, leading to sluggish kinetics. Hence, rational design of TMNs with balanced binding affinity is essential for promoting efficient catalysis and robust cycling performance in LSBs.^[^ ^358^ ^]^

**Figure 34:**
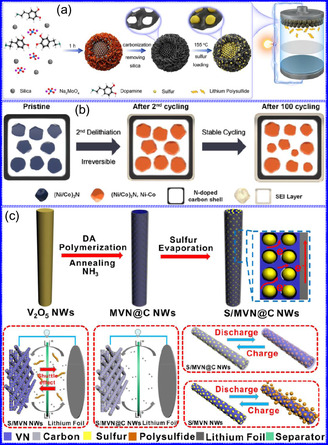
Diagram illustrating the a) cycle stability of C‐MoN_*x*_‐O. Adapted with permission.^[^ ^359^ ^]^ Copyright 2022, Wiley‐VCH. b) Conversion reaction process of (Ni/Co)_3_N MC@H. Adapted with permission.^[^ ^360^ ^]^ Copyright 2021, Elsevier B.V. c) S/MVN@C NWs cathodes during cycling. Adapted with permission.^[^ ^362^ ^]^ Copyright 2017, Elsevier B.V.

In a recent study, Cheng and colleagues^[^ ^359^ ^]^ introduced O‐regulated MoN clusters (C‐MoN_*x*_‐O) as a strategy to enhance the surface interaction between insoluble Li_2_S_*x*_ and TMNs in LSBs. The C‐MoN_*x*_‐O materials demonstrated a moderated binding energy toward Li_2_S_2_ and Li_2_S, thereby promoting efficient and reversible polysulfide catalysis. Li‐S cells utilizing C‐MoN_*x*_‐O electrodes exhibited a high initial discharge capacity of 875 mAh g^−1^ at 0.5 C and maintained stability with a remarkably low‐capacity fade of only 0.1% per cycle across 280 cycles. Supporting evidence from both electrochemical characterization and computational modeling highlighted the superior catalytic activity and suppression of intermediate species, resulting in significantly enhanced kinetics, rate capability, CE, and long‐term cycling behavior compared to conventional MoO_*x*_ and MoN electrodes (**Figure** **34**a).

In an innovative approach, Ryu and colleagues^[^ ^360^ ^]^ engineered a C‐doped (Ni/Co)_3_N multicore nanostructure embedded within a hollow N‐C framework for LIBs anode applications. The use of a PDA coating effectively preserved the 3D architecture of the NCs and mitigated NP aggregation during synthesis. This (Ni/Co)_3_N MC@HC composite was fabricated through an in situ heat‐annealing, retaining its structural integrity throughout. This unique design offers multiple advantages: high Li storage capacity via the conversion reaction of (Ni/Co)_3_N, enhanced electrical conductivity from C doping, and robust cycling stability. Electrochemical testing confirmed the superior performance of this architecture over conventional (Ni/Co)_3_N@C core‐shell materials.^[^ ^361^ ^]^ Postcycling characterization revealed the disappearance of (Ni/Co)_3_N peaks after the second discharge, replaced by Ni peaks that broadened over 100 cycles, indicative of metallic Ni segregation. Meanwhile, Co signals diminished, suggesting disorder and integration of Co into the electrode matrix (Figure 34b).

Huo and colleagues^[^ ^362^ ^]^ introduced an advanced S cathode architecture for LSBs, incorporating S NDs (2–5 nm) uniformly dispersed within mesoporous VN NWs (MVN@C NWs), which are further encapsulated by a microporous C matrix. This hierarchical design promotes effective confinement of LiPSs through synergistic chemical interactions with the conductive VN network and physical restriction by the C coating. The resulting self‐supporting, binder‐free cathode structure demonstrates exceptional mechanical flexibility and electrochemical performance. It delivers a stable capacity of 636 mAh g^−1^ over 200 cycles at 1 C and maintains 543 mAh g^−1^ at 10 C. Coin cell evaluations with Li metal counter electrodes revealed high reversibility and capacity retention, with a maximum reversible capacity of 1040 mAh g^−1^ at 1 C and a S loading of 2.8 mg cm^−2^. CV profiles exhibited well‐defined, consistent redox peaks over five cycles, indicating strong electrochemical reversibility (Figure 34c).

**Figure 35:**
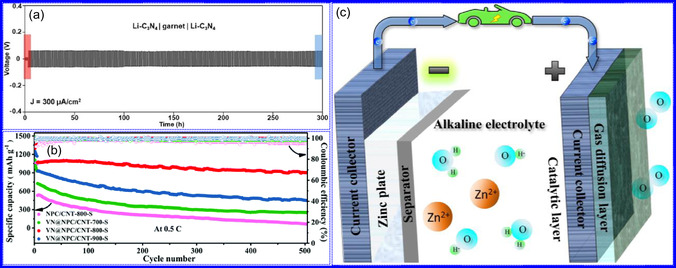
a) Long lasting plating/stripping profiles of symmetric Li‐C_3_N_4_/garnet/Li‐C_3_N_4_ cell at ambient atmosphere. Adapted with permission.^[^ ^363^ ^]^ Copyright 2020, Wiley‐VCH. b) Cycle stability of VN@NPC/CNT‐T‐S and NPC/CNT‐800‐S at 0.5 C. Adapted with permission.^[^ ^364^ ^]^ Copyright 2021, Royal Society of Chemistry. c) Pictorial demonstration of fabrication of aqueous ZABs. Adapted with permission.^[^ ^365^ ^]^ Copyright 2022, Elsevier B.V.

In a recent advancement, Huang and co‐workers^[^ ^363^ ^]^ introduced g‐C_3_N_4_ as an innovative surface modification layer for Li metal anodes in solid‐state Li metal batteries (LMBs). The application of g‐C_3_N_4_ significantly improves the interfacial compatibility between Li and garnet‐type solid‐state electrolytes (SSEs) by transitioning from discrete point contacts to a uniform and intimate interfacial structure. This improved wettability, along with the increased surface viscosity of molten Li and the formation of a beneficial Li_3_N layer, effectively mitigates dendritic growth. Consequently, symmetric cells incorporating Li‐g‐C_3_N_4_ electrodes display markedly reduced interfacial resistance (11 Ω cm^−2^) and support an impressive critical current density of 1500 mA cm^−2^, in stark contrast to unmodified Li electrodes which exhibit a higher resistance of 428 Ω cm^−2^ and a much lower CCD of 50 mA cm^−2^ (**Figure** **35**a).

Li's group^[^ ^364^ ^]^ introduced a novel synthetic approach for generating ultrafine VN NPs uniformly dispersed within a NPC matrix. This methodology utilizes a primary‐chain imidazolium‐based ionic polymer (ImIP) containing metavanadate anions as a multifunctional precursor. Not only does ImIP serve as a source of C and N, but it also effectively suppresses VN NPs aggregation during thermal treatment. The resultant VN NPs exhibit high polarity and catalytic activity, significantly improving the redox kinetics of S species and polysulfide immobilization in LSBs. This synthesis route offers a facile, NH_3_‐free pathway to optimize VN‐based functionality. Electrochemical testing at 0.5 C demonstrated an initial capacity of 1069 mAh g^−1^ and a retained capacity of 906 mAh g^−1^ after 500 cycles, with an ultralow fading rate of 0.03% per cycle and an average CE of 99.6%. Extended cycling at 5 C confirmed a degradation rate as low as 0.035% over 1200 cycles (Figure 35b).

Zhang and colleagues^[^ ^365^ ^]^ discuss the progress in deploying TMNs as efficient electrocatalysts for the ORR and OER in ZABs. ZABs are primarily classified based on their electrolyte composition into aqueous and non‐aqueous types. The latter utilizes ambient ionic liquids or quasi‐solid electrolytes, while aqueous ZABs typically employ alkaline solutions such as LiOH, NaOH, or KOH. These solutions are favored for their low viscosity and high ionic conductivity, which enhance the performance of both Zn electrodes and catalytic materials (Figure 35c). The review also reflects broader advancements in battery technologies, where innovations like S NDs within mesoporous VN NWs, C‐doped (Ni/Co)_3_N multicore clusters, and g‐C_3_N_4_ surface coatings have led to significant gains in capacity, stability, and energy efficiency across LSBs, LIBs, and LMBs. Additionally, embedding ultrafine VN NPs in NPC has shown exceptional electrochemical performance, underscoring the critical role of advanced materials and structural engineering in next‐generation energy storage.

### 5.2 SCs

SCs have emerged as promising energy storage systems due to their exceptional GCD rates, extended operational lifespan, and superior power density compared to conventional batteries.^[^ ^366^ ^]^ A critical objective for practical implementation is the attainment of high energy output with minimal input, which is fundamentally dependent on the properties of electrode materials.^[^ ^367^ ^]^ Conducting polymers and TMOs have been extensively investigated for their ability to contribute pseudocapacitance through rapid and reversible redox processes in aqueous electrolytes. Concurrently, C‐based materials are recognized for their high electrical conductivity, mechanical robustness, and excellent cycling performance, albeit their energy storage capacity is typically limited due to the dominance of EDLC at the electrode‐electrolyte interface.^[^ ^368^ ^]^ As such, current research is increasingly focused on hybridizing these materials to synergistically combine pseudocapacitive and EDLC behaviors, thereby enhancing both energy density and charge retention in next‐generation SCs.^[^ ^369^ ^]^

**Figure 36:**
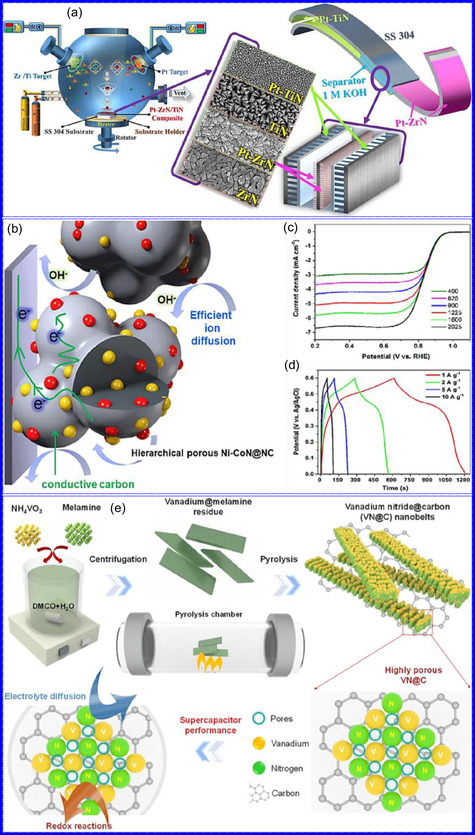
a) Planned working mechanism of flexible ASCs employing the co‐sputtering strategy. Adapted with permission.^[^ ^370^ ^]^ Copyright 2022, Elsevier B.V. b–d) Pictorial demonstration of charge transfer activity and energy storage performance of Ni‐CoN@NC. Adapted with permission.^[^ ^371^ ^]^ Copyright 2019, American Chemical Society. e) Diagram illustrating the device fabrication and electrochemical behavior of VN@C//AC. Adapted with permission.^[^ ^372^ ^]^ Copyright 2022, Elsevier B.V.

In their study, Chandra and collaborators^[^ ^370^ ^]^ explored the synthesis of nanostructured composite thin‐film electrodes by incorporating trace amounts of Pt into ZrN and TiN matrices using an in situ co‐sputtering on flexible stainless‐steel substrates. The introduction of Pt significantly enhanced the electrical conductivity, electrochemical performance, and stability of the resulting electrodes. These enhancements translated into improved performance in ASCs assembled using the Pt‐ZrN and Pt‐TiN electrodes. The device configuration employed an oscillating electrode design, with glass microfiber filter paper soaked in 1 M KOH aqueous electrolyte serving as the separator. This setup enabled an extended voltage window (0–3.2 V) and increased energy density. The ASCs demonstrated a C_sp_ of 60.2 F g^−1^, achieved an energy density of 85 Wh kg^−1^, and maintained 76.8% of their original C_sp_ after 40 000 GCD cycles (**Figure** **36**a).

Wang's team^[^ ^371^ ^]^ reported a novel method for synthesizing Ni‐CoN@NC nanostructures by integrating Ni_3_N and CoN NPs into a N‐C framework through MOF‐derived precursors. The resulting materials exhibited remarkable electrochemical stability and dual functionality as ORR catalysts and high‐performance SC electrodes. Key enhancements in performance are attributed to the synergistic interactions between TMNs and NPC matrices, which facilitate high electron mobility, robust structural stability, and enhanced electrolyte‐electrode interfaces. The Ni‐CoN@NC electrode achieved an ORR onset potential of 0.97 V versus RHE and a half‐wave potential of 0.84 V, along with outstanding durability. The porous architecture also promotes rapid ion transport, further enhancing capacitance (Figure 36b–d).

Joo and colleagues^[^ ^372^ ^]^ introduced an innovative single‐step chemical approach for synthesizing composite electrodes comprising porous CC and VN. In the configuration of ASC devices, VN NPs embedded on C NBs (VN@C) were employed as the positive electrode, while AC served as the negative counterpart. The resulting ASC demonstrated remarkable electrochemical performance, delivering a high C_sp_ of 1850 C g^−1^ at 10 A g^−1^, superior rate capability with 98% retention over 2000 cycles at 30 A g^−1^, and notable energy and power densities of 30 Wh kg^−1^ and 5608 W kg^−1^, respectively (Figure 36e).

**Figure 37:**
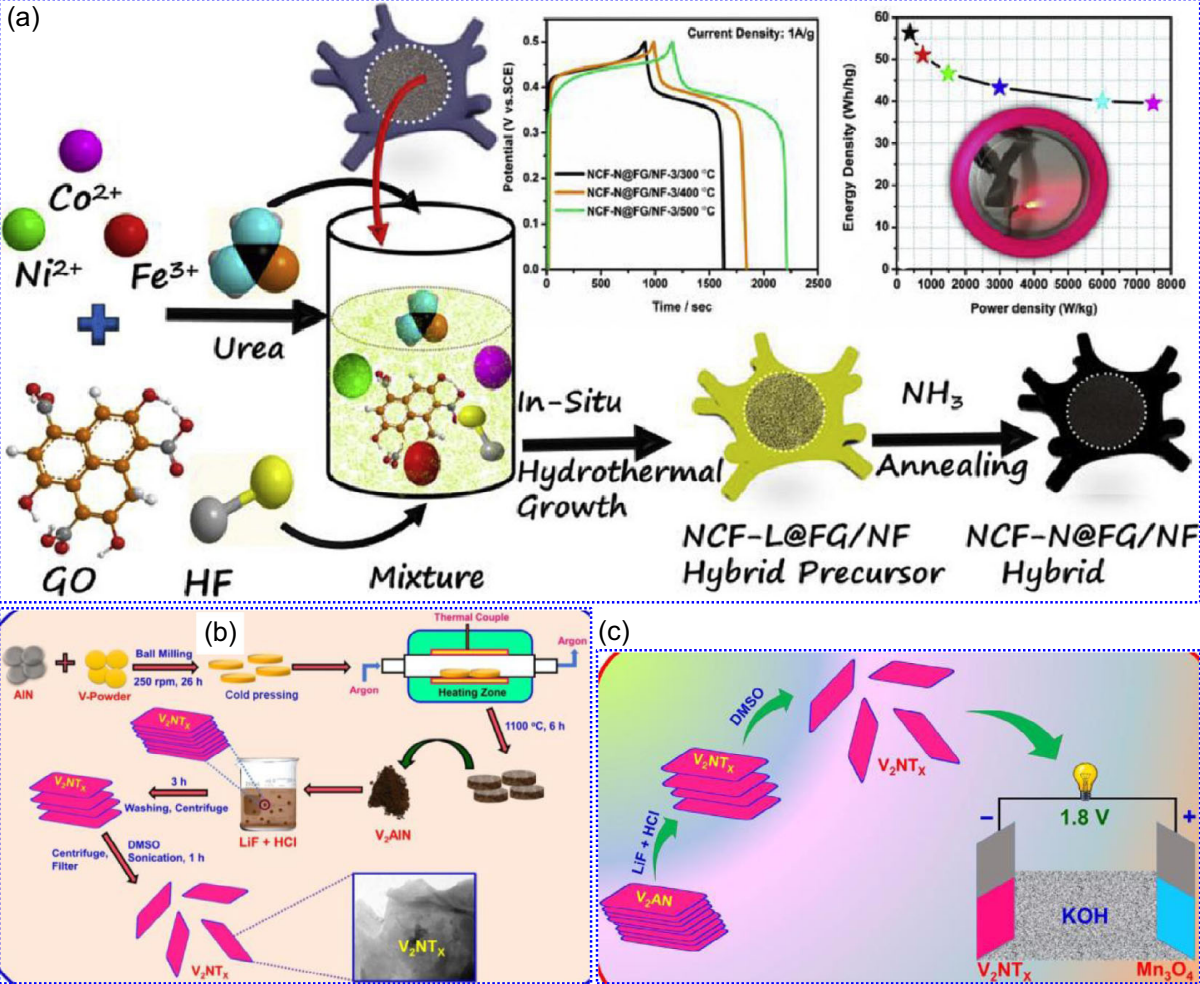
a) Planned working mechanism of NCF‐N@FG/NF‐3. Adapted with permission.^[^ ^373^ ^]^ Copyright 2018, Elsevier B.V. b,c) Preparation strategy, and planned working mechanism of V_2_NT_*x*_ MXene. Adapted with permission.^[^ ^374^ ^]^ Copyright 2020, American Chemical Society.

Deng's group^[^ ^373^ ^]^ developed a high‐performance SC electrode composed of fluorinated graphene (FG) decorated with Ni*‐Co*‐Fe trimetallic nitride NPs on a NF substrate, denoted as NCF‐N@FG/NF. The electrode was synthesized through thermal ammonolysis, with annealing temperatures (300, 400, and 500 °C) systematically varied to assess their influence on structural and electrochemical characteristics. An ASC device employing NCF‐N@FG/NF‐3/500 °C as the anode and AC‐coated NF (AC@NF) as the cathode demonstrated a stable voltage window of 1.5 V and retained 88.5% of its initial C_sp_ over 10 000 GCD cycles. The device delivered a maximum energy density of 56.3 Wh kg^−1^ at a power density of 374.6 W kg^−1^ and maintained 39.5 Wh kg^−1^ even at a high‐power density of 7484.2 W kg^−1^, highlighting its excellent efficiency and long‐term durability (**Figure** **37**a).

Grace's team^[^ ^374^ ^]^ developed a direct synthesis route for V_2_NT_*x*_ MXene via selective etching of Al from the V_2_AlN MAX phase. The V_2_AlN precursor was synthesized through ball milling of V and AlN powders, followed by cold pressing and thermal treatment to enhance structural cohesion. As a SC electrode in aqueous electrolyte, V_2_NT_*x*_ exhibited a C_sp_ of 112.8 F g^−1^ at 1.85 mA cm^−2^, delivering an energy density of 15.66 Wh kg^−1^ and a power density of 3748.4 W kg^−1^ (Figure 37b). An ASC was constructed using V_2_NT_*x*_ as the negative electrode and Mn_3_O_4_ NWs on CFs as the positive electrode. The ASC achieved an extended operating voltage of 1.8 V in aqueous KOH electrolyte, exceeding the typical 1.2 V constraint associated with symmetric devices limited by water decomposition (Figure 37c). These findings underscore the viability of aqueous systems for high‐voltage, safer energy storage. Collectively, this section highlights advancements in SC technologies, particularly through the integration of Pt, Ni–CoN, VN, and V_2_NT_*x*_‐based materials, which have yielded significant enhancements in C_sp_, energy, and power densities. Hybrid electrodes incorporating nitride NPs and C‐based matrices have demonstrated excellent electrochemical performance and durability. The emergence of flexible, high‐voltage SCs via innovative synthesis methods supports their application in next‐generation flexible and high‐performance energy storage systems.

### 5.3 Photocatalytic HER

Photocatalytic HER represents a crucial avenue for achieving sustainable H_2_ production. TMNs, including MoN, TiN, and VN, have garnered significant attention due to their exceptional catalytic properties, which stem from their inherent metallic conductivity and robust chemical stability.^[^ ^375^ ^]^ These properties enhance water dissociation and facilitate HER.^[^ ^376^ ^]^ Moreover, the narrow BGs characteristic of TMNs enable efficient absorption of UV light, thereby augmenting solar energy utilization, especially when integrated with photocatalysts such as g‐C_3_N_4_.^[^ ^377^ ^]^ TMNs also serve to reduce charge carrier recombination by promoting the effective separation and mobility of photogenerated electron–hole pairs, thereby increasing electron availability for H_2_ production.^[^ ^378^ ^]^ Widely employed as cocatalysts, TMNs contribute additional catalytic sites and facilitate charge transfer, leading to superior HER performance. Their structural durability under rigorous conditions renders them highly suitable for long‐term operation.^[^ ^379^ ^]^ Furthermore, synergistic effects between TMNs and other materials, particularly C‐based photocatalysts, further enhance photocatalytic efficiency.^[^ ^380^ ^]^ Consequently, TMNs play a vital role in the progression of efficient and sustainable HER technologies.^[^ ^381^ ^]^

**Figure 38:**
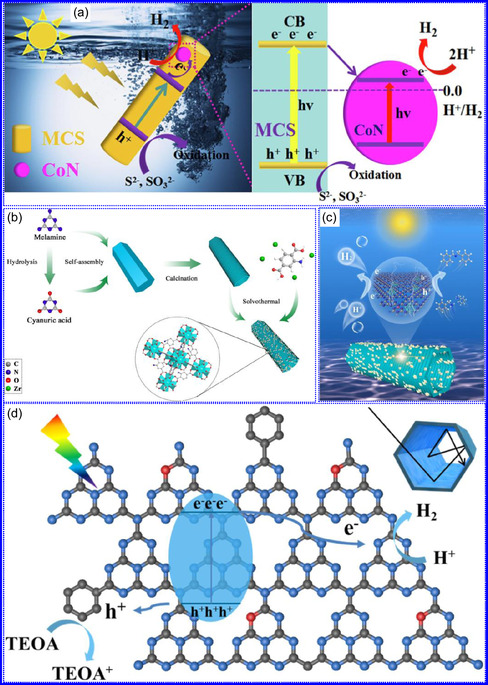
a) Photocatalytic HER activity for MCS composites. Adapted with permission.^[^ ^382^ ^]^ Copyright 2021, Elsevier B.V. b,c) Preparation strategy, and photocatalytic HER activity of p‐TCN@U6‐X composites. Adapted with permission.^[^ ^383^ ^]^ Copyright 2021, Elsevier B.V. d) Diagram illustrating the charge behavior of BOCN_0.1_ in photocatalysis. Adapted with permission.^[^ ^384^ ^]^ Copyright 2024, Elsevier B.V.

Jin's group^[^ ^382^ ^]^ synthesized composite photocatalysts by coupling Mn_0.2_Cd_0.8_S (MCS) solid solutions with CoN, a material characterized by high electrical conductivity. The integration of CoN markedly improved light‐harvesting capability and facilitated the separation of photoinduced charge carriers through effective electron trapping. XRD verified the crystallographic purity of both MCS and CoN, revealing that MCS corresponded to a HEX phase, while CoN matched a cubic structure. Increasing CoN content in the composites was reflected by intensified CoN diffraction peaks, confirming its successful incorporation. SEM and TEM showed close spatial association between MCS NRs (≈1 μm × 10 nm) and CoN NPs, mediated by electrostatic interactions, which promoted charge transfer. HR‐TEM exhibited distinct lattice fringes corresponding to MCS and CoN crystal planes, validating their integration. Photocatalytic HER assessments demonstrated that although CoN alone was inactive and MCS suffered from photocorrosion and charge recombination, the MCN_10_ composite (containing 10 wt% CoN) reached a HER rate of 14.612 mmol g^−1^ h^−1^, ≈17.3 times higher than that of pristine MCS. Excessive CoN, however, impaired performance due to light‐shielding effects and increased carrier trapping. The MCN_10_ sample also exhibited notable photocatalytic and structural stability over 25 h of operation, likely enhanced by the presence of a sacrificial reagent. Post‐reaction XRD confirmed the material's phase integrity (**Figure** **38**a).

Li's team^[^ ^383^ ^]^ synthesized phosphorus (P)‐doped tubular CN (p‐TCN) composites integrated with the MOF (UiO‐66‐NH_2_) (p‐TCN@U6‐X) via a solvothermal approach (Figure 38b). SEM and TEM revealed the uniform in situ deposition of octahedral UiO‐66‐NH_2_ microcrystals across the p‐TCN surface. Structural integrity of both p‐TCN and UiO‐66‐NH_2_ was preserved, particularly in the optimized formulation p‐TCN@U_6‐3_. The degree of integration was effectively modulated through the precise adjustment of ZrCl_4_ and NH_2_‐BDC precursor concentrations. Elemental mapping confirmed homogeneous dispersion of C, N, P, and Zr elements, evidencing successful P doping. Under visible light irradiation (*λ* > 420 nm) and TEA as a sacrificial agent, the p‐TCN@U_6‐3_ composite achieved a HER rate of 2628 μmol g^−1^ h^−1^, representing an 8.19‐fold and 5.36‐fold enhancement relative to pristine p‐TCN and UiO‐66‐NH_2_, respectively. The composite demonstrated robust catalytic performance and structural stability over four consecutive cycles. Substitution of p‐TCN with g‐C_3_N_4_ led to inferior HER activity, attributable to reduced light‐harvesting efficiency. The superior performance was ascribed to synergistic interactions between p‐TCN and UiO‐66‐NH_2_, with the latter serving effectively as a co‐catalyst. Additionally, the system exhibited excellent chemoselectivity (99%) and conversion efficiency (98%) in oxidative amine coupling reactions. The tubular architecture, P doping, and cooperative catalysis collectively underpinned the enhanced photocatalytic performance (Figure 38c).

Xu et al.^[^ ^384^ ^]^ prepared O‐doped, benzene‐modified g‐C_3_N_4_ NTs (BOCN) through a supramolecular self‐assembly strategy involving melamine and phenyl‐substituted melamine as precursors. By modulating the phenyl melamine content, they successfully tailored the phenyl group density and electronic structure of g‐C_3_N_4_, resulting in enhanced visible light absorption. Morphological studies via SEM and TEM demonstrated that traditional bulk g‐C_3_N_4_, obtained by calcining melamine, exhibited a compact, block‐like morphology, whereas BOCN variants formed well‐defined hollow tubular structures characterized by multilayered features and defect‐rich surfaces. These features stemmed from disrupted H‐bonding and the inhibition of heptazine ring formation. BET measurements revealed a substantial increase in SSA, particularly in BOCN_0.1_. However, excess benzene in BOCN_0.2_ led to aggregation during self‐assembly, decreasing the SSA. These morphological enhancements contributed to increased catalytic active sites, boosting proton reduction and HER activity. XRD showed diminished crystallinity in BOCN samples, while FTIR spectra confirmed the preservation of the g‐C_3_N_4_ framework, with intensified C‐C/C=C signals evidencing benzene incorporation. Among the samples, BOCN_0.1_ demonstrated the highest photocatalytic HER performance, attributed to its optimized structural and electronic configuration. The hollow NT morphology facilitated improved light absorption and reactant diffusion, while O‐doping elevated the valence band (VB) energy, and benzene groups promoted orbital separation, effectively suppressing charge carrier recombination. Upon photoexcitation, conduction band (CB) electrons reduced protons to H_2_, while VB holes oxidized TEA, thus enhancing charge separation and overall HER efficiency (Figure 38d).

**Figure 39:**
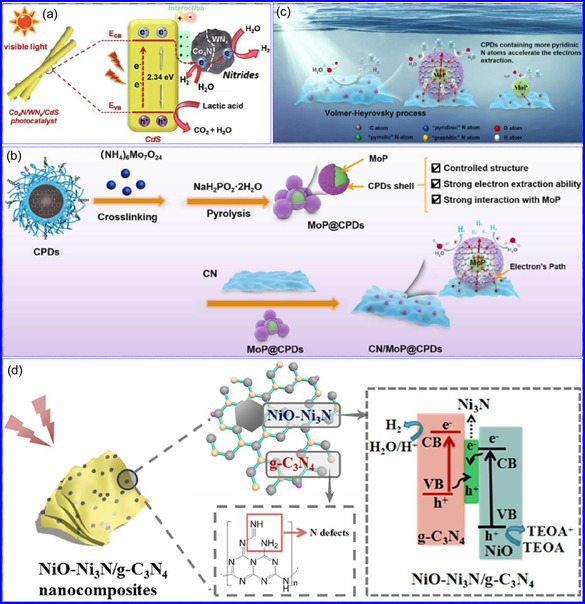
a) Diagram illustrating the photocatalytic HER activity of CWN‐CdS composites. Adapted with permission.^[^ ^385^ ^]^ Copyright 2021, Elsevier B.V. b,c) Preparation strategy, photocatalytic HER activity of CN/MoP@CPDs. Adapted with permission.^[^ ^386^ ^]^ Copyright 2023, Elsevier B.V. d) Photocatalytic HER activity of NiO‐Ni_3_N/g‐C_3_N_4_. Adapted with permission.^[^ ^387^ ^]^ Copyright 2020, Elsevier B.V.

Yang and collaborators^[^ ^385^ ^]^ reported a straightforward electrostatic self‐assembly technique for constructing 1D Co_4_N‐WN_*x*_‐CdS composites designed to enhance photocatalytic HER activity. TMNs, specifically Co_4_N and WN_*x*_, were chosen due to their superior electrical conductivity and high density of catalytically active sites, which collectively facilitate efficient charge transport and minimize the overpotential required for HER. The resulting Co_4_N‐WN_*x*_‐CdS heterostructure exhibited a remarkable HER rate of 14.42 mmol g^−1^ h^−1^ under vacuum illumination, representing an ≈8‐fold enhancement over Pt‐CdS (1.78 mmol g^−1^ h^−1^). XRD of the CoWO_4_ precursor verified the formation of a homogeneous crystalline phase via hydrothermal treatment, with an estimated crystallite size of 46 nm. Under visible light irradiation, the coupling of CdS with Co_4_N‐WN_*x*_ (CWN) significantly enhanced photocatalytic efficiency. Among all tested variants, the CWN‐10‐CdS sample achieved the highest HER performance, outperforming both pristine CdS and Pt‐loaded CdS. However, excessive incorporation of CWN or Pt resulted in diminished activity, likely due to increased charge carrier recombination. Importantly, CWN‐10‐CdS also demonstrated a higher apparent quantum efficiency (AQY) compared to traditional Pt‐based systems, underscoring its potential as an effective, noble‐metal‐free co‐catalyst for solar‐driven OWS, as illustrated in **Figure** **39**a.

Sun's group^[^ ^386^ ^]^ devised an innovative approach employing carbonized polymer dots (CPDs) as an interfacial shell layer to engineer CN/MoP@CPDs composites for enhanced HER photocatalysis. The CPD shell formed uniform and robust interfaces with both the CN photocatalyst and the MoP cocatalyst (Figure 39b), functioning as a highly conductive interlayer that facilitated rapid and efficient charge transfer. MoP was selected due to its Pt‐like electronic structure, high electrical conductivity, and notable chemical stability. Among the synthesized materials, CN/MoP@CPDs‐200 demonstrated the highest HER activity, which was attributed to its enriched pyridinic N content (43.9 at%) that promoted surface electron isolation. Control experiments revealed diminished photocatalytic performance when CPDs were replaced with graphene QDs (GQDs) or when alternate synthesis methods were employed, highlighting the critical role of CPD structure and anchoring precision. Notably, CN/MoP@CPDs‐200 surpassed CN loaded with 3 wt% Pt in HER efficiency, establishing its promise as a noble‐metal‐free photocatalyst. Additionally, the composite exhibited superior AQE across a wide spectral range and maintained long‐term structural and functional stability, in contrast to CN/MoP, which showed a 42.7% decrease in HER activity over time (Figure 39c).

Yang and colleagues^[^ ^387^ ^]^ systematically examined the influence of oxidized impurities in TMNs on their photocatalytic HER activity, selecting Ni_3_N as a representative model. The study demonstrated that in situ calcination‐induced formation of a TMO‐TMN heterostructure significantly improved photocatalytic performance. This enhancement was primarily attributed to a Z‐scheme charge transfer mechanism, which facilitated efficient separation and migration of photoexcited charge carriers. TEM revealed the presence of expansive 2D NSs, exceeding 1 μm in lateral dimensions, dispersed within a g‐C_3_N_4_ matrix. Perpendicularly aligned Ni(OH)_2_ NSs were observed to grow on g‐C_3_N_4_, forming a flower‐like architecture. Upon thermal treatment, composites such as NiO/g‐C_3_N_4_, Ni_3_N/g‐C_3_N_4_, and NiO‐Ni_3_N/g‐C_3_N_4_ were synthesized, all preserving structural integrity while showing an increase in Ni‐based nanostructure content. Notably, nitridation under NH_3_ introduced N vacancies within Ni_3_N, further amplifying its HER performance. The optimized Ni_3_N/g‐C_3_N_4_ photocatalyst leveraged both Z‐scheme electron transfer and defect engineering to achieve superior activity (Figure 39d). More broadly, TMNs, including MoN, TiN, and VN, serve as vital components in HER systems due to their abundant active sites, high charge mobility, and mechanical durability. When integrated with C‐based materials or engineered into heterostructures, TMNs exhibit enhanced catalytic efficiency and long‐term operational stability, underscoring their potential in sustainable H_2_ production technologies.

### 5.4 Photocatalytic OER

**Figure 40:**
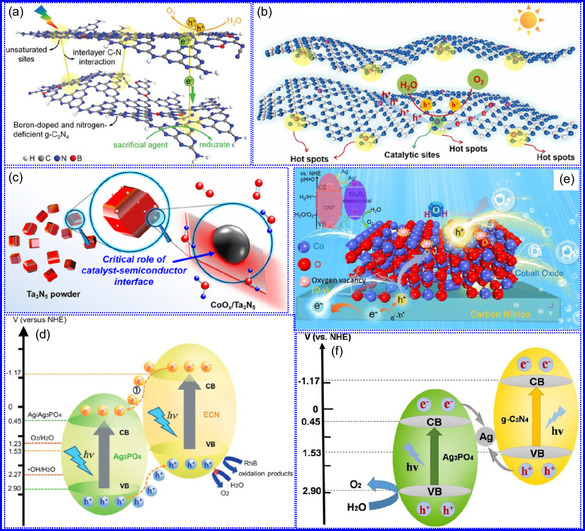
a) Diagram illustrating the photocatalytic OER activity of BH_*x*_. Adapted with permission.^[^ ^391^ ^]^ Copyright 2019, Wiley‐VCH. b) Mechanism of isolating B center over PCN‐B‐20 in photocatalytic OER activity. Adapted with permission.^[^ ^392^ ^]^ Copyright 2023, Elsevier B.V. Photocatalytic OER activity of c) Ta_3_N_5_. Adapted with permission.^[^ ^393^ ^]^ Copyright 2015, American Chemical Society. d) g‐C_3_N_4_ and Ag_3_PO_4_/g‐C_3_N_4_ composite. Adapted with permission.^[^ ^394^ ^]^ Copyright 2015, Wiley‐VCH. e) Co_3_O_4_ nanocrystals. Adapted with permission.^[^ ^395^ ^]^ Copyright 2020, American Chemical Society. f) g‐C_3_N_4_ NRs and g‐C_3_N_4_ NRs/Ag_3_PO_4_ composites. Adapted with permission.^[^ ^396^ ^]^ Copyright 2018, Elsevier B.V.

Photocatalytic OWS using semiconductor materials represents a promising approach for sustainable H_2_ production and the alleviation of global energy challenges.^[^ ^388^ ^]^ This reaction encompasses two critical half‐reactions: the HER and OER. Although substantial advancements have been made in enhancing HER performance, OER remains a significant impediment due to its complex 4‐electron transfer mechanism and high overpotential.^[^ ^389^ ^]^ Consequently, the development of innovative materials to catalyze OER more efficiently is of paramount importance.^[^ ^390^ ^]^ Shen and collaborators^[^ ^391^ ^]^ introduced a strategy involving the incorporation of B species and N vacancies into g‐C_3_N_4_ via thermal treatment with NaBH_4_ under a N_2_ atmosphere at various temperatures. The resulting materials, denoted as BH_*x*_ (where *x* corresponds to the calcination temperature), exhibited a pronounced enhancement in OER performance. Notably, BH_*x*_ achieved a maximum OER rate of 561.2 μmol h^−1^ g^−1^, representing a sixfold increase relative to pristine g‐C_3_N_4_. Structural analyses using XRD and FTIR confirmed significant modifications to the g‐C_3_N_4_ framework, attributable to B doping and the formation of N vacancies. These alterations facilitated band structure modulation, resulting in improved visible‐light absorption and enhanced catalytic activity for water oxidation (**Figure** **40**a).

Wang's group^[^ ^392^ ^]^ conducted a systematic study on the influence of B doping, specifically focusing on two coordination types, B‐N and B‐B, within the tri‐s‐triazine units of porous C_3_N_4_ NSs, to assess their effect on photocatalytic OER activity. The B‐doped porous C_3_N_4_ samples (PCN‐B‐X) were synthesized via a bubble‐template‐assisted process, followed by post‐calcination with NaBH_4_. Initially, melamine and NH_4_Cl served as precursors for the formation of porous C_3_N_4_ (PCN), where gas evolution from NH_4_Cl decomposition contributed to NSs thinning and pore generation. Subsequent thermal treatment in the presence of NaBH_4_ enabled efficient B incorporation. SEM image revealed that NSs morphology and size were largely retained post‐calcination, while EDS mapping verified the homogeneous distribution of C, N, O, and B elements. XRD data showed reduced peak intensities at 2*θ* = 13.1° and 27.3°, indicative of disrupted crystalline order and successful planar B doping. Photocatalytic performance was tested under visible light using AgNO_3_ as a sacrificial electron acceptor and La_2_O_3_ as a pH buffer. Among the variants, PCN‐B‐20 demonstrated the highest OER rate of 55 μmol h^−1^ g^−1^, approximately double that of undoped C_3_N_4_, primarily due to isolated B‐N coordination. In contrast, higher B concentrations promoted B‐B interactions, adversely affecting light absorption and reducing catalytic efficiency (Figure 40b).

Takanabe and colleagues^[^ ^393^ ^]^ explored the surface modification of Ta_3_N_5_ photocatalysts through the encapsulation of Co species using an NH_3_‐mediated deposition process. This technique facilitated the formation of metallic Co on the Ta_3_N_5_ surface, which enhanced OER activity. However, post‐reaction analyses indicated reoxidation of the Co species, negatively affecting the catalyst's long‐term durability. Photocatalytic OER activity was evaluated using CoO_*x*_/Ta_3_N_5_ both before and after mild oxidative treatments, employing AgNO_3_ and Na_2_S_2_O_8_ as sacrificial electron acceptors. The catalyst exhibited reduced activity with Na_2_S_2_O_8_, attributed to its inherently more complex 2‐electron reduction process relative to the simpler 1‐electron mechanism of Ag^+^. Notably, mild oxidation treatment improved OER performance from 40 to 67 μmol h^−1^, confirming CoO_*x*_ as the catalytically active species. The oxidized CoO_*x*_/Ta_3_N_5_ also demonstrated excellent operational stability, sustaining an OER rate of 100 μmol h^−1^ over 10 h. In parallel studies, PCN‐B‐20, featuring B‐N coordinated centers, achieved an OER rate of 55 μmol h^−1^ g^−1^, nearly twice that of undoped C_3_N_4_. Further enhancement was realized through the introduction of Co(OH)_2_ as a cocatalyst, which elevated the OER activity to 248.9 μmol h^−1^ g^−1^. However, prolonged operation led to deactivation, attributed to surface coverage from Ag^+^ photoreduction under irradiation (Figure 40c).

Shalom's team^[^ ^394^ ^]^ synthesized g‐C_3_N_4_/Ag_3_PO_4_ composite photocatalysts via a two‐step procedure to enhance their photocatalytic efficiency. These composites were systematically evaluated for OER performance and RhB dye degradation under visible light irradiation. The Ag_3_PO_4_/ECN heterostructure operates via a Z‐scheme charge transfer mechanism, which facilitates efficient O_2_ generation. The superior oxidative potential of Ag_3_PO_4_ enables the production of hydroxyl (^•^OH) radicals, whereas C‐centered hydroxyl (^•^COH) radicals are generated on ECN surfaces through ORRs. Additionally, both components are capable of promoting direct photo‐oxidation of RhB via hole transfer. The remarkable OER activity observed in Ag_3_PO_4_ is primarily attributed to its suitable VB alignment and intrinsic oxidative strength. Unlike conventional heterojunctions, the Ag_3_PO_4_/ECN system demonstrates band coupling between the CB of Ag_3_PO_4_ and the VB of ECN, leading to enhanced charge separation and overall photocatalytic performance (Figure 40d).

Luo's team^[^ ^395^ ^]^ engineered a nanocomposite comprising Co_3_O_4_ NCs enriched with O‐vacancies and exposing the catalytically favorable (222) crystal facets, uniformly anchored onto a 1D CNFs scaffold. This Co_3_O_4_‐CNF (COCNF) hybrid exhibited significantly enhanced photocatalytic OER activity under visible light irradiation in the presence of electron donors, achieving a maximum rate of 24.9 μmol h^−1^. The incorporation of a La_2_O_3_ buffering medium further enabled systematic evaluation, demonstrating that increasing Co_3_O_4_ loading improved OER performance up to an optimal threshold, beyond which overloading impeded the accessibility of active sites. In contrast, the standalone CNFs and bulk Co_3_O_4_ (BCO) exhibited inferior catalytic performance, largely due to rapid electron‐hole recombination and limited charge transport efficiency (Figure 40e).

Yang's group^[^ ^396^ ^]^ synthesized g‐C_3_N_4_ NRs through the thermal calcination of polymerized intermediates formed from MCA precursor. Upon integration with Ag_3_PO_4_, the resulting g‐C_3_N_4_ NRs/Ag_3_PO_4_ composites demonstrated a substantial improvement in OER activity compared to pristine Ag_3_PO_4_. The NR morphology was obtained via a self‐assembly approach followed by thermal processing. Of the prepared composites, TA‐600 exhibited the highest photocatalytic efficiency, achieving an OER rate of 110.1 mmol L^−1^ g^−1^ h^−1^, followed by TA‐400 at 96 mmol L^−1^ g^−1^ h^−1^. In contrast, TCN‐modified samples showed negligible activity, and both TA‐200 and TA‐800 underperformed relative to bulk Ag_3_PO_4_, indicating that a TCN dosage of 0.6 g optimally enhances catalytic performance (Figure 40f). Despite these advances, the OER component of OWS remains limited by the inherently sluggish 4‐electron transfer kinetics and associated overpotential. To overcome these constraints, strategies including B‐doping, N vacancy engineering, and hybrid composites such as Co_3_O_4_ NCs supported on CNFs have been pursued to improve charge separation, visible‐light response, and catalytic robustness.

### 5.5 POWS

Photocatalytic OWS (POWS) is emerging as an innovative approach to utilize solar energy for the generation of H_2_ fuel, which is vital in the field of artificial photosynthesis.^[^ ^397^ ^]^ Contrasting with traditional photoelectrochemical OWS, which relies on two electrodes placed in a conductive electrolyte, POWS operates effectively on the surface of a semiconductor in nearly neutral pH environments, such as pure or saline water. This characteristic enhances its feasibility for large‐scale applications.^[^ ^398^ ^]^ The successful realization of this approach requires semiconductors with specific electronic band structures; namely, the CB and VB must align adequately with the redox potentials of water, and the BG should be sufficiently narrow to enable effective absorption of visible sunlight.^[^ ^399^ ^]^ Despite extensive research, many known semiconductors either demonstrate suboptimal band edge alignment or present excessively large BGs. Techniques such as doping and surface modification have been explored to enhance the performance of wide‐BG materials like TiO_2_.^[^ ^400^ ^]^

**Figure 41:**
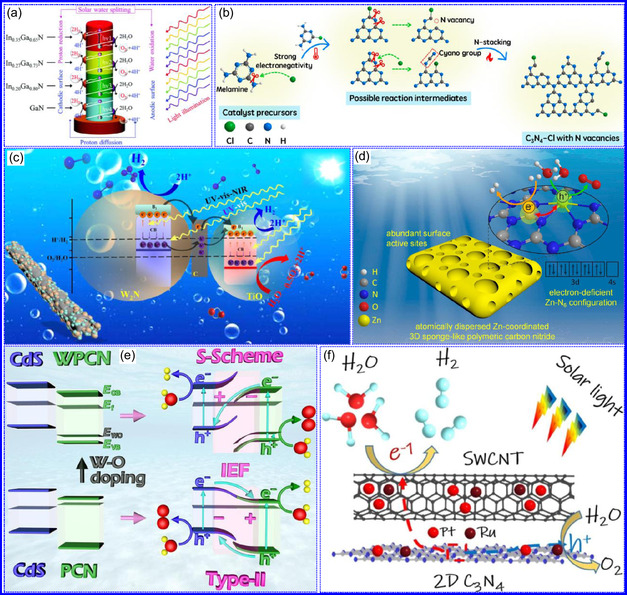
a) POWS behavior of quadruple‐band InGaN NWAs. Adapted with permission.^[^ ^401^ ^]^ Copyright 2019, Royal Society of Chemistry. b) Diagram illustrating the conversion method for C_3_N_4_. Adapted with permission.^[^ ^402^ ^]^ Copyright 2022, American Chemical Society. POWS behavior of c) W_2_N/C/TiO. Adapted with permission.^[^ ^403^ ^]^ Copyright 2021, Elsevier B.V. d) Zn‐PCN. Adapted with permission.^[^ ^404^ ^]^ Copyright 2022, Springer Nature. e) CdS/WPCN SSH. Adapted with permission.^[^ ^405^ ^]^ Copyright 2024, Elsevier B.V. f) 1D SWCNT/2D C_3_N_4_. Adapted with permission.^[^ ^406^ ^]^ Copyright 2023, Elsevier B.V.

Mi and collaborators^[^ ^401^ ^]^ investigated a groundbreaking configuration of quadruple‐band InGaN NWAs aimed at enhancing the efficiency of POWS. These InGaN NWs, capable of capturing light throughout the entire visible spectrum, possess distinct anode and cathode surfaces that facilitate effective water oxidation and proton reduction processes. This unique arrangement allows for meticulous control over charge carrier dynamics at the nanoscale level. As depicted in **Figure** **41**a, incoming photons stimulate electrons across all bands, leading to the generation of spatially separated charge carriers. A gradient in magnesium (Mg) doping creates an internal electric field that efficiently directs electrons toward the cathode and holes toward the anode, thereby promoting the OER and minimizing the occurrence of charge recombination and parasitic reverse reactions. Furthermore, the implementation of a nonplanar Si substrate aids in the lateral distribution of the Mg gradient throughout the NWs, significantly enhancing charge carrier separation and overall photocatalytic performance.

Ye's group^[^ ^402^ ^]^ explored the impact of chloride (Cl^‐^) doping on the performance of polymerized g‐C_3_N_4_ photocatalysts by generating N‐vacancies. Their thermal polymerization approach, tailored through varying Cl^‐^ concentrations, facilitated the emergence of two‐coordinated N‐vacancies, leading to a notable enhancement in photocatalytic efficiency. The g‐C_3_N_4_ modified with a 4% Cl concentration (g‐C_3_N_4_‐Cl_4_) exhibited a remarkable 21‐fold increase in HER and OER rates compared to the unmodified g‐C_3_N_4_. Specifically, under visible light, the HER and OER improved from 2.3 and 1 μmol h^−1^ for pristine g‐C_3_N_4_ to 48.2 and 21.8 μmol h^−1^ for g‐C_3_N_4_‐Cl_4_, respectively. Among the halide modifications tested, Cl^‐^ proved to be superior to F^−^ and Br^−^, though excessive Cl^‐^ incorporation had an adverse effect. Remarkably, the g‐C_3_N_4_‐Cl_4_ photocatalyst maintained stable activity across 10 cycles with minimal structural deterioration, achieving an AQE of 6.9% at 420 nm, positioning it as one of the leading g‐C_3_N_4_ based photocatalytic systems documented to date (Figure 41b).

Li's team^[^ ^403^ ^]^ has devised innovative photocatalysts to enhance OWS through a novel two‐step Z‐scheme photoexcitation method that integrates W_2_N and TiO. While pristine TiO demonstrates the ability to absorb light across both UV and visible spectra, the introduction of C modifies TiO (C/TiO), further extending its light absorption into the NIR region (450–900 nm). The synergistic effect of combining C with W_2_N significantly broadens the material's spectral response, with W_2_N absorbing light up to 850 nm. Additionally, the structural incorporation of W_2_N/C/TiO alongside black CNTs results in a notable redshift in the NIR spectrum, which enhances solar photon utilization and consequently improves photocatalytic efficiency as illustrated in Figure 41c. In an alternative study, Shen and collaborators^[^ ^404^ ^]^ utilized an intermediate coordination approach to create atomically dispersed Zn‐doped, 3D sponge‐like PCN. The pronounced photocatalytic performance observed is attributed to the presence of Zn atoms, which modify the electronic structure of PCN, introducing mid‐gap energy states, increasing charge carrier density, and facilitating efficient charge separation and transport during the photocatalytic HER under visible light (*λ* > 420 nm). Baseline BCN generated H_2_ at a rate of 82.7 μmol h^−1^ g^−1^ in aqueous solutions using TEA as a hole scavenger, while Zn‐PCN (4.79%) exhibited a remarkable enhancement, achieving 1172.9 μmol h^−1^ g^−1^, over 14 times greater. Furthermore, Zn‐PCN (4.79%) enabled OWS in pure water, yielding H_2_ and O_2_ in a 2:1 molar ratio at evolution rates of 35.2 and 17.3 μmol h^−1^ g^−1^, respectively (Figure 41d).

Hou's group^[^ ^405^ ^]^ synthesized a PCN‐based material that incorporated W SAs coordinated to O‐functionalities (W‐O), denoted as WPCN. This modification aimed to optimize the material as an efficient semiconductor for the OER. Subsequent to this, they developed S‐scheme heterojunctions (SSH) by thermally depositing CdS on the WPCN substrate. In comparison to the previously established Pt@Cr_2_O_3_‐modified PCN/CdS type‐II heterojunction, the Pt@Cr_2_O_3_‐doped CdS/WPCN SSH exhibited markedly improved photocatalytic activity for OWS as well as enhanced photochemical stability. The presence of W‐O groups was instrumental not only in the formation of the SSH architecture but also in augmenting its functional efficiency. These enhancements effectively diminished the photocorrosion associated with CdS, a notable drawback of this photocatalyst, enabling CdS to serve proficiently as a HER photocatalyst. While some degradation was observed in the CdS regions distal to the heterojunction interface, enhancements in interfacial contact, potentially through the use of ultrathin NSs or QDs, may alleviate this concern. This investigation presents a broadly applicable methodology for the engineering of PCN‐based SSH systems (Figure 41e).

Xu's team^[^ ^406^ ^]^ engineered a novel composite photocatalyst that combines 1D SWCNTs with 2D g‐C_3_N_4_, targeting improvements in POWS. When tested under visible light without any sacrificial agents, this SWCNT/g‐C_3_N_4_ composite system, augmented with Pt and Ru as co‐catalysts, achieved an HER rate of 1346 μmol h^−1^ g^−1^, significantly outperforming both pristine and solely g‐C_3_N_4_ variants. Notably, the composite maintained robust photocatalytic activity even in the absence of co‐catalysts. Durability assessments conducted over four cycles indicated minimal degradation in HER performance, showcasing remarkable structural and functional integrity. Spectrally resolved analyses recorded a HER rate of 4026 μmol h^−1^ g^−1^ under irradiation from a 400 nm LED, yielding an AQE of 5.2% at 420 nm, a striking improvement relative to pure g‐C_3_N_4_. Mechanistic investigations revealed that g‐C_3_N_4_ acted predominantly as the light‐absorbing component, efficiently transferring electrons to the SWCNTs to enhance charge separation. This facilitated electron accumulation at Pt sites for H_2_ production, while Ru catalyzed the OER. The research underscored the role of nonresonant, concentrated plasmonic effects in semiconductor‐based heterostructures and proposed innovative strategies for creating nonmetallic plasmonic photocatalysts (Figure 41f). Overall, the discussed advancements in POWS signal the significance of tailored material designs. Strategies like introducing N vacancies, B doping in g‐C_3_N_4_ NSs, Co encapsulation on Ta_3_N_5_, and hybrid systems such as g‐C_3_N_4_/Ag_3_PO_4_ and Co_3_O_4_/CNFs have proven effective in enhancing charge transport, increasing catalytic active sites, and boosting system durability, ultimately facilitating more efficient solar‐to‐fuel conversion and presenting potential applications in energy and environmental fields.

### 5.6 Photocatalytic H_2_O_2_ Production

H_2_O_2_ is recognized as a highly effective oxidizing agent with widespread applications in industrial, environmental, and biological contexts.^[^ ^407^ ^]^ In the realm of environmental remediation, its oxidative potential enables it to independently or synergistically break down a wide range of pollutants.^[^ ^408^ ^]^ Additionally, H_2_O_2_ decomposes into harmless H_2_O and O_2_, positioning it as an eco‐friendly alternative for various applications, including organic synthesis, textile bleaching, and fuel cell technology. Traditional methods for large‐scale production, such as anthraquinone autoxidation, alcohol oxidation, and electrochemical synthesis, tend to be energy‐intensive, potentially hazardous, and frequently result in unwanted byproducts.^[^ ^409^ ^]^ Consequently, there is a heightened interest in the development of cost‐effective, safe, and sustainable approaches to H_2_O_2_ production.^[^ ^410^ ^]^ Semiconductor photocatalysis presents a promising alternative by utilizing solar energy to convert O_2_ and H_2_O into H_2_O_2_ under ambient conditions.^[^ ^411^ ^]^ TiO_2_, a prevalent UV‐active photocatalyst, has demonstrated effectiveness in the generation of H_2_O_2_. Nevertheless, given that UV light constitutes only 4% of the solar spectrum and considering the rapid self‐decomposition of H_2_O_2_ under UV exposure, the advancement of visible‐light‐responsive photocatalysts is crucial for optimizing H_2_O_2_ production.^[^ ^412^ ^]^

**Figure 42:**
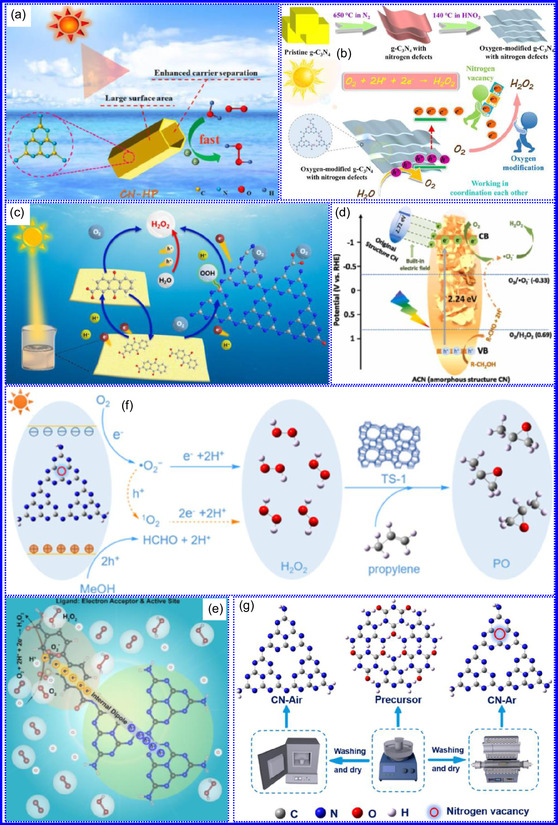
Photocatalytic H_2_O_2_ production activity of a) CN‐HP. Adapted with permission.^[^ ^413^ ^]^ Copyright 2021, Elsevier B.V. b) g‐C_3_N_4_. Adapted with permission.^[^ ^414^ ^]^ Copyright 2023, Elsevier B.V. c) AQ/U‐POCN. Adapted with permission.^[^ ^415^ ^]^ Copyright 2021, Elsevier B.V. d) CAN. Adapted with permission.^[^ ^416^ ^]^ Copyright 2022, Elsevier B.V. e) UCN, UCN‐TDA, UCN‐SDA, and UCN‐DTDA. Adapted with permission.^[^ ^417^ ^]^ Copyright 2023, American Chemical Society. f,g) CN‐Ar, and preparation strategy of CN‐Air and CN‐Ar. Adapted with permission.^[^ ^418^ ^]^ Copyright 2023, American Chemical Society.

Ye's group^[^ ^413^ ^]^ fabricated 1D HEX prisms of CN (CN‐HPs) featuring a hollow design through a hydrothermal modification of the C‐N precursor. This distinctive hollow tubular structure significantly enhances the SSA and promotes an increase in surface O_2_ adsorption. Additionally, the architectural design effectively optimizes the spatial separation of photoexcited charge carriers. Consequently, CN‐HPs demonstrate markedly superior photocatalytic behavior for H_2_O_2_ production, achieving rates nearly seven times higher than those of conventional 2D g‐C_3_N_4_. The evaluation of photocatalytic capability was conducted by measuring H_2_O_2_ generation via the ORR, using IPA as the proton donor. Under visible light irradiation for 40 min, CN‐HP yielded 4.08 mmol of H_2_O_2_, in contrast to only 0.59 mmol produced by traditional g‐C_3_N_4_. Notably, the performance in saline water was equivalent to that in DI water, highlighting the technique's resilience in marine environments. An AQE of 2.41% at 420 nm was observed. Electron spin resonance (ESR) measurements confirmed the generation of superoxide radicals (O_2_^•‐^) through the photoinduced reduction of O_2_, leading to H_2_O_2_ synthesis. The observation of singlet oxygen further substantiates enhanced activity, presenting a promising avenue for sustainable H_2_O_2_ generation (**Figure** **42**a).

Lv's group^[^ ^414^ ^]^ explored the synthesis of O‐modified g‐C_3_N_4_ with N vacancies via secondary calcination followed by acid‐etching. This modified photocatalyst was utilized for H_2_O_2_ production under visible light. Their results showed that the unmodified g‐C_3_N_4_ produced H_2_O_2_ at a rate of 24.79 μmol g^−1^ L^−1^. The application of secondary calcination improved this rate significantly, achieving 62.94 μmol g^−1^ L^−1^ for g‐C_3_N_4_‐ND_4_. The introduction of HNO_3_ further enhanced the performance, resulting in g‐C_3_N_4_‐ND_4_‐OM_3_ reaching an impressive 146.96 μmol g^−1^ L^−1^, nearly six times higher than the original catalyst. The study also assessed the effect of pH, finding that acidic conditions were conducive to H_2_O_2_ generation due to greater proton availability, while alkaline conditions hindered photocatalytic effectiveness. Furthermore, H_2_O_2_ production rates were positively correlated with N_3_C content and negatively with N_2_C, emphasizing the importance of N vacancies created during calcination (Figure 42b).

Zhou and colleagues^[^ ^415^ ^]^ introduced an innovative methodology for enhancing the ORR on g‐C_3_N_4_ NSs through P doping and edge engineering. This approach utilized electron‐withdrawing groups to create Lewis basic sites, which significantly bolstered O_2_ chemisorption and activation on the surface of the photocatalyst. Furthermore, the incorporation of anthraquinone via π‐π stacking interactions allowed it to act as an electron reservoir, effectively capturing photoexcited electrons and facilitating their transfer. Upon interaction with O_2_, anthraquinone was selectively converted into H_2_O_2_, subsequently reverting to its original state, thus enabling cyclic H_2_O_2_ production. The combined effects of P and O co‐doping enhanced water oxidation efficiency, allowing for direct H_2_O_2_ generation under visible light without the necessity for external hole scavengers. This strategy achieved an impressive H_2_O_2_ formation rate of 75 mM h^−1^ under open‐air conditions, comparable to both noble metal‐based and metal‐free g‐C_3_N_4_ photocatalysts operating in pure O_2_ environments (Figure 42c).

Huang's group^[^ ^416^ ^]^ implemented a one‐step H2 plasma treatment to fabricate a hierarchical amorphous CN (ACN) nanostructure. This method yielded two N_2_C‐site vacancies per structural unit without the need for foreign dopants. The ACN produced through plasma exhibited a layered NSs architecture, effectively addressing prevalent issues such as aggregation, excessive BG, and constrained UV light absorption. The resultant hierarchical structure, characterized by a reduced BG and an expanded bandwidth, significantly enhanced charge carrier separation due to a pronounced internal potential drop. This advancement led to a marked improvement in photocatalytic H_2_O_2_ production. The study highlights the superior efficacy of this material under visible light irradiation (*λ* > 420 nm) compared to BCN, as evidenced in Figure 42d, emphasizing its viability in sustainable photocatalysis.

Similarly, Huang and colleagues^[^ ^417^ ^]^ synthesized three different CN materials, designated as UCN‐DTDA, UCN‐SDA, and UCN‐TDA. These materials were developed through the integration of specific organic linkers: 5,5‐dioxo‐5 H‐dibenzo[b,d]thiophene‐3,7‐dicarboxylic acid, 4,4′‐sulfonyl dibenzoic acid, and 2,5‐thiophene dicarboxylic acid, respectively. PL and electrochemical assessments demonstrated that the introduction of these linkers significantly improved the separation and distribution of photoinduced charge carriers. EPR and subsequent electrochemical examinations revealed that H_2_O_2_ was generated via a two‐electron pathway during the ORR, achieving a selectivity greater than 90%. Among the fabricated materials, UCN‐DTDA presented the highest photocatalytic rate for H_2_O_2_ production, measured at 7.3 mmol g^−1^ h^−1^, which is nearly three times greater than that of the unmodified CN. Additionally, it exhibited remarkable AQEs of 20.2, 15.2, 9.4, 6.2, and 4.5% at illumination wavelengths of 420, 435, 460, 475, and 520 nm, respectively (Figure 42e).

Wu's team^[^ ^418^ ^]^ has engineered a novel integrated tandem photocatalytic system that enables the simultaneous in‐situ production of H_2_O_2_ and the subsequent epoxidation of propylene. This innovative device employs a g‐C_3_N_4_ photocatalyst featuring N vacancies (N_3_C), which are formed through a combination of Ar pyrolysis and supramolecular self‐assembly. The presence of these vacancies enhances the separation of electrons and holes, thereby boosting the production of reactive O‐species, specifically superoxide (·O_2_^‐^) and singlet oxygen (^1^O_2_), which are critical for efficient H_2_O_2_ synthesis. Additionally, TiSi‐1 (TS‐1) is utilized as a catalyst for the epoxidation of propylene, catalyzing the reaction with photogenerated H_2_O_2_ under UV light exposure and achieving an impressive yield of propylene oxide (PO) at 5515 mM g^−1^ h^−1^, with a remarkable selectivity of 99.1% (Figure 42g). Furthermore, the implementation of a CH_3_OH/H_2_O solvent system contributes to the reaction's efficiency, allowing for direct utilization of H_2_O_2_ without necessitating extraction or purification. CH_3_OH effectively quenches photoexcited holes, thereby minimizing undesirable side reactions, such as the ring‐opening of PO. This photocatalytic approach thus presents a mild and highly selective pathway for synthesizing PO, in stark contrast to traditional commercial methods that often require H_2_ or elevated temperatures (Figure 42f). Overall, the photocatalytic generation of H_2_O_2_ represents a safe, cost‐effective, and environmentally sustainable alternative. Future enhancements in g‐C_3_N_4_ materials through defect engineering, dopant strategies, and functionalization suggest significant promises for industrial‐scale applications.

### 5.7 Photocatalytic CO_2_RR

The increasing levels of atmospheric CO_2_, primarily resulting from industrial emissions, significantly contribute to the intensification of global warming and have become a central theme of climate research.^[^ ^419^ ^]^ Our continued dependence on fossil fuels, including coal and petroleum, further exacerbates the risk of an imminent energy crisis. Photocatalytic CO_2_RR to produce value‐added fuels or chemical intermediates is emerging as a promising strategy to simultaneously combat climate change and energy shortages.^[^ ^420^ ^]^ Various categories of photocatalysts, such as TMOs,^[^ ^421^ ^]^ chalcogenides,^[^ ^422^ ^]^ nitrides,^[^ ^423^ ^]^ phosphides,^[^ ^424^ ^]^ LDHs,^[^ ^425^ ^]^ and nonmetallic semiconductors,^[^ ^426^ ^]^ have been studied for their efficacy in this application, with an emphasis on enhancing activity and selectivity. However, their overall performance is still inadequate for commercial application. Challenges such as insufficient charge carrier transport and a scarcity of reactive sites limit the efficacy of CO_2_ conversion processes.^[^ ^427^ ^]^ To overcome these obstacles, scientists have investigated various modification strategies, including defect engineering, metal deposition, and atomic doping, to optimize the electronic characteristics of photocatalysts.^[^ ^428^ ^]^

**Figure 43:**
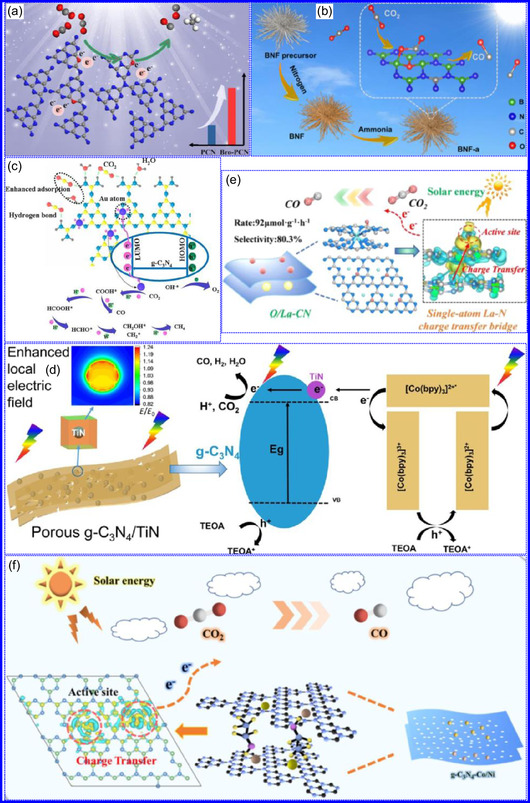
Photocatalytic CO_2_RR behavior of a) Bro‐PCN. Adapted with permission.^[^ ^429^ ^]^ Copyright 2023, Elsevier B.V. b) BNF‐a. Adapted with permission.^[^ ^430^ ^]^ Copyright 2022, American Chemical Society. c) U‐ACN. Adapted with permission.^[^ ^431^ ^]^ Copyright 2020, Wiley‐VCH. d) g‐C_3_N_4_/TiN. Adapted with permission.^[^ ^432^ ^]^ Copyright 2021, Elsevier B.V. e) O/La‐CN. Adapted with permission.^[^ ^433^ ^]^ Copyright 2020, American Chemical Society. f) Co/Ni‐doped g‐C_3_N_4_. Adapted with permission.^[^ ^434^ ^]^ Copyright 2022, Elsevier B.V.

Xu's group^[^ ^429^ ^]^ has created an innovative photocatalyst called Bro‐PCN through a glycolic acid‐assisted thermal polycondensation of urea. This synthesis produced ultrathin NSs featuring a porous structure. Compared to the unaltered PCN, Bro‐PCN displayed a markedly superior photocatalytic behavior in CO_2_RR. This enhancement can be attributed to its distinctive morphology, which promotes increased surface adsorption, improved mass transport, and accelerated charge mobility. Additionally, UV‐visible DRS indicated an expanded photo response, signaling enhanced absorption of visible light. The results also indicated that the CO_2_RR efficiency reached its peak at a specific concentration of glycolic acid, after which performance deteriorated slightly (**Figure** **43**a). In a similar vein, Min and colleagues^[^ ^430^ ^]^ developed a hierarchically structured, ordered BN catalyst (BNF‐a) utilizing a two‐step annealing technique that involved structural ordering and heteroatom doping. BNF‐a exhibited remarkable catalytic performance, achieving CO yields threefold higher than its undoped counterpart (BNF), 7.3 times that of bulk g‐C_3_N_4_, and 26.7 times that of porous BN. This exceptional performance eclipses all previously reported metal‐free BN photocatalysts and demonstrates competitive efficacy against both metal‐free and metal‐doped alternatives. The enhanced activity is predominantly due to the synergistic interaction of B sites with O‐species, which generate highly active sites for CO_2_RR (Figure 43b).

Xiang's team^[^ ^431^ ^]^ introduced an innovative photocatalyst by anchoring atomically dispersed Au onto amino‐functionalized g‐C_3_N_4_ (U‐ACN) through a urea‐mediated reduction strategy under mild and environmentally benign conditions. This system exhibited markedly enhanced photocatalytic performance in CO_2_RR, yielding 1.97‐ and 4.15‐times higher production of CO and CH_4_, respectively, under visible‐light illumination over 2.5 h. The catalytic superiority was ascribed to the atomically dispersed Au, which facilitated CH_4_ generation by lowering the energy barrier, narrowing the BG, and suppressing charge carrier recombination. Furthermore, the amino groups introduced via urea enhanced CO_2_ adsorption, contributing to improved photocatalysis. Comparative evaluations among U‐ACN, P‐ACN, and unmodified CN revealed that U‐ACN achieved the highest yields of CO (21.7 mmol g^−1^) and CH_4_ (2.4 mmol g^−1^) without the use of cocatalysts or sacrificial agents. The CH_4_/CO product ratio for U‐ACN was twice that of pristine CN, indicating a more efficient 8‐electron transfer pathway (Figure 43c). Simultaneously, Chang and colleagues^[^ ^432^ ^]^ synthesized a porous g‐C_3_N_4_/TiN heterojunction via one‐step calcination. The porous architecture enhanced the availability of active sites, while TiN NPs facilitated efficient electron transport and suppressed recombination. Additionally, plasmonic effects from TiN broadened the light absorption range, thereby improving visible light responsiveness. Notably, the TiN NP size was found to critically influence the CO_2_RR productivity (Figure 43d).

Dong's team^[^ ^433^ ^]^ a highly efficient photocatalyst by incorporating single La atoms into g‐C_3_N_4_ (O/La‐CN), achieving notable CO_2_RR activity under visible light. The catalyst attained a CO production rate of 92 μmol g^−1^ h^−1^ and exhibited excellent photostability. DFT calculations, supported by experimental data, revealed that La atoms act as electronic conduits bridging g‐C_3_N_4_ layers, thereby enhancing interlayer charge transfer, facilitating CO_2_ adsorption, and promoting COOH* intermediate formation and subsequent CO desorption. Crucially, the p‐d hybridization involving La 4f and 5d orbitals was instrumental in regulating electron transport within the La‐N bonding framework. Photocatalytic assessments confirmed substantial increases in CO and CH_4_ yields with minimal H_2_ evolution (Figure 43e). In a complementary study, Wu and collaborators^[^ ^434^ ^]^ developed a bimetallic Co/Ni‐doped porous g‐C_3_N_4_ photocatalyst via a bottom‐up strategy involving intercalation with hydrogenated H_3_PO_4_ and metallic precursors, followed by calcination. The dual‐doping configuration significantly enhanced CO evolution compared to undoped g‐C_3_N_4_. This performance gain was attributed to synergistic electronic effects of Co and Ni, which modulated the band structure, improved visible light harvesting, and suppressed charge recombination through the formation of N defects (Figure 43f). These findings underscore the critical role of elemental doping, defect engineering, and heterojunction design in optimizing CO_2_RR performance across a spectrum of semiconductor systems.

### 5.8 Photocatalytic Degradation

The acceleration of industrial activities has significantly intensified the pollution of aquatic ecosystems, particularly through the release of recalcitrant organic contaminants such as synthetic dyes utilized in the textile and leather sectors.^[^ ^435^ ^]^ MB, a frequently employed cationic dye, presents considerable environmental and health hazards due to its intricate molecular structure and inherent resistance to natural degradation mechanisms.^[^ ^436^ ^]^ Although traditional remediation approaches, including adsorption, electrochemical oxidation, and biodegradation, are commonly employed for MB abatement, they suffer from limitations in efficiency and long‐term sustainability.^[^ ^437^ ^]^ Photocatalysis has emerged as a compelling alternative, offering advantages in cost‐efficiency and reusability.^[^ ^438^ ^]^ Nonetheless, conventional photocatalysts like g‐C_3_N_4_ are constrained by rapid electron–hole recombination, insufficient light absorption, and low quantum efficiency.^[^ ^439^ ^]^ To mitigate these drawbacks, multiple enhancement strategies, such as elemental doping, surface modification, defect engineering, and heterojunction construction, have been rigorously investigated.^[^ ^440^ ^]^ Among advanced materials, MOFs, notably NH_2_‐UiO‐66 (Zr‐based), have attracted attention due to their excellent chemical stability and visible‐light responsiveness.^[^ ^441^ ^]^ However, their application is limited by poor charge mobility. Hybridization with semiconductors like g‐C_3_N_4_ has demonstrated synergistic improvements in photocatalytic activity.^[^ ^442^ ^]^ Furthermore, self‐luminescent materials such as SrAl_2_O_4_: Eu^2+^, Dy^3+^ (SAO) provide an innovative solution for maintaining photocatalytic functionality under reduced light conditions, owing to their intrinsic energy storage capacity, rendering them promising for scalable, real‐world applications.^[^ ^443^ ^]^

**Figure 44:**
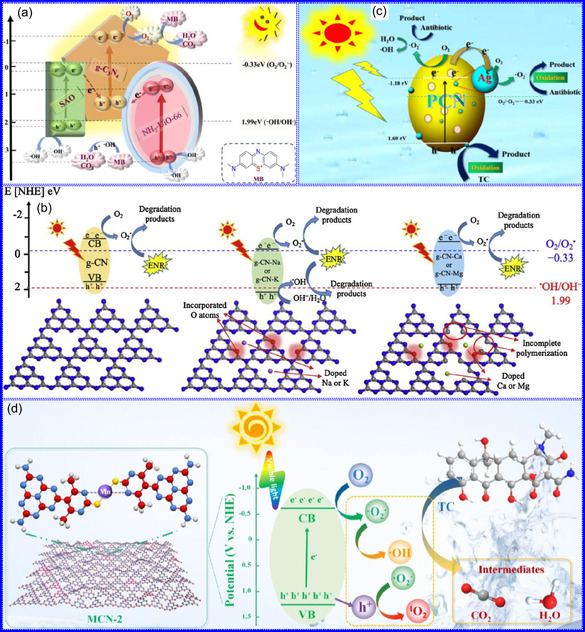
Photocatalytic degradation behavior of a) SGN. Adapted with permission.^[^ ^444^ ^]^ Copyright 2023, Elsevier B.V. b) ENR. Adapted with permission.^[^ ^445^ ^]^ Copyright 2019, Elsevier B.V. c) Ag/PCN. Adapted with permission.^[^ ^446^ ^]^ Copyright 2020, Elsevier B.V. d) MCN‐2. Adapted with permission.^[^ ^447^ ^]^ Copyright 2022, Elsevier B.V.

Cheng's group^[^ ^444^ ^]^ engineered a novel double Z‐scheme ternary photocatalyst, SAO/g‐C_3_N_4_@NH_2_‐UiO‐66 (SGN), via a solvothermal route for the photodegradation of MB. This heterojunction composite exhibited markedly superior visible light absorption and photocatalytic degradation compared to its individual and binary counterparts. Notably, SGN retained catalytic activity in the absence of an external light source, achieving 50% MB degradation over a 5‐h dark period, an indication of intrinsic self‐photocatalytic properties. Comparative analysis demonstrated that incorporation of NH_2_‐UiO‐66 significantly improved the activity of binary systems GN and SN, which achieved 60% and 80% MB degradation, respectively, within 30 min. In contrast, SGN reached 95% degradation under the same conditions, affirming its superior efficacy (**Figure** **44**a). Concurrently, Jing's team^[^ ^445^ ^]^ explored the effects of alkali and alkaline‐earth metal dopants (K^+^, Na^+^, Ca^2+^, and Mg^2+^) on the structural and functional properties of urea‐derived g‐C_3_N_4_. The modified catalysts were evaluated for the photodegradation of enrofloxacin (ENR), tetracycline (TCN), and sulfamethoxazole (SMX) under visible light. Among the doped variants, g‐CN‐K demonstrated the highest activity for ENR degradation, with activity trends observed as: g‐CN‐K > g‐CN‐Na > g‐CN‐Mg > g‐CN‐Ca > g‐CN. These findings underscore the role of cation doping in enhancing ROS generation and modulating photocatalytic mechanisms (Figure 44b).

Wang's team^[^ ^446^ ^]^ synthesized porous plasmonic Ag/g‐C_3_N_4_ (Ag/PCN) photocatalysts through a combination of thermal exfoliation and photoreduction methods. A comprehensive evaluation was conducted on several operational parameters, including catalyst loading, Ag precursor ratios, the presence of inorganic anions, light intensity, and the kinetics of pollutant mineralization. The degradation pathway of TCN was elucidated via LC‐MS, which facilitated the proposal of a plausible degradation mechanism. Among the tested photocatalysts, Ag/PCN‐2 demonstrated notable reusability, maintaining ≈75% of its photocatalytic efficiency after six consecutive cycles, indicating its viability for real‐world wastewater treatment applications (Figure 44c). In a separate investigation, Tao and collaborators^[^ ^447^ ^]^ employed an in‐situ synthesis to incorporate single Mn atoms into the PCN matrix, yielding the MCN photocatalyst. Characterization using HAADF‐STEM and EDS mapping confirmed the atomically uniform dispersion of Mn species. The introduction of Mn enhanced visible light harvesting, improved charge carrier separation, and promoted effective utilization of photogenerated electrons and holes. Photocatalytic assessments using TCN under visible light revealed that MCN achieved 82% degradation within 40 min, with a first‐order rate constant of 0.041 min^−1^, 13.7 times greater than that of undoped UCN. Furthermore, MCN‐2 recorded a TOC removal efficiency of 31%, compared to 12% for UCN, and maintained high catalytic stability across 5 cycles (Figure 44d).

**Figure 45:**
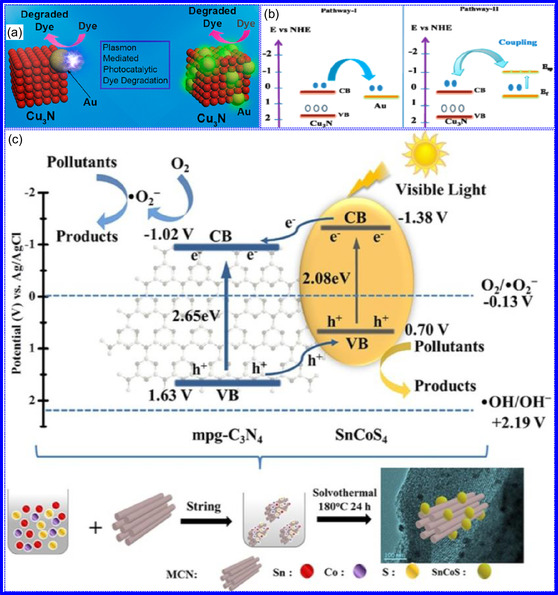
Photocatalytic degradation behavior of a,b) Au‐decorated Cu_3_N NCs. Adapted with permission.^[^ ^448^ ^]^ Copyright 2019, American Chemical Society. c) mpg‐C_3_N_4_/SnCoS_4_. Adapted with permission.^[^ ^449^ ^]^ Copyright 2017, Elsevier B.V.

De's group^[^ ^448^ ^]^ synthesized monodispersed Cu_3_N NCs via an organometallic decomposition route, utilizing Cu_3_N as both the metallic and N source. Surface functionalization strategies enabled colloidal stabilization of the NCs. Au NPs were subsequently anchored onto the Cu_3_N NCs through two approaches: traditional and light‐induced thermal expansion techniques. The latter promoted site‐selective Au deposition due to localized photothermal heating. The presence of LSPR from Au NPs significantly broadened the absorption spectrum into the visible and NIR regions. These Cu_3_N‐Au heterostructures demonstrated superior photocatalytic performance in degrading MB and methyl orange (MO) under visible light irradiation. Kinetic analysis confirmed pseudo‐first‐order reaction behavior, with rate constants (k) derived from linear fits to experimental data (**Figure** **45**a,b).

Li's group^[^ ^449^ ^]^ reported the synthesis of a novel mpg‐C_3_N_4_/SnCoS_4_ heterostructured photocatalyst via an in‐situ hydrothermal method. The photocatalytic efficiency of this material was assessed by the degradation of RhB and MB under visible light irradiation. When compared with its individual constituents and other binary systems, namely mpg‐C_3_N_4_, SnCoS_4_, mpg‐C_3_N_4_/CoS_2_, and mpg‐C_3_N_4_/SnS_2_, the mpg‐C_3_N_4_/SnCoS_4_ heterostructure exhibited markedly superior photocatalytic performance. This improvement was attributed to a higher SSA, enhanced absorption in the visible spectrum, reduced BG, and increased degradation efficiency. The composite achieved degradation rates of 95% for MB and 70% for RhB. The study further investigated the effect of varying mpg‐C_3_N_4_ content on performance and employed radical trapping experiments to elucidate charge‐transfer pathways. In contrast, pure mpg‐C_3_N_4_ and SnCoS_4_ showed minimal activity, with MB degradation efficiencies of 20% and 34%, and RhB degradation of 13% and 18%, respectively (Figure 45c). These findings highlight the potential of advanced g‐C_3_N_4_‐based heterojunctions, enhanced via doping, defect manipulation, and interface engineering, for practical, sustainable photocatalytic water purification under visible and low‐light conditions.

### 5.9 Electrocatalytic HER

The accelerated depletion of fossil fuel reserves, coupled with their adverse environmental impacts, has intensified the pursuit of sustainable energy solutions. H_2_ has emerged as a particularly promising clean energy vector due to its high gravimetric energy density, environmental friendliness, and chemical stability.^[^ ^450^ ^]^ Among the various H_2_ production strategies, electrochemical OWS in alkaline media offers distinct advantages, including operational simplicity and scalability. This process is governed by two fundamental half‐reactions: the HER and OER.^[^ ^451^ ^]^ Although noble metals such as Pt (for HER) and Ir/Ru (for OER) represent current performance benchmarks,^[^ ^452^ ^]^ their high cost, scarcity, and limited long‐term stability impede large‐scale deployment. Accordingly, increasing research efforts have been directed toward the development of cost‐effective alternatives based on TMs, including TMNs, alloys,^[^ ^453^ ^]^ chalcogenides,^[^ ^454^ ^]^ phosphides,^[^ ^455^ ^]^ carbides,^[^ ^456^ ^]^ and nitrides^[^ ^457^ ^]^ for HER and TMOs,^[^ ^458^ ^]^ hydroxides, oxyhydroxides,^[^ ^459^ ^]^ selenides,^[^ ^460^ ^]^ and perovskites^[^ ^461^ ^]^ for OER. Despite notable advances, the inherently sluggish kinetics of the OER, driven by complex steps such as proton‐coupled electron transfer, O—H bond breaking, and O—O bond formation, remain a critical challenge limiting OWS efficiency.^[^ ^462^ ^]^

**Figure 46:**
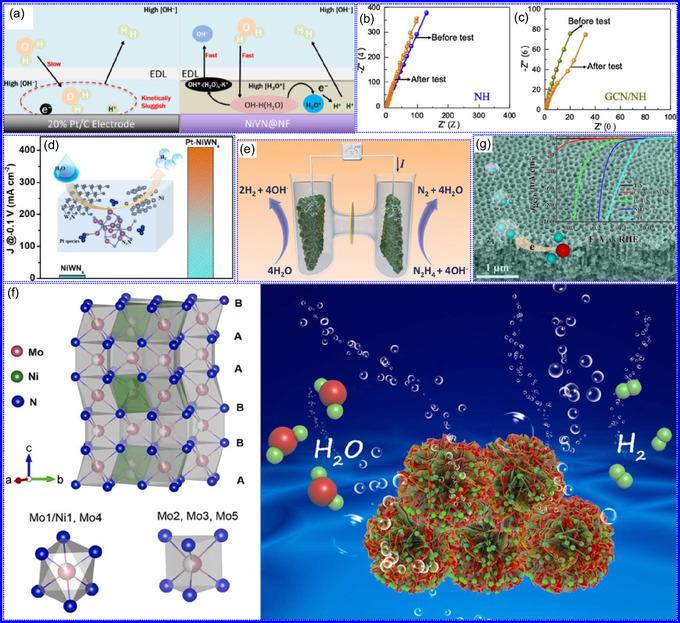
a) Electrocatalytic HER behavior of NiVN@NF and NiCrN@NF oxynitrides. Adapted with permission.^[^ ^463^ ^]^ Copyright 2021, American Chemical Society. b,c) Nyquist curves of GCN/NH composites before and after stability assessment. Adapted with permission.^[^ ^464^ ^]^ Copyright 2022, Elsevier B.V. Electrocatalytic HER behavior of d) Pt‐NiWN_*x*_. Adapted with permission.^[^ ^465^ ^]^ Copyright 2022, Elsevier B.V. e) Cu_1_Ni_2_‐N/CF. Adapted with permission.^[^ ^466^ ^]^ Copyright 2019, Elsevier B.V. f) Ni/Ni_0.8_Mo_4.2_N_6_. Adapted with permission.^[^ ^467^ ^]^ Copyright 2023, Elsevier B.V. g) NiTiN_*x*_@TiN and CoTiN_*x*_@TiN. Adapted with permission.^[^ ^468^ ^]^ Copyright 2020, Elsevier B.V.

Khaled's group^[^ ^463^ ^]^ have synthesized a novel class of binary metal core‐shell nitride@oxynitride electrocatalysts, denoted as Ni‐(ETM)^δ+^‐[O‐N]^δ−^, where ETM represents early TMs such as V and Cr. A key focus of their work was a NiVN catalyst supported on NF, which demonstrated outstanding HER activity in saline environments (0.6 M NaCl, pH 6‐8), requiring only 32 mV overpotential at −10 mA cm^−2^. This enhancement is attributed to improved water dissociation kinetics and high resistance to Cl^−^ induced corrosion. Impressively, the NiVN catalyst maintained operational stability for 100, 50 h each at current densities of −50 and −100 mA cm^−2^, in acidic saline media (1 M phosphate buffer + 0.6 M NaCl). In contrast, a commercial Pt/C@NF catalyst exhibited significant degradation after only 4 h under identical conditions (**Figure** **46**a). Concurrently, Navamathavan's team^[^ ^464^ ^]^ developed g‐C_3_N_4_/Ni(OH)_2_ (GCN/NH) composites via a facile ultrasonication. Structural characterization using XRD, Raman spectroscopy, and FE‐SEM confirmed the formation of ≈20 nm agglomerated NPs. The GCN/NH composite exhibited superior HER performance in alkaline KOH solution, displaying an overpotential of 341 mV at 10 mA cm^−2^, improved from 367 mV for pristine Ni(OH)_2_ NSs, and a reduced Tafel slope of 131 mV dec^−1^, indicating enhanced catalytic kinetics (Figure 46b,c).

Chen's group^[^ ^465^ ^]^ developed an advanced hybrid electrocatalyst, termed NiWN_*x*_, comprising trace amounts of Pt embedded within a mixed TMN matrix of W_2_N, Ni_3_N, and Ni. The strategic incorporation of Pt facilitates enhanced electron coupling with the matrix, while the heterogeneity of the catalyst surface contributes to a high density of active catalytic sites. The inherent high electrical conductivity of TMNs ensures efficient electron transport throughout the material. This catalyst exhibited exceptional HER behavior, with an overpotential of only 61 mV at a current density of 100 mA cm^−2^. Additionally, a remarkable mass activity of 32.8 A mg^−1^ Pt at −0.1 V indicates significant potential for practical applications (Figure 46d). In a separate investigation, Liu and collaborators^[^ ^466^ ^]^ synthesized Cu_1_Ni_2_‐N porous NSs on CF through thermal ammonolysis of a CuNi‐LDH precursor. The resulting 3D architecture delivered excellent bifunctional catalytic activity for both HER and hydrazine oxidation reaction (H_z_OR). Specifically, the Cu_1_Ni_2_‐N catalyst demonstrated an HER overpotential of 71.4 mV at 10 mA cm^−2^ in 1 M KOH, and an ultralow onset potential of 0.5 mV for H_z_OR in 1 M KOH containing 0.5 M hydrazine. When employed as a bifunctional electrode for full‐cell water electrolysis, it achieved 10 mA cm^−2^ at a low cell voltage of 0.24 V and sustained stability over 75 h, attributed to its high conductivity, porous nature, hydrophobic characteristics, and the synergistic interaction of Ni and Cu nitrides (Figure 46e).

Zhu's team^[^ ^467^ ^]^ developed a highly efficient super‐hydrophilic H_2_ evolution catalyst comprising Ni supported on a NiMo‐nitride matrix (Ni/Ni_0.8_Mo_4.2_N_6_), demonstrating catalytic activity superior to that of conventional Pt‐based nanomaterials. While Ni and Ni_0.8_Mo_4.2_N_6_ exhibit only moderate individual catalytic activity, their combination synergistically enhances performance, evidenced by a fivefold (500%) increase in exchange current density and a 150% improvement in TOF relative to commercial Pt/C catalysts. DOS simulations suggest that electron depletion on the Ni support reduces the HER free energy barrier. Kinetic analysis via Tafel slopes yields a value of 38 mV dec^−1^, indicative of the Volmer–Heyrovsky mechanism with electrochemical desorption as the rate‐determining step. Notably, the catalyst achieves an exchange current density (J_0_) of 2.89 mA cm^−2^, 5 times that of Pt/C, and exhibits lower charge transfer resistance (5.2 vs. 7.7 Ω), reflecting enhanced HER kinetics (Figure 46f).

Zhang's group^[^ ^468^ ^]^ synthesized two bimetallic nitride nanoporous matrices, NiTiN_*x*_@TiN and CoTiN_*x*_@TiN, using anodized TiO_2_ NTAs as substrates. Among these, NiTiN_*x*_@TiN exhibited significantly enhanced HER activity in comparison to its monometallic counterparts, thin film analogs, and the CoTiN_*x*_@TiN system. This superior performance is primarily ascribed to the increased density of electrochemically active sites and the conductive TiN nanoporous framework, which simultaneously ensures structural robustness and facilitates electron transport. First‐principles calculations reveal that the unique Ni–Ti atomic coordination improves the adsorption energetics of HER intermediates. Of all evaluated systems, including bare TiN NTAs, compact NiTiN_*x*_ films, CoTiN_*x*_@TiN, and commercial Pt/C, the NiTiN_*x*_@TiN matrix demonstrated the lowest overpotential (125 mV at 10 mA cm^−2^) and the most favorable Tafel slope (71 mV dec^−1^), as shown in Figure 46g. These findings support the advancement of cost‐effective, non‐precious metal alternatives for HER catalysis. TMNs, especially when alloyed or combined with chalcogenides, phosphides, or carbides, offer substantial promise. Notably, early TMs‐integrated Ni‐based oxynitrides and novel compositions such as Cu–Ni and Ni–Mo nitrides have shown impressive HER efficiency and durability, particularly under saline conditions, underscoring their potential for large‐scale H_2_ production.

### 5.10 Electrocatalytic OER

The escalating cost of conventional energy sources, coupled with the adverse environmental effects of fossil fuel dependence, has heightened the global impetus to explore sustainable energy alternatives.^[^ ^469^, ^470^ ^]^ H_2_ is emerging as a promising clean energy carrier, particularly for applications such as fuel cell‐powered transportation, owing to its renewable nature and high purity.^[^ ^471^ ^]^ Among the various H_2_ production technologies, water electrolysis is recognized as a leading method. Nevertheless, its widespread adoption is hindered by the sluggish kinetics of the OER and the substantial overpotentials required at the anode.^[^ ^472^ ^]^ Consequently, there is considerable interest in the development of high‐performance electrocatalysts to enhance OER efficiency and system viability.^[^ ^452^ ^]^

**Figure 47:**
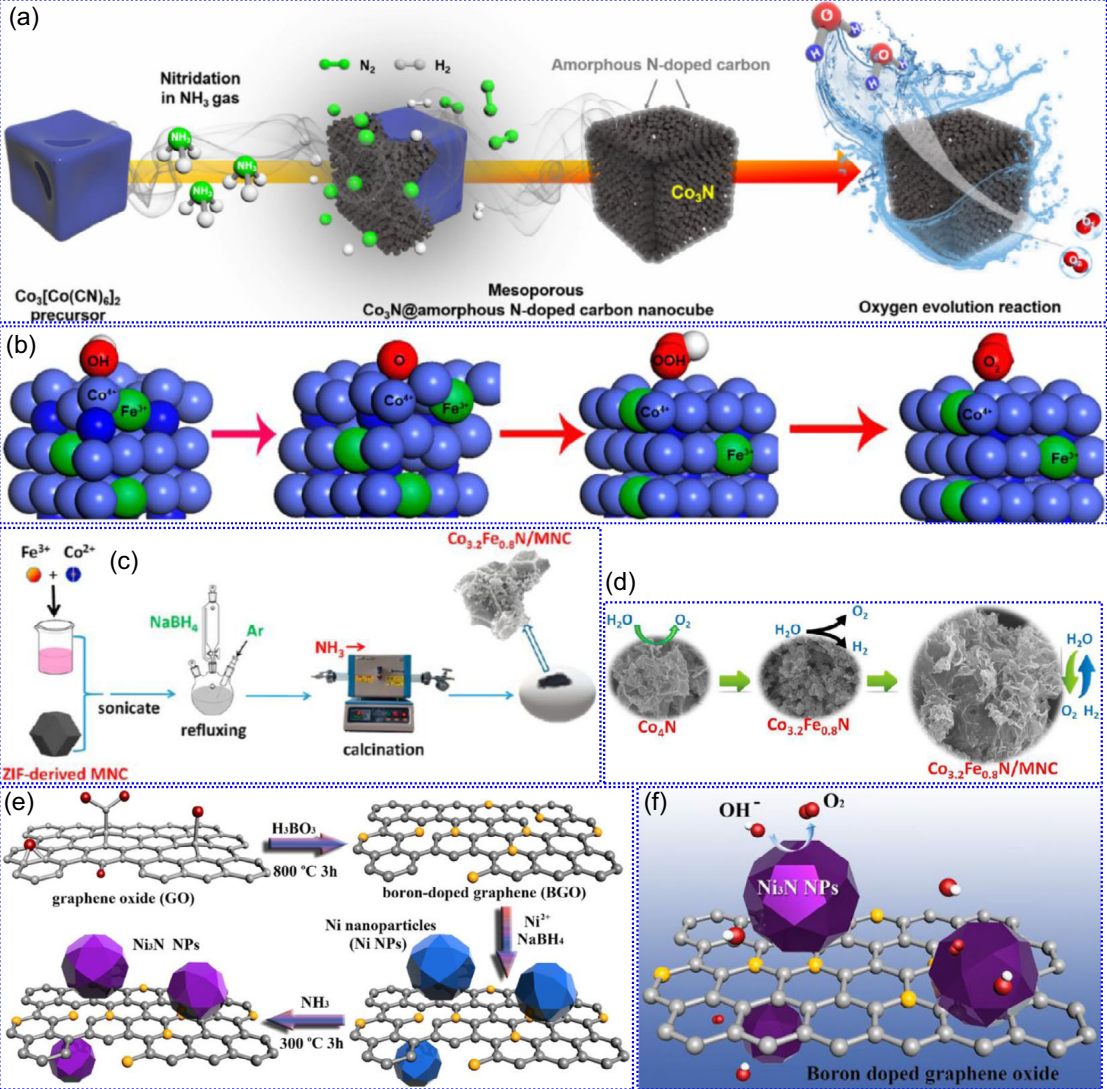
a) Electrocatalytic OER activity of Co_3_N@AN‐C. Adapted with permission.^[^ ^473^ ^]^ Copyright 2019, Springer Nature. b) Co atom of CoFe(3:1)‐N edges in alkaline atmosphere. Adapted with permission.^[^ ^475^ ^]^ Copyright 2018, American Chemical Society. Preparation strategy, and electrocatalytic OER activity of c,d) Co_3.2_Fe_0.8_N/MNC. Adapted with permission.^[^ ^477^ ^]^ Copyright 2019, American Chemical Society. e,f) BGO/Ni_3_N. Adapted with permission.^[^ ^478^ ^]^ Copyright 2020, American Chemical Society.

Lim's group^[^ ^473^ ^]^ reported the synthesis of mesoporous Co_3_N@amorphous N‐C (AN‐C) NCs via in situ nitridation and calcination of Prussian blue analog precursors. The mesoporous structure, coupled with the Co_3_N/AN‐C hybrid composition, enables uniform pore distribution, abundant active sites, and enhanced electrochemical durability. The resulting electrocatalyst demonstrated excellent OER activity, characterized by an overpotential of 280 mV at a current density of 10 mA cm^−2^, a Tafel slope of 69.6 mV dec^−1^, and a C_dl_ of 39 mF cm^−2^. The electrode maintained stable performance at 20 mA cm^−2^ in 1 M KOH over a 24 h operation. Furthermore, a systematic investigation of nitridation duration (ranging from 30 min to 10 h) revealed that a 2 h treatment yielded optimal activity. Compared to Co‐N@N‐C and Co_3_O_4_ NCs, this material exhibited a significantly reduced overpotential (280 vs. 318 and 335 mV, respectively) at 10 mA cm^−2^ (**Figure** **47**a).

Yang's team^[^ ^474^ ^]^ synthesized a 3D mesoporous ternary nitride, NiCo_2_N, via a hard‐template approach employing mesoporous silica (KIT‐6). The resulting material featured a high SSA, uniform mesostructure, and accessible porosity, rendering it a highly efficient OER electrocatalyst under alkaline conditions. NiCo_2_N delivered an overpotential of 289 mV at 10 mA cm^−2^, outperforming benchmark materials such as IrO_2_, CoN, and Ni_3_N. Additionally, it demonstrated a low Tafel slope, reduced charge‐transfer resistance as revealed by EIS, and robust operational stability over 10 h. In a parallel investigation, Zhou and coworkers^[^ ^475^ ^]^ engineered a suite of 3D hierarchically porous Fe‐doped CoN NSs (CoFe(x:y)‐N, with x:y indicating Co/Fe molar ratios). Among these, the CoFe(3:1)‐N variant exhibited superior OER activity, requiring only 200 mV overpotential to achieve 10 mA cm^−2^, coupled with a Tafel slope of 42.44 mV dec^−1^ and a low charge‐transfer resistance (15 Ω). To further assess the role of dopants, a range of CoM(x:y)‐N (M = Ni, Mn, Zn) bimetallic nitrides were synthesized. CoNi(3:1)‐N and CoMn(3:1)‐N exhibited enhanced performance relative to undoped Co_5.47_N, while CoZn(3:1)‐N showed decreased activity. Collectively, these findings affirm the catalytic advantage conferred by Fe doping and the adverse effect of Zn incorporation, establishing CoFe(3:1)‐N as the optimal catalyst in the series (Figure 47b).

Kim's team^[^ ^476^ ^]^ engineered a composite catalyst, denoted as Ir@Co_4_N NFs, consisting of 1D Co_4_N NFs uniformly decorated with finely dispersed Ir NPs to enhance OER efficiency. The Co_4_N NFs backbone promotes effective charge transport and facilitates homogeneous NP distribution. The fabrication included electrospinning Co_4_N followed by thermal annealing and subsequent Ir deposition. The resultant Ir@Co_4_N NFs exhibited superior OER activity and durability in alkaline electrolyte compared to Ir‐loaded Co_3_O_4_ NFs, CNFs, and commercial Ir/C catalysts. In a complementary study, Zhang and colleagues^[^ ^477^ ^]^ synthesized a bimetallic Co‐Fe nitride incorporated into a TM‐N‐C matrix, abbreviated as MNC, via a stepwise modulation strategy (Figure 47c). The integration of Fe into Co_4_N significantly boosted both OER and HER activities, attributed to enhanced electronic interactions arising from Fe's Lewis acidic nature. Furthermore, incorporation of C materials derived from ZIFs into Co_3.2_Fe_0.8_N further improved HER and ORR behavior. The Co_3.2_Fe_0.8_N/MNC hybrid outperformed its pure nitride counterpart in HER and ORR, while maintaining comparable OER activity. This enhancement is ascribed to the generation of Co^4+^ species (critical for OER), additional ORR/HER active sites from the MNC framework, and a tailored nanostructure that ensures improved accessibility and electrochemical stability nanostructure that improves both accessibility and catalyst stability (Figure 47d).

Rao and coworkers^[^ ^478^ ^]^ have reported the development of a high‐efficiency OER electrocatalyst by constructing a p‐n heterojunction between Ni_3_N NPs and B‐doped GO (BGO). The synthesis involved doping GO with H_3_BO_3_ to yield p‐type BGO, which was subsequently dispersed in an aqueous solution containing Ni(NO_3_)_2_. Reduction of Ni^2+^ followed by ammonolysis under NH_3_ resulted in the formation of Ni_3_N nanostructures. The resulting BGO/Ni_3_N composite displayed a high SSA, a rich density of active edge sites, and superior conductivity, attributed to the synergistic interface between the 2D BGO matrix and the embedded Ni_3_N NPs (Figure 47e). In 1 M KOH electrolyte, the composite achieved a low overpotential of 280 mV at 10 mA cm^−2^, surpassing GO/Ni_3_N and Ni/BGO systems, and approaching the performance of RuO_2_. Enhanced OER activity was supported by a reduced Tafel slope (77.9 mV dec^−1^), increased electrochemical SSA, and excellent durability. Long‐term stability assessments confirmed the catalyst's structural robustness via XRD, showing negligible degradation after 24 h at 10 mA cm^−2^ and stable performance at 100 mA cm^−2^ for 10 h (Figure 47f). In summary, considerable progress has been achieved in the design of effective OER catalysts, with recent advances in mesoporous Co_3_N@AN‐C NCs, 3D NiCo_2_N, and Fe‐doped CoN showcasing improvements in activity, overpotential, and longevity through intelligent doping strategies and composite engineering.

### 5.11 Electrocatalytic OWS

Electrocatalytic OWS serves as a pivotal approach for converting surplus electricity from renewable sources, such as solar, wind, and tidal energy, into H_2_ fuel, thereby simultaneously addressing industrial energy demands and mitigating environmental concerns.^[^ ^479^ ^]^ This electrochemical process involves two fundamental half‐reactions: the HER at the cathode and OER at the anode.^[^ ^480^ ^]^ Both reactions are limited by thermodynamic barriers and sluggish kinetics, thereby necessitating the deployment of high‐performance electrocatalysts. While noble metals such as Pt and Ir remain benchmarks due to their high catalytic activity, their limited availability and high cost impede their practical application in large‐scale systems.^[^ ^481^ ^]^ Consequently, significant efforts have been directed toward the development of electrocatalysts derived from earth‐abundant TMs. TMNs have garnered attention due to their intrinsic metallic conductivity and robust chemical stability.^[^ ^482^ ^]^ However, monometallic nitrides typically exhibit catalytic selectivity toward either HER or OER alone. In contrast, bimetallic nitrides, such as Ni_3_FeN, NiCuN, and Co_3_NiN, offer bifunctional activity for OWS, although their performance is frequently hindered by less than‐ideal electronic configurations.^[^ ^483^ ^]^

**Figure 48:**
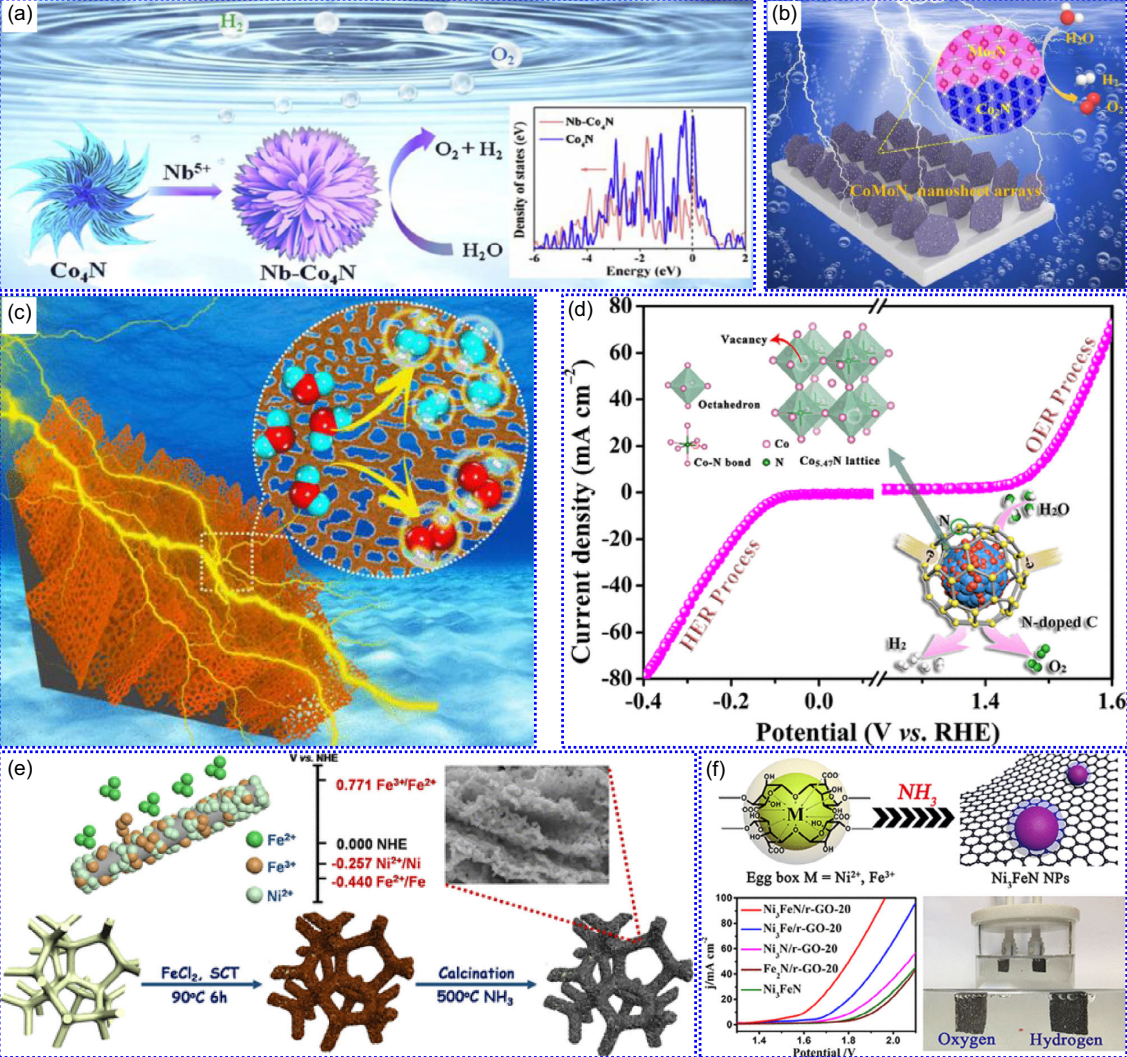
Electrocatalytic OWS behavior of a) Co_4_N/NF and Nb‐Co_4_N/NF. Adapted with permission.^[^ ^484^ ^]^ Copyright 2020, Elsevier B.V. b) CoMoN_*x*_ NSAs/NF. Adapted with permission.^[^ ^485^ ^]^ Copyright 2021, Elsevier B.V. c) CoFeN_*x*_ HNAs/NF. Adapted with permission.^[^ ^486^ ^]^ Copyright 2020, American Chemical Society. d) Co_5.47_N NP@N‐PC. Adapted with permission.^[^ ^126^ ^]^ Copyright 2018, American Chemical Society. e) FeNi_3_N/NF. Adapted with permission.^[^ ^487^ ^]^ Copyright 2016, American Chemical Society. f) Ni_3_FeN/r‐GO. Adapted with permission.^[^ ^488^ ^]^ Copyright 2017, American Chemical Society.

Li and colleagues^[^ ^484^ ^]^ synthesized Nb‐doped Co_4_N NSs supported on a 3D NF substrate (Nb‐Co_4_N/NF) and evaluated their bifunctional catalytic activity toward both the HER and OER. The incorporation of Nb induced significant morphological transformations in Co_4_N, transitioning from NW to multilayered, porous NSs configurations, thereby increasing the exposure of active SSA. This unique nanostructure, characterized by high SSA, metallic conductivity, and mechanical robustness from the 3D scaffold, facilitated enhanced charge transfer kinetics. The Nb‐Co_4_N/NF catalyst demonstrated outstanding HER performance under alkaline conditions and exhibited notable potential for OER catalysis. Optimal doping with 5% Nb yielded substantial improvements in both activity and durability. Electrochemical analyses confirmed that the electrochemical surface area (ECSA) of Nb‐Co_4_N‐0.05/NF significantly exceeded that of the undoped Co_4_N/NF, contributing to superior catalytic behavior. Conversely, higher Nb concentrations led to morphological degradation and NP agglomeration, detrimentally impactingcatalytic efficiency (**Figure** **48**a).

Jiang's group^[^ ^485^ ^]^ developed CoMoN_*x*_ NSAs on NF through a hydrothermal synthesis followed by nitridation. The resulting CoMoN_*x*_ heterojunction, comprising Co_2_N and Mo_2_N, exhibited strong interfacial electronic interactions, which facilitated rapid charge transfer and exposed a high density of active sites for both the OER and HER. The 3D architecture of the NSAs contributed to an increased SSA, improved electrolyte penetration, and efficient gas release, thereby enhancing the OWS performance, as illustrated in Figure 48b. Similarly, Yuan and collaborators^[^ ^486^ ^]^ synthesized a 3D interconnected CoFeN_*x*_ matrix (CoFeN_*x*_ HNAs/NF) on NF using hydrothermal methods followed by thermal nitridation. This design improved the SSA, accelerated electron mobility, and enhanced ion diffusion and gas adsorption. The optimized CoFeN_*x*_‐500 HNAs/NF catalyst demonstrated exceptional electrocatalytic activity, with overpotentials of 200 mV for HER and 260 mV for OER at 10 and 50 mA cm^−2^, respectively. In a two‐electrode OWS setup, a reduced cell voltage of 1.592 V at 10 mA cm^−2^ was observed, surpassing the performance of other TMN electrocatalysts. Under three‐electrode OER evaluation in 1 M KOH, CoFeN_*x*_‐500 HNAs/NF exhibited an overpotential of 259 mV at 50 mA cm^−2^, superior to CoFeN_*x*_‐300 (292 mV), CoFeN_*x*_‐400 (274 mV), and CoFeN_*x*_‐600 (289 mV). In contrast, CoFe‐LDH/NF, Co_2_N/NF, Fe_4_N/NF, and RuO_2_/NF displayed higher overpotentials (361‐369 mV). The Tafel slope for CoFeN_*x*_‐500 HNAs/NF was measured at 57.6 mV dec^−1^, indicating more favorable OER kinetics, as shown in Figure 48c.

Wu's team^[^ ^126^ ^]^ developed a 3D Co_5.47_N NP@N‐PC electrocatalyst using a novel, tunable self‐templating strategy. This catalyst exhibits high electrical conductivity, a large SSA, and a rich density of structural defects, all of which contribute to its superior catalytic performance in OWS. Specifically, it achieved overpotentials of 149 mV for the HER and 248 mV for the OER at a current density of 10 mA cm^−2^. When employed as both the anode and cathode in an alkaline electrolyzer, Co_5.47_N NP@N‐PC significantly enhanced OWS efficiency, underscoring its potential as a bifunctional electrocatalyst. Electrochemical assessments using a three‐electrode configuration revealed a dramatic reduction in HER overpotential compared to Co NP@N‐PC (149 vs. 561 mV), alongside a favorable Tafel slope of 86 mV dec^−1^, demonstrating improved HER kinetics as shown in Figure 48d.

Tang and colleagues^[^ ^487^ ^]^ reported an innovative in situ synthesis for FeNi_3_N nanostructures directly on surface‐etched NF, establishing a robust bifunctional electrocatalyst for OWS. This strategy uniquely leverages commercial NF both as a structural support and as a controlled‐release source of Ni, eliminating the need for supplemental Ni precursors or oxidants. Among tested Fe sources, FeCl_2_ proved superior to FeCl_3_, delivering higher product purity and improved catalytic activity in FeNi_3_N formation. The resulting FeNi_3_N/NF catalyst exhibited excellent HER and OER performance, with overpotentials of 75 and 202 mV, and corresponding Tafel slopes of 40 and 98 mV dec^−1^ at 10 mA cm^−2^. The catalyst also demonstrated exceptional long‐term operational stability, retaining performance for over 400 h under galvanostatic conditions. Durability tests for HER and OER were conducted at ±50 mA cm^−2^, as detailed in Figure 48e.

Yao's group^[^ ^488^ ^]^ engineered highly efficient bifunctional electrocatalysts by incorporating Ni_3_FeN NPs into r‐GO composite aerogels. Utilizing (Ni,Fe)‐alginate precursors with a tailored “egg‐box” configuration, the team systematically adjusted Ni and Fe ratios to construct a porous 3D aerogel. DFT simulations confirmed that covalent Ni–Fe–N interactions enhanced electrical conductivity and optimized electronic surface states, thereby facilitating electron transfer. The Ni_3_FeN/r‐GO‐x composites exhibited outstanding HER and OER activity, reaching 10 mA cm^−2^ at 1.6 V and maintaining performance over 100 h. Notably, Ni_3_FeN/r‐GO‐20 showed the lowest overpotential, earliest onset potential (≈1.48 V vs. RHE), and optimal OER performance (270 mV at 10 mA cm^−2^) in 1 M KOH. HER evaluations revealed an overpotential of 94 mV at 10 mA cm^−2^, surpassing all other tested materials, including Pt/C at elevated current densities (Figure 48f). Collectively, the development of electrocatalysts such as Nb‐Co_4_N/NF, CoMoN_*x*_‐500 NSAs/NF, CoFeN_*x*_‐500 HNAs/NF, Co_5.47_N NP@N‐PC, FeNi_3_N/NF, and Ni_3_FeN/r‐GO‐20 underscores substantial progress in cost‐effective, high‐performance OWS technologies for sustainable H_2_ production.

### 5.12 Electrocatalytic CO_2_RR

The rising atmospheric concentration of CO_2_ has intensified efforts to identify sustainable energy solutions. Among the various strategies, employing CO_2_ as a renewable C feedstock for synthesizing fuels and value‐added chemicals has garnered substantial attention.^[^ ^489^ ^]^ Electrochemical CO_2_RR represents a particularly promising approach; however, its practical implementation is hindered by the high activation energy required to overcome the molecule's inherent thermodynamic stability, resulting in significant overpotentials that negatively affect HER.^[^ ^490^ ^]^ Accordingly, the design of advanced catalysts to reduce the activation barrier remains a critical research focus.^[^ ^491^ ^]^ A broad spectrum of catalysts, including metals,^[^ ^492^ ^]^ metal oxides,^[^ ^493^ ^]^ and molecular complexes,^[^ ^494^ ^]^ has been explored. Among them, Ni‐based materials are particularly notable due to their high selectivity and catalytic efficiency under controlled conditions.^[^ ^495^ ^]^ Nonetheless, a key limitation arises from Ni's concurrent activity in HER, which often competes with CO_2_RR and diminishes its overall effectiveness.

**Figure 49:**
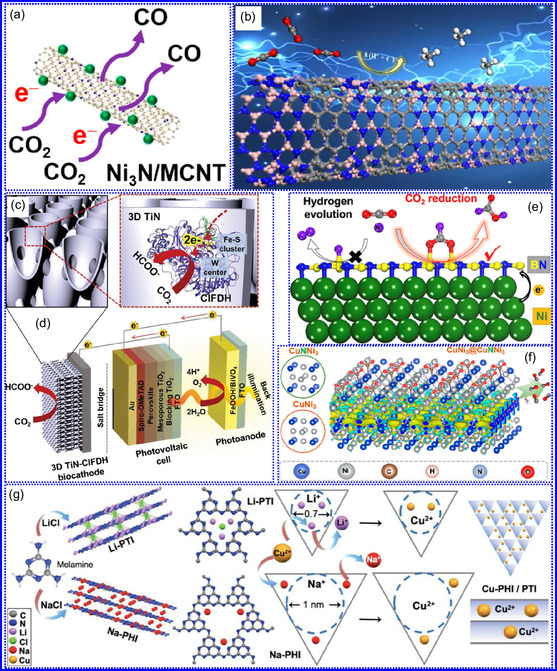
Electrocatalytic CO_2_RR activity of a) MCNT. Adapted with permission.^[^ ^496^ ^]^ Copyright 2019, American Chemical Society. b) TM‐CNT@BNNT. Adapted with permission.^[^ ^497^ ^]^ Copyright 2025, Elsevier B.V. c,d) 3D‐structured TiN nanoshell electrode. Adapted with permission.^[^ ^498^ ^]^ Copyright 2019, Wiley‐VCH. e) h‐BN monolayer. Adapted with permission.^[^ ^499^ ^]^ Copyright 2018, American Chemical Society. f) CuNi_3_@CuNNi_3_/C composite. Adapted with permission.^[^ ^500^ ^]^ Copyright 2022, American Chemical Society. g) Na‐PHI, Li‐PTI, and Cu‐PHI/PTI. Adapted with permission.^[^ ^501^ ^]^ Copyright 2023, Wiley‐VCH.

Kang's group^[^ ^496^ ^]^ have synthesized Ni_3_N/MCNT composites with ultra‐small particle sizes, which display notable efficiency in the electrochemical reduction of CO_2_. These composites exhibit significant selectivity for CO production, even in acidic environments (pH 2.5). The electrocatalytic behavior of Ni_3_N/MCNT toward CO_2_RR was evaluated using CV in a CO_2_‐saturated 0.5 M NaHCO_3_ electrolyte. Among the tested samples, Ni_3_N/MCNT‐1 exhibited superior catalytic current density and a more positive onset potential (–0.35 V vs. RHE) than Ni_3_N/MCNT‐2. Furthermore, Ni_3_N/MCNT‐1 generated a higher current density under CO_2_ conditions than under an Ar atmosphere. Conversely, Ni(OH)_2_/MCNT demonstrated a reduced current response upon CO_2_ exposure, in comparison to Ni_3_N/MCNT. This superior performance of Ni_3_N/MCNT‐1, coupled with a higher exchange current density, highlights improved CO_2_ adsorption and a lower activation barrier, suggesting it is a more effective CO_2_RR catalyst than Ni(OH)_2_/MCNT. The latter, with a Tafel slope of 229 mV dec^−1^, displayed markedly different kinetic behavior (**Figure** **49**a).

In their study, Chen and colleagues^[^ ^497^ ^]^ proposed a heterostructure strategy to improve the catalytic efficiency of SACs for CO_2_ reduction. They engineered a series of catalysts by integrating TMNs, including Cr, Mn, Fe, Co, Ni, and Cu, into heterostructures composed of SWCNTs and BNNTs. These configurations, labeled as TM‐CNT@BNNT and TM‐CNT/BNNT, modulate charge transport and shift the d‐band center, thereby enhancing catalytic activity. Charge transfer showed a moderate linear relationship with CO_2_ adsorption energy, indicating that tuning electron transfer mechanisms can improve CO_2_RR performance. In particular, reduced charge transmission at the metal sites fosters balanced adsorption of intermediates like *OH, as demonstrated in Figure 49b.

Lee's team^[^ ^498^ ^]^ developed 3D TiN nanoshell substrates for biocatalytic PEC cells targeting CO_2_ conversion via direct electron transfer (DET). The TiN electrodes exhibit high conductivity and a porous architecture, supporting efficient DET when integrated with a W‐dependent formate dehydrogenase (ClFDH) from *Clostridium ljungdahlii*. Their extensive SSA, robust electron pathways, and structured porosity facilitate enzyme immobilization and enhance substrate transport. When incorporated into a solar‐driven tandem PEC cell, the ClFDH‐functionalized 3D TiN biocathode achieved a CO_2_‐to‐formate conversion efficiency of 77.3% with an average production rate of 0.78 μmol h^−1^. The study also investigated catalytic performance under varied bias voltages (Figure 49c,d).

Jiang and colleagues^[^ ^499^ ^]^ employed first‐principles calculations to explore the role of a h‐BN monolayer in modulating the electrocatalytic CO_2_RR activity of TMs, specifically Co, Ni, and Cu. Their analysis revealed that electron transfer from the metal substrate to the h‐BN overlayer alters the surface's electronic properties, leading to a substantial decrease in H_2_ adsorption without significantly impacting formate (HCOO) adsorption. This selective modulation enhances the CO_2_‐to‐formic acid (HCOOH) conversion efficiency for h‐BN/Ni and h‐BN/Co systems, while concurrently suppressing the HER. The findings highlight the utility of h‐BN monolayers in tailoring surface reactivity for improved selectivity in electrocatalysis (Figure 49e).

Similarly, Li's group^[^ ^500^ ^]^ synthesized thin nanoshells of anti‐perovskite nitride (CuNNi_3_) encapsulating a metallic CuNi_3_ core, forming CuNi_3_@CuNNi_3_ core‐shell nanostructures within a C‐filled microsphere matrix. Their work emphasizes the significance of nanoscale engineering and surface modulation in enhancing CO_2_RR performance. N‐incorporation into the CuNi_3_ lattice alters surface chemistry and improves catalytic activity. Compared to pristine CuNi_3_ and bulk CuNNi_3_, the CuNi_3_@CuNNi_3_ composite exhibits enhanced CO selectivity and durability. At −0.858 V, CuNi_3_/C achieved a CO FE of 60% with a current density of 1.7 mA cm^−2^, while CuNNi_3_/C reached 70% FE and 8.4 mA cm^−2^. The CuNi_3_@CuNNi_3_/C composite demonstrated superior performance, attaining 96% FE and 3.92 mA cm^−2^, indicating effective HER suppression and significant CO_2_RR enhancement (Figure 49f).

Roy's group^[^ ^501^ ^]^ synthesized robust 2D anionic frameworks, poly(heptazine imide) (PHI) and poly(triazine imide) (PTI), capable of hosting high‐density Cu single‐atom sites (≈1.5 at.%) via a room‐temperature ion exchange process. The distinct heptazine and triazine units allowed precise control over Cu–Cu site spacing, promoting intermediate stabilization and enhancing the rate‐determining steps in CO_2_ reduction to CH_4_. PTI, with superior metal‐site contact and optimized coordination environments, exhibited a reactor efficiency of ≈68% and a limiting current density of 348 mA cm^−2^ at −0.84 V vs. RHE. At milder potentials (–0.6 to −0.65 V), Cu‐PHI favored H_2_ evolution (FE 40–50%), while Cu‐PTI primarily produced CO (FE 30–40%), attributed to differing N‐C site densities. At more negative potentials, both catalysts shifted toward CH_4_ production, achieving peak FEs of 54% (Cu‐PHI, −0.88 V) and 68% (Cu‐PTI, −0.84 V). These findings highlight significant progress in electrocatalytic CO_2_RR through tailored catalysts that address key challenges such as HER suppression and activation energy barriers. Further advances, e.g., Ni_3_N/MWCNT composites, heterostructures with metal NPs and SWCNTs, 3D TiN shells, and Cu SACs on CN scaffolds, demonstrate the potential of engineered nanostructures for selective CO_2_ conversion to CH_4_ and HCOOH.

### 5.13 Solar Cells

Solar energy presents a viable and sustainable alternative to fossil fuels, with solar cells playing a pivotal role in photovoltaic energy conversion.^[^ ^502^ ^]^ Silicon (Si), historically central to first‐generation solar cells, remains prominent due to its semiconducting properties and thermal stability, achieving practical efficiencies near 27%.^[^ ^503^ ^]^ Nonetheless, the intricate and costly purification process for crystalline Si has driven research toward alternative materials and designs. Second‐generation solar cells have prioritized cost reduction and flexibility, while third‐generation technologies, particularly dye‐sensitized solar cells (DSSCs), have gained attention for their simple architecture, low production costs, and competitive power conversion efficiency (PCE).^[^ ^504^ ^]^ DSSCs also offer advantages in terms of manufacturability, scalability, and environmental adaptability, enhancing their commercial viability. Recent innovations in nanocrystalline materials have further elevated the efficiency and performance of DSSCs.^[^ ^505^ ^]^

**Figure 50:**
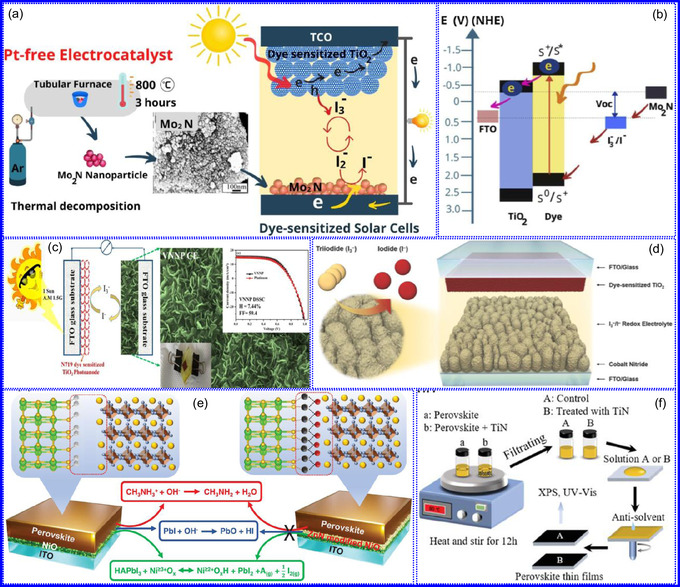
a,b) Diagram illustrating the DSSC device fabrication with Mo_2_N CE. Adapted with permission.^[^ ^506^ ^]^ Copyright 2021, Elsevier B.V. c) Planned working mechanism of VNNP. Adapted with permission.^[^ ^507^ ^]^ Copyright 2021, Elsevier B.V. d) Diagram illustrating the DSSC fabrication employing CoN as the CE which catalyzes the electrochemical reduction of tri‐iodides. Adapted with permission.^[^ ^508^ ^]^ Copyright 2018, Wiley‐VCH. e) Reactive degrading activity of the perovskite with NiO_*x*_ interaction. Adapted with permission.^[^ ^509^ ^]^ Copyright 2024, Wiley‐VCH. f) Diagram evaluating the chemical inertness of TiN with perovskite. Adapted with permission.^[^ ^510^ ^]^ Copyright 2023, Wiley‐VCH.

Jeong's group^[^ ^506^ ^]^ synthesized crystalline Mo_2_N powder using a high‐temperature combustion method of molecular precursors. Electrochemical characterization revealed that Mo_2_N demonstrates catalytic activity and charge transfer resistance comparable to Pt counter electrodes (CEs) in DSSCs. The Mo_2_N‐based DSSCs achieved a PCE of 5.3%, approaching the 6.4% efficiency of Pt‐based counterparts under identical testing conditions, highlighting Mo_2_N as a cost‐effective alternative to Pt (**Figure** **50**a,b). In a parallel effort, Grace and coworkers^[^ ^507^ ^]^ developed VN nanopetals (VNNPs) via a template‐free synthesis followed by high‐temperature ammonolysis. The vertically aligned, petal‐like morphology of VNNPs increased the density of electrocatalytic active sites and facilitated effective I_3_^−^ reduction and charge transfer. When used as CEs in DSSCs, VNNPs achieved a superior PCE of 7.44%, slightly outperforming Pt‐based devices (7.38%). Nyquist analysis further confirmed their high conductivity, with comparable series resistances (*R*_s_) of 10.15 Ω for VNNPs and 10.12 Ω for Pt (Figure 50c).

Kim's team^[^ ^508^ ^]^ synthesized CoN nanofilms via vapor deposition at room temperature under a N_2_ atmosphere, presenting a cost‐effective substitute for Pt in DSSCs. These nanofilms demonstrate PCEs comparable to Pt‐based systems. In inverted planar perovskite solar cells (PSCs), CoN sheets achieved efficiencies of up to 15%, aligning with those of advanced PSCs employing inorganic hole transport layers. Notably, the ambient‐temperature synthesis renders CoN suitable for flexible solar devices (Figure 50d). In addition, Na and colleagues^[^ ^509^ ^]^ investigated degradation phenomena at the NiO_*x*_/perovskite interface in PSCs, attributing them to redox activity involving Ni^3+^ ions and surface hydroxyls. To mitigate this, a Zn_3_N_2_ layer was deposited on NiO_*x*_ via a Zn(NO_3_)_2_‐based treatment, increasing the Ni^3+^/Ni^2+^ ratio, reducing hydroxyl presence, and improving energy level alignment. This modification led to enhanced charge extraction and suppressed recombination, yielding a peak PCE of 21.62%. The Zn_3_N_2_‐modified PSCs maintained 82% of initial efficiency after 1000 h in ambient conditions, and 73% and 54% after 500 and 250 h under continuous illumination at 85 °C, respectively. These findings highlight the utility of TMNs like Zn_3_N_2_ in improving PSC performance and durability (Figure 50e).

The study by Meng's group^[^ ^510^ ^]^ explored the application of TiN as a conductive component in the CE of hole‐transport‐material‐free mesoscopic PSCs (p‐MPSCs). The integration of TiN substantially reduced electrode resistivity from 20 to 10 mΩ cm^−1^ and improved charge transfer at the electrode‐perovskite interface by enhancing surface wettability. Additionally, TiN exhibited excellent chemical stability, showing no adverse interactions with halide perovskites during device fabrication. These advantages led to a high PCE of 19.01%, surpassing that of C black (CB) counterparts. The study highlights the potential of TiN NPs to advance printed CE designs in scalable PSC architecture (Figure 50f).

In summary, TMNs present a promising and cost‐effective alternative to traditional materials like Pt in DSSCs and PSCs. Studies reveal that compounds such as Mo_2_N and VNNPs exhibit electrochemical and photovoltaic performance comparable to, or surpassing, that of Pt. Additionally, materials like CoN nanofilms and Zn_3_N_2_‐modified NiO_*x*_ layers have been shown to enhance the operational stability and overall efficiency of PSC devices. The use of TiN in CEs further exemplifies TMNs capacity to improve charge extraction and device performance. Beyond photovoltaic applications, TMNs demonstrate wide‐ranging industrial relevance, as summarized in **Table** **2**, including catalysis (e.g., hydrogenation and NH_3_ synthesis), protective coatings, photocatalysis, superconductivity, energy storage systems (e.g., batteries and SCs), and environmental sensing technologies.

**Table 2:** A table demonstrating the real‐world applications of TMNs.

S. No.	TMNs	Application	Analysis	Investigation Details	References
1	MoN, VN, TaN	Hydrogen production (WS)	“Electrocatalysis, WS, HER”	HER, OER, “sustainable energy”	[524, 525]
2	TiN, TaN, WN	Batteries and SCs	“Electrode materials” “Electrical conductivity”	“Charge–discharge cycles,” “Energy storage”	[512, 519]
3	TiN, ZrN, WN	Electronics and semiconductors	“Semiconductor devices,” “thin‐film coatings”	“Semiconductor compatibility,” excellent conductivity”	[149, 526]
4	TiN, TaN, MoN	Solar energy conversion	“Photocatalytic systems”	“Solar energy harnessing,” “Sustainable chemical processes”	[138, 527]
5	NiN, MoN	Electrochemical sensors	“Environmental sensors,” “gas sensors”	“Environmental monitoring”	[212, 528]
6	TiN, AlN, CrN	Wear‐resistant coatings	“Wear resistance,” “hard coatings”	“Abrasion,” “stable coatings”	[529, 530]
7	TiN, ZrN, CrN	Optical coatings	“High reflectivity”	“Optical enhancement,” “high reflectivity”	[531, 532]
8	TaN, NbN	Superconductors	“Advanced electronics,” “superconducting properties”	“Superconducting magnets”	[533]
9	WN, CrN, TiN	Surface hardening	“Stability enhancement”	“Heavy‐duty coatings,” “hardness,” “wear resistance”	[529, 534, 535]

## 6 Future Perspectives and Conclusions

TMNs display a wide range of crystal structures, each playing a critical role in determining their physicochemical behavior and long‐term stability. This structural diversity equips TMNs with the capacity to perform reliably across numerous applications, especially in environments requiring durability under fluctuating conditions. In this review, we provide a thorough and comprehensive examination of TMNs, placing particular emphasis on their multifunctional capabilities, intricate morphologies, and structural dimensionality. The scope and analytical depth of our assessment offer readers valuable insights into the fundamental properties and broad technological potential of these materials. We highlight that the pivotal role TMNs can play in driving progress across both scientific inquiry and engineering innovation.

Our principal conclusions are summarized as follows: 1) TMNs exhibit outstanding catalytic performance, marked by high activity, excellent selectivity, and strong resistance to sintering. These properties allow them to operate effectively at lower temperatures than many conventional catalysts. Their enhanced catalytic efficiency stems from their distinctive crystal architectures and electronic structures, which contribute to increased chemical reactivity and thermal stability. 2) DFT investigations reveal that TMNs possess a rich spectrum of properties, including electronic, optical, vibrational, plasmonic, and mechanical characteristics, underscoring their versatility in a wide range of advanced applications. These attributes enable TMNs to deliver consistent performance across a broad spectrum of functional applications. Furthermore, their adaptability is enhanced by variations in both bulk and surface structures, as well as magnetic properties. The dimensional range of TMNs, from 0D to 3D architectures, also plays a crucial role in optimizing their behavior for specific use cases. 3) TMNs can be systematically classified into mono‐, bi‐, tri‐, and multimetallic nitrides, each offering distinct functional benefits. For instance, materials such as FeN, CoN, and TiN exhibit unique properties and application potential due to differences in their elemental composition and crystallographic configuration. 4) Various synthesis methods, including ammonolysis, CVD, electrodeposition, and pyrolysis, allow precise control over the composition, structure, and morphology of TMNs. This level of tunability is essential for engineering materials tailored to meet specific technological demands.

In addition, post‐synthesis modifications such as the incorporation of elements or compounds like B, graphene, and Pt, can markedly enhance the catalytic efficiency, chemical robustness, and overall performance of TMNs. These functionalization strategies expand their applicability across a wide array of advanced technologies. 5) TMNs also exhibit diverse morphologies, including NF, NS, and NTs, each playing a vital role in dictating their physicochemical behavior and performance. By engineering these morphologies with precision, their functional attributes can be fine‐tuned to align with specific application demands. Altogether, TMNs showcase exceptional performance in multiple fields. In photocatalysis, they have proven effective in processes such as the HER, OER, OWS, H_2_O_2_ production, CO_2_ reduction, and environmental cleanup. Within electrocatalysis, their efficiency in HER, OER, and OWS is particularly noteworthy. Beyond catalysis, TMNs have also demonstrated strong potential in energy storage and conversion systems, including batteries, SCs, and solar cells. In conclusion, this review highlights the immense potential of TMNs as versatile, multifunctional materials. Their inherent structural tunability, excellent catalytic performance, and high stability make them strong contenders for driving the development of next‐generation, sustainable technologies.

Although substantial progress has been made in synthesizing TMNs with diverse structures, morphologies, functionalities, and dimensionalities for a wide range of applications, several critical challenges remain. One of the foremost issues is the development of synthesis methods that can reliably produce TMNs with high yield and purity, while also being scalable and reproducible across different fabrication techniques.

### 6.1 Future Research Directions

#### 6.1.1 Heterostructure Optimization

Future research should prioritize the design and fabrication of TMN‐based heterostructures that feature improved interfacial synergy with complementary materials, including metals, oxides, and carbides. Strengthening these interfaces is key to enhancing both electrochemical and catalytic activity. In particular, fine‐tuning the N/M ratio has been shown to significantly affect catalytic performance, especially in critical processes such as the HER and OER.

#### 6.1.2 Advancements in Synthesis Techniques

The evolution of synthesis methodologies will be central to the continued development of advanced TMNs. Among these, ammonolysis has demonstrated effectiveness in achieving better dispersion and enhanced stability of metal NPs within nitride frameworks, leading to superior performance in HER applications. Concurrently, CVD remains a powerful tool for fabricating high‐quality van der Waals heterostructures, such as graphene/h‐BN composites, which hold great promise for future applications in nanoelectronics and energy‐related devices.

#### 6.1.3 Photocatalytic HER

TMNs such as MoN, TiN, and VN are gaining significant attention for their promising performance in photocatalytic HER, attributed to their strong catalytic activity, efficient UV light absorption, and excellent chemical stability. Their H_2_ production efficiency can be further enhanced by forming composites with materials like g‐C_3_N_4_ and other advanced hybrid systems, resulting in more effective and durable H_2_ generation platforms.

#### 6.1.4 Photocatalytic Water Purification

Progress in photocatalytic water treatment is expected to focus on boosting the efficiency of catalysts like g‐C_3_N_4_ through methods such as element doping, defect engineering, and the construction of heterojunctions. Composite systems that combine g‐C_3_N_4_ with MOFs, luminescent materials, or metal NPs have demonstrated superior photocatalytic activity under visible light. These systems show great potential for degrading persistent pollutants, including dyes and antibiotics, even in environments with limited light exposure.

#### 6.1.5 Photocatalytic OWS

Recent advancements in photocatalytic OWS focus on strategies such as elemental doping, structural modification, and the creation of heterojunctions with materials like MOFs and metal NPs. Cutting‐edge nanostructures, including quadruple‐band InGaN NWs and Zn‐doped polymeric CNs, have shown enhanced light‐harvesting capabilities and improved charge carrier separation, paving the way for scalable and efficient H_2_ production systems.

#### 6.1.6 Photocatalytic H_2_O_2_ Production

Developing greener and more efficient methods for H_2_O_2_ generation is also a growing research priority. Modifications to g‐C_3_N_4_, including N‐deficiency engineering, P doping, and ligand grafting, have significantly boosted photocatalytic performance. Furthermore, tandem photocatalytic platforms that simultaneously generate H_2_O_2_ and valuable byproducts such as propylene oxide present exciting opportunities for sustainable industrial‐scale chemical production.

#### 6.1.7 Improvements in LSBs

TMNs are emerging as promising materials to overcome key limitations in LSB technology. Structures such as sulfur NDs, VN NWs, and C‐doped (Ni/Co)_3_N frameworks are being explored for their ability to enhance cycle life, suppress Li dendrite growth, and minimize internal resistance, factors that collectively contribute to improved battery efficiency and stability.

#### 6.1.8 Electrocatalytic OWS

Progress in electrocatalytic OWS depends heavily on the development of cost‐effective, non‐noble metal catalysts that offer high activity and robust long‐term performance. Promising recent materials include Nb‐Co_4_N grown on NF, CoMoN_*x*_‐500 NSAs/NF, and Ni_3_FeN supported on r‐GO. These systems demonstrate lower overpotentials, accelerated reaction kinetics, and enhanced structural integrity, bringing the technology closer to commercial water‐splitting applications.

#### 6.1.9 Solar Energy Applications

TMNs are gaining recognition as promising substitutes for costly noble metals in solar cell technologies, owing to their superior electrical conductivity, catalytic efficiency, and chemical durability. Nanostructured materials like Mo_2_N and VN nanopetals have demonstrated performance comparable to, or even surpassing, that of Pt in photovoltaic devices. Additionally, recent advancements, such as CoN nanofilms and Zn_3_N_2_‐modified NiO_*x*_ layers in PSCs, along with TiN‐based CEs, highlight the potential of TMNs to enhance device longevity, optimize charge transport, and boost overall solar energy conversion efficiency.

## Conflict of Interest

The authors declare no conflict of interest.
